# Computational characterization of enzyme-bound thiamin diphosphate reveals a surprisingly stable tricyclic state: implications for catalysis

**DOI:** 10.3762/bjoc.15.15

**Published:** 2019-01-16

**Authors:** Ferran Planas, Michael J McLeish, Fahmi Himo

**Affiliations:** 1 Department of Organic Chemistry, Arrhenius Laboratory, Stockholm University, SE-10691 Stockholm, Sweden; 2 Department of Chemistry and Chemical Biology, Indiana University-Purdue University Indianapolis, Indianapolis IN 46202, USA

**Keywords:** binding site, DFT, enzyme mechanism, quantum chemical calculations, ThDP-dependent

## Abstract

Thiamin diphosphate (ThDP)-dependent enzymes constitute a large class of enzymes that catalyze a diverse range of reactions. Many are involved in stereospecific carbon–carbon bond formation and, consequently, have found increasing interest and utility as chiral catalysts in various biocatalytic applications. All ThDP-catalyzed reactions require the reaction of the ThDP ylide (the activated state of the cofactor) with the substrate. Given that the cofactor can adopt up to seven states on an enzyme, identifying the factors affecting the stability of the pre-reactant states is important for the overall understanding of the kinetics and mechanism of the individual reactions.

In this paper we use density functional theory calculations to systematically study the different cofactor states in terms of energies and geometries. Benzoylformate decarboxylase (BFDC), which is a well characterized chiral catalyst, serves as the prototypical ThDP-dependent enzyme. A model of the active site was constructed on the basis of available crystal structures, and the cofactor states were characterized in the presence of three different ligands (crystallographic water, benzoylformate as substrate, and (*R*)-mandelate as inhibitor). Overall, the calculations reveal that the relative stabilities of the cofactor states are greatly affected by the presence and identity of the bound ligands. A surprising finding is that benzoylformate binding, while favoring ylide formation, provided even greater stabilization to a catalytically inactive tricyclic state. Conversely, the inhibitor binding greatly destabilized the ylide formation. Together, these observations have significant implications for the reaction kinetics of the ThDP-dependent enzymes, and, potentially, for the use of unnatural substrates in such reactions.

## Introduction

**Scheme 1:**
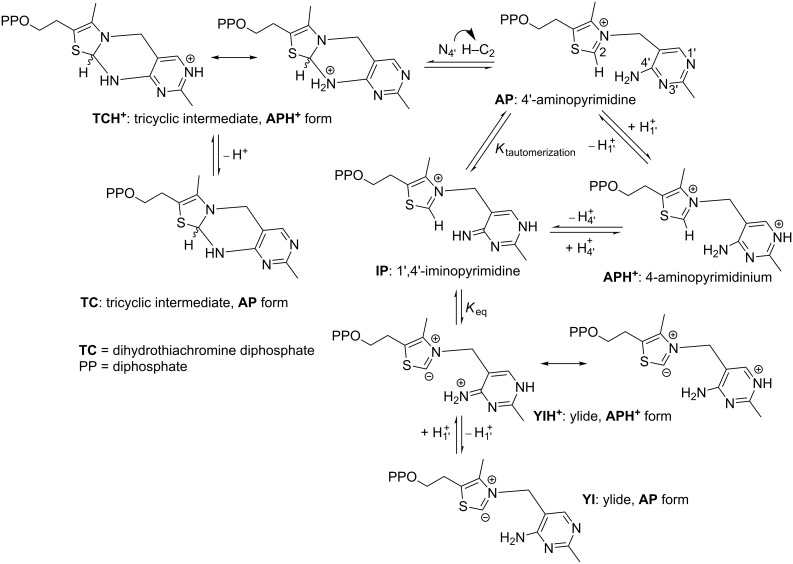
The variety of forms of enzyme-bound ThDP.

Enzymes that depend on thiamin diphosphate (ThDP, Scheme 1) can be found in a wide range of metabolic pathways. Although they are known to catalyze the formation of C–N, C–O and C–S bonds, ThDP-dependent enzymes generally catalyze the breakdown and formation of C–C bonds adjacent to a carbonyl group [1–2]. The resultant 2-hydroxyketones are often chiral, so these enzymes are being increasingly studied for their use as biocatalysts in the preparation of pharmaceuticals and agrochemicals [3]. ThDP is an unusual cofactor in that, even without the enzyme, it can catalyze many of these reactions [2]. For example, the decarboxylation of pyruvate in water can be accomplished by ThDP, but when it is bound to the enzyme pyruvate decarboxylase (PDC), the decarboxylation rate is increased by 12 orders of magnitude [2,4]. Clearly, the catalytic power of the cofactor is greatly enhanced by the enzyme-bound environment. A fundamental understanding of how this enhancement is achieved could potentially lead to the development of new and improved biocatalysts.

At a minimum, ThDP-catalyzed reactions all require the formation of a C2-carbanion or ylide [5] (Scheme 1). This is achieved through a series of proton transfers during which several different states of the cofactor are formed [6]. Starting from the neutral form of ThDP (**AP**), the cofactor can be protonated at the N1′ position, resulting in the **APH^+^** state. With only one known exception [7], the protonation/deprotonation of the N1′ position is performed by a highly conserved glutamic acid residue that is thought to stabilize the imino tautomer **IP** [6]. The subsequent loss of a proton from N4′ of **APH^+^** gives the **IP** state. Deprotonation of the C2 position results in the ylide form which can be either protonated (**YIH^+^**) or deprotonated (**YI**) at the N1′ position. The C2 deprotonation is believed to be performed by the N4′ nitrogen [2,8–9], and is assisted by the cofactor being held in a “V” conformation in which the imino group is located within a hydrogen bonding distance of the C2 of the thiazolium ring [9–14].

While the importance of the catalytically critical ylide was readily recognized, obtaining evidence for the participation of the 4′-amino group and the imino tautomer **IP** proved more challenging. Initially, model compounds were used to identify the signature UV absorbances for the **IP** form of ThDP. These were then used to demonstrate the presence of **IP** on yeast PDC [15]. Subsequently, the **IP** form was shown to have a positive CD signal around 300–310 nm, while a negative peak around 320–330 nm, similar to that observed upon binding of ThDP to apo transketolase [16], was assigned to the **AP** form [15]. These, along with signature CD and UV signals for intermediates further along the reaction pathway, have now been observed for more than 10 ThDP-dependent enzymes [6,17]. As yet, no electronic signature has been observed for the **APH^+^** form. However, solid-state NMR using ^15^N and ^13^C-labeled ThDP has been used to identify **APH^+^** on pyruvate decarboxylase and the E1 component of the pyruvate dehydrogenase complex [18].

In addition to the plethora of experimental investigations, a number of computational studies have addressed issues regarding the various states of ThDP. For example, in some very early work, Jordan used semi-empirical methods to study the electronic structure and conformational space of the cofactor in the gas phase, acknowledging the difficulty of comparing these results to reactions in solution and on the enzyme [19–20]. Thirty years later, density functional theory (DFT) calculations showed that the 4′-amino moiety of the cofactor can either accept or donate a proton in the reactions, depending on the protonation state of N1′ [21].

Orbital analysis of the **IP**/**YIH^+^** reaction showed that full formation of ylide was dependent on deprotonation of N1′ and, consistent with experimental findings, deprotonation was, in turn, likely dependent on conformational changes induced by the presence of substrate [22]. More recently, the relative stabilities of a number of the ThDP states (**AP**, **APH^+^**, **IP** and **YI**) were obtained using DFT methods, employing a model of the cofactor along with the hydrogen-bonding carboxylate moiety [23]. Subsequently, a similar approach was used to characterize the nucleophilicity of the N1′ and N4′ centers [24]. In many cases, rather than simply focus on the cofactor, computational studies have been used to investigate full reaction mechanisms of ThDP enzymes, including pyruvate decarboxylase (PDC) [25–28], benzoylformate decarboxylase (BFDC) [29–30], acetohydroxy acid synthase [24,31–35], pyruvate dehydrogenase (PDH) [36], benzaldehyde lyase [37], cyclohexane dione hydrolase [38], oxalyl-CoA decarboxylase [39], DXP synthase [40] and transketolase [41–42].

It is surprising that almost none of these studies acknowledged that there is a second, albeit less well discussed, path for the ThDP cofactor, i.e., the formation of a tricyclic, dihydrothiachromine species from the **AP** form [43–45]. Nucleophilic attack of N4′ on C2 results in the formation of a C2–N4′ bond, giving rise to the tricyclic intermediate **TCH^+^**. Loss of the N1′ proton **TCH^+^** will result in the **TC** form of the cofactor [45]. While admittedly not common, the tricyclic form of the cofactor has been observed on at least two ThDP-dependent enzymes. Dihydrothiachromine diphosphate (**TC**) was observed in the X-ray structure of phosphoketolase from *Bifidobacterium breve* [46], and its hydroxyethyl derivative was identified in the structure of acetolactate synthase from *Klebsiella pneumoniae* whose crystals had been soaked with pyruvate [47].

In a very recent study, we used quantum chemical methodology to investigate the detailed reaction mechanism of benzoylformate decarboxylase (BFDC) [29]. A model of the active site was designed on the basis of the X-ray structure of BFDC in complex with the substrate analog inhibitor, (*R*)-mandelate. In that study all intermediates and transition states were located and characterized. Intriguingly, we identified the tricyclic **TCH^+^** state of the cofactor as an off-cycle intermediate species. It was found to be about 5 kcal/mol lower in energy than the **IP** state, thereby raising the barrier for the formation of the cofactor–substrate adduct (C2α-mandelyl–ThDP). Of course this has important implications for the overall kinetics of any BFDC-catalyzed reaction and, potentially, for all THDP-dependent enzymes [29].

This unexpected result prompted us to conduct a systematic study of the energetics of the various enzyme-bound states of ThDP (Scheme 1). To this end, we have used BFDC as a representative ThDP-dependent enzyme, and employed the quantum chemical approach used to study the BFDC reaction mechanism to characterize the various states of the ThDP cofactor. Models representing different enzymatic and non-enzymatic environments have been generated and, for each model, the cofactor has been characterized in terms of energies and geometries.

## Results

**Figure 1:**
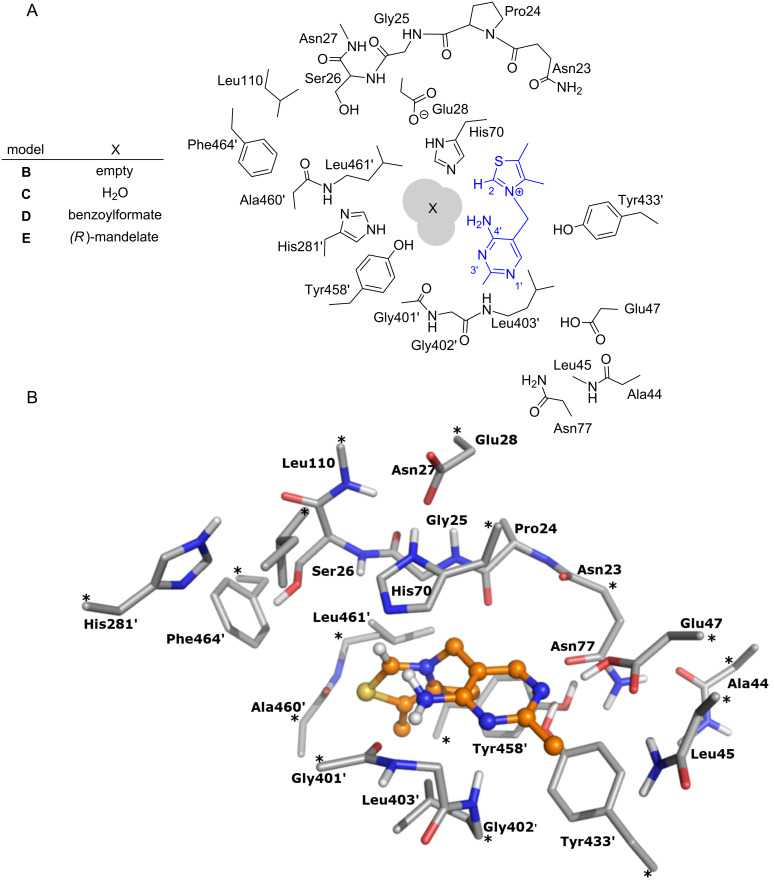
A) 2D representation of ThDP (blue) and the residues included in the active site models, and B) optimized structure of model **B** with an empty active site. Asterisks mark atoms that were kept fixed to their crystallographic positions during the geometry optimization. The BFDC active site has contributions from two monomers and primes re-used to indicate residues from the second monomer. For clarity, the non-polar hydrogens of the residues are not included.

The various states of the cofactor have been studied using five different models. In all cases the diphosphate group is omitted since it is thought to act primarily as an anchor for the cofactor and, consequently, was not deemed relevant to the current study. Model **A** is the simplest, representing the cofactor alone in solution. It comprises 31 atoms and has a net charge of +1. Models **B**–**E** represent the cofactor in the BFDC active site in the absence and presence of bound ligands. The active site model is built on the basis of the crystal structure (PDB ID 1MCZ) and is identical to that used in the mechanistic study [29]. As shown in Figure 1, the model comprises all groups that make up the active site pocket, including residues that surround the ThDP cofactor and the ligand. A detailed description of the residues included in the model and the choice of protonation states is provided in reference [29].

In model **B** the active site does not contain any ligand, and is considered for comparative purposes. It has a total of 291 atoms and a net charge of 0. In model **C** the active site contains a crystallographic water molecule and includes 294 atoms with net charge of 0. In model **D** the water is replaced by the benzoylformate substrate in its deprotonated form and has thus 307 atoms and a net charge of −1. Finally, in model **E** the active site contains (*R*)-mandelate, again in its deprotonated form, and consists of 309 atoms with a net charge of −1. In the active site models **B**–**E** a number of atoms are kept fixed in the geometry optimizations in order to preserve the overall structure of the active site and avoid excessive movements of the various groups. The fixed atoms are indicated by asterisks in Figure 1B.

The states of the ThDP cofactor considered here are shown in Scheme 1. The starting point for each model is the **AP** state, the energy of which is set to zero, and the energies of the other states are then compared to it.

### Model **A**: cofactor in solution

**Figure 2:**
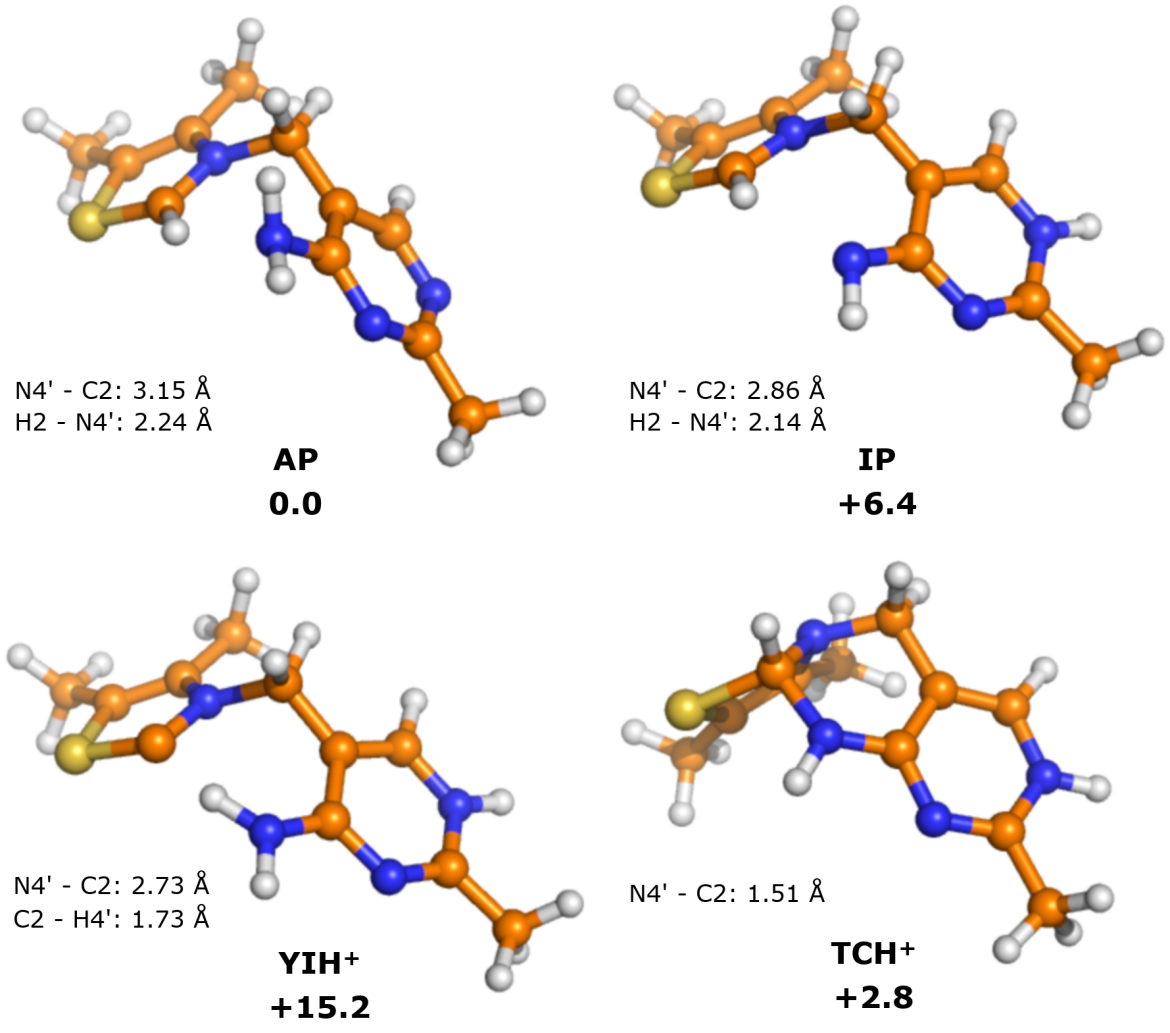
Optimized structures of the states of ThDP in the absence of enzyme (model **A**). Relative energies are indicated in kcal/mol.

In order to analyze the effect of the enzyme environment on the properties of the various ThDP states, it is important to first consider the solution states of the cofactor in the absence of enzyme. The calculations show that the difference in energy between the lowest energy conformer and the typical V-conformation of enzyme-bound ThDP [48] is 4.2 kcal/mol. Interestingly, the lowest energy structure also adopts a V-shape, but one in which thiazolium ring is perpendicular to the pyrimidine ring (see Supporting Information File 1 for an optimized structure). Given that this study compares enzyme-bound states of ThDP, it is appropriate to use the typical V-conformation of the AP form as the starting/reference point. With that in mind, the optimized geometries of the various V-states of the cofactor alone are displayed in Figure 2.

Calculations on model **A** show that the **AP** state is the most stable, but the tricyclic form **TCH^+^** is only 2.8 kcal/mol higher in energy (Table 1). Presumably the proximity of N4' to C2 in the V-conformation makes this state more accessible than it would be if ThDP was unconstrained in solution. Both the **IP** and **YIH^+^** states are considerably higher in energy, at +6.4 and +15.2 kcal/mol, respectively. It should be noted that the acid/base conjugates of these states (**APH^+^**, **TC** and **YI**, respectively) were not calculated, as these structures would have different numbers of atoms and so the energies would not be directly comparable.

**Table 1:** Calculated relative energies (kcal/mol) of the various ThDP states. The most stable state for each model is indicated in bold face.

state	model **A**	model **B**	model **C**	model **D**	model **E**

**AP**	**0.0**	**0.0**	**0.0**	0.0	0.0
**APH^+^**	–	+1.2^a^	+1.9^a^	+3.0	**−0.3**
**IP**	+6.4	+12.8	+16.0	−0.9	+10.2
**YI**	–	+11.6	+11.0	+3.0	+23.2
**YIH^+^**	+15.2	+11.3^a^	+11.1^a^	+6.0	+20.2
**TC**	–	+2.6	+8.9	−4.9	+7.9
**TCH^+^**	+2.8	+2.0^a^	+8.5^a^	**−6.3**	+11.3

^a^Values are calculated with the N1′–H distance constrained to 1.15 Å (see text).

### Model **B**: ThDP in the empty active site

**Figure 3:**
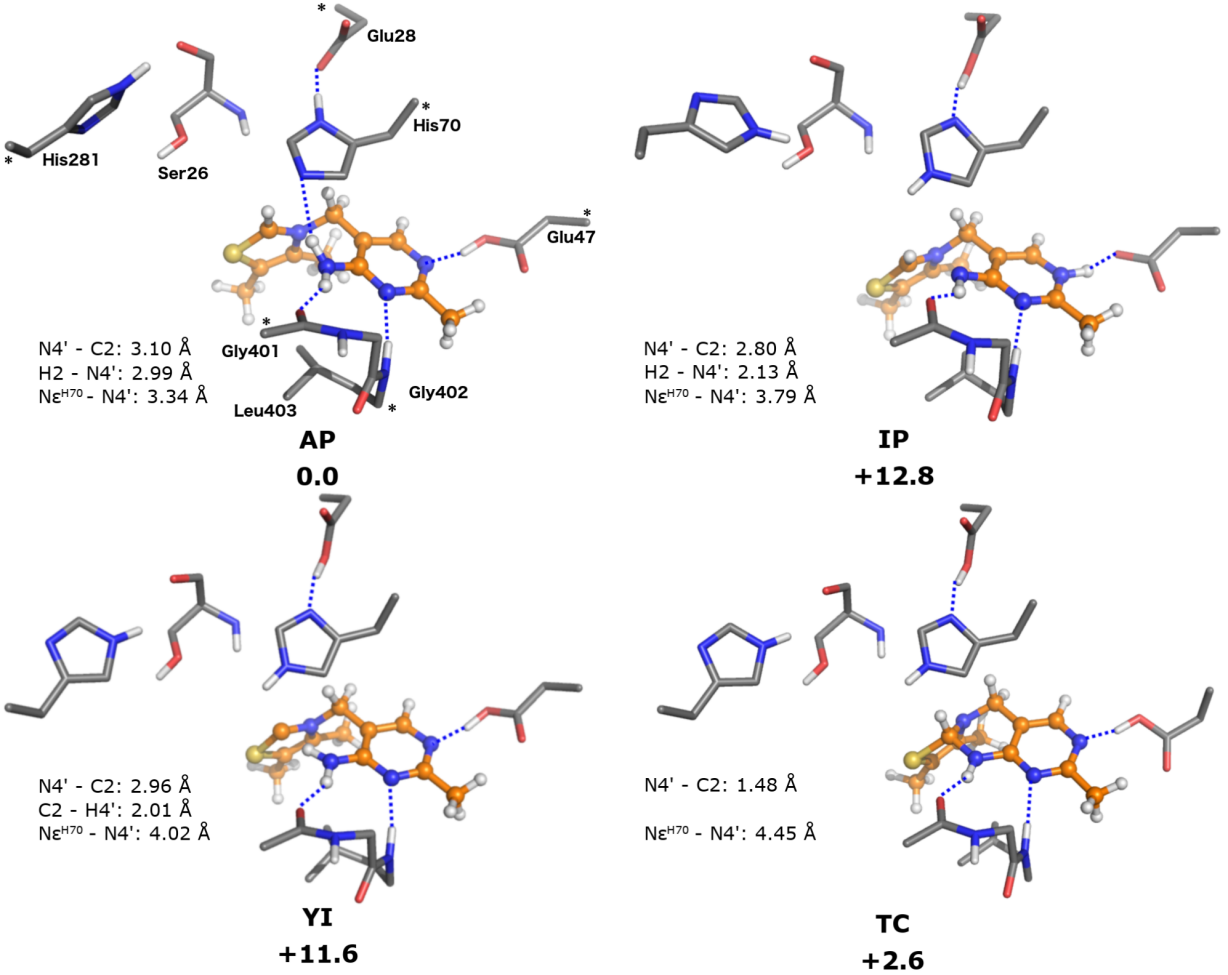
Optimized structures of the states of BFDC-bound ThDP in the absence of ligand (model **B**). Relative energies are indicated in kcal/mol. For clarity, only a selected part of the model is shown, for full model, see Figure 1.

Model **B** represents ThDP in the active site of BFDC in the absence of ligand. The geometries of the different ThDP states were optimized (Figure 3) and their energies evaluated (Table 1). Here, the enzyme provides both electrostatic and steric interactions with ThDP, all of which are expected to affect the cofactor’s geometry and energy. Of particular interest is the conserved Glu47 residue which forms a hydrogen bond to N1' of the pyrimidine ring. It is important to note that, during the geometry optimizations of the three states **YIH^+^**, **APH^+^** and **TCH^+^**, the N1' proton invariably transferred spontaneously to the carboxylate of Glu47 thereby yielding the conjugated states **YI**, **AP** and **TC**. In order to assess independently the effect of the N1′ protonation state, approximate energies of **YIH^+^**, **APH^+^** and **TCH^+^** were calculated by restraining the N1′–H distance to 1.15 Å. Even with that constraint the energies obtained are within 2 kcal/mol of those of their conjugates (Table 1), showing that N1′ protonation/deprotonation has only marginal impact on the relative energies of the cofactor states.

In the **AP** state, His70 interacts with the cofactor through a hydrogen bond between the Nε and the exocyclic N4' amino group, a bond that is not present in the other states. With that notable exception, the overall geometries of the different states are quite similar, and bond distances are also fairly consistent (Figure 3).

As with model **A**, the **AP** state is found to be the lowest energy state in model **B**. The stability of **TC** state in model **B** is similar to that of the **TCH^+^** in the model of the cofactor alone (+2.6 compared to +2.8 kcal/mol, respectively).The energy of the **YI** state is also reasonably close to that of **YIH^+^** in the cofactor alone (+11.6 vs +15.2 kcal/mol, relative to their respective **AP** states). Indeed, it was not until the energy of the **IP** state was calculated that the enzyme showed any significant effect. In this instance the **IP** state was calculated to be 12.8 kcal/mol higher than **AP**, i.e., more than 6 kcal/mol higher than the value calculated in the absence of enzyme.

### Model **C**: active site including the crystallographic water

**Figure 4:**
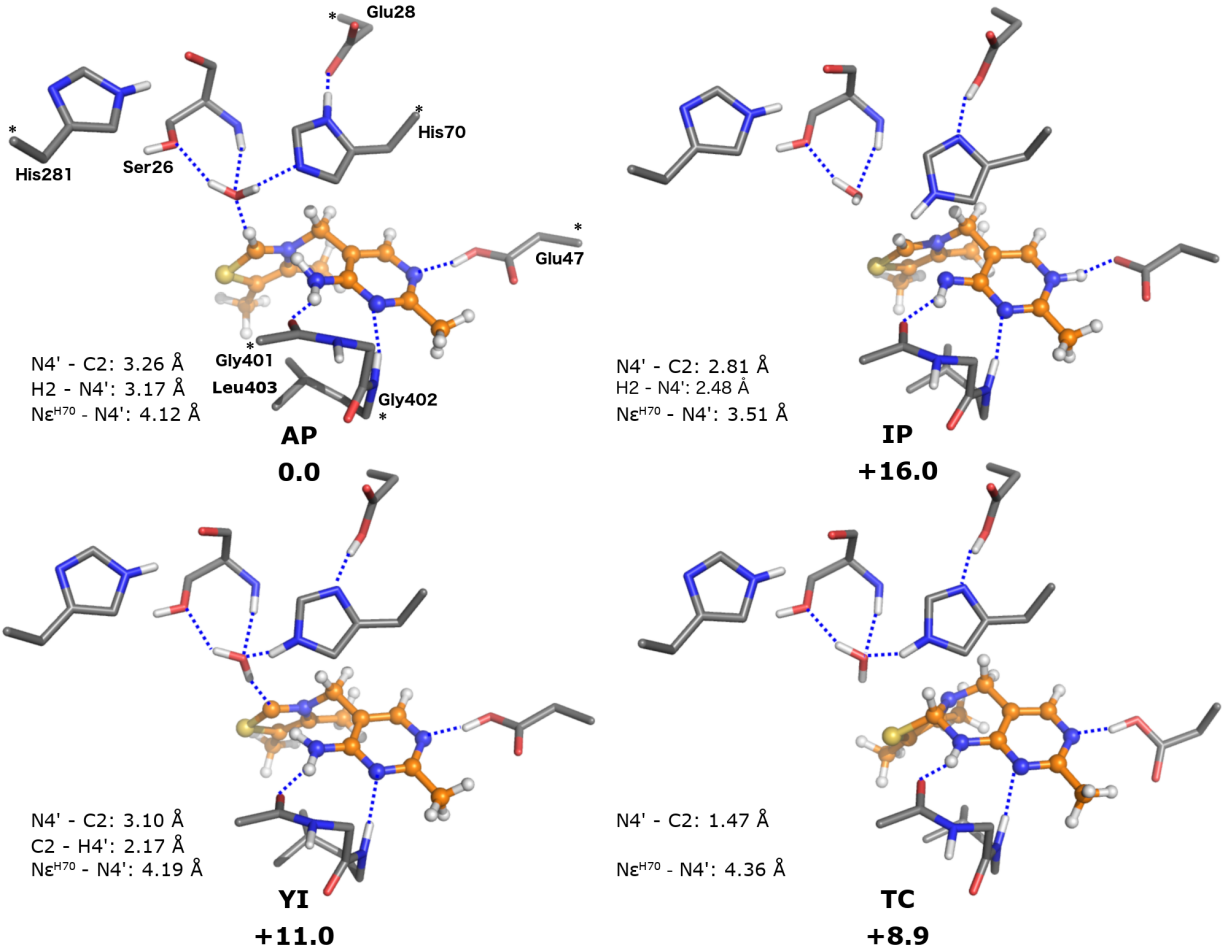
Optimized structures of the ThDP states for the model including the crystallographic water (model **C**). Relative energies are indicated in kcal/mol.

In the X-ray structure of unliganded BFDC, there is a crystallographic water molecule that is displaced when a ligand is present [13,49]. In model **C**, that water molecule is included and is found to bind in the same position regardless of the state of the cofactor. A superposition with the crystal structure without substrate (PDB 1BFD) shows that the model calculations reproduce very well the position of this water (see Supporting Information File 1), even though the hydrogen-bonding pattern of the water molecule changes somewhat between the states (Figure 4). Interestingly, there is a hydrogen bond from the water to the anionic C2 carbon in the **YI** state (OH^…^C distance of 1.91 Å), with the negative charge on C2 further stabilized by interaction with the exocyclic NH_2_ group (NH^…^C distance of 2.17 Å). The superposition of the structures of model **B** and model **C** reveals that inclusion of the water molecule causes also a slight movement of the thiazolium ring of the cofactor towards the interior of the active site cavity (see Supporting Information File 1).

Energetically, we note that the **IP** state is destabilized compared to that in model **B**, now being 16.0 kcal/mol higher than **AP**, an increase of 3.2 kcal/mol. Conversely, the stability of the **YI** state was very similar (+11.0 vs +11.6 kcal/mol) to that observed for model **B** suggesting the water molecule has little effect on the stability of the ylide. However, the water molecule reserves its largest effect for the **TC** state which now is 8.9 kcal/mol less stable than **AP**, an increase of 6.3 kcal/mol over that observed in model **B**. Overall, it would appear that the effect of the water molecule is to stabilize the **AP** state compared to the other states. The exception is the **YI** state, which seemingly benefits from the new hydrogen bond from the water molecule to the C2 carbanion.

Finally, as with model **B**, the geometries for the **YIH^+^**, **APH^+^** and **TCH^+^** states could not be obtained, as geometry optimizations lead to their respective conjugates. However, constrained optimizations again show that the energies are not affected significantly by the protonation (see Table 1).

### Model **D**: active site including benzoylformate

**Figure 5:**
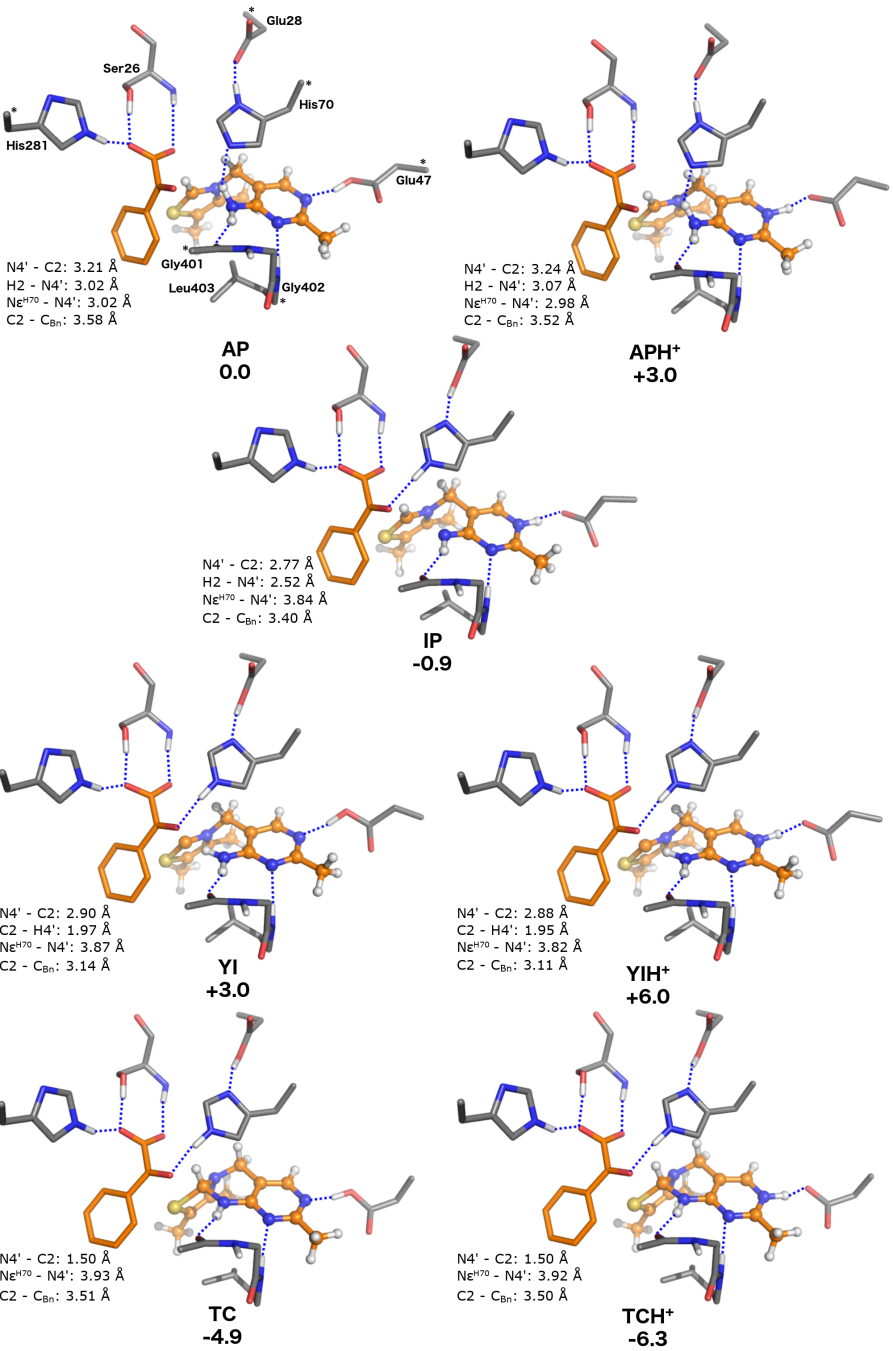
Optimized structures of the ThDP states in the BFDC active site containing the substrate, benzoylformate (model **D**). Relative energies are indicated in kcal/mol.

In model **D**, which includes the native substrate, benzoylformate (BF), all the states shown in Scheme 1 could be located by the geometry optimizations. As with the crystallographic water in model **C**, the presence of the substrate pushes the thiazolium ring somewhat towards the interior of the cavity. In all ThDP states the carboxylate of BF forms hydrogen bonds to the side chain hydroxy group and the backbone NH of Ser26, and to the Nε of His281 (Figure 5). In the **AP** and **APH^+^** states, the Nδ of His70 accepts a hydrogen bond from the exocyclic NH_2_ group, with an N^…^HN distance of 2.1 Å. In the other states, the Nε of His70 is protonated and donates a hydrogen bond to the carbonyl of the substrate. In **YI** and **YIH^+^**, the exocyclic NH_2_ interacts with the C2 carbanion of the thiazolium ring.

Strikingly, the presence of the substrate has a dramatic impact on the relative stabilities of the various states as compared to the water (model **C**) or the empty cavity (model **B**). Presumably this effect is primarily due to the overall negative charge of the benzoylformate and the bulk of the phenyl substituent. The most significant changes are seen in the energies of the two catalytically productive states, **IP** and **YI**. Now, the former is more stable, by 0.9 kcal/mol, than the **AP** state. This may not seem much but the overall change is substantial as the **IP** state was calculated to be 6.4, 12.8 and 16.0 kcal/mol higher in energy than the **AP** state in models **A**, **B**, and **C**, respectively. The energy of the ylide is also lowered in the presence of the substrate and the **YI** state is now only +3.0 kcal/mol compared to **AP**. In the other models the difference was more than 11 kcal/mol (Table 1).

Although these results clearly suggest that substrate binding results in catalytically productive states of the cofactor, this is not the whole story. Model **D** also indicates that substrate binding produces a major stabilization of the two non-productive tricyclic species. In fact, the most stable state is found to be **TCH^+^**, which is calculated to be 6.3 kcal/mol more stable than the **AP** state. Also the deprotonated **TC** state is 4.9 kcal/mol more stable than **AP**, and both tricyclic states are at least 4 kcal/mol lower in energy than the **IP** form. While substrate binding favoring the non-productive species is surprising and seems counterintuitive, benzoylformate binding also makes the catalytically essential **IP** and **YI** forms more accessible than in any of the other models. Importantly, as detailed in our recent paper on the reaction mechanism of BFDC, this model is consistent with the kinetics of the BFDC reaction [29].

In a final note on model **D**, although the calculations show that proton transfer from N1' to Glu47 is not spontaneous in this model, the energy difference between the conjugated pairs **AP/APH^+^**, **TC/TCH^+^**, **YI/YIH^+^** remains very low, suggesting the forms are readily interchangeable (Table 1).

### Model **E**: active site of BFDC with (*R*)-mandelate bound

**Figure 6:**
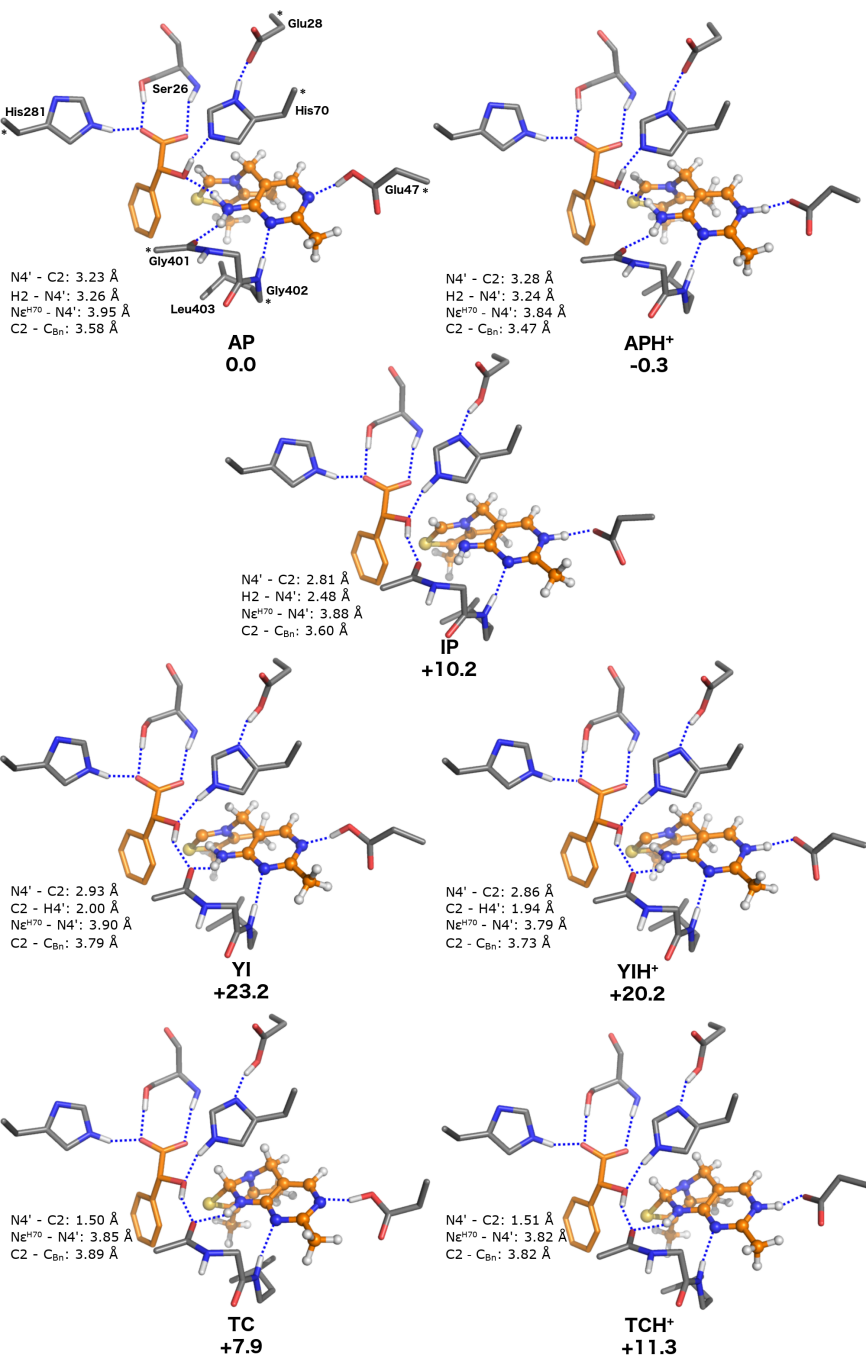
Optimized structures of the ThDP states for the model including (*R*)-mandelate (model **E**). Relative energies are indicated in kcal/mol.

In model **E**, in which the active site of BFDC contains the inhibitor (*R*)-mandelate, the hydrogen-bonding network is very similar to that of benzoylformate in model **D**. However, as shown in Figure 6, the benzylic hydroxy group provides a source of additional interactions. In the **AP** and **APH^+^** states, the hydroxy group forms hydrogen bonds with His70 and the exocyclic NH_2_. Support was lent to the validity of model **E** when superposition of the structure of the **APH^+^** form on the structure of BFDC:(*R*)-mandelate complex (PDB 1MCZ) showed no major movements (see Supporting Information File 1). In the other states, the bond to His 70 is maintained but that to the exocyclic NH2 is broken. Instead the hydroxy group forms a hydrogen bond to the backbone carbonyl of Gly401.

Energetically, we note that, in this model, **AP**/**APH^+^** are by far the most stable states with the **IP**, **YI** and **TC** states being 10.2, 23.2 and 7.9 kcal/mol higher than **AP**, respectively (Table 1). Further, each of these states is more than 10 kcal/mol higher in energy relative to the **AP** state than its counterpart in model **D** in which the substrate is bound (Table 1). Clearly the binding of the (*R*)-mandelate causes a stabilization of the **AP/APH^+^** states relative to the others.

As seen from Table 1, despite having the same overall charge and similar bulkiness of the substituents, the binding of the benzoylformate (model **D**) and (*R*)-mandelate (model **E**) result in quite different energies. The superposition of the **AP** states of the two models (see Supporting Information File 1) shows that the additional hydrogen bond provided by the benzylic hydroxy group of (*R*)-mandelate (vide supra) contributes significantly to this difference. Further, changing from the sp^2^ carbonyl carbon to the sp^3^ benzylic carbon results in a substantial movement of the substituent oxygen which also contributes to the energy difference between the models.

According to Table 1 the **AP**/**APH^+^** forms are the most stable states for models **C** and **E**, i.e., BFDC in the absence of ligand and in the presence of (*R*)-mandelate. The CD spectrum of BFDC shows a small minimum at around 325 nm, attributed to the **AP** form. Titrating BFDC with methyl benzoylphosphonate (MBP), a mechanism-based inhibitor, gave rise to a new maximum at around 300 nm, attributed to the **IP** form, with a concurrent loss of signal at 325 nm [50]. Based on the data in Table 1, it is not certain that the titration with benzoylformate would give rise to the **IP** form. However, the data unambiguously suggest that titration of BFDC with (*R*)-mandelate should result in no change in its CD spectrum, even when the enzyme is saturated. Accordingly, the titration was carried out and, indeed, even at (*R*)-mandelate concentrations well in excess of its *K*_i_ value of 1 mM [49], no change in the spectrum was observed (see Supporting Information File 1 for details).

## Discussion

Since it was first purified from beer yeast over 80 years ago [51], the structure of ThDP and its related intermediates and ionization states have undergone intensive investigation (summarized in references [2,52]). Most of these investigations have focused on the structure and properties of the covalently modified ThDP intermediates of diverse ThDP-dependent enzymes. Less attention has been paid to the variety of states the cofactor itself can adopt on the enzyme. As shown in Scheme 1, when various tautomers and ionization states are included, ThDP can adopt at least seven forms on any given ThDP-dependent enzyme. This is prior to any reaction taking place. Most of these are accounted for in typical analyses [52] but the two tricyclic forms, **TC** and **TCH^+^**, are rarely considered. Unlike the tricyclic states, which could be regarded to be non-productive, the **IP** and the **YI** forms arising from it are essential for catalysis and always considered in any mechanistic study. However, conceivably all seven states could be energetically accessible and could influence the catalytic mechanism. Over the past several years there has been an increasing use of ThDP-dependent enzymes as chiral catalysts [53]. Given that all of these enzymes will require ready access to the **IP** and **YI** forms, it seemed logical to take a closer look at the relative energies of the various states and how those energies may be affected by the binding of different ligands. Toward that end, we have used DFT calculations to explore the energetics of the various states of enzyme-bound ThDP using benzoylformate decarboxylase as the model enzyme.

The work was predicated on two elements. First, that the cofactor was held in a V-conformation on the enzyme, and second, that the resting (reference) state of the cofactor was the **AP** form. Both have been confirmed experimentally [13,49–50] and are typical of most, if not all, of the ThDP-dependent enzymes studied to date. In total, five models were employed: models **A** and **B** providing a comparison of the cofactor states in the presence and absence of enzyme, and models **C**–**E** examining the effects of active site ligands.

Models **A** and **B** both predict the **AP** state to be the most stable, vindicating its use as the reference state. Perhaps the first surprise was the difference in magnitude and overall effect the active site ligands had on the relative energy levels. For example, the simple addition of a crystallographic water destabilized both the **IP** and **TC** forms by 3 and 6 kcal/mol, respectively, thereby ensuring that BFDC largely exists as the **AP** form. Even more surprising was the comparison of the substrate, benzoylformate, and the inhibitor, (*R*)-mandelate. With the exception of an sp^3^ rather than sp^2^ hybridized benzylic carbon, (*R*)-mandelate is identical to benzoylformate. However, they have markedly different effects on the states of the cofactor. In model **C**, corresponding to the native enzyme, the **IP** form is 16 kcal/mol less stable than the **AP** form. When benzoylformate binds (model **D**), the **IP** form becomes energetically favored by 0.9 kcal/mol, an overall change of 17 kcal/mol. This is accompanied by an 8 kcal/mol stabilization of the catalytically essential **YI** form. Conversely, when the inhibitor is bound (model **E**), the **IP** and **YI** forms are ca. 10 and 20 kcal/mol less stable than in model **D**. Clearly the substrate-induced changes combine to facilitate catalysis, while those brought about by the inhibitor make reaction more difficult.

Considering that they have been largely ignored in previous studies, the next surprise was that the tricyclic forms **TC** and **TCH^+^** were relatively stable, in both the absence and presence of enzyme. In fact, it seemed that the primary effect of the binding of ThDP to the enzyme was to bring about the destabilization of the **IP** form. Over all five models, the tricyclic forms were consistently more energetically stable than the catalytically essential **IP** and **YI** forms (Table 1). While it may be argued that stabilization of the tricyclic forms could prove to be enzyme specific, the relatively low energy of the tricyclic state in the absence of enzyme cannot be disregarded, and certainly suggests that the **TC**/**TCH^+^** forms may be more common than previously recognized. Further, and consistent with results for the **YI/YIH^+^** forms, the relative stabilities of **TC**/**TCH^+^** states proved to be ligand specific. In model **C**, the **AP** form is ca. 9 kcal/mol more stable than the **TC**/**TCH^+^** forms. However, after substrate binding (model **D**) the **TC**/**TCH^+^** states are ca. 5 kcal/mol more stable than **AP**. Thus, the presence of benzoylformate shifts the relative energies by 14 kcal/mol and, concomitantly, makes the tricyclic forms the most stable species. The inhibitor again provides the contrast, for the binding of (*R*)-mandelate (model **E**) has virtually no effect on the relative energy levels of the tricyclic forms, and **AP** remains clearly the most stable state.

At this point, it is reasonable to assess the validity of the current computational results in light of available experimental information. In the first instance the results confirm that the **AP** state is the lowest energy, i.e., resting state. This was one of the elements on which the work was predicated and is consistent with data obtained from, among others, BFDC, benzaldehyde lyase, pyruvate oxidase, pyruvate decarboxylase and the E1 subunit of the pyruvate dehydrogenase complex (summarized in [54]). In fact, there are only two cases in which the **IP** form has been observed in the resting enzyme, namely pyruvate oxidase and the pyruvate dehydrogenase complex. In both cases, the **AP** state was the predominant form [54].

Secondly, the results show that substrate binding dramatically lowers the energy of the **IP** and **YI** states, which would, presumably, increase the rate and extent of ylide formation. This observation is more difficult to demonstrate experimentally. While H/D exchange experiments have been used as a measure of the rate of ylide formation, substrate activation has only been observed with allosteric enzymes such as yeast PDC [8]. Further, even though there is a CD signature for the **IP** state, it is usually associated with formation of a tetrahedral reaction intermediate. As a result it is difficult to separate any increase in the **IP** signal arising from substrate binding from that due to intermediate formation. Possibly the closest to experimental support came from an experiment in which the reaction of BFDC with MBP was monitored by stopped-flow measurements at 308 nm using the intrinsic absorbance of the **IP** state. In that case the results implied that there was a transient formation of a Michaelis complex which was accompanied by an increase in the **IP** form [55].

Next, the calculations suggest that the binding of (*R*)-mandelate should not change the state of the cofactor. Again, somewhat difficult to prove conclusively but titration of BFDC with the substrate analogue, MBP, provided clear evidence for the conversion of the **AP** to the **IP** state for the former. Conversely, and consistent with predictions, the **AP** state remained unchanged when a similar titration was carried out with the inhibitor, (*R*)-mandelate (see Supporting Information File 1).

Finally, what evidence is there for the formation of tricyclic states? As noted in the introduction, there has been little or no effort to identify tricyclic intermediates on ThDP-dependent enzymes. Critically, even though they were treated dismissively, there are two X-ray structures which, at a minimum, provide unambiguous evidence for the formation of stable tricyclic intermediates on an enzyme [46–47]. Additional evidence, albeit more indirect, comes from an inhibition study using omeprazole, which was predicted to possibly interact with ThDP-dependent enzymes. The prediction was based on the similarity of omeprazole to the tricyclic form of thiamin. This was confirmed experimentally when omeprazole was subsequently shown to be a competitive inhibitor of both transketolase and PDC, with a *K*_i_ value for the latter only ca. 20 times the *K*_m_ for ThDP measured in the same experiment [56].

The current calculations show that, in the presence of substrate, **TC**/**TCH^+^** are the most stable states of ThDP on BFDC. Yet, even though a large number of high-resolution structures of BFDC variants, in the presence and absence of ligands, have been determined, none of them shows the tricyclic intermediate. This may seem surprising but it must be considered that when benzoylformate is present the catalytic cycle is in operation, reactions are running and covalent ThDP intermediates are being formed. As detailed in our recent paper, the enamine is the most stable reaction intermediate [29], which makes it unlikely that the **TC** state will be detected experimentally. Furthermore, in the absence of the substrate (model **C**) or in the presence of the inhibitor (model **E**), the **TC** states are clearly disfavored, with calculated energies of +8.9 and +7.9 kcal/mol, respectively, relative to the **AP** state.

Are the tricyclic forms even relevant? That is really the crux of the matter, and the answer is, for BFDC at least, yes! It is important to note that the tricyclic forms **TC**/**TCH^+^** are calculated to be more stable than the ylide forms **YI**/**YIH^+^** in all considered models. Further, the calculations on model **D** indicate that an energy penalty of ca. 5 kcal/mol must be paid to go from the **TCH^+^** to the **YI** state, which is the catalytically active form of the cofactor. This, in turn, effectively increases the barrier for formation of the first reaction intermediate. In fact, as shown in the paper on the catalytic mechanism that inspired this work, the energy barrier brought about by the stable tricyclic state fits well with the experimental evidence for the slow first step, i.e., formation of the mandelylThDP adduct [29].

In addition to BFDC, the X-ray structures of tricyclic intermediates suggest that an even greater stabilization is present on phosphoketolase and acetolactate synthase. It could well be argued that **TC** stabilization may prove to be rare and specific to only a few enzymes. Yet, the relatively low energy of the tricyclic state in the absence of enzyme cannot be disregarded, and certainly suggests that the **TC** forms may be more common than previously thought. Over the past few years, a rapid-quench NMR technique has been employed to determine microscopic rate constants for elementary steps in several ThDP-dependent enzymes. It is notable that, in addition to BFDC [55], *E. coli* AHAS I and II [57], glyoxylate carboligase [7], DXP synthase [58] and indolepyruvate decarboxylase [59] all have formation of the first tetrahedral intermediate as the rate-determining step. Of course, in the absence of the corresponding calculations it is impossible to definitively state that this is due to stabilization of the **TC** state, but the question is worth asking. The pyruvate oxidase from *Lactobacillus plantarum* provides some support in that both the **AP** and **IP** forms are present in the resting enzyme [54] and decarboxylation, rather than formation of the first intermediate, was found to be rate limiting [60]. On the other hand, product release was the slowest step for *Zm*PDC and *Sc*PDC [61], so clearly not all ThDP-dependent enzymes behave in the same manner.

Of course, while the relative stability of the **TC** form may slow down the BFDC reaction, it is conceivable that it may also play a beneficial role. As the p*K*_a_ of the C2 proton decreases, the activity of the ThDP cofactor increases [62]. However, concomitantly, the thiazolium ring becomes more susceptible to hydrolysis to a catalytically inactive form [44]. The stable tricyclic form of the cofactor, which can readily revert to its active form, may provide a protective mechanism against hydrolysis [44].

Two final thoughts: first, the current results show that even when substrate is bound, the tricyclic state, not the ylide, is the most energetically stable. This observation implies that starting the computational investigations of the ThDP-dependent catalytic mechanism directly from the ylide, as done in numerous examples in the literature, may give rise to an incomplete, if not inaccurate, picture of the energy profile of the reaction. Second, many ThDP-dependent enzymes are being evaluated for use as biocatalysts. The stark difference in the effect of two very similar ligands, benzoylformate and (*R*)-mandelate, on the activation of the cofactor suggests that the use of alternative substrates or, possibly more importantly, the evolution of ThDP-dependent enzymes to accept a wide range of non-native substrates, might not be as simple as may have been expected.

## Experimental

All calculations were performed with the B3LYP-D3(BJ) [62–65] density functional method and using the Gaussian 09 package [66]. The geometries were optimized with the 6-31G(d,p) basis set, and the energy of the stationary points was refined by single-point calculations with 6-311+G(2d,2p) basis set. Frequency calculations were done at the same level of theory as the optimizations to obtain zero-point energy corrections, and solvation energies were calculated using the implicit solvent method SMD [67] with a dielectric constant ε = 4.

## Supporting Information

File 1 Lowest-energy conformation of model **A**, superpositions of the **AP** state/model **C** and **APH^+^** state/model **E** with crystal structures, superposition of the **AP** state/models **B** and **C**, superposition of the **AP** states/models **D** and **E**, experimental CD spectra, calculated energies and energy corrections, and Cartesian coordinates of all optimized structures.
