# Supplementary material for: Computational characterization of enzyme-bound thiamin diphosphate reveals a surprisingly stable tricyclic state: implications for catalysis
Source: Beilstein J Org Chem. 2019 Jan 16;15:145–59. doi: 10.3762/bjoc.15.15 (PMC6350894; doi:10.3762/bjoc.15.15)
Supplement: File 1 — Lowest-energy conformation of model A, superpositions of the AP state/model C and APH+ state/model E with crystal structures, superposition of the AP state/models B and C, superposition of the AP states/models D and E, experimental CD spectra, calculated energies and energy corrections, and Cartesian coordinates of all optimized structures. [file Beilstein_J_Org_Chem-15-145-s001.pdf]

BEILSTEIN JOURNAL OF ORGANIC CHEMISTRY

Supporting Information

for

Computational characterization of enzyme-bound thiamin diphosphate reveals a surprisingly stable tricyclic state: implications for catalysis

Ferran Planas, Michael J. McLeish and Fahmi Himo

Beilstein J. Org. Chem. **2019,** *15,* 145–159. doi:10.3762/bjoc.15.15

Lowest-energy conformation of model A, superpositions of the AP state/model C and APH$^+$ state/model E with crystal structures, superposition of the AP state/models B and C, superposition of the AP states/models D and E, experimental CD spectra, calculated energies and energy corrections, and Cartesian coordinates of all optimized structures

License and Terms: This is a supporting information file under the terms of the Creative Commons Attribution License (http://creativecommons.org/licenses/by/4.0). Please note that the reuse, redistribution and reproduction in particular requires that the authors and source are credited.
The license is subject to the *Beilstein Journal of Organic Chemistry* terms and conditions: (https://www.beilstein-journals.org/bjoc)

Table of Contents

1. Lowest-energy structure of the cofactor alone S2
2. Superposition of **AP** state in **Model C** with crystal structure S3
3. Superposition of **APH**$^+$ state in **Model E** with crystal structure S4
4. Superposition of the **AP** states for **Models B** and **C** S5
5. Superposition of the **AP** states for **Models D** and **E** S6
6. Experimental CD spectra S7
7. Energy decomposition of the calculated stationary points S8
8. Cartesian coordinates of optimized structures S10

1. Lowest-energy structure of the cofactor alone

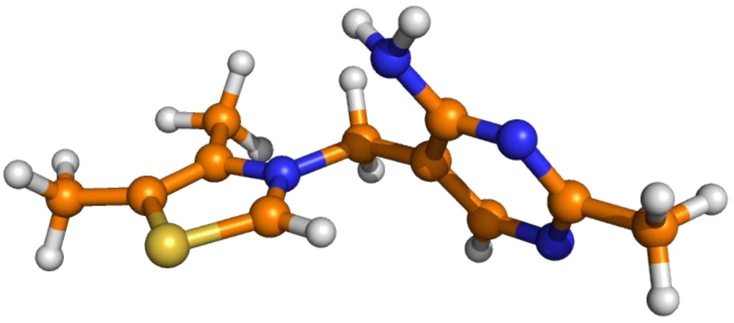

Figure S1: *Lowest-energy conformation of the cofactor alone in solution (**Model A**). This structure is calculated to be 4.2 kcal/mol lower in energy than the typical V-shape of the AP state shown in Figure 2 in the main text.*

2. Superposition of AP state in Model C with crystal structure

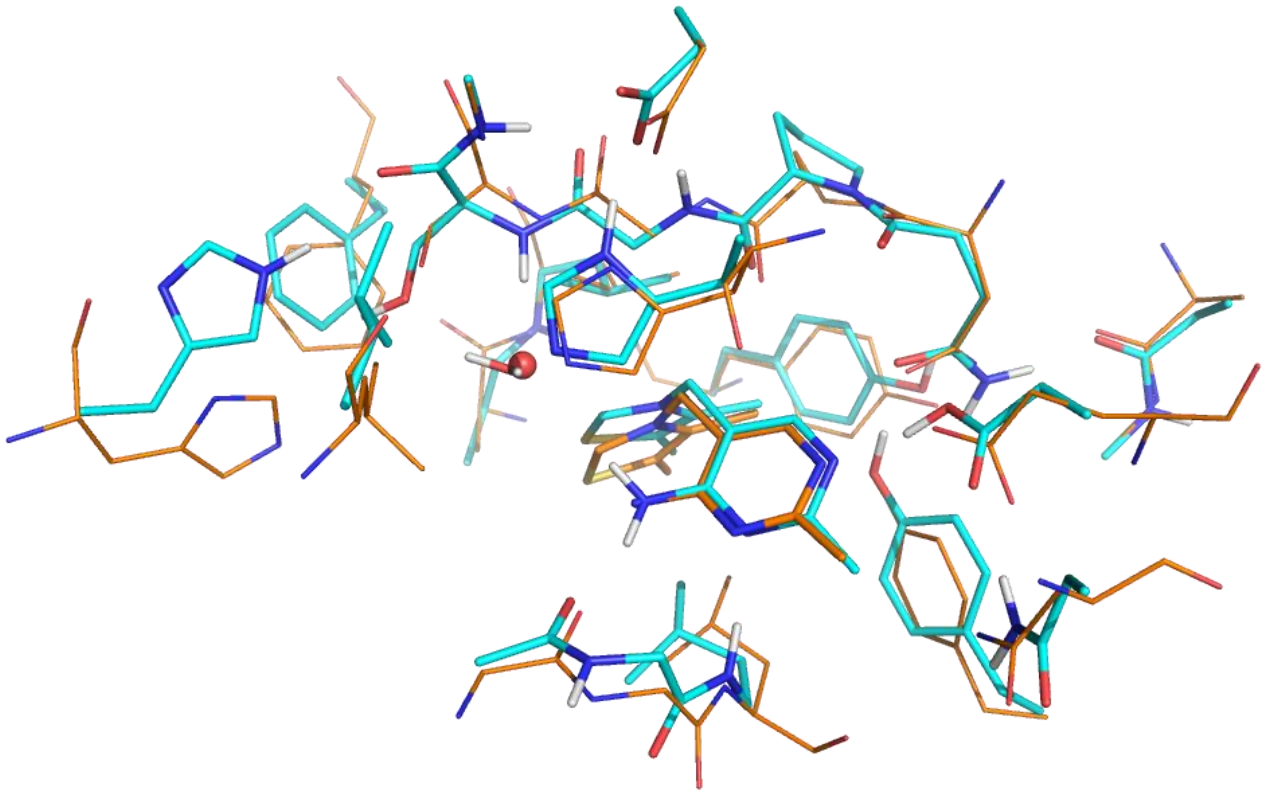

Figure S2: *Residues colored in orange and the water represented as a sphere correspond to the crystal structure containing the crystallographic water (PDB 1BFD). Residues in turquoise and the water represented with lines correspond to the* ***AP*** *state of* ***Model C****.*

3. Superposition of APH$^+$ state in Model E with crystal structure

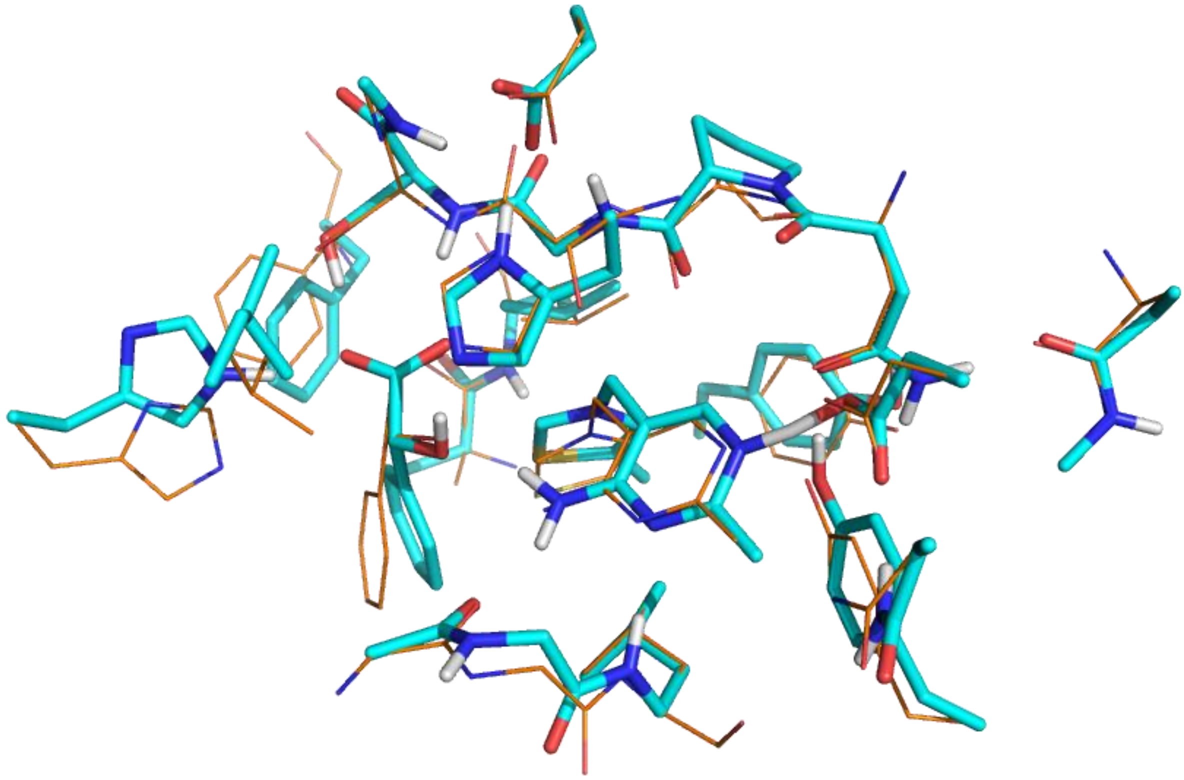

Figure S3: *Residues colored in orange correspond to the crystal structure containing R-Mandelate (PDB 1MCZ), while residues colored in turquoise correspond to the **APH**$^+$ state of **Model E**.*

4. Superposition of the AP states for Models B and C

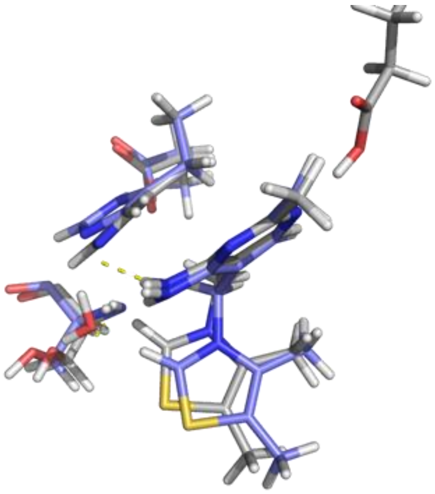

Figure S4: *Superposition of the* ***AP*** *states of* ***Model B*** *representing the empty cavity (grey color) with* ***Model C*** *that includes the crystallographic water (blue color).*

5. Superposition of the AP states for Models D and E

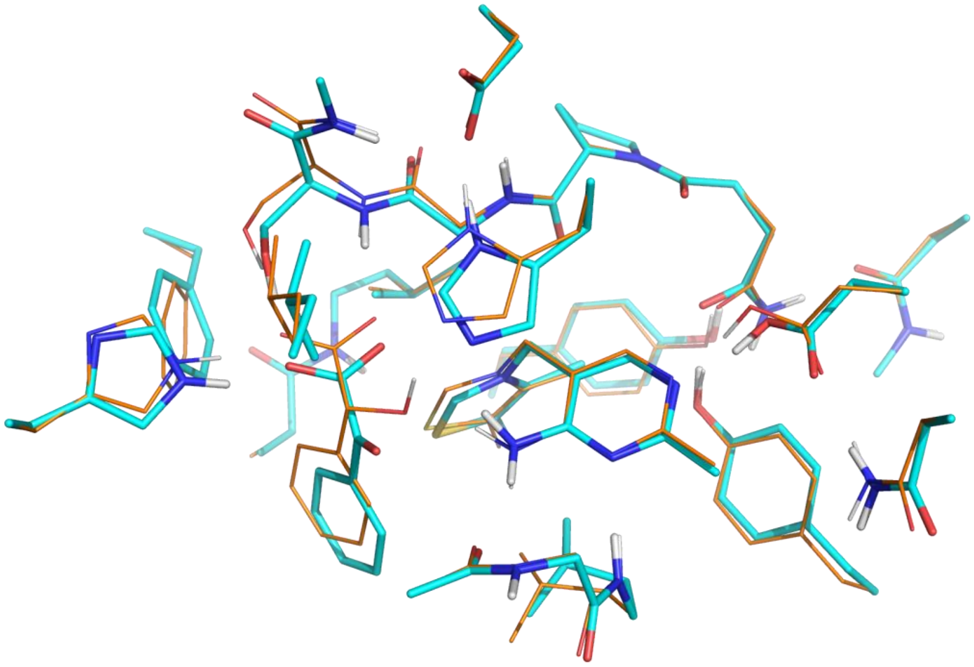

Figure S5: *Superposition of the* ***AP*** *states of* ***Model D*** *representing the active site with benzoylformate substrate (blue color) with* ***Model E*** *that includes the (R)-mandelate inhibitor (orange color).*

6. Experimental CD spectra

Aliquots of (*R*)-mandelate or methyl benzoyl phosphonate (MBP) were added to BFDC (2.0 mg/mL, 35 µM concentration of active centers) in 20 mM KH_2PO_4 (pH 6.5), containing $MgCl_2$ (2.5 mM) and ThDP (0.50 mM). The CD spectra were recorded in the near-UV region using a Jasco J-810 circular dichroism spectropolarimeter. Difference spectra were obtained by subtraction of the spectrum of BFDC in the absence of ligand using SigmaPlot 12.

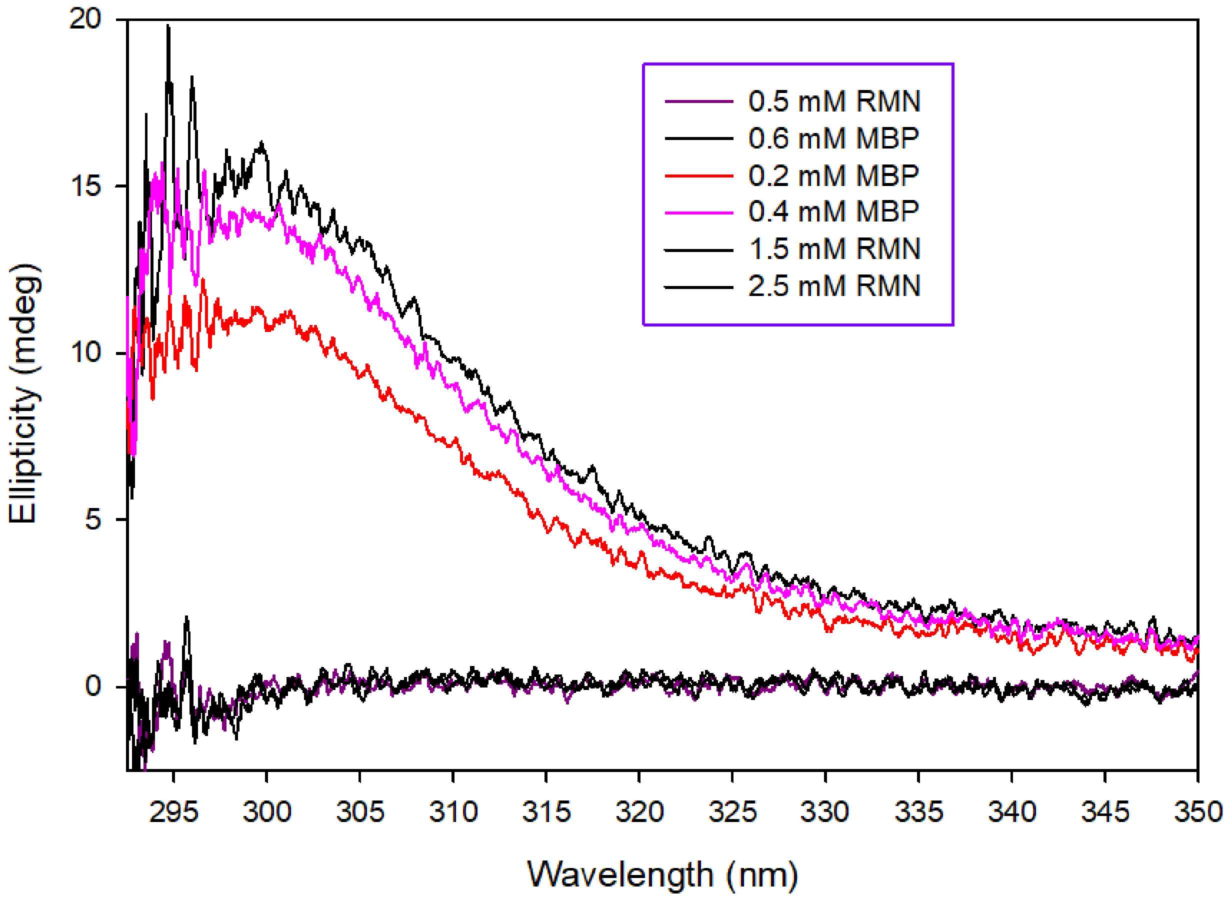

Figure S6: *Titration of BFDC (2 mg/mL) with methyl benzoyl phosphonate and R-mandelate at pH 6.5. Difference spectra were obtained by subtracting the spectra of BFDC in the absence of ligand.*

7. Energy decomposition of the calculated stationary points

	State	E_{el} (a.u.)	E_{bb} (a.u.)	E_{solv} (a.u.)	ZPE (a.u.)	E_{tot} (a.u.)	E_{rel} (kcal/mol)
**Cofactor alone (Model A)**	**AP**	-1045.28221	-1045.47855	-1045.35311	0.20993	-1045.33951	0.0
	**IP**	-1045.27665	-1045.47334	-1045.34318	0.21059	-1045.32928	7.4
	**YIH^+**	-1045.26352	-1045.45822	-1045.33111	0.21056	-1045.31526	15.6
	**TCH^+**	-1045.28181	-1045.47440	-1045.35747	0.21505	-1045.33502	2.9
**Empty Cavity (Model B)**	**AP**	-6380.26084	-6381.95241	-6380.34894	2.51861	-6379.54903	0.0
	**APH^{+a}**	-6380.25933	-6381.95903	-6380.37001	2.52255	-6379.54715	1.2
	**IP**	-6380.23940	-6381.94395	-6380.34264	2.51862	-6379.52857	12.8
	**YI**	-6380.25425	-6381.95597	-6380.34900	2.52024	-6379.53048	11.6
	**$YIH^{+\,a}$**	-6380.25170	-6381.95242	-6380.34895	2.51861	-6379.53105	11.3
	**TC**	-6380.27087	-6381.97007	-6380.36693	2.52131	-6379.54483	2.6
	**$TCH^{+\,a}$**	-6380.26542	-6381.96515	-6380.36540	2.51928	-6379.54585	2.0
**Crystallographic water (Model C)**	**AP**	-6456.72502	-6458.45660	-6456.82834	2.54663	-6456.01329	0.0
	**$APH^{+\,a}$**	-6456.71750	-6458.44888	-6456.82408	2.54513	-6456.01032	1.9
	**IP**	-6456.69510	-6458.42979	-6456.79865	2.54562	-6455.98772	16.0
	**YI**	-6456.72457	-6458.45349	-6456.81461	2.54778	-6455.99576	11.0
	**$YIH^{+\,a}$**	-6456.71868	-6458.44791	-6456.81255	2.54625	-6455.99553	11.1
	**TC**	-6456.72875	-6458.45789	-6456.81906	2.54904	-6455.99916	8.9
	**$TCH^{+\,a}$**	-6913.97667	-6915.84279	-6914.10174	2.61823	-6913.33804	8.5
**Benzoylformate (Model D)**	**AP**	-6913.99977	-6915.86226	-6914.13179	2.63767	-6913.35662	0.0
	**APH^+**	-6913.98709	-6915.85037	-6914.12303	2.63451	-6913.35180	3.0
	**IP**	-6914.00520	-6915.86876	-6914.13032	2.63584	-6913.35804	-0.9
	**YI**	-6914.00338	-6915.86418	-6914.12611	2.63507	-6913.35183	3.0
	**YIH^+**	-6914.00856	-6915.86697	-6914.12503	2.63632	-6913.34713	6.0
	**TC**	-6914.02630	-6915.88361	-6914.14442	2.63727	-6913.36446	-4.9
	**TCH^+**	-6914.02416	-6915.88291	-6914.14564	2.63779	-6913.36660	-6.3
***R*-Mandelate (Model E)**	**AP**	-6915.23515	-6917.09728	-6915.36515	2.66107	-6914.56621	0.0
	**APH^+**	-6915.23045	-6917.09363	-6915.36243	2.65900	-6914.56661	-0.3
	**IP**	-6915.22148	-6917.08475	-6915.34650	2.65985	-6914.54993	10.2

	**YI**	-6915.21059	-6917.07054	-6915.32913	2.65988	-6914.52920	23.2
	**YIH^+**	-6915.20512	-6917.06794	-6915.33055	2.65936	-6914.53401	20.2
	**TC**	-6915.23856	-6917.09747	-6915.35729	2.66259	-6914.55361	7.9
	**TCH^+**	-6915.22551	-6917.08637	-6915.34831	2.66092	-6914.54825	11.3

[a] These states have the N1'-H bond restrained to 1.15 Å in the geometry optimization.

$\boldsymbol{E}_{\mathbf{el}}$ = Energy of the optimized geometry at B3LYP/6-31G(d,p) level.

$\boldsymbol{E}_{\mathbf{bb}}$ = Single-point energy of the optimized geometry at B3LYP/6-311+G(2d,2p) level.

$\boldsymbol{E}_{\mathbf{solv}}$ = Single-point energy of the optimized geometry at B3LYP/6-31G(d,p) and SMD solvation method with $\varepsilon = 4$

$\boldsymbol{E}_{\mathbf{tot}} = E_{bb} + (E_{solv} - E_{el}) + ZPE$

$\boldsymbol{E}_{\mathbf{rel}}$ = Energy relative to **AP** in each respective model

All calculations include empirical dispersion according to Grimme's D3(BJ) method.

8. Cartesian coordinates of optimized structures

Cofactor alone
Model A

AP

C	1.7589	1.5106	0.0781
C	1.2530	0.4114	0.7730
C	2.0354	-0.7595	0.6971
N	1.6526	-1.9482	1.3110
N	3.1485	-0.8083	-0.0387
C	3.5212	0.3019	-0.6920
N	2.8721	1.4817	-0.6467
S	-2.5493	-1.5611	-0.7980
C	-1.1872	-1.2410	0.1524
C	4.7656	0.2389	-1.5215
N	-1.1972	-0.0079	0.6446
C	-2.3125	0.7586	0.2802
C	-2.4646	2.1683	0.7549
C	-0.0608	0.4934	1.4908
H	-0.3720	-1.9328	0.3478
H	2.3827	-2.6495	1.2333
H	-0.2920	1.5259	1.7431
H	-0.0892	-0.0804	2.4203
H	4.5587	0.6002	-2.5327
H	5.5242	0.9047	-1.0971
H	5.1551	-0.7777	-1.5575
H	1.2286	2.4633	0.1129
H	-2.4876	2.2254	1.8476
H	-1.6532	2.8060	0.3903
H	-3.4009	2.5857	0.3870
C	-3.1677	0.0513	-0.5219
C	-4.4706	0.4687	-1.1282
H	-5.3073	-0.0652	-0.6670
H	-4.6356	1.5378	-0.9904
H	-4.4896	0.2640	-2.2021
H	1.3121	-1.8670	2.2624

IP

C	-1.9121	1.3430	-0.5982
C	-1.2056	0.2075	-0.8043
C	-1.7469	-1.0614	-0.2980
N	-1.1146	-2.1624	-0.5238
N	-2.9562	-1.0228	0.4085
C	-3.5775	0.0960	0.5813
N	-3.0934	1.3000	0.0971
S	2.9870	-1.4196	0.4860
C	1.6249	-1.3539	-0.5190
C	-4.8777	0.1560	1.3234
N	1.2610	-0.1066	-0.7841
C	2.0593	0.8793	-0.1853
C	1.7604	2.3278	-0.3965
C	0.0525	0.1812	-1.6150
H	1.0061	-2.1878	-0.8367
H	-1.6425	-2.9406	-0.1238
H	0.2320	1.1249	-2.1286
H	-0.0006	-0.6173	-2.3569
H	-4.8015	0.8194	2.1916
H	-5.6761	0.5366	0.6771
H	-5.1396	-0.8455	1.6596
H	-1.6100	2.3146	-0.9729
H	1.8453	2.6086	-1.4513
H	0.7523	2.5760	-0.0520
H	-3.6328	2.1433	0.2309
H	2.4646	2.9421	0.1630
C	3.0689	0.3300	0.5532
C	4.1559	1.0021	1.3311
H	5.1337	0.8304	0.8705
H	3.9901	2.0793	1.3752
H	4.1965	0.6273	2.3577

YIH^+

C	1.9316	1.4317	0.3610
C	1.2029	0.3262	0.6698
C	1.7944	-0.9508	0.3130
N	1.1596	-2.0844	0.5186
N	3.0483	-1.0121	-0.2340
C	3.6968	0.0864	-0.5007
N	3.1551	1.3168	-0.2399
S	-2.9106	-1.5272	-0.3590
C	-1.4712	-1.3379	0.5430
C	5.0638	0.0461	-1.1054
N	-1.2784	-0.0135	0.6934
C	-2.2176	0.8802	0.1275
C	-2.0562	2.3604	0.2686
C	-0.0901	0.4311	1.4446
H	1.6400	-2.9295	0.2308
H	-0.2397	1.4587	1.7742
H	-0.0252	-0.1957	2.3374
H	5.0790	0.5730	-2.0653
H	5.7939	0.5248	-0.4442
H	5.3518	-0.9916	-1.2634
H	1.6005	2.4385	0.5866
H	-2.1107	2.6820	1.3147
H	-1.1032	2.7075	-0.1468
H	-2.8508	2.8770	-0.2697
C	-3.2133	0.2010	-0.5014
C	-4.4244	0.7234	-1.2068
H	-5.3400	0.4392	-0.6784
H	-4.4028	1.8129	-1.2753
H	-4.4916	0.3249	-2.2235
H	0.0907	-2.0697	0.6822
H	3.6857	2.1500	-0.4612

TCH^+

C	-2.2195	1.5196	-0.3289
C	-1.1279	0.8355	-0.7562
C	-1.1128	-0.5766	-0.4859
N	-0.0730	-1.3061	-0.8683
N	-2.1413	-1.2057	0.1431
C	-3.1803	-0.4999	0.5111
N	-3.2402	0.8486	0.2950
S	2.5939	-1.4650	-0.3676
C	1.2384	-0.7096	-1.3185
C	-4.3483	-1.1496	1.1806
N	1.2830	0.6978	-1.0241
C	1.8008	0.9279	0.2945
C	1.5973	2.2748	0.9090
C	2.5243	-0.0916	0.7867
C	3.3107	-0.1981	2.0518
C	0.0819	1.4065	-1.4568
H	1.3511	-0.9141	-2.3846
H	-0.0847	-2.2903	-0.6174
H	-0.0164	1.2774	-2.5409
H	-4.5939	-0.6453	2.1206
H	-5.2310	-1.1090	0.5329
H	-2.3483	2.5878	-0.4582
H	1.9734	3.0648	0.2488
H	2.1353	2.3500	1.8532
H	0.5402	2.4842	1.1147
H	3.2269	0.7140	2.6454
H	4.3737	-0.3635	1.8432
H	2.9627	-1.0354	2.6659
H	0.1886	2.4753	-1.2689
H	-4.1080	-2.1918	1.3830
H	-4.0598	1.3597	0.5997

Empty cavity

Model B

AP

C	11.5114	5.5184	-1.5740
C	10.8579	4.1408	-1.7482
C	10.0720	3.6562	-0.5625
C	9.7913	4.2941	0.6237
N	9.5102	2.3878	-0.5509
C	8.9016	2.2671	0.6154
N	9.0437	3.3951	1.3627
H	10.7588	6.2925	-1.3886
H	11.6280	3.3935	-1.9779
H	10.1973	4.1557	-2.6254
H	10.0423	5.2727	0.9999
H	8.3502	1.4118	0.9818
H	8.6371	3.5461	2.2724
C	-9.4680	1.9224	-4.3238
C	-8.3756	2.6355	-3.5072
C	-7.2895	1.7008	-3.0226
C	-6.2428	1.3057	-3.8687
C	-7.2957	1.1830	-1.7227
C	-5.2458	0.4345	-3.4383
C	-6.3038	0.3121	-1.2720
C	-5.2628	-0.0687	-2.1303
O	-4.2556	-0.9010	-1.7670
H	-9.9588	1.1505	-3.7221
H	-8.8348	3.1368	-2.6468
H	-7.9299	3.4253	-4.1246
H	-6.2011	1.6955	-4.8830
H	-8.0760	1.4909	-1.0309
H	-4.4350	0.1421	-4.0970
H	-6.3157	-0.0306	-0.2457
H	-4.3044	-1.1755	-0.8170
C	-0.1697	-6.7752	-5.1636
C	-0.1913	-6.5664	-3.6457
C	-1.4157	-5.8771	-3.0778
C	-1.7712	-6.1039	-1.7421
C	-2.2052	-4.9838	-3.8150
C	-2.8640	-5.4721	-1.1584
C	-3.3110	-4.3483	-3.2492
C	-3.6469	-4.5985	-1.9174
O	-4.7614	-3.9970	-1.4070
H	-0.1524	-5.8231	-5.7038
H	-0.0805	-7.5371	-3.1479
H	0.7026	-5.9913	-3.3637
H	-1.1819	-6.7941	-1.1448
H	-1.9654	-4.7812	-4.8540
H	-3.1179	-5.6659	-0.1191
H	-3.9155	-3.6500	-3.8170
H	-4.7670	-4.0981	-0.4455
C	4.9875	-4.3007	-5.9139
C	4.5141	-4.1976	-4.4739
O	4.2960	-3.1148	-3.9253
C	4.4778	-3.1495	-6.7791
H	6.0851	-4.2828	-5.8747
H	4.7222	-2.1949	-6.3088
H	3.3903	-3.1969	-6.8942
H	4.9267	-3.1786	-7.7751
N	4.4213	-5.3883	-3.8260
C	4.5262	-5.5148	-2.3752
C	3.2237	-5.5889	-1.5809
C	2.5060	-4.2505	-1.3581
C	1.2739	-4.4732	-0.4763
C	3.4432	-3.2114	-0.7329
H	4.6496	-6.2096	-4.3658
H	5.1287	-4.6833	-2.0077
H	3.4752	-6.0047	-0.5953
H	2.5464	-6.3087	-2.0571
H	2.1837	-3.8722	-2.3345
H	1.5739	-4.8138	0.5221
H	0.6930	-3.5608	-0.3346
H	0.6020	-5.2219	-0.9039
H	4.2501	-2.9277	-1.4088
H	3.8673	-3.5831	0.2057
H	2.8959	-2.2975	-0.5014

C 9.5105 -5.2180 -1.3683
C 8.2681 -4.4440 -0.9044
C 8.2305 -3.0069 -1.3793
C 7.2467 -2.5633 -2.2693
C 9.1677 -2.0760 -0.9085
C 7.1732 -1.2230 -2.6573
C 9.1103 -0.7395 -1.2980
C 8.1040 -0.3042 -2.1674
H 10.4281 -4.7388 -1.0154
H 8.2275 -4.4585 0.1923
H 7.3674 -4.9634 -1.2496
H 6.5141 -3.2606 -2.6573
H 9.9360 -2.3979 -0.2101
H 6.3865 -0.9146 -3.3401
H 9.8209 -0.0102 -0.9243
H 8.0747 0.7443 -2.4485
C -2.5742 0.8441 0.4263
C -1.3164 1.1261 -0.0707
C -1.1889 2.3320 -0.8187
N 0.0024 2.8015 -1.2504
N -2.2938 3.0411 -1.1477
C -3.4728 2.6646 -0.6434
N -3.6466 1.6182 0.1808
S 2.4318 -0.3743 -2.6324
C 1.7921 0.2732 -1.2065
C -4.6820 3.4627 -1.0199
N 0.5991 -0.2318 -0.9163
C 0.1251 -1.1822 -1.8255
C -1.1930 -1.8467 -1.6162
C 1.0163 -1.3758 -2.8473
C 0.8901 -2.2641 -4.0433
C -0.1684 0.2237 0.2728
H 2.2882 1.0019 -0.5743
H 0.0289 3.6924 -1.7504
H -0.5190 -0.6702 0.7929
H 0.5448 0.7335 0.9163
H -5.1527 3.0155 -1.9016
H -5.4046 3.4421 -0.2054
H -4.4097 4.4910 -1.2599
H -2.7485 -0.0299 1.0503
H -1.2993 -2.1973 -0.5856
H -1.2830 -2.7102 -2.2741
H -2.0259 -1.1724 -1.8369
H 0.2469 -3.1169 -3.8129
H 1.8705 -2.6351 -4.3513
C 2.4643 -2.4817 7.1084
C 1.6856 -1.1650 6.9822
C 2.3851 -0.1470 6.0707
O 2.5390 -0.5259 4.8326
O 2.7525 0.9427 6.5212
C -7.9311 2.7694 4.0749
C -7.1059 1.6026 3.5436
C -6.3667 1.9308 2.2624
O -6.5300 2.9754 1.6407
O -5.5277 0.9777 1.9000
H -8.4282 2.4921 5.0090
H -7.2997 3.6388 4.2703
H -7.7342 0.7247 3.3440
H -6.3587 1.2692 4.2724
C -0.3642 2.1749 5.1491
C -0.2904 1.5609 3.7494
C 0.8817 2.0086 2.9344
C 0.9413 2.8431 1.8373
N 2.1604 1.5098 3.0747
C 2.9118 2.0149 2.0567
N 2.2006 2.8209 1.2681
H 0.5374 1.9541 5.7279
H -0.2754 0.4682 3.8244
H -1.1975 1.8099 3.1875
H 0.1379 3.4315 1.4186
H 3.9688 1.8166 1.9459
C 5.6882 7.3426 2.1978
C 5.3352 6.1081 3.0354
C 6.0498 4.8114 2.6109
C 5.8067 3.6981 3.6406
C 5.6440 4.3511 1.2049
H 5.3842 7.2241 1.1523
H 4.2510 5.9353 3.0009
H 5.5771 6.3112 4.0869
H 7.1289 5.0370 2.5949
H 4.7373 3.4791 3.7321
H 6.3078 2.7625 3.3642
H 6.1634 3.9890 4.6346
H 4.5593 4.2122 1.1393
H 6.1229 3.3981 0.9579
H 5.9404 5.0743 0.4388
H 0.4415 -1.7176 -4.8804
H -8.6944 3.0690 3.3555
H 12.2068 5.5196 -0.7280
H 12.0688 5.7999 -2.4712
H -10.2319 2.6289 -4.6639
H -9.0387 1.4331 -5.2041
H -0.4652 3.2634 5.0893
H -1.2295 1.7809 5.6913
H 6.7687 7.5292 2.2074
H 5.1922 8.2377 2.5855
H 0.6912 -1.3684 6.5605
H 1.5395 -0.7016 7.9611
H 2.6566 -2.9089 6.1195
H 1.9151 -3.2153 7.7077
H 3.4333 -2.3127 7.5893
H 9.4987 -6.2445 -0.9886
H 9.5589 -5.2566 -2.4616
H 5.1028 -6.4277 -2.1922
H 0.7239 -7.3331 -5.4584
H -1.0481 -7.3339 -5.5015
H 4.7078 -5.2707 -6.3415
H -4.9073 1.2800 1.1350
C 2.1251 6.9081 -3.4514
C 0.9034 6.2656 -2.8388
O 0.8044 5.0407 -2.7358
N -0.0750 7.1139 -2.4194
C -1.4056 6.5818 -2.1869
C -2.0281 6.1347 -3.5202
O -1.8200 6.7776 -4.5466
N -2.8031 5.0305 -3.4305
C -3.3486 4.3817 -4.6167
C -2.8614 2.9290 -4.7163
C -1.3356 2.7511 -4.6599
C -0.9795 1.2629 -4.5647
C -0.6195 3.4328 -5.8300
H 3.0091 6.3349 -3.1688
H 2.0258 6.8668 -4.5411
H -1.3522 5.7606 -1.4701
H -2.0244 7.3714 -1.7521
H -3.3127 2.3555 -3.8986
H -3.2536 2.4957 -5.6471
H -0.9777 3.2288 -3.7439
H 0.4631 3.2870 -5.7554
H -0.9511 3.0204 -6.7915
H -0.8051 4.5103 -5.8373
H -1.3079 0.7185 -5.4594
H -1.4582 0.7971 -3.6959
H 0.1028 1.1264 -4.4668
H -3.0409 4.9800 -5.4759
C -4.5521 -3.1288 3.5644
C -3.1504 -2.5345 3.7245
O -2.9641 -1.3208 3.7944
C -5.5095 -2.1809 2.8265
C -5.2891 -2.1346 1.3263
N -6.3160 -2.5482 0.5559
O -4.2277 -1.7238 0.8238
N -2.1368 -3.4263 3.8870
C -0.7825 -2.9505 4.1212
C -0.1381 -2.4459 2.8265
O -0.6089 -2.7123 1.7124
C -0.0463 -4.2200 4.5946
C -0.7123 -5.3147 3.7473
C -2.1858 -4.8779 3.6643
N 1.0159 -1.7864 3.0197
C 1.9500 -1.5846 1.9455
C 3.3526 -2.0202 2.3602
O 3.5816 -3.1068 2.8808
N 4.2944 -1.0903 2.0661
C 5.7196 -1.2655 2.2414
C 6.3064 -0.4029 3.3845
O 7.4675 0.0097 3.3129
C 6.4347 -1.0014 0.9284
O 6.0065 0.2767 0.4449
N 5.5000 -0.2224 4.4482
C 5.9914 0.3647 5.6871
H -6.5439 -2.4670 3.0340
H -5.3367 -1.1625 3.1842
H -4.5243 -4.1072 3.0776
H -0.2362 -4.3751 5.6613
H 1.0320 -4.1515 4.4381
H -0.6013 -6.3159 4.1700
H -0.2766 -5.3148 2.7445
H -2.7964 -5.3666 4.4341
H -2.6162 -5.1122 2.6853
H -0.7846 -2.1515 4.8663
H 1.9705 -0.5410 1.6274
H 1.6286 -2.1980 1.1050
H 7.5110 -1.0145 1.1094
H 6.1792 -1.7866 0.2090
H 6.4784 0.4280 -0.3863
H 5.8717 -2.3116 2.5298
H 6.7543 1.1084 5.4536
H 2.2508 7.9513 -3.1516
H -4.4451 4.3945 -4.5767
H -2.6727 4.4450 -2.6049
H -0.0406 8.0516 -2.7935
H 6.4430 -0.3986 6.3341
H 5.1510 0.8305 6.2039
H 4.5282 -0.5305 4.4315
H 1.3836 -1.5158 3.9483
H -7.1386 -3.0278 0.9260
H -6.1953 -2.5095 -0.4476
H 4.0185 -0.2501 1.5808
H -4.9373 -3.3008 4.5768
C -10.4257 -5.2186 2.1683
C -9.5409 -4.5138 1.1657
O -8.3939 -4.1688 1.4721
C -10.7220 -4.2360 3.3053
N -10.0935 -4.2175 -0.0296
C -9.3902 -3.4241 -1.0201
C -8.6256 6.9745 3.1146
C -8.5156 5.7269 2.2447
C -8.4444 6.0244 0.7485
N -8.0355 4.9573 -0.0056
O -8.7723 7.0984 0.2604
H -9.7865 -3.8847 3.7458
H -11.3203 -4.7091 4.0886
H -11.2741 -3.3657 2.9369
H -11.3515 -5.5812 1.7086
H -8.4493 -3.9058 -1.3035
H -7.6472 5.1225 2.5191
H -9.3898 5.0805 2.4000
H -7.5718 4.1714 0.4341
H -7.8730 5.1279 -0.9866
H -9.4766 7.5857 2.8056
H -8.7483 6.7018 4.1671
H -7.7299 7.5959 3.0238
H -9.8787 -6.0857 2.5499
H -9.1615 -2.4238 -0.6382
H -10.0145 -3.3268 -1.9086
H -11.0405 -4.5084 -0.2135
H 0.8274 2.6289 -0.6892
H 2.4528 0.8138 3.8084

APH$^+$

C 11.4278 5.5960 -1.3822
C 10.6047 4.3247 -1.6319
C 9.9456 3.7431 -0.4128
C 9.9213 4.2140 0.8796
N 9.2286 2.5570 -0.4875
C 8.7837 2.3193 0.7336
N 9.1833 3.2909 1.5990
H 10.8023 6.3980 -0.9758
H 11.2436 3.5528 -2.0796

H	9.8298	4.5313	-2.3821
H	10.3501	5.0880	1.3425
H	8.1752	1.4845	1.0540
H	8.9349	3.3417	2.5749
C	-9.5124	1.8968	-4.2891
C	-8.5698	2.6845	-3.3637
C	-7.4066	1.8479	-2.8824
C	-6.2973	1.6161	-3.7097
C	-7.4152	1.2409	-1.6217
C	-5.2350	0.8191	-3.2924
C	-6.3584	0.4424	-1.1834
C	-5.2536	0.2423	-2.0174
O	-4.1524	-0.4597	-1.6332
H	-9.9251	1.0266	-3.7687
H	-9.1326	3.0606	-2.5016
H	-8.1953	3.5649	-3.9004
H	-6.2647	2.0681	-4.6981
H	-8.2495	1.4212	-0.9489
H	-4.3805	0.6381	-3.9340
H	-6.3623	0.0407	-0.1765
H	-4.3027	-0.9377	-0.7922
C	-0.1315	-6.6812	-5.3970
C	-0.1575	-6.5228	-3.8730
C	-1.2969	-5.7046	-3.3037
C	-1.4930	-5.6835	-1.9161
C	-2.1578	-4.9351	-4.0943
C	-2.4773	-4.9008	-1.3268
C	-3.1690	-4.1628	-3.5222
C	-3.3248	-4.1332	-2.1352
O	-4.2995	-3.3476	-1.6059
H	-0.0141	-5.7155	-5.8992
H	-0.1691	-7.5163	-3.4076
H	0.7920	-6.0646	-3.5610
H	-0.8573	-6.2956	-1.2829
H	-2.0465	-4.9326	-5.1735
H	-2.5826	-4.8614	-0.2463
H	-3.8301	-3.5561	-4.1319
H	-4.0279	-3.0052	-0.7212
C	5.0035	-4.1353	-6.0555
C	4.5772	-4.0594	-4.5982
O	4.4793	-2.9873	-3.9970
C	4.6031	-2.8921	-6.8472
H	6.0968	-4.2398	-6.0467
H	4.9647	-1.9954	-6.3401
H	3.5145	-2.8138	-6.9302
H	5.0201	-2.9200	-7.8570
N	4.3849	-5.2643	-3.9982
C	4.5478	-5.4696	-2.5597
C	3.2873	-5.4973	-1.7003
C	2.6343	-4.1364	-1.4224
C	1.4612	-4.3284	-0.4575
C	3.6439	-3.1358	-0.8486
H	4.5193	-6.0769	-4.5814
H	5.2235	-4.6989	-2.1846
H	3.5758	-5.9364	-0.7350
H	2.5543	-6.1833	-2.1436
H	2.2551	-3.7437	-2.3736
H	1.8276	-4.5343	0.5549
H	0.8212	-3.4469	-0.3972
H	0.8227	-5.1579	-0.7660
H	4.3976	-2.8480	-1.5813
H	4.1334	-3.5518	0.0377
H	3.1433	-2.2195	-0.5315
C	9.5275	-5.1595	-1.5339
C	8.3421	-4.3794	-0.9468
C	8.2858	-2.9323	-1.3871
C	7.3194	-2.4908	-2.2971
C	9.1886	-1.9926	-0.8696
C	7.2252	-1.1440	-2.6561
C	9.1086	-0.6487	-1.2299
C	8.1174	-0.2148	-2.1167
H	10.4785	-4.6972	-1.2519
H	8.3961	-4.4178	0.1483
H	7.4066	-4.8781	-1.2246
H	6.6136	-3.1941	-2.7230
H	9.9441	-2.3146	-0.1571
H	6.4505	-0.8396	-3.3540
H	9.7869	0.0878	-0.8147
H	8.0698	0.8409	-2.3662
C	-2.6723	0.8651	0.5839
C	-1.4437	1.1604	0.0469
C	-1.3491	2.3976	-0.6680
N	-0.1906	2.8938	-1.1187
N	-2.4642	3.1211	-0.9421
C	-3.6232	2.7586	-0.4112
N	-3.7324	1.6806	0.3948
S	2.5589	-0.0730	-2.3648
C	1.7907	0.4688	-0.9596
C	-4.8452	3.5571	-0.7073
N	0.5768	-0.0577	-0.8206
C	0.1963	-0.9391	-1.8362
C	-1.1235	-1.6285	-1.7830
C	1.1792	-1.0585	-2.7834
C	1.1737	-1.8645	-4.0431
C	-0.2788	0.2598	0.3497
H	2.2239	1.1635	-0.2421
H	-0.1578	3.8022	-1.5910
H	-0.6194	-0.6922	0.7656
H	0.3751	0.7392	1.0768
H	-5.3389	3.1391	-1.5912
H	-5.5434	3.4941	0.1307
H	-4.5714	4.5909	-0.9201
H	-2.8582	-0.0206	1.1879
H	-1.2510	-2.1376	-0.8229
H	-1.1854	-2.3794	-2.5692
H	-1.9579	-0.9336	-1.9165
H	0.5129	-2.7277	-3.9335
H	2.1794	-2.2226	-4.2770
C	2.4423	-2.7698	7.0147
C	1.6358	-1.4668	6.9337
C	2.3190	-0.4117	6.0557
O	2.4747	-0.7442	4.8045
O	2.6800	0.6658	6.5409
C	-7.9971	2.4810	4.1358
C	-7.1858	1.3252	3.5570
C	-6.4206	1.6881	2.2855
O	-6.6182	2.7992	1.7442
O	-5.5932	0.8063	1.8589
H	-8.4935	2.1807	5.0649
H	-7.3564	3.3380	4.3538
H	-7.8311	0.4685	3.3207
H	-6.4523	0.9486	4.2792
C	-0.4266	1.9222	5.2044
C	-0.3142	1.4442	3.7550
C	0.8993	1.9312	3.0262
C	1.0209	2.8013	1.9597
N	2.1708	1.4372	3.2296
C	2.9804	1.9838	2.2814
N	2.3152	2.8120	1.4766
H	0.4541	1.6369	5.7866
H	-0.3318	0.3484	3.7280
H	-1.1935	1.7748	3.1903
H	0.2446	3.4101	1.5186
H	4.0421	1.7888	2.2271
C	5.5816	7.2405	2.4364
C	5.4234	5.9348	3.2231
C	6.1783	4.7271	2.6368
C	6.1008	3.5335	3.6007
C	5.6690	4.3411	1.2410
H	5.1624	7.1594	1.4287
H	4.3584	5.6761	3.2981
H	5.7700	6.0962	4.2526
H	7.2352	5.0193	2.5370
H	5.0590	3.2601	3.8005
H	6.5998	2.6437	3.1997
H	6.5581	3.7715	4.5682
H	4.5895	4.1544	1.2535
H	6.1642	3.4316	0.8852
H	5.8667	5.1293	0.5081
H	0.8090	-1.2606	-4.8812
H	-8.7610	2.8100	3.4311
H	12.2369	5.4086	-0.6686
H	11.8746	5.9572	-2.3125
H	-10.3457	2.5188	-4.6312
H	-8.9747	1.5311	-5.1699
H	-0.5145	3.0124	5.2461
H	-1.3129	1.4886	5.6772
H	6.6388	7.5128	2.3338
H	5.0722	8.0681	2.9404
H	0.6452	-1.6757	6.5059
H	1.4835	-1.0380	7.9272
H	2.6242	-3.1690	6.0122
H	1.9179	-3.5286	7.6047
H	3.4159	-2.5933	7.4835
H	9.5347	-6.1940	-1.1760
H	9.4806	-5.1745	-2.6277
H	5.0688	-6.4266	-2.4484
H	0.7070	-7.3149	-5.6999
H	-1.0529	-7.1407	-5.7691
H	4.6095	-5.0457	-6.5231
H	-4.6369	1.3971	0.9319
C	2.0319	6.9590	-3.2309
C	0.7892	6.3512	-2.6253
O	0.6855	5.1317	-2.4626
N	-0.2036	7.2160	-2.2847
C	-1.5440	6.6996	-2.0697
C	-2.1304	6.2068	-3.4037
O	-1.8784	6.8087	-4.4447
N	-2.9242	5.1158	-3.3039
C	-3.4160	4.4215	-4.4891
C	-2.7506	3.0446	-4.6263
C	-1.2147	3.0583	-4.6824
C	-0.6719	1.6271	-4.5812
C	-0.6785	3.7792	-5.9239
H	2.9024	6.3845	-2.9124
H	1.9527	6.8866	-4.3207
H	-1.5140	5.9069	-1.3198
H	-2.1704	7.5074	-1.6819
H	-3.0648	2.4330	-3.7729
H	-3.1512	2.5513	-5.5232
H	-0.8503	3.6061	-3.8075
H	0.4163	3.7527	-5.9411
H	-1.0392	3.3013	-6.8436
H	-0.9838	4.8288	-5.9368
H	-0.9940	1.0190	-5.4360
H	-1.0252	1.1337	-3.6691
H	0.4236	1.6245	-4.5616
H	-3.2093	5.0667	-5.3436
C	-4.5620	-3.3654	3.4378
C	-3.1379	-2.8178	3.5633
O	-2.9165	-1.6157	3.7082
C	-5.4916	-2.3807	2.7031
C	-5.2706	-2.3758	1.2085
N	-6.2928	-2.7472	0.4319
O	-4.1633	-2.0398	0.7098
N	-2.1332	-3.7330	3.6154
C	-0.7712	-3.2835	3.8589
C	-0.1747	-2.5876	2.6347
O	-0.6391	-2.7407	1.4965
C	-0.0045	-4.5981	4.1140
C	-0.7255	-5.5822	3.1812
C	-2.2013	-5.1619	3.2775
N	0.9453	-1.8934	2.9054
C	1.8901	-1.5492	1.8782
C	3.2848	-2.0592	2.2343
O	3.4887	-3.2004	2.6361
N	4.2459	-1.1272	2.0271
C	5.6681	-1.3393	2.1861
C	6.2661	-0.5456	3.3735
O	7.4386	-0.1622	3.3363
C	6.3897	-1.0193	0.8880
O	5.9626	0.2786	0.4556
N	5.4503	-0.3810	4.4331
C	5.9448	0.1546	5.6947
H	-6.5351	-2.6249	2.9164
H	-5.2875	-1.3606	3.0426
H	-4.5857	-4.3500	2.9651
H	-0.1233	-4.8913	5.1620
H	1.0616	-4.4957	3.9001
H	-0.5749	-6.6287	3.4559
H	-0.3722	-5.4388	2.1565

H -2.7279 -5.7220 4.0612
H -2.7296 -5.3195 2.3325
H -0.7433 -2.6004 4.7118
H 1.9247 -0.4712 1.7195
H 1.5717 -2.0306 0.9549
H 7.4646 -1.0391 1.0749
H 6.1391 -1.7727 0.1345
H 6.4430 0.4680 -0.3630
H 5.8012 -2.4019 2.4170
H 6.6907 0.9246 5.4902
H 2.1631 8.0093 -2.9599
H -4.5021 4.2944 -4.4121
H -2.8437 4.5623 -2.4530
H -0.1528 8.1381 -2.6940
H 6.4180 -0.6288 6.2994
H 5.1006 0.5796 6.2394
H 4.4767 -0.6833 4.4020
H 1.2983 -1.7127 3.8584
H -7.1207 -3.2167 0.8028
H -6.1133 -2.8361 -0.5609
H 3.9823 -0.2321 1.6438
H -4.9342 -3.4981 4.4604
C -10.4060 -5.4629 1.9650
C -9.5291 -4.7131 0.9884
O -8.3906 -4.3583 1.3110
C -10.7207 -4.5210 3.1307
N -10.0819 -4.3907 -0.2006
C -9.3897 -3.5547 -1.1631
C -8.7295 6.7088 3.3131
C -8.6454 5.4496 2.4591
C -8.6389 5.7110 0.9554
N -8.2750 4.6178 0.2157
O -8.9684 6.7797 0.4532
H -9.7924 -4.1688 3.5848
H -11.3137 -5.0275 3.8972
H -11.2851 -3.6481 2.7882
H -11.3244 -5.8256 1.4906
H -8.4697 -4.0374 -1.5082
H -7.7617 4.8548 2.7034
H -9.5050 4.7985 2.6648
H -7.7819 3.8469 0.6621
H -8.1502 4.7665 -0.7747
H -9.6005 7.3079 3.0363
H -8.8005 6.4503 4.3739
H -7.8460 7.3388 3.1729
H -9.8462 -6.3328 2.3209
H -9.1259 -2.5868 -0.7260
H -10.0395 -3.3870 -2.0222
H -11.0216 -4.6954 -0.3984
H 0.6813 2.6066 -0.7037
H 2.4206 0.6881 3.9281

IP

C 11.6909 5.0205 -2.3691
C 11.7871 3.6380 -1.7048
C 10.5584 3.1879 -0.9609
C 9.2521 3.6107 -1.0724
N 10.6447 2.1988 0.0098
C 9.4222 2.0361 0.4789
N 8.5390 2.8623 -0.1501
H 10.8989 5.0460 -3.1257
H 12.6201 3.6254 -0.9932
H 12.0425 2.8879 -2.4662
H 8.7789 4.3571 -1.6903
H 9.1043 1.3623 1.2630
H 7.5541 2.9240 0.0552
C -9.4488 1.8702 -4.3227
C -8.1587 2.4309 -3.6982
C -7.2520 1.3562 -3.1370
C -6.6144 0.4413 -3.9886
C -7.0332 1.2237 -1.7609
C -5.7930 -0.5650 -3.4902
C -6.2126 0.2191 -1.2433
C -5.5812 -0.6755 -2.1109
O -4.7604 -1.6799 -1.6743
H -10.0318 1.3223 -3.5756
H -8.4189 3.1362 -2.9005
H -7.6188 3.0095 -4.4597
H -6.7571 0.5226 -5.0636
H -7.4846 1.9220 -1.0611
H -5.2933 -1.2637 -4.1531
H -6.0547 0.1711 -0.1718
H -4.5202 -1.5703 -0.7191
C -0.4487 -7.1736 -4.4667
C -0.4285 -6.8132 -2.9774
C -1.7302 -6.2723 -2.4113
C -1.9626 -6.3225 -1.0300
C -2.7281 -5.6973 -3.2113
C -3.1388 -5.8336 -0.4662
C -3.9105 -5.2026 -2.6648
C -4.1272 -5.2813 -1.2877
O -5.3282 -4.8461 -0.8091
H -0.6016 -6.2917 -5.0970
H -0.1454 -7.7004 -2.3980
H 0.3745 -6.0833 -2.8011
H -1.2131 -6.7655 -0.3796
H -2.5924 -5.6377 -4.2861
H -3.2983 -5.8976 0.6074
H -4.6738 -4.7505 -3.2875
H -5.3038 -4.7844 0.1566
C 4.7645 -4.9604 -5.5655
C 4.6504 -4.6816 -4.0811
O 5.0429 -3.6302 -3.5781
C 4.6256 -3.6919 -6.4069
H 5.7558 -5.4066 -5.7220
H 5.3270 -2.9334 -6.0542
H 3.6164 -3.2758 -6.3242
H 4.8238 -3.8973 -7.4619
N 4.1326 -5.7051 -3.3475
C 4.3478 -5.7979 -1.9139
C 3.0964 -5.6622 -1.0504
C 2.4107 -4.2896 -1.0741
C 1.1900 -4.3281 -0.1531
C 3.3614 -3.1502 -0.6848
H 3.8826 -6.5501 -3.8395
H 5.0685 -5.0208 -1.6552
H 3.3897 -5.8897 -0.0161
H 2.3692 -6.4336 -1.3379
H 2.0717 -4.1013 -2.1013
H 1.4858 -4.6022 0.8668
H 0.6935 -3.3624 -0.0828
H 0.4485 -5.0573 -0.4920
H 4.1834 -3.0446 -1.3960
H 3.7773 -3.3107 0.3174
H 2.8226 -2.1972 -0.6709
C 9.3609 -5.5684 -1.0418
C 8.2440 -4.5712 -0.6905
C 8.5353 -3.1396 -1.0958
C 7.7713 -2.5017 -2.0800
C 9.5650 -2.4147 -0.4751
C 8.0228 -1.1690 -2.4252
C 9.8206 -1.0878 -0.8173
C 9.0436 -0.4555 -1.7941
H 10.3037 -5.2906 -0.5608
H 8.0644 -4.6082 0.3922
H 7.3123 -4.8926 -1.1684
H 6.9659 -3.0357 -2.5752
H 10.1648 -2.8929 0.2956
H 7.4209 -0.6986 -3.1987
H 10.6078 -0.5263 -0.3249
H 9.2505 0.5777 -2.0566
C -2.7830 0.9538 0.2789
C -1.5645 1.0796 -0.3108
C -1.2792 2.2804 -1.0888
N -0.0652 2.5203 -1.4893
N -2.3551 3.1347 -1.3517
C -3.5153 2.9251 -0.7748
N -3.7479 1.8899 0.0710
S 1.4608 -0.9274 -3.3125
C 1.0364 -0.0155 -1.9470
C -4.6720 3.8296 -1.0650
N 0.0393 -0.5658 -1.2702
C -0.4719 -1.7487 -1.8222
C -1.6561 -2.4315 -1.2299
C 0.2066 -2.0966 -2.9577
C -0.0416 -3.2429 -3.8829
C -0.4777 0.0988 -0.0331
H 1.3896 0.9769 -1.6876
H 0.0131 3.4057 -1.9940
H -0.7922 -0.6916 0.6456
H 0.3848 0.6065 0.3983
H -5.2664 3.3943 -1.8766
H -5.3319 3.9063 -0.1985
H -4.3166 4.8109 -1.3785
H -3.0569 0.1306 0.9328
H -1.6225 -2.4437 -0.1398
H -1.7040 -3.4638 -1.5739
H -2.5743 -1.9330 -1.5508
H -0.7627 -3.9298 -3.4401
H 0.8783 -3.7969 -4.0936
C 2.6025 -1.7631 7.2547
C 1.9745 -0.3808 7.4695
C 2.4937 0.6453 6.4844
O 2.2678 0.2766 5.2143
O 3.0490 1.6824 6.7907
C -7.6915 3.5022 3.9154
C -7.0757 2.1926 3.4323
C -6.5107 2.2558 2.0064
O -6.9106 3.1607 1.2381
O -5.6657 1.3444 1.7102
H -8.0931 3.4000 4.9295
H -6.9453 4.3040 3.9310
H -7.8272 1.3904 3.4461
H -6.2687 1.8574 4.0922
C -0.1239 2.7658 4.8928
C -0.1413 2.0114 3.5557
C 1.0939 2.2547 2.7447
C 1.2218 2.7840 1.4838
N 2.3617 1.9537 3.2313
C 3.2302 2.2909 2.2943
N 2.5783 2.7875 1.2096
H 0.7680 2.5310 5.4769
H -0.2585 0.9383 3.7388
H -1.0130 2.3079 2.9638
H 0.5078 3.1150 0.7473
H 4.3014 2.1886 2.3577
H 3.0012 3.1552 0.3704
C 6.0167 7.4068 1.3151
C 6.0803 6.3445 2.4141
C 6.4961 4.9408 1.9408
C 6.5215 3.9643 3.1225
C 5.5689 4.4343 0.8237
H 5.2433 7.1811 0.5742
H 5.0990 6.2674 2.9034
H 6.7825 6.6685 3.1922
H 7.5148 5.0058 1.5329
H 5.5225 3.8588 3.5632
H 6.8756 2.9717 2.8285
H 7.1862 4.3277 3.9132
H 4.5240 4.6021 1.1054
H 5.6919 3.3566 0.6499
H 5.7388 4.9552 -0.1233
H -0.4548 -2.8921 -4.8348
H -8.4954 3.8173 3.2509
H 11.4707 5.7969 -1.6295
H 12.6313 5.2764 -2.8652
H -10.0746 2.6734 -4.7247
H -9.2203 1.1772 -5.1387
H -0.1300 3.8475 4.7269
H -1.0055 2.5077 5.4866
H 6.9730 7.4843 0.7841
H 5.7900 8.3920 1.7339
H 0.8870 -0.4426 7.3376
H 2.1715 -0.0041 8.4750
H 2.4591 -2.1080 6.2279
H 2.1640 -2.4977 7.9364
H 3.6803 -1.7336 7.4438
H 9.1003 -6.5798 -0.7140
H 9.5358 -5.5910 -2.1220
H 4.8239 -6.7640 -1.7047

H 0.5029 -7.6249 -4.7637
H -1.2475 -7.8866 -4.6920
H 4.0329 -5.7178 -5.8716
H 2.4713 1.0302 4.5432
H -4.6590 1.7643 0.6348
C 2.3115 6.5281 -4.1892
C 1.2160 6.0427 -3.2614
O 1.3016 4.9775 -2.6525
N 0.1365 6.8779 -3.1755
C -1.1526 6.3493 -2.7602
C -1.8830 5.8507 -4.0153
O -1.7840 6.4795 -5.0714
N -2.5859 4.7154 -3.8616
C -3.2640 4.0820 -4.9842
C -3.4246 2.5801 -4.7337
C -2.1044 1.8036 -4.5775
C -2.3786 0.3869 -4.0649
C -1.3000 1.7686 -5.8818
H 3.2658 6.1011 -3.8781
H 2.0910 6.1785 -5.2041
H -0.9995 5.5619 -2.0229
H -1.7421 7.1477 -2.2975
H -4.0360 2.4308 -3.8355
H -4.0032 2.1492 -5.5611
H -1.4914 2.3119 -3.8254
H -0.3598 1.2228 -5.7435
H -1.8642 1.2667 -6.6778
H -1.0466 2.7734 -6.2323
H -3.0263 -0.1650 -4.7555
H -2.8774 0.4002 -3.0918
H -1.4470 -0.1772 -3.9606
H -2.6846 4.2881 -5.8889
C -4.5111 -2.5271 3.9349
C -3.0727 -1.9920 3.9269
O -2.8281 -0.7886 3.8972
C -5.4450 -1.7258 3.0099
C -5.2187 -2.0245 1.5433
N -6.2043 -2.6603 0.8839
O -4.1624 -1.6989 0.9629
N -2.0720 -2.9100 4.0740
C -0.7008 -2.4643 4.2707
C -0.0319 -2.0489 2.9565
O -0.4268 -2.4657 1.8654
C -0.0029 -3.7343 4.8026
C -0.7153 -4.8506 4.0236
C -2.1748 -4.3697 3.9426
N 1.0761 -1.2912 3.1122
C 2.0296 -1.0926 2.0497
C 3.4517 -1.3327 2.5502
O 3.6970 -2.0344 3.5332
N 4.4167 -0.7173 1.8293
C 5.8426 -0.9016 2.0705
C 6.4242 0.0427 3.1542
O 7.4786 0.6507 2.9680
C 6.5916 -0.7722 0.7572
O 6.2111 0.4586 0.1291
N 5.7106 0.0937 4.3024
C 6.1828 0.8072 5.4835
H -6.4848 -1.9394 3.2718
H -5.2659 -0.6523 3.1273
H -4.5551 -3.5925 3.6994
H -0.1888 -3.8255 5.8774
H 1.0771 -3.7109 4.6399
H -0.6287 -5.8299 4.5002
H -0.2984 -4.9183 3.0157
H -2.7810 -4.7924 4.7534
H -2.6347 -4.6435 2.9877
H -0.6748 -1.6294 4.9760
H 1.9592 -0.0875 1.6238
H 1.8006 -1.8088 1.2576
H 7.6642 -0.7956 0.9562
H 6.3329 -1.6192 0.1132
H 6.7125 0.4901 -0.6986
H 5.9738 -1.9229 2.4476
H 6.9623 1.5005 5.1694
H 2.3846 7.6186 -4.2141
H -4.2510 4.5383 -5.1361
H -2.5167 4.2098 -2.9722
H 0.0439 7.5405 -3.9375
H 6.6068 0.1127 6.2186
H 5.3573 1.3561 5.9418
H 4.9272 -0.5446 4.3999
H 1.3536 -0.9688 4.0366
H -7.0751 -2.9669 1.3210
H -6.0853 -2.7991 -0.1134
H 4.1776 -0.0893 1.0742
H -4.8636 -2.4175 4.9672
C -10.6092 -4.5354 2.8385
C -9.6696 -4.0459 1.7583
O -8.4636 -3.8969 1.9894
C -10.7169 -3.4479 3.9120
N -10.2301 -3.7191 0.5752
C -9.4612 -3.1182 -0.4989
C -8.2764 7.6108 2.5500
C -8.2726 6.3050 1.7642
C -8.1299 6.5020 0.2564
N -7.7881 5.3656 -0.4121
O -8.3336 7.5757 -0.3023
H -9.7244 -3.2098 4.3018
H -11.3475 -3.7764 4.7425
H -11.1490 -2.5309 3.4998
H -11.5954 -4.7857 2.4325
H -8.6301 -3.7689 -0.7877
H -7.4817 5.6302 2.1009
H -9.2108 5.7586 1.9296
H -7.5118 4.5213 0.0830
H -7.6464 5.4385 -1.4076
H -9.0467 8.2865 2.1697
H -8.4615 7.4243 3.6128
H -7.3173 8.1293 2.4558
H -10.1817 -5.4475 3.2653
H -9.0486 -2.1481 -0.2030
H -10.1092 -2.9740 -1.3638
H -11.2244 -3.8358 0.4610

YI

C 11.5239 5.2217 -2.0493
C 10.4290 4.1580 -2.2756
C 9.9386 3.4430 -1.0409
C 8.6387 3.1559 -0.6710
N 10.8118 2.8836 -0.1225
C 10.0518 2.2812 0.7739
N 8.7252 2.4139 0.4939
H 11.1511 6.0444 -1.4309
H 10.8235 3.4072 -2.9746
H 9.5666 4.6121 -2.7786
H 7.6878 3.4175 -1.1083
H 10.4006 1.7380 1.6406
H 7.9669 1.9473 0.9858
C -9.5455 1.8316 -4.3314
C -8.4279 2.5694 -3.5729
C -7.3739 1.6252 -3.0416
C -6.3651 1.1263 -3.8777
C -7.3960 1.1767 -1.7163
C -5.4245 0.2103 -3.4165
C -6.4636 0.2569 -1.2371
C -5.4682 -0.2369 -2.0898
O -4.5377 -1.1498 -1.7094
H -10.0461 1.1122 -3.6752
H -8.8640 3.1380 -2.7429
H -7.9642 3.3025 -4.2444
H -6.3108 1.4662 -4.9092
H -8.1494 1.5658 -1.0355
H -4.6393 -0.1644 -4.0644
H -6.4932 -0.0497 -0.1996
H -4.4822 -1.2769 -0.7303
C -0.3969 -7.0483 -4.8434
C -0.3939 -6.7612 -3.3381
C -1.6466 -6.1113 -2.7805
C -1.9003 -6.1745 -1.4042
C -2.5724 -5.4248 -3.5774
C -3.0309 -5.5905 -0.8412
C -3.7138 -4.8389 -3.0313
C -3.9519 -4.9304 -1.6595
O -5.1089 -4.3928 -1.1708
H -0.4437 -6.1254 -5.4302
H -0.2199 -7.6980 -2.7943
H 0.4702 -6.1223 -3.1041
H -1.2036 -6.7017 -0.7582
H -2.4138 -5.3490 -4.6479
H -3.2055 -5.6572 0.2301
H -4.4257 -4.3065 -3.6519
H -5.1023 -4.4325 -0.2046
C 4.7873 -4.7002 -5.7888
C 4.8492 -4.5543 -4.2828
O 5.5457 -3.7044 -3.7341
C 4.8605 -3.3486 -6.5004
H 5.6450 -5.3222 -6.0780
H 5.7371 -2.7959 -6.1574
H 3.9772 -2.7421 -6.2775
H 4.9226 -3.4783 -7.5841
N 4.1064 -5.4631 -3.5904
C 4.3569 -5.7226 -2.1863
C 3.1235 -5.6456 -1.2930
C 2.4870 -4.2553 -1.1811
C 1.2571 -4.3265 -0.2754
C 3.4870 -3.2033 -0.6863
H 3.6213 -6.1715 -4.1212
H 5.1045 -4.9954 -1.8717
H 3.4212 -5.9839 -0.2907
H 2.3702 -6.3647 -1.6440
H 2.1577 -3.9536 -2.1817
H 1.5357 -4.6452 0.7370
H 0.7582 -3.3633 -0.1837
H 0.5186 -5.0373 -0.6581
H 4.3194 -3.0701 -1.3827
H 3.8933 -3.4814 0.2946
H 3.0006 -2.2304 -0.5894
C 9.3589 -5.4558 -1.2622
C 8.2483 -4.4617 -0.8793
C 8.6203 -3.0091 -1.0981
C 7.9794 -2.2454 -2.0817
C 9.6092 -2.3960 -0.3146
C 8.3156 -0.9003 -2.2689
C 9.9488 -1.0568 -0.5044
C 9.2998 -0.2980 -1.4826
H 10.2698 -5.2721 -0.6841
H 7.9868 -4.6132 0.1766
H 7.3470 -4.6843 -1.4589
H 7.2137 -2.7034 -2.6994
H 10.1144 -2.9735 0.4560
H 7.8161 -0.3290 -3.0476
H 10.7152 -0.5947 0.1091
H 9.5653 0.7440 -1.6256
C -2.7548 0.8501 0.2375
C -1.5054 1.0396 -0.3090
C -1.3178 2.2468 -1.0435
N -0.1051 2.6432 -1.4615
N -2.3755 3.0595 -1.2967
C -3.5625 2.7487 -0.7692
N -3.7852 1.6957 0.0335
S 1.9783 -0.7082 -2.9616
C 1.4726 0.1401 -1.5456
C -4.7356 3.6169 -1.1063
N 0.3211 -0.4504 -1.1625
C -0.1934 -1.5322 -1.9110
C -1.4740 -2.1955 -1.5233
C 0.6145 -1.8194 -2.9669
C 0.4342 -2.8621 -4.0250
C -0.3854 0.0968 0.0159
H 0.6814 1.9846 -1.4445
H 0.0074 3.4964 -2.0089
H -0.7499 -0.7314 0.6226
H 0.3807 0.6223 0.5837
H -5.3302 3.1305 -1.8879
H -5.3733 3.7261 -0.2298
H -4.4123 4.5927 -1.4682
H -2.9569 -0.0028 0.8798
H -1.4868 -2.4633 -0.4635
H -1.6142 -3.1106 -2.0983

H -2.3326 -1.5489 -1.7222
H -0.3299 -3.5847 -3.7280
H 1.3654 -3.4087 -4.2082
C 2.4762 -2.1719 7.1542
C 1.8440 -0.8175 7.4937
C 2.3530 0.2783 6.5813
O 2.0875 0.0109 5.2960
O 2.9353 1.2789 6.9533
C -7.8776 3.0848 3.9911
C -7.1059 1.8726 3.4833
C -6.3837 2.1450 2.1797
O -6.4984 3.2041 1.5690
O -5.6232 1.1425 1.7890
H -8.3579 2.8609 4.9484
H -7.2131 3.9394 4.1344
H -7.7667 1.0109 3.3232
H -6.3541 1.5367 4.2068
C -0.3065 2.4231 4.9943
C -0.2352 1.8547 3.5694
C 1.0618 2.1400 2.8742
C 1.2875 2.7117 1.6452
N 2.2862 1.7858 3.4275
C 3.2228 2.1270 2.5596
N 2.6580 2.6855 1.4593
H 0.5243 2.0720 5.6074
H -0.3962 0.7711 3.6027
H -1.0517 2.2572 2.9608
H 0.6229 3.1056 0.8935
H 4.2855 1.9933 2.6827
H 3.1539 3.0019 0.6395
C 5.7836 7.3325 1.6998
C 5.8826 6.2972 2.8204
C 6.4604 4.9346 2.4011
C 6.4600 3.9767 3.5978
C 5.6891 4.3382 1.2155
H 5.0685 7.0299 0.9287
H 4.8842 6.1313 3.2502
H 6.4984 6.7000 3.6344
H 7.5015 5.0823 2.0801
H 5.4354 3.7964 3.9483
H 6.9056 3.0110 3.3474
H 7.0186 4.4012 4.4393
H 4.6148 4.3637 1.4284
H 5.9809 3.2994 1.0286
H 5.8575 4.9037 0.2944
H 0.1199 -2.4120 -4.9737
H -8.6504 3.3820 3.2811
H 12.3781 4.7766 -1.5334
H 11.8625 5.6328 -3.0049
H -10.2976 2.5307 -4.7111
H -9.1354 1.2757 -5.1807
H -0.2657 3.5167 4.9807
H -1.2419 2.1207 5.4738
H 6.7538 7.4812 1.2115
H 5.4553 8.3023 2.0868
H 0.7553 -0.8735 7.3715
H 2.0555 -0.5230 8.5235
H 2.3381 -2.4194 6.0996
H 2.0415 -2.9696 7.7636
H 3.5537 -2.1546 7.3469
H 9.0421 -6.4870 -1.0759
H 9.6142 -5.3605 -2.3220
H 4.8142 -6.7171 -2.0866
H 0.5170 -7.5755 -5.1340
H -1.2509 -7.6702 -5.1288
H 3.8875 -5.2521 -6.0866
H 2.3178 0.7987 4.6704
H -5.0026 1.4206 0.9976
C 2.1351 6.6658 -3.8717
C 0.9586 6.0519 -3.1513
O 0.8954 4.8408 -2.9360
N -0.0219 6.9222 -2.7722
C -1.3487 6.3935 -2.5081
C -1.9801 5.9065 -3.8234
O -1.7423 6.4943 -4.8775
N -2.7943 4.8399 -3.6846
C -3.3932 4.1724 -4.8321
C -3.0632 2.6754 -4.8272
C -1.5639 2.3448 -4.7700
C -1.3593 0.8384 -4.5782
C -0.8007 2.8735 -5.9887
H 3.0260 6.0654 -3.6860
H 1.9253 6.6477 -4.9467
H -1.2882 5.5941 -1.7684
H -1.9667 7.1934 -2.0902
H -3.5605 2.2093 -3.9691
H -3.5100 2.2205 -5.7223
H -1.1471 2.8422 -3.8893
H 0.2604 2.6115 -5.9214
H -1.1946 2.4438 -6.9186
H -0.8664 3.9631 -6.0618
H -1.7497 0.2730 -5.4342
H -1.8744 0.4798 -3.6804
H -0.2980 0.5960 -4.4726
H -3.0184 4.6742 -5.7262
C -4.5985 -2.8862 3.7416
C -3.1950 -2.2655 3.8232
O -3.0325 -1.0493 3.8926
C -5.5780 -2.0201 2.9360
C -5.3429 -2.0981 1.4408
N -6.3535 -2.5770 0.6890
O -4.2734 -1.7273 0.9260
N -2.1491 -3.1335 3.9283
C -0.7976 -2.6444 4.1675
C -0.1108 -2.2057 2.8662
O -0.5054 -2.5963 1.7668
C -0.0787 -3.8974 4.7098
C -0.7171 -5.0230 3.8813
C -2.1874 -4.5904 3.7393
N 1.0000 -1.4562 3.0502
C 1.9544 -1.2131 1.9949
C 3.3800 -1.4247 2.4874
O 3.6439 -2.1181 3.4748
N 4.3295 -0.7908 1.7622
C 5.7583 -0.9450 2.0168
C 6.2809 -0.0036 3.1345
O 7.2147 0.7778 2.9327
C 6.5293 -0.7619 0.7230
O 6.1574 0.4932 0.1419
N 5.6489 -0.1601 4.3185
C 6.0270 0.5395 5.5410
H -6.6060 -2.3127 3.1651
H -5.4303 -0.9719 3.2101
H -4.5726 -3.9046 3.3466
H -0.3059 -4.0144 5.7743
H 1.0051 -3.8370 4.5916
H -0.6194 -6.0070 4.3455
H -0.2520 -5.0597 2.8932
H -2.8196 -5.0626 4.5013
H -2.5851 -4.8459 2.7520
H -0.8190 -1.8133 4.8766
H 1.8661 -0.2047 1.5843
H 1.7468 -1.9109 1.1817
H 7.6005 -0.7936 0.9346
H 6.2833 -1.5829 0.0422
H 6.5748 0.5157 -0.7312
H 5.9129 -1.9714 2.3702
H 6.8203 1.2447 5.2949
H 2.3151 7.7018 -3.5733
H -4.4829 4.3048 -4.8128
H -2.6994 4.2965 -2.8235
H -0.0174 7.8080 -3.2601
H 6.3994 -0.1705 6.2876
H 5.1703 1.0755 5.9580
H 4.9318 -0.8803 4.3664
H 1.2731 -1.1701 3.9876
H -7.1984 -2.9964 1.0826
H -6.2068 -2.6233 -0.3120
H 4.0741 -0.1955 0.9840
H -4.9601 -2.9617 4.7744
C -10.5536 -4.9473 2.5248
C -9.6585 -4.3256 1.4771
O -8.4914 -4.0195 1.7487
C -10.7880 -3.9045 3.6219
N -10.2212 -4.0558 0.2811
C -9.5047 -3.3382 -0.7570
C -8.5198 7.2456 2.8239
C -8.4631 5.9503 2.0205
C -8.4166 6.1664 0.5095
N -8.0352 5.0557 -0.1956
O -8.7396 7.2177 -0.0290
H -9.8309 -3.5741 4.0308
H -11.3911 -4.3174 4.4350
H -11.3103 -3.0286 3.2243
H -11.5021 -5.2894 2.0967
H -8.6072 -3.8869 -1.0600
H -7.6063 5.3366 2.3090
H -9.3523 5.3396 2.2272
H -7.5611 4.2932 0.2739
H -7.8847 5.1777 -1.1859
H -9.3580 7.8652 2.4969
H -8.6303 7.0333 3.8915
H -7.6077 7.8331 2.6840
H -10.0370 -5.8199 2.9352
H -9.1988 -2.3452 -0.4130
H -10.1541 -3.2245 -1.6251
H -11.1823 -4.3157 0.1262

YIH^+

C 11.4890 5.2541 -2.0447
C 10.3850 4.1899 -2.2237
C 9.9525 3.4694 -0.9707
C 8.6716 3.1856 -0.5365
N 10.8683 2.9033 -0.1001
C 10.1522 2.3038 0.8339
N 8.8135 2.4460 0.6240
H 11.1460 6.0734 -1.4053
H 10.7473 3.4422 -2.9428
H 9.5005 4.6465 -2.6842
H 7.7010 3.4469 -0.9274
H 10.5435 1.7593 1.6816
H 8.0799 2.0024 1.1718
C -9.5597 1.7591 -4.3593
C -8.4680 2.5286 -3.5950
C -7.3853 1.6188 -3.0604
C -6.3655 1.1395 -3.8959
C -7.3839 1.1899 -1.7284
C -5.3937 0.2607 -3.4272
C -6.4187 0.3092 -1.2390
C -5.4143 -0.1656 -2.0921
O -4.4392 -1.0266 -1.7013
H -10.0415 1.0243 -3.7061
H -8.9255 3.0798 -2.7646
H -8.0267 3.2794 -4.2627
H -6.3300 1.4627 -4.9338
H -8.1380 1.5706 -1.0440
H -4.6044 -0.1032 -4.0765
H -6.4222 0.0430 -0.1894
H -4.4608 -1.2483 -0.7385
C -0.3650 -7.0731 -4.8707
C -0.3655 -6.7880 -3.3651
C -1.5928 -6.0931 -2.8079
C -1.7705 -6.0323 -1.4188
C -2.5580 -5.4743 -3.6118
C -2.8547 -5.3752 -0.8489
C -3.6633 -4.8281 -3.0574
C -3.8140 -4.7765 -1.6717
O -4.9198 -4.1590 -1.1634
H -0.3915 -6.1482 -5.4559
H -0.2181 -7.7271 -2.8173
H 0.5164 -6.1732 -3.1309
H -1.0416 -6.5070 -0.7678
H -2.4581 -5.4958 -4.6919
H -2.9610 -5.3272 0.2318
H -4.4034 -4.3419 -3.6830
H -4.7583 -3.9645 -0.2289
C 4.8083 -4.6972 -5.8062
C 4.9409 -4.5953 -4.3012
O 5.7575 -3.8511 -3.7659
C 5.0711 -3.3600 -6.4995

H	5.5481	-5.4398	-6.1343
H	6.0345	-2.9592	-6.1792
H	4.3022	-2.6267	-6.2378
H	5.0774	-3.4770	-7.5863
N	4.1176	-5.4194	-3.5979
C	4.3782	-5.7267	-2.2057
C	3.1514	-5.6240	-1.3053
C	2.5549	-4.2178	-1.1790
C	1.3226	-4.2652	-0.2773
C	3.5814	-3.1975	-0.6720
H	3.5282	-6.0505	-4.1215
H	5.1533	-5.0391	-1.8794
H	3.4446	-5.9808	-0.3085
H	2.3740	-6.3165	-1.6581
H	2.2351	-3.8979	-2.1769
H	1.5904	-4.5929	0.7350
H	0.8412	-3.2937	-0.1859
H	0.5703	-4.9575	-0.6651
H	4.4284	-3.0898	-1.3557
H	3.9665	-3.4870	0.3137
H	3.1243	-2.2100	-0.5809
C	9.3778	-5.4353	-1.2745
C	8.2181	-4.4400	-1.1040
C	8.6209	-2.9785	-1.1936
C	8.0279	-2.1341	-2.1421
C	9.5839	-2.4345	-0.3300
C	8.3952	-0.7870	-2.2284
C	9.9538	-1.0924	-0.4183
C	9.3623	-0.2584	-1.3714
H	10.1452	-5.2953	-0.5049
H	7.7366	-4.6212	-0.1328
H	7.4646	-4.6378	-1.8703
H	7.2778	-2.5416	-2.8115
H	10.0516	-3.0688	0.4189
H	7.9385	-0.1555	-2.9869
H	10.7040	-0.6878	0.2537
H	9.6600	0.7819	-1.4440
C	-2.8078	0.8486	0.3303
C	-1.5743	1.0431	-0.2333
C	-1.4022	2.2526	-0.9816
N	-0.2029	2.6551	-1.4122
N	-2.4694	3.0564	-1.2423
C	-3.6474	2.7654	-0.7060
N	-3.8279	1.7119	0.1152
S	2.0079	-0.5839	-2.8383
C	1.4423	0.2304	-1.4265
C	-4.8313	3.6092	-1.0371
N	0.2955	-0.3939	-1.0831
C	-0.1756	-1.4715	-1.8651
C	-1.4504	-2.1697	-1.5274
C	0.6682	-1.7223	-2.9021
C	0.5331	-2.7447	-3.9868
C	-0.4466	0.1056	0.0904
H	0.6034	2.0209	-1.3572
H	-0.0960	3.5093	-1.9628
H	-0.8131	-0.7437	0.6662
H	0.2920	0.6307	0.6945
H	-5.4050	3.1165	-1.8301
H	-5.4804	3.6938	-0.1623
H	-4.5133	4.5906	-1.3881
H	-3.0298	0.0069	0.9806
H	-1.4726	-2.4889	-0.4820
H	-1.5642	-3.0574	-2.1476
H	-2.3178	-1.5299	-1.7095
H	-0.2062	-3.5017	-3.7127
H	1.4832	-3.2536	-4.1802
C	2.4672	-2.1979	7.1370
C	1.8100	-0.8543	7.4721
C	2.3147	0.2464	6.5647
O	2.0489	-0.0166	5.2787
O	2.8945	1.2475	6.9398
C	-7.9093	3.0099	3.9671
C	-7.1437	1.7996	3.4364
C	-6.4146	2.0629	2.1197
O	-6.5578	3.1555	1.5383
O	-5.6615	1.1025	1.7094
H	-8.3783	2.7838	4.9307
H	-7.2420	3.8634	4.1049
H	-7.8135	0.9448	3.2765
H	-6.3935	1.4553	4.1572
C	-0.3362	2.3857	4.9795
C	-0.2529	1.8247	3.5534
C	1.0437	2.1315	2.8681
C	1.2710	2.7466	1.6608
N	2.2679	1.7636	3.4127
C	3.2047	2.1347	2.5577
N	2.6412	2.7275	1.4754
H	0.5024	2.0498	5.5908
H	-0.3991	0.7391	3.5812
H	-1.0725	2.2208	2.9445
H	0.6058	3.1694	0.9263
H	4.2673	1.9986	2.6774
H	3.1375	3.0589	0.6623
C	5.7330	7.3305	1.6995
C	5.7940	6.3246	2.8488
C	6.4013	4.9574	2.4887
C	6.3517	4.0274	3.7060
C	5.6893	4.3293	1.2818
H	5.0631	6.9957	0.9015
H	4.7795	6.1586	3.2389
H	6.3696	6.7541	3.6784
H	7.4552	5.1061	2.2137
H	5.3139	3.8480	4.0152
H	6.8119	3.0601	3.4913
H	6.8708	4.4730	4.5617
H	4.6057	4.3874	1.4304
H	5.9621	3.2768	1.1489
H	5.9225	4.8570	0.3526
H	0.2070	-2.2811	-4.9248
H	-8.6917	3.3116	3.2694
H	12.3684	4.8078	-1.5736
H	11.7802	5.6695	-3.0142
H	-10.3306	2.4367	-4.7406
H	-9.1304	1.2169	-5.2083
H	-0.3183	3.4798	4.9696
H	-1.2653	2.0628	5.4575
H	6.7235	7.4857	1.2562
H	5.3687	8.3031	2.0458
H	0.7236	-0.9268	7.3393
H	2.0077	-0.5573	8.5038
H	2.3399	-2.4481	6.0816
H	2.0415	-3.0024	7.7437
H	3.5429	-2.1627	7.3370
H	9.0198	-6.4664	-1.2053
H	9.8598	-5.3042	-2.2485
H	4.7977	-6.7405	-2.1335
H	0.5391	-7.6190	-5.1562
H	-1.2295	-7.6777	-5.1628
H	3.8248	-5.0962	-6.0810
H	2.2921	0.7684	4.6570
H	-4.7678	1.5204	0.7495
C	2.0953	6.6525	-3.8777
C	0.9188	6.0466	-3.1457
O	0.8553	4.8406	-2.8977
N	-0.0676	6.9227	-2.7943
C	-1.3975	6.3979	-2.5420
C	-1.9998	5.8723	-3.8565
O	-1.7139	6.4104	-4.9243
N	-2.8483	4.8327	-3.7049
C	-3.4188	4.1321	-4.8487
C	-3.0437	2.6459	-4.8289
C	-1.5365	2.3571	-4.7695
C	-1.2945	0.8615	-4.5406
C	-0.7904	2.8725	-6.0046
H	2.9823	6.0419	-3.7085
H	1.8685	6.6464	-4.9494
H	-1.3509	5.6201	-1.7780
H	-2.0258	7.2068	-2.1586
H	-3.5286	2.1770	-3.9651
H	-3.4795	2.1681	-5.7172
H	-1.1295	2.8892	-3.9039
H	0.2764	2.6354	-5.9359
H	-1.1786	2.4105	-6.9212
H	-0.8808	3.9580	-6.1033
H	-1.6745	0.2662	-5.3805
H	-1.7986	0.5114	-3.6332
H	-0.2272	0.6472	-4.4332
H	-3.0547	4.6373	-5.7444
C	-4.5993	-2.9439	3.7141
C	-3.1884	-2.3357	3.7844
O	-3.0101	-1.1204	3.8346
C	-5.5739	-2.0477	2.9319
C	-5.3714	-2.1027	1.4327
N	-6.4273	-2.4804	0.6903
O	-4.2802	-1.8158	0.8983
N	-2.1464	-3.2083	3.8957
C	-0.7949	-2.7179	4.1335
C	-0.1163	-2.2507	2.8376
O	-0.5114	-2.6230	1.7316
C	-0.0678	-3.9749	4.6569
C	-0.7177	-5.0961	3.8320
C	-2.1911	-4.6667	3.7237
N	0.9895	-1.4949	3.0316
C	1.9393	-1.2179	1.9808
C	3.3695	-1.4149	2.4669
O	3.6468	-2.1188	3.4429
N	4.3063	-0.7605	1.7433
C	5.7387	-0.8990	1.9876
C	6.2510	0.0420	3.1100
O	7.1565	0.8580	2.9090
C	6.4996	-0.7007	0.6902
O	6.0954	0.5416	0.1089
N	5.6417	-0.1546	4.2991
C	6.0062	0.5338	5.5322
H	-6.6040	-2.3215	3.1724
H	-5.4081	-1.0010	3.2067
H	-4.5878	-3.9570	3.3039
H	-0.2800	-4.0988	5.7237
H	1.0143	-3.9126	4.5248
H	-0.6073	-6.0836	4.2857
H	-0.2723	-5.1227	2.8342
H	-2.8035	-5.1311	4.5066
H	-2.6160	-4.9341	2.7511
H	-0.8153	-1.8972	4.8549
H	1.8397	-0.2017	1.5912
H	1.7408	-1.9017	1.1548
H	7.5716	-0.7089	0.8999
H	6.2723	-1.5315	0.0138
H	6.5675	0.6074	-0.7332
H	5.9077	-1.9253	2.3340
H	6.7874	1.2565	5.2991
H	2.2881	7.6841	-3.5722
H	-4.5117	4.2297	-4.8329
H	-2.7928	4.3194	-2.8255
H	-0.0567	7.7998	-3.2979
H	6.3902	-0.1817	6.2675
H	5.1401	1.0496	5.9552
H	4.9359	-0.8870	4.3370
H	1.2572	-1.2162	3.9725
H	-7.2600	-2.9161	1.0899
H	-6.3049	-2.5056	-0.3138
H	4.0384	-0.1626	0.9711
H	-4.9499	-3.0291	4.7495
C	-10.5222	-5.0338	2.4918
C	-9.6420	-4.3899	1.4462
O	-8.4902	-4.0380	1.7249
C	-10.7833	-3.9938	3.5848
N	-10.2016	-4.1516	0.2414
C	-9.4971	-3.4151	-0.7916
C	-8.5712	7.1689	2.8045
C	-8.5356	5.8566	2.0288
C	-8.5214	6.0341	0.5124
N	-8.1475	4.9049	-0.1647
O	-8.8504	7.0728	-0.0488
H	-9.8351	-3.6380	3.9930
H	-11.3765	-4.4187	4.3992
H	-11.3266	-3.1322	3.1843
H	-11.4605	-5.4014	2.0624
H	-8.5603	-3.9172	-1.0516
H	-7.6761	5.2443	2.3109
H	-9.4223	5.2550	2.2689

H	-7.6649	4.1564	0.3267
H	-8.0195	4.9963	-1.1613
H	-9.4084	7.7893	2.4756
H	-8.6695	6.9794	3.8776
H	-7.6558	7.7459	2.6419
H	-9.9837	-5.8915	2.9056
H	-9.2618	-2.3976	-0.4629
H	-10.1238	-3.3605	-1.6820
H	-11.1534	-4.4398	0.0796

TC

C	11.4701	5.0422	-2.3640
C	10.3633	3.9817	-2.5414
C	9.8771	3.3168	-1.2774
C	8.5792	3.0275	-0.8992
N	10.7539	2.8078	-0.3345
C	9.9992	2.2344	0.5855
N	8.6723	2.3356	0.2944
H	11.1114	5.8901	-1.7718
H	10.7453	3.2016	-3.2146
H	9.5013	4.4253	-3.0542
H	7.6265	3.2472	-1.3558
H	10.3529	1.7361	1.4768
H	7.9139	1.9105	0.8228
C	-9.6115	1.4868	-4.2485
C	-8.1883	2.0394	-4.0592
C	-7.2556	1.0622	-3.3783
C	-6.6923	-0.0088	-4.0863
C	-6.9509	1.1742	-2.0176
C	-5.8520	-0.9262	-3.4655
C	-6.1074	0.2631	-1.3785
C	-5.5422	-0.7892	-2.1057
O	-4.6903	-1.6995	-1.5587
H	-10.0605	1.2392	-3.2812
H	-8.2357	2.9637	-3.4716
H	-7.7794	2.3164	-5.0391
H	-6.9067	-0.1193	-5.1468
H	-7.3712	1.9831	-1.4293
H	-5.3983	-1.7385	-4.0234
H	-5.8788	0.3768	-0.3257
H	-4.4943	-1.5007	-0.6063
C	-0.4306	-7.3693	-4.5537
C	-0.1658	-6.0037	-3.9048
C	-1.3424	-5.5567	-3.0713
C	-1.4139	-5.8838	-1.7133
C	-2.4387	-4.8978	-3.6451
C	-2.5456	-5.5928	-0.9549
C	-3.5781	-4.6006	-2.9034
C	-3.6406	-4.9708	-1.5577
O	-4.8073	-4.7353	-0.8855
H	-1.3110	-7.3235	-5.2019
H	0.7313	-6.0575	-3.2758
H	0.0449	-5.2620	-4.6852
H	-0.5749	-6.3856	-1.2392
H	-2.4015	-4.6147	-4.6940
H	-2.5815	-5.8568	0.0994
H	-4.4201	-4.0769	-3.3391
H	-4.6737	-4.8671	0.0626
C	4.7306	-5.0366	-5.6513
C	5.0252	-5.0274	-4.1758
O	5.9764	-4.4060	-3.7015
C	4.8243	-3.6300	-6.2512
H	5.4763	-5.6875	-6.1250
H	5.7943	-3.1903	-6.0112
H	4.0478	-2.9775	-5.8389
H	4.7056	-3.6576	-7.3375
N	4.1751	-5.7710	-3.4290
C	4.3506	-5.9285	-2.0084
C	3.1406	-5.4894	-1.1898
C	2.7047	-4.0324	-1.3878
C	1.4923	-3.7562	-0.5034
C	3.8314	-3.0302	-1.1030
H	3.4117	-6.2453	-3.8864
H	5.2298	-5.3421	-1.7435
H	3.3791	-5.6521	-0.1299
H	2.2887	-6.1491	-1.4104
H	2.4022	-3.9060	-2.4369
H	1.7215	-3.9523	0.5496
H	1.1750	-2.7190	-0.5932
H	0.6378	-4.3820	-0.7725
H	4.6871	-3.1667	-1.7703
H	4.1856	-3.1301	-0.0692
H	3.4677	-2.0051	-1.2333
C	9.3629	-5.6078	-1.1594
C	8.2032	-4.6088	-1.0010
C	8.6031	-3.1556	-1.1731
C	8.0192	-2.3705	-2.1768
C	9.5556	-2.5600	-0.3327
C	8.3810	-1.0281	-2.3333
C	9.9227	-1.2242	-0.4934
C	9.3364	-0.4479	-1.4968
H	10.1559	-5.4256	-0.4271
H	7.7543	-4.7429	-0.0065
H	7.4267	-4.8405	-1.7349
H	7.2906	-2.8216	-2.8423
H	10.0169	-3.1508	0.4550
H	7.9329	-0.4423	-3.1326
H	10.6672	-0.7798	0.1598
H	9.6309	0.5876	-1.6254
C	-3.0937	1.2412	0.7504
C	-1.8657	1.1475	0.1457
C	-1.5602	2.1252	-0.8360
N	-0.3434	2.1404	-1.4348
N	-2.4766	3.0505	-1.1916
C	-3.6616	3.0424	-0.5617
N	-3.9976	2.1907	0.4143
S	1.6315	0.5969	-2.5991
C	0.6797	1.1197	-1.1208
C	-4.6975	4.0267	-1.0183
N	0.0764	-0.0981	-0.6338
C	-0.2178	-1.0046	-1.6890
C	-1.2803	-2.0339	-1.4784
C	0.5580	-0.8369	-2.7764
C	0.6850	-1.6805	-4.0013
C	-0.8408	0.1058	0.4813
H	1.3778	1.5248	-0.3841
H	-0.0811	2.9299	-2.0255
H	-1.3044	-0.8389	0.7592
H	-0.2484	0.4209	1.3436
H	-5.2266	3.6089	-1.8828
H	-5.4408	4.1980	-0.2424
H	-4.2378	4.9662	-1.3277
H	-3.3907	0.5290	1.5152
H	-1.1525	-2.5503	-0.5242
H	-1.2600	-2.7806	-2.2703
H	-2.2756	-1.5792	-1.4885
H	0.0191	-2.5450	-3.9408
H	1.7106	-2.0568	-4.1166
C	2.5735	-2.0457	7.2196
C	1.9084	-0.6957	7.5129
C	2.4009	0.3674	6.5561
O	2.0789	0.0746	5.2897
O	3.0259	1.3601	6.8778
C	-7.8433	3.0504	4.0004
C	-8.2462	2.2471	2.7536
C	-7.2048	2.3915	1.6653
O	-7.3993	3.0213	0.6289
O	-6.0606	1.8088	1.9669
H	-8.5952	2.9419	4.7872
H	-6.8838	2.6979	4.3864
H	-9.2034	2.5895	2.3545
H	-8.3345	1.1862	3.0122
C	-0.2571	2.4560	4.9294
C	-0.1640	1.9583	3.4818
C	1.1519	2.2755	2.8399
C	1.4370	3.0161	1.7175
N	2.3458	1.8075	3.3737
C	3.3222	2.2483	2.6029
N	2.8160	2.9814	1.5789
H	0.5602	2.0694	5.5374
H	-0.3252	0.8745	3.4678
H	-0.9672	2.3937	2.8786
H	0.8046	3.5464	1.0229
H	4.3761	2.0669	2.7394
H	3.3654	3.4648	0.8837
C	5.7686	7.2660	1.3793
C	6.6616	6.5491	2.3951
C	7.0818	5.1209	2.0034
C	7.8970	4.4792	3.1325
C	5.8645	4.2551	1.6613
H	4.7911	6.7833	1.2838
H	6.1412	6.5047	3.3624
H	7.5677	7.1439	2.5660
H	7.7187	5.1781	1.1095
H	7.2980	4.4146	4.0494
H	8.2109	3.4658	2.8741
H	8.7903	5.0712	3.3582
H	5.1445	4.2906	2.4866
H	6.1461	3.2106	1.5040
H	5.3625	4.6169	0.7557
H	0.4370	-1.1257	-4.9140
H	-7.7496	4.1144	3.7689
H	12.3263	4.6086	-1.8414
H	11.8020	5.4152	-3.3374
H	-10.2573	2.2152	-4.7498
H	-9.5968	0.5726	-4.8511
H	-0.2123	3.5486	4.9709
H	-1.2020	2.1344	5.3769
H	6.2309	7.2802	0.3852
H	5.5910	8.3043	1.6763
H	0.8217	-0.7816	7.3942
H	2.1175	-0.3589	8.5301
H	2.4382	-2.3304	6.1738
H	2.1587	-2.8326	7.8561
H	3.6502	-1.9948	7.4103
H	9.0102	-6.6351	-1.0237
H	9.8069	-5.5278	-2.1563
H	4.5853	-6.9774	-1.7796
H	0.4201	-7.7005	-5.1601
H	-0.6226	-8.1292	-3.7899
H	3.7491	-5.4801	-5.8548
H	2.3367	0.8232	4.6335
H	-5.3260	2.0407	1.2724
C	2.0523	6.3812	-4.1166
C	0.9569	5.7946	-3.2389
O	1.0247	4.6649	-2.7496
N	-0.1069	6.6307	-3.0480
C	-1.3933	6.0843	-2.6503
C	-2.1305	5.6184	-3.9140
O	-2.0947	6.3062	-4.9352
N	-2.7659	4.4391	-3.7993
C	-3.4766	3.8323	-4.9138
C	-3.6354	2.3253	-4.7057
C	-2.3215	1.5397	-4.5443
C	-2.6264	0.0565	-4.3217
C	-1.3669	1.7395	-5.7257
H	3.0074	5.9198	-3.8636
H	1.8227	6.1475	-5.1618
H	-1.2370	5.2760	-1.9372
H	-1.9815	6.8693	-2.1644
H	-4.2683	2.1409	-3.8279
H	-4.1925	1.9235	-5.5620
H	-1.8096	1.9053	-3.6473
H	-0.4506	1.1588	-5.5785
H	-1.8289	1.4118	-6.6655
H	-1.0724	2.7868	-5.8411
H	-3.1603	-0.3665	-5.1816
H	-3.2551	-0.0921	-3.4413
H	-1.7077	-0.5120	-4.1738
H	-2.9261	4.0587	-5.8310
C	-4.5409	-2.9126	3.9269
C	-3.1376	-2.2814	3.9571
O	-2.9763	-1.0622	3.9486
C	-5.5375	-2.0529	3.1219
C	-5.2814	-2.1268	1.6245
N	-6.1097	-2.9075	0.9072
O	-4.3305	-1.5190	1.0974
N	-2.0832	-3.1360	4.0840
C	-0.7302	-2.6228	4.2685
C	-0.0664	-2.2567	2.9312

O -0.4324 -2.7590 1.8703
C 0.0095 -3.8302 4.8806
C -0.6460 -5.0178 4.1594
C -2.1222 -4.6036 4.0317
N 0.9855 -1.4107 3.0603
C 1.9236 -1.1500 1.9938
C 3.3602 -1.3564 2.4643
O 3.6396 -2.0156 3.4704
N 4.3044 -0.7566 1.7001
C 5.7342 -0.8917 1.9639
C 6.2218 0.1198 3.0331
O 6.9943 1.0456 2.7608
C 6.5177 -0.7853 0.6665
O 6.1093 0.3936 -0.0311
N 5.7342 -0.1365 4.2672
C 6.0912 0.6190 5.4627
H -6.5566 -2.3864 3.3362
H -5.4247 -1.0080 3.4200
H -4.5198 -3.9331 3.5367
H -0.1834 -3.8680 5.9576
H 1.0890 -3.7747 4.7253
H -0.5272 -5.9642 4.6919
H -0.2121 -5.1234 3.1623
H -2.7255 -5.0048 4.8558
H -2.5545 -4.9520 3.0884
H -0.7512 -1.7489 4.9240
H 1.8201 -0.1315 1.6082
H 1.6980 -1.8238 1.1675
H 7.5865 -0.7583 0.8961
H 6.3211 -1.6747 0.0589
H 6.5376 0.3491 -0.8982
H 5.8894 -1.8950 2.3753
H 6.8005 1.3935 5.1694
H 2.1243 7.4666 -4.0136
H -4.4665 4.2952 -5.0243
H -2.6584 3.9168 -2.9279
H -0.1952 7.3792 -3.7251
H 6.5641 -0.0389 6.1991
H 5.2069 1.0811 5.9102
H 5.0661 -0.9007 4.3438
H 1.2522 -1.0893 3.9876
H -6.9382 -3.3525 1.3039
H -5.9257 -2.9941 -0.0873
H 4.0508 -0.1929 0.8991
H -4.8826 -2.9788 4.9668
C -10.4322 -5.0490 2.8871
C -9.5629 -4.4586 1.7964
O -8.3885 -4.1488 2.0254
C -10.7269 -3.9594 3.9244
N -10.1688 -4.2219 0.6125
C -9.5020 -3.5485 -0.4884
C -8.5188 7.1631 2.6904
C -8.0361 6.0928 1.7179
C -8.0018 6.5856 0.2705
N -7.9814 5.5871 -0.6566
O -7.9843 7.7745 -0.0246
H -9.7908 -3.5538 4.3153
H -11.3077 -4.3602 4.7594
H -11.2954 -3.1371 3.4789
H -11.3620 -5.4630 2.4817
H -8.7850 -4.2069 -0.9910
H -7.0166 5.7732 1.9732
H -8.6525 5.1903 1.7733
H -7.9026 4.6171 -0.3764
H -7.8549 5.8486 -1.6221
H -9.5620 7.4279 2.4947
H -8.4417 6.8157 3.7254
H -7.9264 8.0733 2.5764
H -9.8746 -5.8688 3.3489
H -8.9605 -2.6741 -0.1198
H -10.2487 -3.2222 -1.2131
H -11.1215 -4.5268 0.4907

TCH$^+$

C 11.4927 5.0532 -2.3524
C 10.3895 3.9902 -2.5373
C 9.8938 3.3257 -1.2767
C 8.5933 3.0330 -0.9101
N 10.7633 2.8200 -0.3252
C 10.0019 2.2452 0.5884
N 8.6774 2.3423 0.2849
H 11.1276 5.9012 -1.7642
H 10.7785 3.2099 -3.2062
H 9.5309 4.4314 -3.0579
H 7.6443 3.2492 -1.3760
H 10.3489 1.7483 1.4831
H 7.9156 1.9138 0.8056
C -9.5891 1.5128 -4.2630
C -8.2081 2.1053 -3.9333
C -7.2682 1.1046 -3.2974
C -6.6595 0.1020 -4.0656
C -7.0042 1.1239 -1.9237
C -5.8107 -0.8369 -3.4900
C -6.1534 0.1887 -1.3293
C -5.5384 -0.7903 -2.1162
O -4.6717 -1.7153 -1.6159
H -10.0783 1.1483 -3.3541
H -8.3334 2.9605 -3.2593
H -7.7599 2.4990 -4.8547
H -6.8450 0.0618 -5.1365
H -7.4535 1.8787 -1.2857
H -5.3246 -1.5970 -4.0923
H -5.9707 0.2301 -0.2615
H -4.4901 -1.5715 -0.6519
C -0.4118 -7.3463 -4.5886
C -0.1469 -5.9829 -3.9353
C -1.3239 -5.5378 -3.1012
C -1.4108 -5.9004 -1.7531
C -2.4072 -4.8505 -3.6664
C -2.5437 -5.6123 -0.9956
C -3.5482 -4.5565 -2.9259
C -3.6247 -4.9584 -1.5900
O -4.7911 -4.7204 -0.9173
H -1.2919 -7.2980 -5.2369
H 0.7502 -6.0382 -3.3063
H 0.0638 -5.2390 -4.7136
H -0.5843 -6.4302 -1.2874
H -2.3583 -4.5422 -4.7077
H -2.5912 -5.9028 0.0512
H -4.3788 -4.0081 -3.3527
H -4.6648 -4.8936 0.0250
C 4.7512 -5.0125 -5.6752
C 5.0290 -4.9892 -4.1968
O 5.9552 -4.3365 -3.7151
C 4.7871 -3.6034 -6.2758
H 5.5314 -5.6286 -6.1399
H 5.7341 -3.1200 -6.0276
H 3.9780 -2.9863 -5.8716
H 4.6799 -3.6371 -7.3630
N 4.1937 -5.7552 -3.4560
C 4.3682 -5.9154 -2.0354
C 3.1482 -5.5041 -1.2173
C 2.6929 -4.0513 -1.4018
C 1.4769 -3.7979 -0.5156
C 3.8064 -3.0370 -1.1066
H 3.4553 -6.2608 -3.9210
H 5.2340 -5.3117 -1.7651
H 3.3850 -5.6742 -0.1582
H 2.3070 -6.1737 -1.4483
H 2.3901 -3.9195 -2.4500
H 1.7070 -4.0017 0.5356
H 1.1494 -2.7630 -0.5949
H 0.6281 -4.4286 -0.7910
H 4.6607 -3.1504 -1.7796
H 4.1669 -3.1466 -0.0759
H 3.4267 -2.0156 -1.2192
C 9.3800 -5.5995 -1.1818
C 8.2204 -4.6004 -1.0233
C 8.6229 -3.1468 -1.1854
C 8.0418 -2.3541 -2.1846
C 9.5759 -2.5588 -0.3401
C 8.4076 -1.0118 -2.3329
C 9.9465 -1.2228 -0.4922
C 9.3637 -0.4392 -1.4919
H 10.1691 -5.4224 -0.4442
H 7.7668 -4.7397 -0.0317
H 7.4464 -4.8271 -1.7615
H 7.3120 -2.7987 -2.8530
H 10.0351 -3.1554 0.4443
H 7.9628 -0.4202 -3.1296
H 10.6913 -0.7844 0.1647
H 9.6616 0.5959 -1.6148
C -3.1860 1.1671 0.7887
C -1.9560 1.1065 0.1974
C -1.6581 2.1019 -0.7782
N -0.4376 2.1473 -1.3512
N -2.5898 3.0120 -1.1466
C -3.7820 2.9876 -0.5486
N -4.0887 2.1114 0.4229
S 1.5865 0.6577 -2.5033
C 0.6051 1.1357 -1.0344
C -4.8311 3.9583 -0.9896
N 0.0143 -0.0940 -0.5738
C -0.2352 -1.0022 -1.6396
C -1.2774 -2.0563 -1.4574
C 0.5565 -0.8045 -2.7102
C 0.7325 -1.6341 -3.9387
C -0.9169 0.0763 0.5328
H 1.2757 1.5537 -0.2806
H -0.1911 2.9207 -1.9695
H -1.3728 -0.8777 0.7905
H -0.3416 0.3876 1.4074
H -5.0985 3.7450 -2.0294
H -5.7314 3.8666 -0.3811
H -4.4389 4.9780 -0.9555
H -3.5034 0.4585 1.5473
H -1.1664 -2.5673 -0.4982
H -1.2136 -2.8046 -2.2452
H -2.2828 -1.6264 -1.5020
H 0.0899 -2.5171 -3.8986
H 1.7704 -1.9804 -4.0341
C 2.5860 -2.0600 7.2031
C 1.9087 -0.7149 7.4919
C 2.4031 0.3488 6.5372
O 2.0764 0.0597 5.2707
O 3.0363 1.3365 6.8579
C -7.8262 3.0505 3.9920
C -8.2387 2.2321 2.7592
C -7.1946 2.3466 1.6526
O -7.4427 3.0353 0.6379
O -6.0860 1.7365 1.8685
H -8.5600 2.9453 4.7969
H -6.8556 2.7080 4.3612
H -9.2003 2.5745 2.3682
H -8.3332 1.1766 3.0390
C -0.2410 2.4498 4.9247
C -0.1460 1.9581 3.4756
C 1.1694 2.2812 2.8357
C 1.4577 3.0593 1.7398
N 2.3605 1.7854 3.3491
C 3.3385 2.2454 2.5915
N 2.8359 3.0181 1.5956
H 0.5788 2.0652 5.5304
H -0.3042 0.8742 3.4596
H -0.9508 2.3950 2.8753
H 0.8289 3.6263 1.0716
H 4.3914 2.0513 2.7187
H 3.3885 3.5215 0.9173
C 5.7894 7.2680 1.3936
C 6.6924 6.5541 2.4028
C 7.1076 5.1243 2.0115
C 7.9324 4.4841 3.1344
C 5.8869 4.2584 1.6829
H 4.8099 6.7869 1.3113
H 6.1820 6.5136 3.3756
H 7.6006 7.1489 2.5620
H 7.7362 5.1782 1.1115
H 7.3427 4.4244 4.0576
H 8.2404 3.4689 2.8757
H 8.8299 5.0739 3.3488

H	5.1747	4.2946	2.5152
H	6.1675	3.2139	1.5245
H	5.3772	4.6194	0.7810
H	0.4878	-1.0788	-4.8519
H	-7.7413	4.1139	3.7526
H	12.3455	4.6221	-1.8223
H	11.8315	5.4255	-3.3236
H	-10.2412	2.2593	-4.7279
H	-9.4953	0.6665	-4.9516
H	-0.2017	3.5425	4.9701
H	-1.1840	2.1223	5.3714
H	6.2406	7.2770	0.3943
H	5.6170	8.3080	1.6878
H	0.8237	-0.8085	7.3645
H	2.1082	-0.3766	8.5104
H	2.4647	-2.3427	6.1551
H	2.1692	-2.8513	7.8327
H	3.6600	-2.0021	7.4069
H	9.0258	-6.6273	-1.0542
H	9.8296	-5.5138	-2.1757
H	4.6238	-6.9604	-1.8114
H	0.4390	-7.6760	-5.1956
H	-0.6044	-8.1086	-3.8273
H	3.7934	-5.5006	-5.8894
H	2.3442	0.8028	4.6140
H	-5.0865	2.0483	0.9911
C	2.0767	6.4017	-4.1076
C	0.9863	5.7973	-3.2335
O	1.0351	4.6480	-2.7910
N	-0.0616	6.6439	-2.9875
C	-1.3522	6.0791	-2.6328
C	-2.0423	5.5903	-3.9158
O	-1.8967	6.2154	-4.9664
N	-2.7801	4.4726	-3.7809
C	-3.4527	3.8576	-4.9167
C	-3.5949	2.3472	-4.7258
C	-2.2756	1.5854	-4.5080
C	-2.5592	0.0913	-4.3359
C	-1.2560	1.8357	-5.6236
H	3.0113	5.8624	-3.9517
H	1.7787	6.2916	-5.1559
H	-1.2103	5.2773	-1.9090
H	-1.9683	6.8557	-2.1686
H	-4.2671	2.1419	-3.8822
H	-4.1029	1.9438	-5.6114
H	-1.8269	1.9415	-3.5745
H	-0.3389	1.2684	-5.4348
H	-1.6531	1.5250	-6.5981
H	-0.9756	2.8909	-5.6927
H	-3.0370	-0.3212	-5.2331
H	-3.2305	-0.0921	-3.4942
H	-1.6374	-0.4614	-4.1531
H	-2.8762	4.0999	-5.8128
C	-4.5264	-2.9138	3.9026
C	-3.1220	-2.2843	3.9075
O	-2.9507	-1.0685	3.8271
C	-5.5306	-2.0802	3.0804
C	-5.2792	-2.1771	1.5836
N	-6.1435	-2.9199	0.8719
O	-4.3039	-1.6129	1.0489
N	-2.0727	-3.1340	4.0952
C	-0.7213	-2.6130	4.2693
C	-0.0569	-2.2725	2.9260
O	-0.4211	-2.7918	1.8729
C	0.0224	-3.8013	4.9134
C	-0.6380	-5.0107	4.2355
C	-2.1155	-4.6022	4.1159
N	0.9932	-1.4215	3.0414
C	1.9340	-1.1822	1.9718
C	3.3698	-1.3696	2.4530
O	3.6478	-2.0196	3.4654
N	4.3146	-0.7695	1.6899
C	5.7438	-0.9026	1.9591
C	6.2280	0.1129	3.0261
O	6.9921	1.0448	2.7509
C	6.5320	-0.8024	0.6639
O	6.1268	0.3727	-0.0413
N	5.7478	-0.1476	4.2621
C	6.1062	0.6085	5.4569
H	-6.5459	-2.4200	3.3024
H	-5.4375	-1.0263	3.3549
H	-4.5050	-3.9453	3.5421
H	-0.1615	-3.8056	5.9926
H	1.1006	-3.7505	4.7482
H	-0.5101	-5.9411	4.7934
H	-0.2168	-5.1439	3.2361
H	-2.7031	-4.9620	4.9701
H	-2.5667	-4.9953	3.1996
H	-0.7446	-1.7237	4.9042
H	1.8229	-0.1765	1.5559
H	1.7175	-1.8820	1.1645
H	7.6000	-0.7744	0.8974
H	6.3377	-1.6950	0.0601
H	6.5636	0.3261	-0.9042
H	5.8975	-1.9043	2.3752
H	6.8123	1.3852	5.1618
H	2.2196	7.4653	-3.9008
H	-4.4455	4.3069	-5.0534
H	-2.7342	3.9694	-2.8956
H	-0.1446	7.4113	-3.6439
H	6.5830	-0.0485	6.1915
H	5.2217	1.0677	5.9071
H	5.0824	-0.9140	4.3399
H	1.2635	-1.0938	3.9655
H	-6.9738	-3.3561	1.2758
H	-5.9658	-3.0218	-0.1217
H	4.0631	-0.2132	0.8831
H	-4.8613	-2.9517	4.9462
C	-10.4691	-5.0421	2.8367
C	-9.5808	-4.4698	1.7520
O	-8.3944	-4.2079	1.9808
C	-10.7128	-3.9578	3.8925
N	-10.1782	-4.1971	0.5722
C	-9.4846	-3.5340	-0.5182
C	-8.4990	7.1674	2.6940
C	-7.8828	6.1819	1.7062
C	-8.1617	6.5616	0.2517
N	-8.2397	5.4976	-0.5921
O	-8.2847	7.7255	-0.1162
H	-9.7590	-3.5994	4.2862
H	-11.3083	-4.3462	4.7231
H	-11.2459	-3.1044	3.4621
H	-11.4172	-5.4092	2.4286
H	-8.7192	-4.1829	-0.9574
H	-6.7910	6.1569	1.8258
H	-8.2258	5.1608	1.8854
H	-8.1156	4.5475	-0.2528
H	-8.3709	5.6852	-1.5738
H	-9.5922	7.1302	2.6541
H	-8.1887	6.9439	3.7196
H	-8.1972	8.1874	2.4453
H	-9.9451	-5.8926	3.2821
H	-8.9959	-2.6224	-0.1640
H	-10.2066	-3.2692	-1.2908
H	-11.1461	-4.4500	0.4525

Crystallographic water
Model C

AP

C	11.4992	5.0827	-2.1138
C	10.3586	4.0471	-2.2260
C	10.0056	3.3124	-0.9564
C	8.7614	3.1269	-0.3840
N	10.9605	2.6449	-0.2080
C	10.3037	2.0831	0.7911
N	8.9664	2.3378	0.7354
H	11.2279	5.8935	-1.4305
H	10.6449	3.3065	-2.9857
H	9.4529	4.5354	-2.6053
H	7.7823	3.4923	-0.6475
H	10.7401	1.4887	1.5814
H	8.2657	1.9769	1.3822
C	-9.5789	1.7387	-4.3840
C	-8.5383	2.5447	-3.5861
C	-7.4597	1.6611	-3.0026
C	-6.4492	1.1339	-3.8204
C	-7.4611	1.2905	-1.6537
C	-5.4942	0.2561	-3.3193
C	-6.5102	0.4118	-1.1326
C	-5.5226	-0.1208	-1.9704
O	-4.5873	-1.0145	-1.5561
H	-10.0690	0.9999	-3.7418
H	-9.0381	3.0954	-2.7809
H	-8.0871	3.2970	-4.2453
H	-6.4111	1.4155	-4.8699
H	-8.2135	1.7082	-0.9890
H	-4.7146	-0.1499	-3.9550
H	-6.5222	0.1605	-0.0795
H	-4.5204	-1.0947	-0.5683
C	-0.4729	-7.1960	-4.6371
C	-0.4737	-6.8707	-3.1396
C	-1.7332	-6.2083	-2.6075
C	-1.9293	-6.0999	-1.2233
C	-2.7254	-5.6754	-3.4412
C	-3.0627	-5.4942	-0.6881
C	-3.8691	-5.0687	-2.9229
C	-4.0475	-4.9838	-1.5412
O	-5.2029	-4.4213	-1.0829
H	-0.5398	-6.2902	-5.2485
H	-0.2998	-7.7927	-2.5711
H	0.3880	-6.2248	-2.9180
H	-1.1815	-6.5029	-0.5461
H	-2.6177	-5.7365	-4.5189
H	-3.1841	-5.4190	0.3897
H	-4.6321	-4.6590	-3.5754
H	-5.1724	-4.3311	-0.1197
C	4.7261	-4.8981	-5.6236
C	4.6605	-4.6658	-4.1270
O	5.1593	-3.6773	-3.5921
C	4.6482	-3.5916	-6.4138
H	5.6855	-5.3952	-5.8189
H	5.4008	-2.8912	-6.0467
H	3.6677	-3.1192	-6.2943
H	4.8140	-3.7659	-7.4799
N	4.0508	-5.6598	-3.4226
C	4.2784	-5.8264	-1.9976
C	3.0460	-5.6494	-1.1128
C	2.4816	-4.2248	-1.0547
C	1.2232	-4.2121	-0.1828
C	3.5210	-3.2173	-0.5464
H	3.7241	-6.4620	-3.9407
H	5.0506	-5.1097	-1.7188
H	3.3237	-5.9616	-0.0968
H	2.2592	-6.3419	-1.4420
H	2.2023	-3.9402	-2.0780
H	1.4585	-4.5334	0.8385
H	0.7775	-3.2212	-0.1015
H	0.4526	-4.8806	-0.5785
H	4.3851	-3.1539	-1.2122
H	3.8618	-3.4851	0.4604
H	3.0942	-2.2133	-0.4850
C	9.2782	-5.5605	-1.0628
C	8.1134	-4.5599	-0.9587
C	8.5092	-3.1069	-1.1411
C	7.8913	-2.3140	-2.1168
C	9.4903	-2.5185	-0.3284
C	8.2472	-0.9703	-2.2744
C	9.8520	-1.1823	-0.4909
C	9.2305	-0.3963	-1.4666
H	10.0393	-5.3711	-0.3003
H	7.6294	-4.6811	0.0205
H	7.3544	-4.8073	-1.7081
H	7.1267	-2.7464	-2.7561
H	9.9768	-3.1132	0.4402
H	7.7607	-0.3764	-3.0437
H	10.6185	-0.7435	0.1388
H	9.5252	0.6406	-1.5940

C -2.5090 0.8338 0.3037
C -1.2950 1.0253 -0.3296
C -1.1891 2.1795 -1.1515
N -0.0251 2.5629 -1.7066
N -2.2840 2.9297 -1.4125
C -3.4301 2.6234 -0.7965
N -3.5742 1.6320 0.1005
S 1.8464 -1.2364 -3.2048
C 1.5309 -0.3016 -1.8293
C -4.6380 3.4383 -1.1402
N 0.3560 -0.5954 -1.2852
C -0.3568 -1.6151 -1.9335
C -1.6609 -2.1000 -1.4038
C 0.3224 -2.0711 -3.0295
C -0.0785 -3.1284 -4.0059
C -0.1358 0.1178 -0.0663
H 2.2574 0.3924 -1.3872
H 0.0439 3.4368 -2.2355
H -0.4018 -0.6505 0.6632
H 0.7181 0.6760 0.3210
H -5.2006 2.9353 -1.9342
H -5.2870 3.5252 -0.2702
H -4.3489 4.4270 -1.4975
H -2.6514 0.0161 1.0063
H -1.6164 -2.2520 -0.3218
H -1.9134 -3.0538 -1.8659
H -2.4707 -1.4018 -1.6274
H -0.8611 -3.7587 -3.5819
H 0.7700 -3.7697 -4.2634
C 2.3831 -2.0297 7.2426
C 1.6412 -0.6904 7.2453
C 2.3087 0.3021 6.2904
O 2.3434 -0.0838 5.0491
O 2.7759 1.3672 6.7073
C -7.9334 3.1954 3.9097
C -7.0569 2.0066 3.5304
C -6.2616 2.2395 2.2634
O -6.3310 3.2728 1.6078
O -5.4849 1.2135 1.9535
H -8.4755 2.9897 4.8374
H -7.3321 4.0943 4.0577
H -7.6507 1.0954 3.3857
H -6.3361 1.7639 4.3201
C -0.3692 2.5224 4.9566
C -0.3026 1.8797 3.5628
C 0.9599 2.2240 2.8416
C 1.2159 2.8947 1.6629
N 2.2009 1.9088 3.3563
C 3.1316 2.3754 2.4994
N 2.5828 2.9825 1.4497
H 0.4943 2.2451 5.5663
H -0.3942 0.7923 3.6630
H -1.1530 2.2021 2.9524
H 0.5075 3.3373 0.9773
H 4.1892 2.2346 2.6577
H 0.8438 2.2017 -1.3495
C 5.7566 7.3164 1.5598
C 5.6908 6.3223 2.7206
C 6.3716 4.9678 2.4547
C 6.2830 4.0826 3.7043
C 5.7696 4.2636 1.2299
H 5.2156 6.9480 0.6825
H 4.6395 6.1341 2.9790
H 6.1474 6.7754 3.6102
H 7.4363 5.1553 2.2483
H 5.2376 3.8804 3.9652
H 6.7850 3.1222 3.5555
H 6.7423 4.5758 4.5683
H 4.6773 4.2412 1.2846
H 6.1162 3.2267 1.1583
H 6.0407 4.7743 0.3000
H -0.4634 -2.6829 -4.9295
H -8.6627 3.4079 3.1270
H 12.4032 4.6061 -1.7261
H 11.7213 5.5152 -3.0939
H -10.3493 2.3926 -4.8049
H -9.1029 1.1976 -5.2080
H -0.3805 3.6139 4.8778
H -1.2779 2.2043 5.4766
H 6.7940 7.5004 1.2559
H 5.3160 8.2793 1.8381
H 0.6052 -0.8407 6.9120
H 1.6121 -0.2539 8.2467
H 2.4763 -2.4113 6.2221
H 1.8726 -2.7809 7.8552
H 3.3964 -1.9089 7.6409
H 8.9198 -6.5861 -0.9292
H 9.7642 -5.4903 -2.0408
H 4.6943 -6.8289 -1.8348
H 0.4504 -7.7128 -4.9152
H -1.3141 -7.8425 -4.9049
H 3.9432 -5.5978 -5.9401
H 2.3731 1.2285 4.1548
H -4.8531 1.4261 1.1789
C 2.1241 6.5259 -4.0059
C 0.9090 5.9250 -3.3480
O 0.7861 4.7028 -3.2303
N -0.0368 6.7982 -2.9136
C -1.3721 6.2993 -2.6401
C -2.0179 5.7983 -3.9433
O -1.8151 6.3865 -5.0030
N -2.8070 4.7131 -3.7860
C -3.4133 4.0359 -4.9223
C -3.0285 2.5521 -4.9545
C -1.5169 2.2768 -4.9502
C -1.2515 0.7742 -4.8032
C -0.8086 2.8629 -6.1757
H 2.9996 5.9245 -3.7588
H 1.9806 6.4898 -5.0910
H -1.3260 5.5119 -1.8852
H -1.9715 7.1191 -2.2360
H -3.4808 2.0548 -4.0887
H -3.4877 2.0972 -5.8436
H -1.0921 2.7666 -4.0704
H 0.2633 2.6421 -6.1433
H -1.2121 2.4421 -7.1054
H -0.9170 3.9502 -6.2162
H -1.6442 0.2151 -5.6621
H -1.7271 0.3752 -3.8999
H -0.1773 0.5720 -4.7360
H -3.0858 4.5630 -5.8200
C -4.6834 -2.7960 3.8241
C -3.2850 -2.1520 3.9114
O -3.1531 -0.9346 4.0099
C -5.6625 -1.9306 3.0178
C -5.3892 -1.9644 1.5284
N -6.3532 -2.4882 0.7434
O -4.3305 -1.5256 1.0452
N -2.2186 -2.9984 3.9715
C -0.8690 -2.5017 4.2308
C -0.1529 -2.0632 2.9418
O -0.6119 -2.3406 1.8222
C -0.1619 -3.7466 4.8038
C -0.7731 -4.8768 3.9623
C -2.2444 -4.4566 3.7977
N 1.0272 -1.4553 3.1475
C 1.9295 -1.1445 2.0663
C 3.3787 -1.4545 2.4270
O 3.7021 -2.3615 3.1915
N 4.2670 -0.6359 1.8079
C 5.7012 -0.7671 2.0060
C 6.2452 0.1310 3.1489
O 7.2673 0.8140 2.9975
C 6.4775 -0.6034 0.7124
O 6.2445 0.6894 0.1206
N 5.5509 0.0299 4.2998
C 5.9530 0.6223 5.5736
H -6.6889 -2.2505 3.2150
H -5.5349 -0.8888 3.3234
H -4.6464 -3.8108 3.4207
H -0.4220 -3.8592 5.8611
H 0.9238 -3.6813 4.7179
H -0.6747 -5.8621 4.4236
H -0.2895 -4.9067 2.9821
H -2.8798 -4.9267 4.5584
H -2.6333 -4.7283 2.8103
H -0.9072 -1.6644 4.9311
H 1.8613 -0.0908 1.7931
H 1.6499 -1.7483 1.2015
H 7.5404 -0.7159 0.9276
H 6.1758 -1.3914 0.0146
H 6.9742 0.8415 -0.4977
H 5.8623 -1.8004 2.3388
H 6.7561 1.3335 5.3833
H 2.2963 7.5643 -3.7124
H -4.5063 4.1229 -4.8681
H -2.6803 4.1704 -2.9297
H 0.0142 7.7331 -3.2921
H 6.3168 -0.1545 6.2557
H 5.0942 1.1270 6.0246
H 4.7186 -0.5504 4.3191
H 1.3483 -1.1228 4.0786
H -7.2127 -2.8961 1.1164
H -6.2025 -2.4792 -0.2575
H 3.9293 0.0781 1.1636
H -5.0506 -2.8804 4.8537
C -10.5336 -4.8627 2.6728
C -9.6757 -4.2335 1.5982
O -8.5279 -3.8495 1.8515
C -10.8773 -3.7867 3.7082
N -10.2534 -4.0459 0.3926
C -9.5761 -3.3385 -0.6791
C -8.5508 7.3282 2.6347
C -8.4158 5.9927 1.9116
C -8.3793 6.1100 0.3896
N -7.9816 4.9594 -0.2434
O -8.7237 7.1162 -0.2163
H -9.9604 -3.3505 4.1108
H -11.4537 -4.2090 4.5356
H -11.4688 -2.9831 3.2584
H -11.4417 -5.3100 2.2542
H -8.6696 -3.8698 -0.9873
H -7.5240 5.4511 2.2371
H -9.2663 5.3440 2.1587
H -7.4748 4.2506 0.2737
H -7.8232 5.0252 -1.2381
H -9.4276 7.8736 2.2777
H -8.6445 7.1745 3.7138
H -7.6783 7.9625 2.4533
H -9.9507 -5.6623 3.1388
H -9.2906 -2.3303 -0.3630
H -10.2465 -3.2626 -1.5353
H -11.1944 -4.3754 0.2467
O 3.5546 1.2559 -0.4252
H 3.3263 2.0699 0.0953
H 4.5270 1.1910 -0.4634

APH^{+}

C 11.4811 5.1447 -2.0499
C 10.3519 4.0984 -2.1775
C 10.0115 3.3378 -0.9201
C 8.7717 3.1334 -0.3442
N 10.9747 2.6596 -0.1929
C 10.3278 2.0733 0.7983
N 8.9886 2.3212 0.7564
H 11.2024 5.9403 -1.3519
H 10.6453 3.3741 -2.9498
H 9.4396 4.5825 -2.5462
H 7.7905 3.5030 -0.5943
H 10.7723 1.4622 1.5711
H 8.2970 1.9410 1.4008
C -9.5797 1.7299 -4.3741
C -8.5736 2.5669 -3.5651
C -7.4723 1.7104 -2.9852
C -6.4371 1.2327 -3.8021
C -7.4815 1.3081 -1.6449
C -5.4660 0.3679 -3.3092
C -6.5131 0.4447 -1.1299
C -5.5035 -0.0401 -1.9697
O -4.5387 -0.9061 -1.5627

H	-10.0459	0.9698	-3.7390
H	-9.0958	3.0939	-2.7585
H	-8.1438	3.3380	-4.2166
H	-6.3959	1.5382	-4.8449
H	-8.2482	1.6942	-0.9782
H	-4.6702	-0.0061	-3.9446
H	-6.5171	0.1927	-0.0765
H	-4.5277	-1.0773	-0.5846
C	-0.4311	-7.1573	-4.7376
C	-0.4341	-6.8514	-3.2361
C	-1.6823	-6.1698	-2.7010
C	-1.8870	-6.0836	-1.3163
C	-2.6547	-5.5969	-3.5314
C	-3.0103	-5.4623	-0.7777
C	-3.7879	-4.9733	-3.0096
C	-3.9756	-4.9112	-1.6277
O	-5.1203	-4.3296	-1.1667
H	-0.4749	-6.2419	-5.3367
H	-0.2819	-7.7840	-2.6788
H	0.4395	-6.2261	-3.0015
H	-1.1536	-6.5161	-0.6415
H	-2.5404	-5.6407	-4.6093
H	-3.1391	-5.4059	0.3003
H	-4.5368	-4.5344	-3.6596
H	-5.0954	-4.2568	-0.2018
C	4.7574	-4.8222	-5.6916
C	4.6619	-4.6000	-4.1936
O	5.1158	-3.5990	-3.6429
C	4.6960	-3.5131	-6.4778
H	5.7195	-5.3209	-5.8696
H	5.4389	-2.8126	-6.0916
H	3.7124	-3.0425	-6.3793
H	4.8864	-3.6837	-7.5404
N	4.0805	-5.6206	-3.5028
C	4.3124	-5.7992	-2.0782
C	3.0819	-5.6328	-1.1900
C	2.5169	-4.2086	-1.1190
C	1.2452	-4.2105	-0.2673
C	3.5505	-3.2118	-0.5796
H	3.7905	-6.4300	-4.0316
H	5.0847	-5.0850	-1.7927
H	3.3591	-5.9544	-0.1769
H	2.2966	-6.3229	-1.5274
H	2.2558	-3.9097	-2.1432
H	1.4691	-4.5257	0.7585
H	0.7821	-3.2267	-0.1976
H	0.4917	-4.8914	-0.6745
H	4.4217	-3.1388	-1.2349
H	3.8760	-3.4957	0.4269
H	3.1267	-2.2067	-0.5103
C	9.3104	-5.5217	-1.1375
C	8.1297	-4.5408	-1.0177
C	8.5000	-3.0793	-1.1883
C	7.8471	-2.2812	-2.1367
C	9.4917	-2.4883	-0.3905
C	8.1816	-0.9309	-2.2844
C	9.8310	-1.1450	-0.5422
C	9.1769	-0.3549	-1.4930
H	10.0711	-5.3287	-0.3755
H	7.6525	-4.6793	-0.0376
H	7.3711	-4.7947	-1.7656
H	7.0711	-2.7126	-2.7629
H	10.0052	-3.0865	0.3572
H	7.6690	-0.3338	-3.0342
H	10.6067	-0.7043	0.0745
H	9.4545	0.6876	-1.6127
C	-2.6236	0.8737	0.4162
C	-1.4195	1.0708	-0.2194
C	-1.3125	2.2456	-1.0263
N	-0.1524	2.6497	-1.5535
N	-2.4086	3.0005	-1.2882
C	-3.5544	2.7111	-0.6848
N	-3.6712	1.7022	0.2072
S	1.8721	-1.0906	-3.0265
C	1.4844	-0.1805	-1.6543
C	-4.7642	3.5200	-1.0006
N	0.2929	-0.5033	-1.1626
C	-0.3705	-1.5311	-1.8506
C	-1.6814	-2.0526	-1.3814
C	0.3610	-1.9570	-2.9257
C	0.0166	-3.0049	-3.9340
C	-0.2520	0.1708	0.0528
H	2.2012	0.5094	-1.1854
H	-0.0689	3.5215	-2.0882
H	-0.5209	-0.6191	0.7576
H	0.5759	0.7417	0.4779
H	-5.3340	3.0158	-1.7890
H	-5.4037	3.5954	-0.1166
H	-4.4726	4.5076	-1.3578
H	-2.8011	0.0578	1.1121
H	-1.6764	-2.2218	-0.3015
H	-1.8910	-3.0057	-1.8654
H	-2.4966	-1.3709	-1.6317
H	-0.7598	-3.6657	-3.5461
H	0.8889	-3.6169	-4.1817
C	2.3947	-2.1312	7.2092
C	1.6661	-0.7864	7.1782
C	2.3468	0.1870	6.2155
O	2.4293	-0.2284	4.9839
O	2.7708	1.2775	6.6132
C	-7.9451	3.0872	3.9387
C	-7.0765	1.9160	3.4775
C	-6.3050	2.1761	2.1828
O	-6.4401	3.2671	1.5919
O	-5.5364	1.2178	1.7996
H	-8.4447	2.8494	4.8836
H	-7.3423	3.9853	4.0879
H	-7.6805	1.0124	3.3237
H	-6.3369	1.6456	4.2402
C	-0.3782	2.4368	4.9806
C	-0.2754	1.8051	3.5840
C	0.9901	2.1843	2.8845
C	1.2468	2.9114	1.7388
N	2.2321	1.8587	3.3900
C	3.1633	2.3725	2.5598
N	2.6132	3.0200	1.5358
H	0.4803	2.1685	5.6021
H	-0.3435	0.7150	3.6732
H	-1.1240	2.1156	2.9649
H	0.5375	3.3928	1.0804
H	4.2215	2.2299	2.7150
H	0.7171	2.2355	-1.2610
C	5.7263	7.3033	1.6493
C	5.7308	6.2978	2.8027
C	6.4135	4.9529	2.4940
C	6.3601	4.0455	3.7305
C	5.7821	4.2689	1.2725
H	5.1462	6.9353	0.7972
H	4.6952	6.0942	3.1086
H	6.2232	6.7488	3.6741
H	7.4715	5.1498	2.2642
H	5.3220	3.8465	4.0220
H	6.8515	3.0840	3.5532
H	6.8492	4.5224	4.5873
H	4.6910	4.2569	1.3487
H	6.1184	3.2305	1.1796
H	6.0390	4.7894	0.3437
H	-0.3560	-2.5499	-4.8580
H	-8.7100	3.3203	3.1968
H	12.3914	4.6715	-1.6729
H	11.6958	5.5965	-3.0229
H	-10.3715	2.3574	-4.7956
H	-9.0791	1.2116	-5.1981
H	-0.4055	3.5284	4.9077
H	-1.2906	2.1024	5.4835
H	6.7445	7.5042	1.2952
H	5.2879	8.2576	1.9591
H	0.6318	-0.9328	6.8371
H	1.6283	-0.3300	8.1703
H	2.4845	-2.5405	6.1991
H	1.8749	-2.8603	7.8400
H	3.4089	-2.0120	7.6054
H	8.9698	-6.5545	-1.0141
H	9.7919	-5.4326	-2.1161
H	4.7307	-6.8026	-1.9285
H	0.4830	-7.6895	-5.0169
H	-1.2838	-7.7824	-5.0196
H	3.9804	-5.5205	-6.0260
H	2.4153	1.1441	4.1494
H	-4.5990	1.5425	0.8677
C	2.1001	6.5675	-3.9281
C	0.8851	5.9809	-3.2509
O	0.7658	4.7633	-3.0790
N	-0.0718	6.8655	-2.8629
C	-1.4128	6.3719	-2.6102
C	-2.0182	5.8257	-3.9155
O	-1.7456	6.3509	-4.9919
N	-2.8620	4.7830	-3.7449
C	-3.4249	4.0631	-4.8794
C	-2.9674	2.5992	-4.8916
C	-1.4449	2.3954	-4.8775
C	-1.1121	0.9144	-4.6658
C	-0.7642	2.9583	-6.1296
H	2.9777	5.9724	-3.6732
H	1.9470	6.5085	-5.0109
H	-1.3873	5.6107	-1.8279
H	-2.0271	7.2011	-2.2497
H	-3.4019	2.0945	-4.0208
H	-3.4014	2.1094	-5.7745
H	-1.0428	2.9459	-4.0221
H	0.3153	2.7780	-6.0950
H	-1.1553	2.4863	-7.0397
H	-0.9132	4.0384	-6.2098
H	-1.4832	0.3015	-5.4970
H	-1.5675	0.5331	-3.7447
H	-0.0300	0.7625	-4.5963
H	-3.1137	4.5970	-5.7784
C	-4.6664	-2.8870	3.7774
C	-3.2493	-2.2799	3.8569
O	-3.0823	-1.0665	3.9558
C	-5.6212	-1.9771	2.9856
C	-5.3663	-1.9985	1.4936
N	-6.3507	-2.4902	0.7143
O	-4.3018	-1.5841	0.9967
N	-2.1993	-3.1481	3.9042
C	-0.8421	-2.6690	4.1565
C	-0.1562	-2.1597	2.8787
O	-0.6260	-2.3947	1.7547
C	-0.1223	-3.9418	4.6481
C	-0.7722	-5.0327	3.7842
C	-2.2462	-4.6014	3.6990
N	1.0162	-1.5363	3.0879
C	1.9043	-1.1824	2.0068
C	3.3538	-1.5315	2.3318
O	3.6706	-2.5216	2.9837
N	4.2440	-0.6447	1.8111
C	5.6770	-0.7836	1.9973
C	6.2312	0.0945	3.1502
O	7.2972	0.7117	3.0240
C	6.4525	-0.6041	0.7049
O	6.2358	0.7058	0.1490
N	5.5061	0.0407	4.2860
C	5.9527	0.5591	5.5764
H	-6.6563	-2.2644	3.1856
H	-5.4638	-0.9388	3.2923
H	-4.6598	-3.8986	3.3637
H	-0.3422	-4.0979	5.7089
H	0.9601	-3.8773	4.5235
H	-0.6589	-6.0377	4.1973
H	-0.3294	-5.0205	2.7841
H	-2.8495	-5.0848	4.4778
H	-2.6836	-4.8477	2.7257
H	-0.8600	-1.8698	4.9013
H	1.8415	-0.1160	1.7808
H	1.6095	-1.7490	1.1221
H	7.5143	-0.7390	0.9156
H	6.1365	-1.3698	-0.0111
H	6.9546	0.8569	-0.4824
H	5.8311	-1.8212	2.3192
H	6.7918	1.2338	5.4077
H	2.2702	7.6120	-3.6567

H -4.5203 4.0988 -4.8310
H -2.7947 4.2765 -2.8636
H -0.0162 7.7858 -3.2756
H 6.2789 -0.2629 6.2240
H 5.1251 1.0889 6.0543
H 4.6062 -0.4305 4.2894
H 1.3629 -1.2561 4.0236
H -7.2125 -2.8928 1.0895
H -6.2203 -2.4563 -0.2888
H 3.9054 0.1244 1.2383
H -5.0284 -2.9685 4.8088
C -10.4842 -4.9642 2.6021
C -9.6409 -4.3021 1.5361
O -8.5098 -3.8777 1.7993
C -10.8555 -3.9056 3.6455
N -10.2140 -4.1338 0.3257
C -9.5544 -3.3946 -0.7349
C -8.5816 7.2332 2.7167
C -8.5127 5.8940 1.9920
C -8.4948 6.0066 0.4696
N -8.0962 4.8548 -0.1558
O -8.8509 7.0092 -0.1388
H -9.9501 -3.4505 4.0529
H -11.4234 -4.3481 4.4683
H -11.4651 -3.1129 3.2009
H -11.3801 -5.4297 2.1774
H -8.6212 -3.8853 -1.0303
H -7.6407 5.3140 2.3018
H -9.3861 5.2821 2.2529
H -7.5789 4.1526 0.3705
H -7.9630 4.9105 -1.1546
H -9.4306 7.8223 2.3615
H -8.6810 7.0826 3.7958
H -7.6785 7.8243 2.5367
H -9.8829 -5.7539 3.0621
H -9.3187 -2.3751 -0.4141
H -10.2148 -3.3480 -1.6010
H -11.1458 -4.4860 0.1747
O 3.5731 1.3596 -0.4056
H 3.3688 2.1478 0.1585
H 4.5447 1.2435 -0.4118

IP

C 11.5691 5.1455 -2.1551
C 10.4503 4.0929 -2.3140
C 10.0162 3.3930 -1.0499
C 8.7345 3.1729 -0.5819
N 10.9254 2.7880 -0.1987
C 10.2064 2.2285 0.7570
N 8.8703 2.4278 0.5770
H 11.2413 5.9751 -1.5209
H 10.7988 3.3321 -3.0261
H 9.5685 4.5553 -2.7733
H 7.7678 3.4772 -0.9509
H 10.5939 1.6741 1.6000
H 8.1309 2.0341 1.1546
C -9.5079 1.7869 -4.4140
C -8.3774 2.4853 -3.6389
C -7.3636 1.5094 -3.0845
C -6.5255 0.7857 -3.9448
C -7.2406 1.2758 -1.7106
C -5.6106 -0.1414 -3.4573
C -6.3294 0.3485 -1.2011
C -5.5096 -0.3664 -2.0793
O -4.6095 -1.3064 -1.6657
H -10.0453 1.0877 -3.7651
H -8.8043 3.0720 -2.8176
H -7.8790 3.2014 -4.3055
H -6.5835 0.9571 -5.0169
H -7.8468 1.8453 -1.0111
H -4.9583 -0.6926 -4.1259
H -6.2363 0.2296 -0.1275
H -4.4687 -1.3071 -0.6866
C -0.4089 -7.1579 -4.5255
C -0.4120 -6.8115 -3.0327
C -1.7053 -6.2190 -2.4985
C -1.9424 -6.1905 -1.1172
C -2.6901 -5.6696 -3.3314
C -3.1073 -5.6443 -0.5842
C -3.8626 -5.1205 -2.8154
C -4.0808 -5.1124 -1.4366
O -5.2675 -4.6149 -0.9849
H -0.5177 -6.2650 -5.1492
H -0.1773 -7.7127 -2.4528
H 0.4117 -6.1112 -2.8313
H -1.2048 -6.6130 -0.4404
H -2.5523 -5.6717 -4.4075
H -3.2671 -5.6413 0.4911
H -4.6162 -4.6910 -3.4657
H -5.2354 -4.4815 -0.0264
C 4.7936 -4.8784 -5.5353
C 4.8173 -4.6620 -4.0389
O 5.4327 -3.7343 -3.5152
C 4.7569 -3.5591 -6.3085
H 5.7088 -5.4307 -5.7869
H 5.5738 -2.9134 -5.9806
H 3.8181 -3.0257 -6.1286
H 4.8500 -3.7316 -7.3837
N 4.1377 -5.5971 -3.3230
C 4.3390 -5.7550 -1.8975
C 3.0772 -5.6071 -1.0556
C 2.4334 -4.2161 -1.0825
C 1.1916 -4.2302 -0.1918
C 3.4191 -3.1166 -0.6655
H 3.7078 -6.3567 -3.8293
H 5.0799 -5.0102 -1.6078
H 3.3423 -5.8551 -0.0186
H 2.3361 -6.3557 -1.3672
H 2.1207 -4.0102 -2.1146
H 1.4534 -4.5128 0.8347
H 0.7049 -3.2596 -0.1357
H 0.4482 -4.9450 -0.5554
H 4.2528 -3.0282 -1.3674
H 3.8242 -3.3152 0.3344
H 2.9247 -2.1422 -0.6337
C 9.3374 -5.4801 -0.9579
C 8.1997 -4.4504 -0.8661
C 8.6299 -3.0148 -1.0968
C 8.0091 -2.2386 -2.0833
C 9.6334 -2.4231 -0.3138
C 8.3818 -0.9039 -2.2793
C 10.0044 -1.0933 -0.5058
C 9.3792 -0.3219 -1.4918
H 10.1164 -5.2879 -0.2141
H 7.7298 -4.5297 0.1246
H 7.4224 -4.6996 -1.5953
H 7.2300 -2.6814 -2.6960
H 10.1241 -3.0078 0.4597
H 7.8983 -0.3228 -3.0602
H 10.7827 -0.6477 0.1047
H 9.6778 0.7102 -1.6423
C -2.6735 0.8380 0.3272
C -1.4746 0.9581 -0.3005
C -1.2274 2.1222 -1.1424
N -0.0237 2.3593 -1.5653
N -2.3299 2.9425 -1.4211
C -3.4724 2.7420 -0.8058
N -3.6641 1.7466 0.1024
S 1.6618 -0.9861 -3.2514
C 1.2562 -0.1234 -1.8480
C -4.6510 3.6106 -1.1116
N 0.1881 -0.6198 -1.2478
C -0.4036 -1.7138 -1.8981
C -1.6493 -2.3328 -1.3674
C 0.2876 -2.0524 -3.0253
C -0.0129 -3.1241 -4.0222
C -0.3532 0.0225 -0.0142
H 1.8235 0.7047 -1.4600
H 0.0265 3.2113 -2.1287
H -0.6381 -0.7762 0.6688
H 0.4869 0.5722 0.4078
H -5.2775 3.1090 -1.8579
H -5.2639 3.7456 -0.2161
H -4.3210 4.5668 -1.5166
H -2.9148 0.0424 1.0244
H -1.6084 -2.4681 -0.2849
H -1.8052 -3.3094 -1.8230
H -2.5145 -1.7114 -1.6088
H -0.7622 -3.8077 -3.6214
H 0.8823 -3.7049 -4.2673
C 2.4312 -1.8269 7.2851
C 1.7631 -0.4468 7.3912
C 2.3208 0.5700 6.4127
O 2.1148 0.2072 5.1374
O 2.8807 1.6005 6.7320
C -7.8753 3.3591 3.8612
C -7.1058 2.1109 3.4351
C -6.3510 2.2574 2.1077
O -6.4754 3.3211 1.4544
O -5.6249 1.2634 1.7689
H -8.3767 3.2009 4.8228
H -7.2048 4.2150 3.9626
H -7.7773 1.2476 3.3363
H -6.3681 1.8210 4.1930
C -0.3134 2.6948 4.9303
C -0.3788 1.8456 3.6577
C 0.7651 2.1724 2.7569
C 0.7569 2.8426 1.5603
N 2.0804 1.8823 3.1048
C 2.8439 2.3536 2.1321
N 2.0811 2.9476 1.1785
H 0.6395 2.5672 5.4494
H -0.3695 0.7813 3.9097
H -1.3181 2.0219 3.1255
H -0.0389 3.2245 0.9460
H 3.9176 2.2918 2.0807
H 2.3816 3.3170 0.2881
C 5.8222 7.4354 1.4768
C 5.5677 6.3780 2.5516
C 6.1247 4.9770 2.2453
C 5.8979 4.0490 3.4446
C 5.5160 4.3832 0.9646
H 5.3117 7.1968 0.5381
H 4.4872 6.2912 2.7349
H 6.0065 6.7188 3.4979
H 7.2089 5.0666 2.0845
H 4.8271 3.9169 3.6416
H 6.3433 3.0646 3.2793
H 6.3429 4.4700 4.3524
H 4.4215 4.4201 1.0201
H 5.8226 3.3377 0.8332
H 5.8207 4.9370 0.0719
H -0.4076 -2.6975 -4.9508
H -8.6312 3.6196 3.1195
H 12.4478 4.6936 -1.6881
H 11.8563 5.5479 -3.1309
H -10.2278 2.5127 -4.8060
H -9.1099 1.2153 -5.2584
H -0.4158 3.7573 4.6890
H -1.1203 2.4251 5.6188
H 6.8924 7.5256 1.2581
H 5.4649 8.4177 1.8010
H 0.6896 -0.5408 7.1858
H 1.8797 -0.0312 8.3939
H 2.3833 -2.2130 6.2632
H 1.9430 -2.5410 7.9539
H 3.4871 -1.7696 7.5674
H 8.9583 -6.4927 -0.7886
H 9.8083 -5.4511 -1.9453
H 4.7836 -6.7425 -1.7131
H 0.5330 -7.6398 -4.8051
H -1.2251 -7.8420 -4.7766
H 3.9533 -5.5229 -5.8193
H 2.2993 0.9553 4.4621
H -4.5597 1.6450 0.6911
C 2.1984 6.5694 -4.0834
C 1.0987 5.9086 -3.2826
O 1.1144 4.7077 -3.0238
N 0.0878 6.7466 -2.8898
C -1.2103 6.1597 -2.6021

C	-1.8752	5.7293	-3.9202
O	-1.6559	6.3578	-4.9567
N	-2.6921	4.6638	-3.8119
C	-3.3395	4.0712	-4.9740
C	-3.3136	2.5422	-4.8790
C	-1.9111	1.9257	-4.7502
C	-2.0174	0.4240	-4.4637
C	-1.0385	2.2013	-5.9791
H	3.1051	5.9675	-4.0212
H	1.8805	6.6167	-5.1309
H	-1.0936	5.3209	-1.9157
H	-1.8384	6.9117	-2.1147
H	-3.9185	2.2291	-4.0201
H	-3.8152	2.1318	-5.7660
H	-1.4141	2.3815	-3.8886
H	-0.0527	1.7372	-5.8649
H	-1.4976	1.7961	-6.8899
H	-0.8801	3.2735	-6.1276
H	-2.5302	-0.1011	-5.2800
H	-2.5767	0.2332	-3.5421
H	-1.0236	-0.0207	-4.3546
H	-2.8217	4.4375	-5.8639
C	-4.6301	-2.6356	3.8657
C	-3.1895	-2.1089	3.9055
O	-2.9397	-0.9073	3.9514
C	-5.5474	-1.7930	2.9650
C	-5.2787	-2.0010	1.4905
N	-6.2777	-2.5221	0.7515
O	-4.1848	-1.7036	0.9745
N	-2.1933	-3.0393	4.0036
C	-0.8202	-2.6186	4.2384
C	-0.1172	-2.1839	2.9460
O	-0.5132	-2.5537	1.8383
C	-0.1557	-3.9124	4.7551
C	-0.8587	-4.9947	3.9215
C	-2.3094	-4.4914	3.8166
N	1.0156	-1.4752	3.1429
C	1.9723	-1.2142	2.0954
C	3.4011	-1.3848	2.5995
O	3.6776	-2.0651	3.5911
N	4.3411	-0.7383	1.8649
C	5.7625	-0.8968	2.1625
C	6.2712	0.1387	3.1980
O	7.1549	0.9601	2.9305
C	6.6173	-0.9594	0.9088
O	6.4767	0.2206	0.1045
N	5.6780	0.0049	4.4043
C	6.0061	0.7985	5.5850
H	-6.5915	-2.0276	3.1872
H	-5.3776	-0.7258	3.1421
H	-4.6750	-3.6897	3.5815
H	-0.3789	-4.0324	5.8202
H	0.9292	-3.9000	4.6286
H	-0.7979	-5.9899	4.3681
H	-0.4150	-5.0353	2.9236
H	-2.9423	-4.9350	4.5951
H	-2.7466	-4.7255	2.8408
H	-0.7991	-1.8013	4.9640
H	1.8641	-0.2076	1.6865
H	1.7920	-1.9246	1.2873
H	7.6622	-1.0714	1.2065
H	6.3252	-1.8408	0.3267
H	7.1978	0.1897	-0.5473
H	5.8528	-1.8771	2.6446
H	6.8172	1.4764	5.3198
H	2.4032	7.5886	-3.7438
H	-4.3799	4.4152	-5.0440
H	-2.6112	4.0960	-2.9605
H	0.0210	7.5952	-3.4382
H	6.3370	0.1434	6.3973
H	5.1383	1.3732	5.9188
H	4.9718	-0.7217	4.4882
H	1.2604	-1.1391	4.0705
H	-7.1636	-2.8348	1.1516
H	-6.1387	-2.5835	-0.2498
H	4.0612	-0.1105	1.1128
H	-4.9988	-2.5721	4.8965
C	-10.5456	-4.7108	2.7120
C	-9.6604	-4.1177	1.6392
O	-8.5025	-3.7699	1.8974
C	-10.8247	-3.6228	3.7532
N	-10.2236	-3.9212	0.4287
C	-9.5157	-3.2376	-0.6378
C	-8.4871	7.4740	2.5270
C	-8.3987	6.1247	1.8235
C	-8.3341	6.2198	0.3009
N	-7.9460	5.0553	-0.3000
O	-8.6437	7.2292	-0.3239
H	-9.8828	-3.2374	4.1495
H	-11.4182	-4.0155	4.5833
H	-11.3743	-2.7869	3.3094
H	-11.4784	-5.1052	2.2945
H	-8.6282	-3.8020	-0.9425
H	-7.5377	5.5453	2.1654
H	-9.2789	5.5157	2.0677
H	-7.4852	4.3287	0.2480
H	-7.7804	5.0968	-1.2946
H	-9.3294	8.0550	2.1435
H	-8.6104	7.3396	3.6060
H	-7.5824	8.0657	2.3571
H	-10.0053	-5.5443	3.1705
H	-9.1946	-2.2410	-0.3198
H	-10.1780	-3.1359	-1.4977
H	-11.1779	-4.2088	0.2820
O	3.9757	0.8941	-0.6246
H	4.1469	1.8430	-0.5938
H	4.8842	0.5161	-0.6157

YI

C	11.4691	5.0975	-2.2313
C	10.3472	4.0424	-2.3468
C	9.9326	3.3739	-1.0591
C	8.6576	3.1442	-0.5772
N	10.8581	2.8099	-0.1971
C	10.1535	2.2652	0.7787
N	8.8134	2.4345	0.6004
H	11.1514	5.9431	-1.6131
H	10.6839	3.2649	-3.0466
H	9.4586	4.4947	-2.8034
H	7.6829	3.4230	-0.9451
H	10.5553	1.7402	1.6337
H	8.0831	2.0515	1.1980
C	-9.5977	1.6295	-4.4187
C	-8.5616	2.4539	-3.6315
C	-7.4755	1.5910	-3.0285
C	-6.4740	1.0358	-3.8383
C	-7.4569	1.2718	-1.6660
C	-5.5128	0.1761	-3.3184
C	-6.4983	0.4121	-1.1251
C	-5.5248	-0.1546	-1.9572
O	-4.5908	-1.0442	-1.5294
H	-10.0856	0.8986	-3.7657
H	-9.0664	3.0166	-2.8377
H	-8.1144	3.1973	-4.3035
H	-6.4472	1.2809	-4.8973
H	-8.2013	1.7138	-1.0078
H	-4.7402	-0.2515	-3.9485
H	-6.4948	0.1997	-0.0630
H	-4.4970	-1.0854	-0.5442
C	-0.4597	-7.2758	-4.4828
C	-0.4611	-6.9182	-2.9927
C	-1.7320	-6.2696	-2.4706
C	-1.8980	-6.0773	-1.0919
C	-2.7650	-5.8331	-3.3100
C	-3.0425	-5.4831	-0.5671
C	-3.9191	-5.2383	-2.8016
C	-4.0664	-5.0667	-1.4247
O	-5.2330	-4.5184	-0.9734
H	-0.5543	-6.3844	-5.1110
H	-0.2636	-7.8236	-2.4049
H	0.3842	-6.2456	-2.7884
H	-1.1173	-6.4036	-0.4101
H	-2.6808	-5.9603	-4.3838
H	-3.1415	-5.3422	0.5064
H	-4.7131	-4.9021	-3.4591
H	-5.1726	-4.3387	-0.0242
C	4.7306	-4.9812	-5.5212
C	4.8750	-4.8252	-4.0264
O	5.6737	-4.0376	-3.5221
C	4.7370	-3.6267	-6.2351
H	5.5837	-5.5829	-5.8602
H	5.6269	-3.0623	-5.9495
H	3.8611	-3.0332	-5.9539
H	4.7307	-3.7542	-7.3207
N	4.0766	-5.6437	-3.2926
C	4.2876	-5.8323	-1.8758
C	3.0558	-5.5697	-1.0131
C	2.5294	-4.1299	-1.0468
C	1.2896	-4.0257	-0.1583
C	3.5994	-3.1107	-0.6339
H	3.4895	-6.3018	-3.7825
H	5.1011	-5.1641	-1.5960
H	3.3156	-5.8330	0.0215
H	2.2497	-6.2572	-1.3068
H	2.2345	-3.9030	-2.0784
H	1.5315	-4.2780	0.8812
H	0.8703	-3.0217	-0.1564
H	0.4961	-4.7017	-0.4912
H	4.4504	-3.1127	-1.3209
H	3.9688	-3.3211	0.3776
H	3.1903	-2.0972	-0.6339
C	9.2868	-5.5284	-0.9493
C	8.1488	-4.4970	-0.8571
C	8.5906	-3.0576	-1.0391
C	8.0314	-2.2650	-2.0496
C	9.5480	-2.4797	-0.1910
C	8.4207	-0.9301	-2.2061
C	9.9370	-1.1500	-0.3461
C	9.3747	-0.3636	-1.3571
H	10.0516	-5.3539	-0.1863
H	7.6575	-4.6000	0.1209
H	7.3896	-4.7219	-1.6121
H	7.2903	-2.6983	-2.7135
H	9.9899	-3.0766	0.6028
H	7.9869	-0.3367	-3.0069
H	10.6804	-0.7168	0.3152
H	9.6900	0.6669	-1.4810
C	-2.5077	0.7888	0.2169
C	-1.3274	0.9115	-0.4877
C	-1.2000	2.0758	-1.2961
N	-0.0321	2.4267	-1.8579
N	-2.2623	2.8967	-1.4782
C	-3.3902	2.6419	-0.8092
N	-3.5412	1.6493	0.0847
S	1.7980	-1.2327	-3.4098
C	1.5033	-0.2569	-2.0230
C	-4.5763	3.5102	-1.0950
N	0.3377	-0.6806	-1.4988
C	-0.3432	-1.7481	-2.1233
C	-1.6312	-2.2631	-1.5726
C	0.3387	-2.1885	-3.2154
C	-0.0038	-3.2998	-4.1549
C	-0.1800	-0.0294	-0.2698
H	0.7411	1.7695	-1.8930
H	0.0470	3.2773	-2.4178
H	-0.4551	-0.8057	0.4452
H	0.6737	0.5154	0.1321
H	-5.2544	2.9765	-1.7704
H	-5.1208	3.7124	-0.1732
H	-4.2729	4.4419	-1.5717
H	-2.6527	-0.0217	0.9256
H	-1.5588	-2.4667	-0.5004
H	-1.9050	-3.1937	-2.0686
H	-2.4474	-1.5536	-1.7290
H	-0.8801	-3.8470	-3.8030
H	0.8256	-4.0110	-4.2424
C	2.3824	-1.8435	7.2811
C	1.7994	-0.4652	7.6086
C	2.2996	0.5890	6.6441
O	1.9664	0.2994	5.3808

O 2.9325 1.5768 6.9656
C -7.9542 3.2712 3.8408
C -7.0406 2.0989 3.4868
C -6.1873 2.3524 2.2582
O -6.1979 3.4204 1.6537
O -5.4378 1.3156 1.9300
H -8.5212 3.0503 4.7500
H -7.3762 4.1815 4.0083
H -7.6122 1.1794 3.3106
H -6.3527 1.8618 4.3077
C -0.3872 2.6482 4.8983
C -0.3719 2.0542 3.4875
C 0.9445 2.2499 2.8031
C 1.2232 2.6315 1.5127
N 2.1451 2.0035 3.4580
C 3.1119 2.2174 2.5807
N 2.6022 2.6173 1.3928
H 0.3801 2.1998 5.5273
H -0.6052 0.9837 3.5434
H -1.1615 2.4999 2.8733
H 0.5884 2.9198 0.6906
H 4.1665 2.0885 2.7615
H 3.1100 2.5168 0.5137
C 5.7199 7.3895 1.3959
C 5.6605 6.4380 2.5915
C 6.2805 5.0499 2.3538
C 6.1829 4.2049 3.6299
C 5.6245 4.3312 1.1658
H 5.1327 7.0157 0.5511
H 4.6131 6.3012 2.8964
H 6.1663 6.9026 3.4478
H 7.3462 5.1862 2.1174
H 5.1346 4.0407 3.9086
H 6.6554 3.2283 3.4969
H 6.6670 4.7092 4.4735
H 4.5347 4.3481 1.2687
H 5.9385 3.2833 1.1059
H 5.8730 4.8097 0.2135
H -0.2220 -2.9190 -5.1587
H -8.6623 3.4687 3.0352
H 12.3538 4.6563 -1.7656
H 11.7423 5.4751 -3.2211
H -10.3702 2.2733 -4.8514
H -9.1173 1.0778 -5.2328
H -0.2039 3.7267 4.8707
H -1.3588 2.4779 5.3722
H 6.7510 7.5228 1.0481
H 5.3270 8.3772 1.6575
H 0.7055 -0.4958 7.5340
H 2.0633 -0.1490 8.6197
H 2.1861 -2.1202 6.2430
H 1.9609 -2.6122 7.9356
H 3.4684 -1.8473 7.4184
H 8.9018 -6.5437 -0.8110
H 9.7764 -5.4780 -1.9267
H 4.6395 -6.8584 -1.6994
H 0.4752 -7.7748 -4.7557
H -1.2841 -7.9509 -4.7321
H 3.8261 -5.5486 -5.7703
H 2.1797 1.0757 4.7265
H -4.7755 1.5152 1.1652
C 2.0880 6.4657 -4.1497
C 0.8943 5.8341 -3.4806
O 0.7853 4.6098 -3.3881
N -0.0480 6.6929 -3.0041
C -1.3762 6.1760 -2.7266
C -2.0325 5.6935 -4.0316
O -1.8446 6.2988 -5.0846
N -2.8139 4.6036 -3.8787
C -3.4407 3.9366 -5.0094
C -3.1041 2.4424 -5.0206
C -1.6031 2.1196 -5.0597
C -1.3791 0.6180 -4.8539
C -0.9290 2.6271 -6.3385
H 2.9686 5.8475 -3.9718
H 1.9027 6.4931 -5.2289
H -1.3167 5.3748 -1.9877
H -1.9794 6.9828 -2.3016
H -3.5440 1.9796 -4.1294
H -3.6056 1.9812 -5.8831
H -1.1289 2.6371 -4.2210
H 0.1345 2.3666 -6.3431
H -1.3881 2.1831 -7.2310
H -0.9999 3.7154 -6.4226
H -1.8407 0.0334 -5.6601
H -1.8137 0.2793 -3.9069
H -0.3121 0.3770 -4.8361
H -3.0967 4.4430 -5.9133
C -4.6826 -2.7090 3.8833
C -3.2812 -2.0836 3.9727
O -3.1279 -0.8694 4.0762
C -5.6505 -1.8536 3.0527
C -5.3612 -1.9271 1.5677
N -6.3175 -2.4653 0.7825
O -4.2913 -1.5119 1.0915
N -2.2271 -2.9465 4.0357
C -0.8762 -2.4563 4.2761
C -0.1866 -2.0105 2.9777
O -0.5955 -2.3755 1.8722
C -0.1566 -3.7115 4.8130
C -0.7901 -4.8314 3.9731
C -2.2616 -4.4013 3.8348
N 0.9432 -1.2964 3.1723
C 1.8956 -1.0336 2.1197
C 3.3266 -1.2522 2.5963
O 3.5909 -1.9127 3.6085
N 4.2616 -0.6630 1.8204
C 5.6882 -0.8209 2.0838
C 6.2086 0.1877 3.1440
O 7.0991 1.0081 2.8954
C 6.5079 -0.8198 0.8037
O 6.3098 0.3771 0.0408
N 5.6115 0.0332 4.3469
C 5.9420 0.7846 5.5534
H -6.6800 -2.1633 3.2492
H -5.5202 -0.8052 3.3342
H -4.6535 -3.7322 3.5015
H -0.3893 -3.8373 5.8753
H 0.9277 -3.6480 4.7004
H -0.6901 -5.8198 4.4274
H -0.3239 -4.8572 2.9849
H -2.8914 -4.8821 4.5932
H -2.6605 -4.6511 2.8463
H -0.8993 -1.6279 4.9881
H 1.8028 -0.0148 1.7405
H 1.6873 -1.7144 1.2928
H 7.5618 -0.9151 1.0746
H 6.2268 -1.6952 0.2071
H 7.0436 0.4078 -0.5929
H 5.8095 -1.8161 2.5293
H 6.7447 1.4813 5.3120
H 2.2759 7.4854 -3.8043
H -4.5304 4.0611 -4.9578
H -2.6764 4.0564 -3.0261
H -0.0161 7.6307 -3.3785
H 6.2849 0.1026 6.3385
H 5.0718 1.3369 5.9180
H 4.9070 -0.6995 4.4128
H 1.2030 -0.9909 4.1073
H -7.1913 -2.8444 1.1533
H -6.1553 -2.4714 -0.2173
H 3.9683 -0.0774 1.0296
H -5.0581 -2.7685 4.9119
C -10.4757 -4.8171 2.7943
C -9.6433 -4.1841 1.7018
O -8.5184 -3.7327 1.9470
C -10.8731 -3.7242 3.7918
N -10.2220 -4.0695 0.4881
C -9.5752 -3.3663 -0.6047
C -8.5871 7.3732 2.4770
C -8.4256 6.0151 1.8045
C -8.3850 6.0764 0.2797
N -7.9078 4.9314 -0.3045
O -8.7939 7.0324 -0.3666
H -9.9789 -3.2331 4.1820
H -11.4341 -4.1440 4.6310
H -11.4981 -2.9642 3.3126
H -11.3606 -5.3189 2.3882
H -8.6547 -3.8753 -0.9095
H -7.5292 5.4988 2.1562
H -9.2703 5.3658 2.0691
H -7.3558 4.2801 0.2413
H -7.7565 4.9623 -1.3020
H -9.4613 7.8960 2.0823
H -8.7013 7.2574 3.5589
H -7.7167 8.0096 2.2915
H -9.8592 -5.5738 3.2882
H -9.3194 -2.3435 -0.3120
H -10.2542 -3.3301 -1.4568
H -11.1432 -4.4536 0.3498
O 3.6419 0.9508 -0.4954
H 4.5530 0.7227 -0.7513
H 2.9817 0.5691 -1.1362

YIH^+

C 11.4657 5.1255 -2.2010
C 10.3491 4.0661 -2.3266
C 9.9325 3.3885 -1.0443
C 8.6566 3.1467 -0.5707
N 10.8574 2.8269 -0.1802
C 10.1517 2.2716 0.7888
N 8.8114 2.4321 0.6041
H 11.1419 5.9664 -1.5797
H 10.6921 3.2938 -3.0292
H 9.4603 4.5168 -2.7843
H 7.6819 3.4199 -0.9427
H 10.5529 1.7449 1.6431
H 8.0808 2.0384 1.1942
C -9.5968 1.6464 -4.4115
C -8.5638 2.4705 -3.6202
C -7.4676 1.6109 -3.0305
C -6.4681 1.0665 -3.8507
C -7.4342 1.2887 -1.6689
C -5.4952 0.2136 -3.3408
C -6.4635 0.4367 -1.1360
C -5.4935 -0.1182 -1.9794
O -4.5346 -0.9862 -1.5594
H -10.0764 0.9055 -3.7638
H -9.0699 3.0195 -2.8180
H -8.1262 3.2252 -4.2861
H -6.4534 1.3131 -4.9097
H -8.1712 1.7270 -1.0004
H -4.7261 -0.2086 -3.9790
H -6.4380 0.2460 -0.0697
H -4.4958 -1.1079 -0.5769
C -0.4459 -7.2447 -4.5578
C -0.4473 -6.9003 -3.0645
C -1.7108 -6.2435 -2.5354
C -1.8790 -6.0768 -1.1534
C -2.7327 -5.7720 -3.3696
C -3.0143 -5.4727 -0.6207
C -3.8775 -5.1662 -2.8531
C -4.0263 -5.0187 -1.4734
O -5.1813 -4.4530 -1.0151
H -0.5286 -6.3468 -5.1786
H -0.2595 -7.8129 -2.4850
H 0.4052 -6.2388 -2.8533
H -1.1064 -6.4305 -0.4759
H -2.6466 -5.8795 -4.4454
H -3.1139 -5.3492 0.4549
H -4.6624 -4.8013 -3.5063
H -5.1212 -4.2961 -0.0621
C 4.7407 -4.9334 -5.5777
C 4.8927 -4.7955 -4.0817
O 5.7068 -4.0279 -3.5715
C 4.7860 -3.5742 -6.2806
H 5.5750 -5.5572 -5.9236
H 5.6914 -3.0376 -5.9905
H 3.9270 -2.9583 -5.9951
H 4.7762 -3.6928 -7.3671

N 4.0831 -5.6082 -3.3531
C 4.3003 -5.8174 -1.9398
C 3.0721 -5.5639 -1.0699
C 2.5491 -4.1225 -1.0838
C 1.3063 -4.0298 -0.1986
C 3.6195 -3.1122 -0.6509
H 3.4870 -6.2549 -3.8477
H 5.1164 -5.1550 -1.6541
H 3.3340 -5.8410 -0.0395
H 2.2634 -6.2452 -1.3701
H 2.2595 -3.8803 -2.1136
H 1.5435 -4.3014 0.8370
H 0.8899 -3.0248 -0.1800
H 0.5117 -4.6969 -0.5465
H 4.4767 -3.1098 -1.3301
H 3.9791 -3.3357 0.3612
H 3.2147 -2.0967 -0.6418
C 9.2994 -5.5145 -1.0125
C 8.1576 -4.4872 -0.9200
C 8.5978 -3.0446 -1.0798
C 8.0448 -2.2393 -2.0838
C 9.5485 -2.4765 -0.2177
C 8.4347 -0.9024 -2.2208
C 9.9377 -1.1447 -0.3532
C 9.3827 -0.3461 -1.3585
H 10.0555 -5.3478 -0.2391
H 7.6565 -4.6030 0.0515
H 7.4073 -4.7048 -1.6859
H 7.3093 -2.6645 -2.7591
H 9.9854 -3.0830 0.5716
H 8.0069 -0.2992 -3.0177
H 10.6762 -0.7197 0.3190
H 9.6989 0.6856 -1.4684
C -2.5934 0.8020 0.2967
C -1.4206 0.9353 -0.4050
C -1.2931 2.1125 -1.2074
N -0.1316 2.4643 -1.7650
N -2.3566 2.9373 -1.3921
C -3.4860 2.6978 -0.7384
N -3.6106 1.6869 0.1496
S 1.7772 -1.1102 -3.3158
C 1.4378 -0.1555 -1.9267
C -4.6754 3.5541 -1.0050
N 0.2803 -0.6207 -1.4172
C -0.3598 -1.7046 -2.0576
C -1.6340 -2.2663 -1.5243
C 0.3472 -2.1123 -3.1466
C 0.0510 -3.2257 -4.0993
C -0.2668 -0.0013 -0.1869
H 0.6552 1.8179 -1.7780
H -0.0356 3.3302 -2.3020
H -0.5442 -0.7944 0.5084
H 0.5679 0.5507 0.2442
H -5.3336 3.0315 -1.7090
H -5.2417 3.7098 -0.0816
H -4.3683 4.5013 -1.4473
H -2.7723 -0.0046 1.0016
H -1.5663 -2.4748 -0.4529
H -1.8693 -3.2023 -2.0291
H -2.4713 -1.5844 -1.6900
H -0.8087 -3.8060 -3.7600
H 0.9055 -3.9063 -4.1861
C 2.3927 -1.9128 7.2527
C 1.8119 -0.5379 7.5968
C 2.3046 0.5232 6.6369
O 1.9721 0.2373 5.3720
O 2.9328 1.5131 6.9609
C -7.9526 3.2172 3.8617
C -7.0632 2.0432 3.4455
C -6.2282 2.2935 2.1860
O -6.2849 3.4096 1.6258
O -5.4978 1.3082 1.8021
H -8.4864 2.9861 4.7897
H -7.3590 4.1190 4.0217
H -7.6575 1.1370 3.2724
H -6.3578 1.7799 4.2436
C -0.3844 2.5959 4.9109
C -0.3588 2.0199 3.4932
C 0.9597 2.2360 2.8202
C 1.2457 2.6643 1.5461
N 2.1569 1.9681 3.4722
C 3.1284 2.2157 2.6097
N 2.6253 2.6589 1.4346
H 0.3855 2.1467 5.5364
H -0.5841 0.9468 3.5341
H -1.1490 2.4699 2.8827
H 0.6142 2.9868 0.7343
H 4.1823 2.0810 2.7910
H 3.1402 2.5877 0.5577
C 5.7146 7.3769 1.4484
C 5.6880 6.4194 2.6404
C 6.3119 5.0367 2.3820
C 6.2311 4.1787 3.6504
C 5.6450 4.3284 1.1938
H 5.1102 7.0037 0.6156
H 4.6481 6.2735 2.9661
H 6.2082 6.8831 3.4883
H 7.3742 5.1794 2.1351
H 5.1863 4.0105 3.9400
H 6.7026 3.2038 3.5021
H 6.7244 4.6753 4.4932
H 4.5562 4.3518 1.3048
H 5.9526 3.2792 1.1250
H 5.8895 4.8115 0.2430
H -0.1721 -2.8424 -5.1010
H -8.6908 3.4375 3.0896
H 12.3506 4.6859 -1.7340
H 11.7412 5.5095 -3.1877
H -10.3759 2.2890 -4.8339
H -9.1163 1.1065 -5.2336
H -0.2111 3.6762 4.8968
H -1.3559 2.4111 5.3789
H 6.7367 7.5179 1.0777
H 5.3224 8.3611 1.7239
H 0.7174 -0.5678 7.5311
H 2.0838 -0.2296 8.6082
H 2.1830 -2.1838 6.2157
H 1.9812 -2.6862 7.9081
H 3.4804 -1.9159 7.3757
H 8.9155 -6.5323 -0.8910
H 9.7994 -5.4509 -1.9838
H 4.6508 -6.8465 -1.7787
H 0.4839 -7.7520 -4.8325
H -1.2776 -7.9077 -4.8152
H 3.8195 -5.4722 -5.8294
H 2.1857 1.0182 4.7253
H -4.5183 1.5769 0.8304
C 2.0820 6.4970 -4.1038
C 0.9026 5.8795 -3.3918
O 0.8167 4.6616 -3.2162
N -0.0599 6.7493 -2.9759
C -1.3886 6.2302 -2.7078
C -2.0141 5.7115 -4.0142
O -1.7688 6.2678 -5.0823
N -2.8370 4.6532 -3.8518
C -3.4433 3.9677 -4.9839
C -3.0829 2.4795 -4.9901
C -1.5775 2.1792 -5.0245
C -1.3345 0.6797 -4.8258
C -0.9053 2.7012 -6.2988
H 2.9662 5.8802 -3.9417
H 1.8631 6.5120 -5.1770
H -1.3361 5.4488 -1.9476
H -2.0065 7.0413 -2.3128
H -3.5210 2.0103 -4.1011
H -3.5741 2.0097 -5.8537
H -1.1141 2.7013 -4.1818
H 0.1605 2.4503 -6.3027
H -1.3580 2.2574 -7.1945
H -0.9869 3.7891 -6.3766
H -1.7865 0.0950 -5.6371
H -1.7680 0.3274 -3.8832
H -0.2648 0.4525 -4.8073
H -3.1002 4.4774 -5.8856
C -4.6723 -2.7584 3.8499
C -3.2579 -2.1572 3.9273
O -3.0794 -0.9441 4.0040
C -5.6210 -1.8787 3.0183
C -5.3525 -1.9609 1.5306
N -6.3430 -2.4487 0.7577
O -4.2704 -1.5954 1.0348
N -2.2144 -3.0326 4.0066
C -0.8607 -2.5478 4.2453
C -0.1799 -2.0720 2.9536
O -0.5837 -2.4267 1.8427
C -0.1362 -3.8123 4.7540
C -0.7885 -4.9212 3.9145
C -2.2615 -4.4878 3.8157
N 0.9376 -1.3390 3.1549
C 1.8891 -1.0547 2.1062
C 3.3220 -1.2811 2.5759
O 3.5889 -1.9614 3.5737
N 4.2555 -0.6770 1.8092
C 5.6824 -0.8420 2.0666
C 6.2078 0.1562 3.1334
O 7.1011 0.9750 2.8905
C 6.5010 -0.8361 0.7856
O 6.3090 0.3661 0.0293
N 5.6104 -0.0044 4.3356
C 5.9478 0.7357 5.5472
H -6.6575 -2.1574 3.2232
H -5.4666 -0.8282 3.2839
H -4.6640 -3.7838 3.4725
H -0.3499 -3.9492 5.8189
H 0.9463 -3.7499 4.6237
H -0.6787 -5.9156 4.3533
H -0.3451 -4.9336 2.9154
H -2.8716 -4.9624 4.5944
H -2.6901 -4.7422 2.8411
H -0.8768 -1.7341 4.9745
H 1.7970 -0.0280 1.7477
H 1.6804 -1.7193 1.2662
H 7.5545 -0.9376 1.0558
H 6.2159 -1.7067 0.1840
H 7.0433 0.3964 -0.6040
H 5.8000 -1.8409 2.5045
H 6.7428 1.4409 5.3051
H 2.2782 7.5212 -3.7766
H -4.5347 4.0750 -4.9382
H -2.7540 4.1344 -2.9778
H -0.0384 7.6603 -3.4129
H 6.3041 0.0482 6.3214
H 5.0771 1.2774 5.9259
H 4.9066 -0.7376 4.3989
H 1.1964 -1.0450 4.0940
H -7.2129 -2.8249 1.1402
H -6.1976 -2.4615 -0.2440
H 3.9632 -0.0663 1.0373
H -5.0448 -2.8039 4.8800
C -10.4732 -4.8656 2.7409
C -9.6383 -4.2245 1.6552
O -8.5117 -3.7804 1.9050
C -10.8613 -3.7817 3.7517
N -10.2155 -4.0969 0.4419
C -9.5656 -3.3830 -0.6420
C -8.5919 7.3302 2.5345
C -8.4790 5.9641 1.8699
C -8.4592 6.0084 0.3446
N -8.0164 4.8445 -0.2264
O -8.8506 6.9682 -0.3095
H -9.9632 -3.3007 4.1453
H -11.4234 -4.2075 4.5873
H -11.4822 -3.0120 3.2828
H -11.3620 -5.3568 2.3300
H -8.6348 -3.8799 -0.9345
H -7.5926 5.4233 2.2087
H -9.3364 5.3403 2.1541
H -7.4683 4.1922 0.3308
H -7.8796 4.8595 -1.2263
H -9.4568 7.8766 2.1504
H -8.6923 7.2245 3.6190

H -7.7066 7.9409 2.3322
H -9.8610 -5.6322 3.2247
H -9.3278 -2.3564 -0.3468
H -10.2341 -3.3565 -1.5028
H -11.1420 -4.4671 0.3019
O 3.6578 1.0075 -0.4575
H 4.5557 0.7523 -0.7341
H 2.9708 0.6444 -1.0756

TC

C 11.4885 4.9675 -2.4192
C 10.4480 3.8356 -2.5445
C 10.0338 3.1938 -1.2453
C 8.7609 2.9422 -0.7705
N 10.9649 2.6968 -0.3494
C 10.2646 2.1707 0.6399
N 8.9232 2.2903 0.4382
H 11.0876 5.8068 -1.8421
H 10.8645 3.0579 -3.1993
H 9.5500 4.2096 -3.0505
H 7.7832 3.1712 -1.1641
H 10.6724 1.6959 1.5211
H 8.1910 1.9133 1.0365
C -9.6046 1.4625 -4.2684
C -8.1490 1.9430 -4.1269
C -7.2485 0.9602 -3.4085
C -6.8114 -0.2126 -4.0415
C -6.8337 1.1809 -2.0910
C -5.9868 -1.1254 -3.3920
C -6.0048 0.2764 -1.4235
C -5.5672 -0.8763 -2.0801
O -4.7370 -1.7965 -1.4962
H -10.0527 1.2929 -3.2841
H -8.1363 2.8983 -3.5897
H -7.7432 2.1467 -5.1261
H -7.1123 -0.4093 -5.0678
H -7.1574 2.0679 -1.5571
H -5.6340 -2.0220 -3.8906
H -5.6955 0.4736 -0.4040
H -4.4432 -1.5017 -0.5985
C -0.4581 -7.4334 -4.4098
C -0.4376 -7.0657 -2.9222
C -1.7340 -6.5329 -2.3372
C -1.7104 -5.5544 -1.3344
C -2.9860 -7.0307 -2.7218
C -2.8776 -5.0911 -0.7245
C -4.1603 -6.5853 -2.1233
C -4.1136 -5.6186 -1.1136
O -5.3000 -5.2500 -0.5483
H -0.7626 -6.5797 -5.0235
H -0.1233 -7.9502 -2.3487
H 0.3447 -6.3153 -2.7529
H -0.7593 -5.1440 -1.0076
H -3.0527 -7.7870 -3.4980
H -2.8214 -4.3141 0.0353
H -5.1259 -6.9804 -2.4209
H -5.1522 -4.6008 0.1575
C 4.7134 -5.1400 -5.5408
C 5.0141 -5.1088 -4.0666
O 5.8796 -4.3744 -3.5885
C 4.3938 -3.7329 -6.0626
H 5.6066 -5.5214 -6.0495
H 5.2085 -3.0514 -5.8088
H 3.4749 -3.3461 -5.6090
H 4.2611 -3.7369 -7.1476
N 4.2544 -5.9469 -3.3230
C 4.3261 -5.9674 -1.8837
C 3.1384 -5.2865 -1.2114
C 3.0048 -3.7880 -1.5051
C 1.6628 -3.2984 -0.9711
C 4.1713 -2.9781 -0.9298
H 3.5136 -6.4582 -3.7777
H 5.2616 -5.4794 -1.6104
H 3.2233 -5.4319 -0.1259
H 2.2152 -5.7994 -1.5147
H 3.0080 -3.6497 -2.5954
H 1.5214 -3.5649 0.0812
H 1.5696 -2.2166 -1.0641
H 0.8363 -3.7435 -1.5315
H 5.1185 -3.2453 -1.4045
H 4.2593 -3.1385 0.1513
H 4.0113 -1.9066 -1.0891
C 9.3379 -5.6519 -1.0340
C 8.1990 -4.6215 -0.9469
C 8.6430 -3.1834 -1.1313
C 8.1085 -2.4000 -2.1624
C 9.5873 -2.5998 -0.2718
C 8.5142 -1.0711 -2.3319
C 9.9918 -1.2766 -0.4387
C 9.4590 -0.5013 -1.4744
H 10.1016 -5.4773 -0.2696
H 7.7042 -4.7211 0.0297
H 7.4460 -4.8483 -1.7065
H 7.3813 -2.8397 -2.8380
H 10.0099 -3.1901 0.5373
H 8.1032 -0.4863 -3.1509
H 10.7281 -0.8406 0.2289
H 9.7905 0.5225 -1.6111
C -3.0508 1.3326 0.6719
C -1.8435 1.2576 0.0241
C -1.5629 2.2779 -0.9208
N -0.3791 2.3059 -1.5808
N -2.4725 3.2366 -1.1858
C -3.6301 3.2208 -0.5081
N -3.9516 2.3125 0.4208
S 1.5374 0.8218 -2.8848
C 0.6485 1.2806 -1.3453
C -4.6162 4.3111 -0.8089
N 0.0655 0.0433 -0.8799
C -0.2507 -0.8314 -1.9500
C -1.2575 -1.9094 -1.7148
C 0.4848 -0.6273 -3.0597
C 0.6362 -1.4723 -4.2803
C -0.8086 0.2024 0.2792
H 1.3817 1.6479 -0.6280
H -0.1250 3.1272 -2.1364
H -1.2574 -0.7539 0.5404
H -0.1882 0.4919 1.1316
H -4.7180 4.4411 -1.8893
H -5.5877 4.0832 -0.3740
H -4.2535 5.2633 -0.4058
H -3.3335 0.5851 1.4086
H -1.0451 -2.4421 -0.7842
H -1.2570 -2.6332 -2.5292
H -2.2709 -1.5019 -1.6477
H -0.0450 -2.3254 -4.2448
H 1.6589 -1.8671 -4.3587
C 2.5529 -1.9196 7.2739
C 1.9167 -0.5481 7.5282
C 2.4256 0.4805 6.5415
O 2.0747 0.1825 5.2832
O 3.0868 1.4568 6.8415
C -7.8397 3.1609 3.9545
C -8.2272 2.3433 2.7119
C -7.1710 2.4604 1.6346
O -7.3647 3.0286 0.5619
O -6.0173 1.9254 1.9828
H -8.6066 3.0683 4.7286
H -6.8902 2.8062 4.3618
H -9.1767 2.6826 2.2924
H -8.3261 1.2865 2.9845
C -0.2572 2.5531 4.9030
C -0.2237 2.0830 3.4479
C 1.0967 2.3175 2.7835
C 1.3777 2.8544 1.5512
N 2.2911 1.9273 3.3783
C 3.2573 2.2120 2.5236
N 2.7553 2.7912 1.4051
H 0.5079 2.0568 5.4979
H -0.4600 1.0112 3.4143
H -1.0044 2.5822 2.8644
H 0.7406 3.2963 0.8013
H 4.3082 2.0255 2.6679
H 3.2602 2.7986 0.5246
C 5.7915 7.2775 1.2782
C 5.4773 6.4124 2.5003
C 6.2558 5.0876 2.5684
C 5.9066 4.3246 3.8520
C 6.0167 4.2190 1.3243
H 5.4987 6.7827 0.3467
H 4.4018 6.1847 2.5203
H 5.6826 6.9868 3.4128
H 7.3291 5.3263 2.6019
H 4.8339 4.1036 3.9001
H 6.4495 3.3766 3.8988
H 6.1566 4.9118 4.7422
H 4.9468 4.1523 1.1019
H 6.3920 3.2040 1.4802
H 6.5111 4.6338 0.4420
H 0.4286 -0.9168 -5.2012
H -7.7334 4.2209 3.7111
H 12.3798 4.6024 -1.9024
H 11.7802 5.3351 -3.4074
H -10.2134 2.2006 -4.8003
H -9.6526 0.5192 -4.8223
H -0.0837 3.6318 4.9728
H -1.2317 2.3328 5.3483
H 6.8638 7.4942 1.2140
H 5.2604 8.2337 1.3231
H 0.8281 -0.6168 7.4186
H 2.1399 -0.1869 8.5339
H 2.3784 -2.2469 6.2458
H 2.1461 -2.6723 7.9555
H 3.6360 -1.8789 7.4286
H 8.9521 -6.6667 -0.8959
H 9.8290 -5.6032 -2.0106
H 4.3939 -7.0094 -1.5466
H 0.5348 -7.7560 -4.7405
H -1.1500 -8.2548 -4.6174
H 3.8951 -5.8322 -5.7682
H 2.3153 0.9458 4.6305
H -5.2747 2.1469 1.2961
C 2.0782 6.3128 -4.2063
C 0.9413 5.7662 -3.3589
O 0.9603 4.6303 -2.8763
N -0.0978 6.6324 -3.1856
C -1.4033 6.1306 -2.7888
C -2.1178 5.5887 -4.0347
O -2.0669 6.2176 -5.0920
N -2.7520 4.4127 -3.8693
C -3.4597 3.7722 -4.9664
C -3.6654 2.2792 -4.7257
C -2.3808 1.4463 -4.5661
C -2.7458 -0.0395 -4.5180
C -1.3521 1.7334 -5.6645
H 3.0139 5.8402 -3.9056
H 1.8848 6.0568 -5.2535
H -1.2835 5.3756 -2.0133
H -1.9897 6.9581 -2.3781
H -4.3057 2.1247 -3.8473
H -4.2357 1.8902 -5.5796
H -1.9126 1.7059 -3.6095
H -0.4686 1.1028 -5.5340
H -1.7685 1.5339 -6.6599
H -1.0106 2.7720 -5.6399
H -3.2263 -0.3516 -5.4535
H -3.4452 -0.2492 -3.7060
H -1.8601 -0.6573 -4.3682
H -2.8933 3.9529 -5.8836
C -4.5610 -2.8153 3.9879
C -3.1495 -2.1985 3.9720
O -2.9921 -0.9838 4.0856
C -5.5398 -1.9808 3.1359
C -5.2436 -2.0599 1.6470
N -6.0845 -2.8098 0.8980
O -4.2612 -1.4842 1.1448
N -2.0890 -3.0521 3.8980
C -0.7275 -2.5605 4.0935
C -0.0490 -2.1453 2.7793
O -0.3780 -2.6297 1.6951

C 0.0043 -3.8040 4.6431
C -0.6630 -4.9474 3.8633
C -2.1374 -4.5173 3.7665
N 0.9874 -1.2863 2.9476
C 1.9573 -1.0202 1.9096
C 3.3829 -1.2429 2.4131
O 3.6325 -1.9207 3.4155
N 4.3431 -0.6453 1.6685
C 5.7611 -0.8151 1.9725
C 6.2605 0.1894 3.0464
O 7.0812 1.0799 2.7961
C 6.6364 -0.8416 0.7308
O 6.5324 0.3637 -0.0445
N 5.7284 -0.0396 4.2674
C 6.0833 0.7007 5.4758
H -6.5626 -2.3154 3.3258
H -5.4398 -0.9302 3.4217
H -4.5666 -3.8569 3.6604
H -0.1912 -3.8928 5.7167
H 1.0850 -3.7475 4.4923
H -0.5502 -5.9208 4.3460
H -0.2354 -5.0058 2.8600
H -2.7329 -4.9564 4.5759
H -2.5803 -4.8132 2.8106
H -0.7390 -1.7186 4.7892
H 1.8694 0.0004 1.5264
H 1.7499 -1.6872 1.0724
H 7.6734 -0.9735 1.0488
H 6.3547 -1.7013 0.1162
H 7.2524 0.3107 -0.6944
H 5.8539 -1.8118 2.4196
H 6.9249 1.3513 5.2391
H 2.1675 7.3987 -4.1255
H -4.4353 4.2565 -5.1099
H -2.6723 3.9449 -2.9642
H -0.1474 7.3907 -3.8545
H 6.3780 -0.0001 6.2622
H 5.2400 1.2990 5.8315
H 5.0343 -0.7818 4.3316
H 1.2334 -0.9810 3.8860
H -6.9622 -3.1711 1.2799
H -5.9688 -2.7540 -0.1104
H 4.0751 -0.0267 0.9004
H -4.9006 -2.7982 5.0293
C -10.3179 -4.9341 3.0159
C -9.5161 -4.2983 1.9008
O -8.3958 -3.8234 2.1213
C -10.7509 -3.8377 3.9957
N -10.1238 -4.2055 0.6991
C -9.5192 -3.5078 -0.4221
C -8.4977 7.2531 2.5730
C -8.0717 6.1403 1.6213
C -8.1124 6.5727 0.1550
N -8.1474 5.5364 -0.7292
O -8.1035 7.7483 -0.1896
H -9.8740 -3.3044 4.3700
H -11.2888 -4.2633 4.8470
H -11.4084 -3.1123 3.5065
H -11.1869 -5.4766 2.6277
H -8.6705 -4.0671 -0.8312
H -7.0424 5.8227 1.8358
H -8.6918 5.2472 1.7470
H -8.0388 4.5796 -0.4141
H -8.0671 5.7581 -1.7096
H -9.5475 7.5188 2.4190
H -8.3707 6.9465 3.6159
H -7.9056 8.1527 2.3930
H -9.6701 -5.6568 3.5201
H -9.1600 -2.5256 -0.1042
H -10.2656 -3.3759 -1.2059
H -11.0276 -4.6346 0.5797
O 3.9972 1.1646 -0.6035
H 3.5737 0.8047 -1.4030
H 4.9523 0.9332 -0.6486

TCH$^+$

C 11.5128 4.9718 -2.3944
C 10.4155 3.8896 -2.4819
C 10.0312 3.2388 -1.1768
C 8.7685 3.0112 -0.6636
N 10.9781 2.6968 -0.3245
C 10.2983 2.1672 0.6767
N 8.9537 2.3263 0.5248
H 11.1804 5.8202 -1.7878
H 10.7639 3.1062 -3.1691
H 9.5122 4.3124 -2.9377
H 7.7852 3.2792 -1.0159
H 10.7225 1.6607 1.5321
H 8.2362 1.9503 1.1421
C -9.5877 1.5266 -4.2708
C -8.1465 2.0278 -4.0690
C -7.2481 1.0318 -3.3661
C -6.7704 -0.1039 -4.0356
C -6.8814 1.2014 -2.0266
C -5.9479 -1.0288 -3.4001
C -6.0545 0.2842 -1.3728
C -5.5716 -0.8285 -2.0670
O -4.7394 -1.7594 -1.5006
H -10.0590 1.3092 -3.3070
H -8.1667 2.9596 -3.4925
H -7.7174 2.2793 -5.0474
H -7.0377 -0.2622 -5.0778
H -7.2424 2.0510 -1.4564
H -5.5650 -1.8979 -3.9245
H -5.7991 0.4384 -0.3297
H -4.4546 -1.4853 -0.5923
C -0.4624 -7.3902 -4.4535
C -0.4417 -7.0302 -2.9641
C -1.7409 -6.5048 -2.3767
C -1.7183 -5.5730 -1.3301
C -2.9947 -6.9588 -2.8063
C -2.8876 -5.1138 -0.7217
C -4.1717 -6.5172 -2.2094
C -4.1263 -5.5980 -1.1564
O -5.3155 -5.2334 -0.5953
H -0.7796 -6.5374 -5.0619
H -0.1227 -7.9154 -2.3945
H 0.3376 -6.2768 -2.7923
H -0.7661 -5.1973 -0.9677
H -3.0619 -7.6761 -3.6184
H -2.8306 -4.3713 0.0719
H -5.1388 -6.8785 -2.5427
H -5.1715 -4.6075 0.1320
C 4.7150 -5.1035 -5.5706
C 4.9965 -5.0592 -4.0928
O 5.8433 -4.3083 -3.6071
C 4.4366 -3.6988 -6.1198
H 5.6061 -5.5146 -6.0600
H 5.2605 -3.0311 -5.8594
H 3.5174 -3.2854 -5.6908
H 4.3255 -3.7167 -7.2070
N 4.2466 -5.9113 -3.3556
C 4.3241 -5.9486 -1.9178
C 3.1271 -5.2975 -1.2313
C 2.9620 -3.7984 -1.5070
C 1.6176 -3.3411 -0.9512
C 4.1190 -2.9722 -0.9345
H 3.5238 -6.4426 -3.8163
H 5.2512 -5.4460 -1.6413
H 3.2214 -5.4535 -0.1481
H 2.2120 -5.8243 -1.5350
H 2.9503 -3.6479 -2.5956
H 1.4989 -3.6159 0.1017
H 1.4987 -2.2610 -1.0345
H 0.7919 -3.8031 -1.4982
H 5.0647 -3.2115 -1.4269
H 4.2266 -3.1487 0.1425
H 3.9334 -1.9018 -1.0734
C 9.3363 -5.6494 -1.0643
C 8.1975 -4.6185 -0.9724
C 8.6416 -3.1776 -1.1372
C 8.1059 -2.3796 -2.1568
C 9.5842 -2.6047 -0.2690
C 8.5056 -1.0465 -2.3039
C 9.9855 -1.2779 -0.4162
C 9.4488 -0.4872 -1.4381
H 10.0955 -5.4831 -0.2937
H 7.6979 -4.7296 0.0005
H 7.4478 -4.8363 -1.7382
H 7.3807 -2.8109 -2.8398
H 10.0084 -3.2058 0.5314
H 8.0925 -0.4503 -3.1135
H 10.7204 -0.8508 0.2587
H 9.7788 0.5391 -1.5598
C -3.0776 1.2962 0.7532
C -1.8881 1.2205 0.0841
C -1.6092 2.2551 -0.8547
N -0.4331 2.2845 -1.5137
N -2.5160 3.2289 -1.1040
C -3.6569 3.2369 -0.4161
N -3.9501 2.3067 0.5108
S 1.4714 0.8055 -2.8519
C 0.5938 1.2378 -1.3032
C -4.6452 4.3322 -0.6562
N 0.0001 0.0032 -0.8568
C -0.3067 -0.8688 -1.9328
C -1.3092 -1.9523 -1.7064
C 0.4307 -0.6505 -3.0382
C 0.5918 -1.4819 -4.2672
C -0.8619 0.1484 0.3107
H 1.3264 1.5965 -0.5809
H -0.1907 3.0878 -2.1002
H -1.3205 -0.8064 0.5600
H -0.2306 0.4174 1.1616
H -4.5771 4.6765 -1.6892
H -5.6569 3.9946 -0.4179
H -4.4085 5.1831 -0.0061
H -3.3853 0.5537 1.4835
H -1.1078 -2.4783 -0.7697
H -1.2889 -2.6822 -2.5151
H -2.3275 -1.5541 -1.6605
H -0.0653 -2.3535 -4.2296
H 1.6240 -1.8475 -4.3579
C 2.5565 -1.9433 7.2595
C 1.8989 -0.5795 7.4992
C 2.4013 0.4521 6.5131
O 2.0738 0.1406 5.2513
O 3.0383 1.4440 6.8139
C -7.8226 3.1788 3.9614
C -8.1822 2.4068 2.6829
C -7.0737 2.4861 1.6349
O -7.2934 3.0521 0.5390
O -5.9512 1.9649 1.9707
H -8.6125 3.0828 4.7127
H -6.8914 2.7903 4.3819
H -9.1073 2.7857 2.2405
H -8.3337 1.3494 2.9314
C -0.2419 2.5481 4.9102
C -0.2255 2.0200 3.4743
C 1.0749 2.2804 2.7823
C 1.3263 2.8640 1.5641
N 2.2847 1.8940 3.3445
C 3.2330 2.2233 2.4859
N 2.7003 2.8226 1.3945
H 0.5786 2.1363 5.4957
H -0.4256 0.9414 3.4834
H -1.0347 2.4732 2.8921
H 0.6722 3.3171 0.8364
H 4.2887 2.0478 2.6104
H 3.2035 2.9429 0.5240
C 5.8196 7.2764 1.3122
C 5.8164 6.3537 2.5314
C 6.4728 4.9797 2.3104
C 6.3779 4.1377 3.5883
C 5.8442 4.2452 1.1173
H 5.2214 6.8676 0.4919
H 4.7799 6.1912 2.8605
H 6.3239 6.8529 3.3665
H 7.5373 5.1378 2.0846
H 5.3299 3.9486 3.8527

H 6.8740 3.1717 3.4655
H 6.8366 4.6571 4.4367
H 4.7534 4.2925 1.1916
H 6.1385 3.1910 1.0887
H 6.1271 4.7005 0.1639
H 0.3594 -0.9265 -5.1823
H -7.6826 4.2434 3.7555
H 12.4115 4.5593 -1.9291
H 11.7691 5.3389 -3.3925
H -10.1966 2.2739 -4.7895
H -9.6019 0.6056 -4.8630
H -0.1430 3.6379 4.9259
H -1.1824 2.2830 5.4018
H 6.8373 7.4295 0.9346
H 5.4067 8.2592 1.5607
H 0.8123 -0.6632 7.3795
H 2.1067 -0.2094 8.5050
H 2.3995 -2.2784 6.2310
H 2.1505 -2.6971 7.9402
H 3.6367 -1.8873 7.4278
H 8.9486 -6.6651 -0.9390
H 9.8330 -5.5907 -2.0375
H 4.4125 -6.9929 -1.5931
H 0.5335 -7.6982 -4.7888
H -1.1440 -8.2198 -4.6632
H 3.8845 -5.7806 -5.7992
H 2.3132 0.9013 4.6009
H -4.9213 2.2540 1.1054
C 2.1065 6.3486 -4.1787
C 0.9812 5.7629 -3.3391
O 0.9774 4.5951 -2.9405
N -0.0347 6.6373 -3.0719
C -1.3433 6.1100 -2.7244
C -2.0135 5.5674 -3.9961
O -1.8583 6.1527 -5.0670
N -2.7492 4.4496 -3.8326
C -3.4371 3.8251 -4.9542
C -3.6589 2.3313 -4.7356
C -2.3838 1.4871 -4.5611
C -2.7602 0.0036 -4.5458
C -1.3277 1.7881 -5.6293
H 3.0246 5.7887 -3.9997
H 1.8355 6.2470 -5.2351
H -1.2416 5.3497 -1.9510
H -1.9550 6.9230 -2.3219
H -4.3197 2.1714 -3.8733
H -4.2144 1.9572 -5.6056
H -1.9370 1.7269 -3.5888
H -0.4542 1.1445 -5.4945
H -1.7247 1.6155 -6.6374
H -0.9753 2.8220 -5.5741
H -3.2231 -0.2889 -5.4963
H -3.4789 -0.2133 -3.7528
H -1.8835 -0.6241 -4.3871
H -2.8440 4.0148 -5.8518
C -4.5581 -2.8052 3.9658
C -3.1365 -2.2132 3.9336
O -2.9515 -0.9986 4.0009
C -5.5337 -1.9633 3.1175
C -5.2395 -2.0504 1.6300
N -6.0782 -2.8026 0.8829
O -4.2534 -1.4800 1.1251
N -2.0921 -3.0891 3.9010
C -0.7243 -2.6135 4.0886
C -0.0528 -2.1902 2.7735
O -0.3914 -2.6607 1.6862
C -0.0004 -3.8671 4.6250
C -0.6934 -5.0008 3.8541
C -2.1639 -4.5548 3.7897
N 0.9880 -1.3372 2.9457
C 1.9545 -1.0575 1.9074
C 3.3815 -1.2630 2.4133
O 3.6340 -1.9435 3.4133
N 4.3358 -0.6423 1.6809
C 5.7577 -0.8263 1.9635
C 6.2985 0.1413 3.0522
O 7.2218 0.9317 2.8258
C 6.6097 -0.8158 0.7049
O 6.4881 0.4177 -0.0226
N 5.6959 -0.0143 4.2516
C 6.0939 0.6777 5.4763
H -6.5578 -2.2927 3.3095
H -5.4324 -0.9110 3.3969
H -4.5804 -3.8489 3.6452
H -0.1810 -3.9560 5.7012
H 1.0786 -3.8233 4.4592
H -0.5802 -5.9777 4.3295
H -0.2871 -5.0577 2.8417
H -2.7442 -4.9767 4.6194
H -2.6353 -4.8568 2.8496
H -0.7205 -1.7767 4.7909
H 1.8533 -0.0375 1.5263
H 1.7581 -1.7290 1.0711
H 7.6514 -0.9571 0.9988
H 6.3158 -1.6536 0.0666
H 7.2180 0.4189 -0.6627
H 5.8516 -1.8339 2.3871
H 6.8930 1.3752 5.2272
H 2.2671 7.4079 -3.9635
H -4.4056 4.3176 -5.1141
H -2.7239 3.9848 -2.9265
H -0.0791 7.4372 -3.6907
H 6.4643 -0.0431 6.2128
H 5.2456 1.2203 5.9007
H 4.9699 -0.7260 4.3076
H 1.2385 -1.0433 3.8865
H -6.9595 -3.1593 1.2628
H -5.9637 -2.7454 -0.1258
H 4.0613 -0.0460 0.8970
H -4.8867 -2.7793 5.0108
C -10.3342 -4.9077 2.9765
C -9.5214 -4.2780 1.8654
O -8.3874 -3.8362 2.0850
C -10.7504 -3.8130 3.9657
N -10.1319 -4.1576 0.6676
C -9.5160 -3.4634 -0.4498
C -8.4701 7.2795 2.6006
C -8.0881 6.1403 1.6625
C -8.2709 6.5018 0.1876
N -8.3402 5.4219 -0.6372
O -8.3413 7.6611 -0.2074
H -9.8658 -3.2961 4.3447
H -11.2950 -4.2377 4.8132
H -11.3963 -3.0730 3.4830
H -11.2119 -5.4327 2.5837
H -8.6678 -4.0289 -0.8512
H -7.0350 5.8613 1.8009
H -8.6623 5.2351 1.8800
H -8.1497 4.4861 -0.2821
H -8.3605 5.5988 -1.6298
H -9.5384 7.5040 2.5282
H -8.2437 7.0232 3.6402
H -7.9297 8.1901 2.3330
H -9.6987 -5.6453 3.4746
H -9.1539 -2.4816 -0.1332
H -10.2560 -3.3302 -1.2392
H -11.0513 -4.5531 0.5518
O 3.9279 1.1219 -0.6092
H 3.5198 0.7394 -1.4042
H 4.8912 0.9348 -0.6509

Benzoylformate Model D

AP

C 11.4501 5.3380 -0.0942
C 11.1173 4.0900 -0.9240
C 9.8670 3.4215 -0.4453
C 8.6116 3.4387 -1.0112
N 9.8340 2.7294 0.7517
C 8.5778 2.3359 0.8968
N 7.7980 2.7489 -0.1399
H 10.6513 6.0821 -0.1793
H 11.9523 3.3813 -0.8618
H 11.0066 4.3616 -1.9811
H 8.2344 3.8788 -1.9219
H 8.1738 1.7314 1.6981
H 6.8270 2.4659 -0.2890
C -9.3408 2.3192 -4.4820
C -8.4081 2.9808 -3.4546
C -7.3974 1.9927 -2.9258
C -6.3840 1.5117 -3.7661
C -7.4764 1.4717 -1.6303
C -5.4983 0.5297 -3.3423
C -6.5941 0.4861 -1.1864
C -5.6054 0.0018 -2.0509
O -4.7445 -0.9936 -1.7135
H -9.8803 1.4819 -4.0274
H -8.9968 3.3916 -2.6265
H -7.8961 3.8290 -3.9271
H -6.2860 1.9167 -4.7702
H -8.2329 1.8523 -0.9485
H -4.7092 0.1622 -3.9887
H -6.6541 0.1142 -0.1708
H -4.6454 -1.1049 -0.7273
C -0.1297 -6.4752 -5.2736
C -0.3086 -6.3219 -3.7596
C -1.6894 -5.9042 -3.2798
C -1.9291 -5.7738 -1.9040
C -2.7532 -5.6226 -4.1458
C -3.1695 -5.3839 -1.4072
C -4.0037 -5.2318 -3.6656
C -4.2203 -5.1137 -2.2916
O -5.4692 -4.7516 -1.8686
H -0.3234 -5.5342 -5.7997
H -0.0424 -7.2694 -3.2734
H 0.4247 -5.5945 -3.3857
H -1.1262 -5.9751 -1.2017
H -2.6132 -5.7050 -5.2184
H -3.3151 -5.2719 -0.3355
H -4.8202 -5.0128 -4.3452
H -5.4598 -4.5889 -0.9123
C 5.1010 -4.0526 -5.5087
C 5.2908 -4.8912 -4.2668
O 6.3433 -5.4781 -4.0257
C 5.8464 -2.7168 -5.3898
H 5.4992 -4.6234 -6.3531
H 6.9097 -2.8873 -5.2073
H 5.4532 -2.1290 -4.5556
H 5.7391 -2.1278 -6.3056
N 4.2287 -4.9122 -3.4197
C 4.3820 -5.4878 -2.0980
C 3.0783 -5.4977 -1.3136
C 2.4196 -4.1217 -1.1056
C 1.2493 -4.2789 -0.1303
C 3.4245 -3.0657 -0.6234
H 3.4683 -4.2685 -3.5765
H 5.1466 -4.9355 -1.5420
H 3.2955 -5.9276 -0.3281
H 2.3570 -6.1707 -1.7964
H 2.0022 -3.7873 -2.0703
H 1.6189 -4.4233 0.8909
H 0.5880 -3.4131 -0.1159
H 0.6366 -5.1474 -0.3946
H 4.2180 -2.8795 -1.3538
H 3.8970 -3.3904 0.3088
H 2.9473 -2.1037 -0.4237
C 9.2896 -5.3498 -0.7407
C 7.9342 -4.6318 -0.7399
C 8.0280 -3.1473 -1.0242
C 7.3961 -2.6018 -2.1475
C 8.7397 -2.2843 -0.1798
C 7.4604 -1.2320 -2.4120
C 8.8081 -0.9161 -0.4406
C 8.1617 -0.3825 -1.5574
H 9.9657 -4.9327 0.0125
H 7.4471 -4.7845 0.2330
H 7.2956 -5.0973 -1.4959
H 6.8742 -3.2699 -2.8222

H	9.2318	-2.6842	0.7029
H	6.9744	-0.8285	-3.2964
H	9.3505	-0.2564	0.2283
H	8.2108	0.6829	-1.7508
C	-2.6500	0.7384	0.5010
C	-1.4008	0.9913	-0.0291
C	-1.2191	2.2515	-0.6641
N	0.0041	2.7011	-1.0291
N	-2.3010	3.0234	-0.9193
C	-3.4892	2.6602	-0.4238
N	-3.6969	1.5764	0.3437
S	1.6072	-0.8906	-3.2029
C	1.3112	-0.1499	-1.7068
C	-4.6730	3.5228	-0.7398
N	0.1739	-0.5522	-1.1662
C	-0.5380	-1.5015	-1.9160
C	-1.8383	-2.0389	-1.4375
C	0.1134	-1.7963	-3.0794
C	-0.3333	-2.6796	-4.1987
C	-0.2812	0.0128	0.1414
H	2.0293	0.4579	-1.1478
H	0.0481	3.6244	-1.4623
H	-0.5892	-0.8308	0.7588
H	0.5977	0.4770	0.5853
H	-5.3424	3.5587	0.1198
H	-4.3580	4.5266	-1.0237
H	-5.2292	3.0853	-1.5755
H	-2.8404	-0.1583	1.0834
H	-1.8019	-2.2888	-0.3747
H	-2.0895	-2.9448	-1.9875
H	-2.6388	-1.3183	-1.6097
H	-1.1080	-3.3683	-3.8600
H	0.4940	-3.2751	-4.5965
C	1.7199	-3.0460	7.4069
C	0.8596	-1.8066	7.1048
C	1.6791	-0.6703	6.4605
O	1.7980	-0.7174	5.1682
O	2.1842	0.1855	7.1950
C	-8.3697	2.5845	4.0389
C	-7.3961	1.5088	3.5584
C	-6.5261	1.9382	2.3896
O	-6.5851	3.0554	1.8846
O	-5.7079	0.9813	1.9956
H	-8.9581	2.2132	4.8833
H	-7.8361	3.4805	4.3615
H	-7.9246	0.5967	3.2544
H	-6.7157	1.1958	4.3592
C	-0.9026	1.7807	5.5771
C	-1.0469	1.2731	4.1436
C	0.0510	1.7800	3.2771
C	0.0493	2.6407	2.2064
N	1.3762	1.4308	3.4564
C	2.0967	2.0472	2.4785
N	1.3238	2.7835	1.6895
H	0.0472	1.4696	6.0199
H	-1.0648	0.1784	4.1313
H	-2.0026	1.5948	3.7159
H	-0.7978	3.1626	1.7857
H	3.1612	1.9211	2.3708
C	5.4090	7.0180	3.3908
C	5.1126	5.6500	4.0170
C	5.5774	4.4342	3.1929
C	5.3347	3.1365	3.9781
C	4.9019	4.3728	1.8177
H	4.8726	7.1540	2.4466
H	4.0315	5.5570	4.1927
H	5.5857	5.6019	5.0071
H	6.6619	4.5318	3.0306
H	4.2698	3.0205	4.2124
H	5.6548	2.2526	3.4170
H	5.8766	3.1404	4.9307
H	3.8108	4.3294	1.9033
H	5.2238	3.4839	1.2713
H	5.1506	5.2395	1.1971
H	-0.7460	-2.0851	-5.0215
H	-9.0565	2.8746	3.2421
H	11.5411	5.0679	0.9615

H	12.3894	5.8018	-0.4179
H	-10.0770	3.0300	-4.8726
H	-8.7695	1.9236	-5.3273
H	-0.9386	2.8747	5.6027
H	-1.7095	1.3981	6.2121
H	6.4794	7.1307	3.1814
H	5.1120	7.8325	4.0609
H	0.0348	-2.0868	6.4402
H	0.4288	-1.4197	8.0331
H	2.1757	-3.4281	6.4874
H	1.1236	-3.8465	7.8583
H	2.5278	-2.7862	8.0976
H	9.1665	-6.4170	-0.5282
H	9.7758	-5.2507	-1.7163
H	4.7661	-6.5051	-2.2168
H	0.8948	-6.7807	-5.5034
H	-0.8069	-7.2314	-5.6835
H	4.0361	-3.8765	-5.7045
H	-5.0172	1.2868	1.2952
C	2.2345	7.0214	-2.5014
C	0.9204	6.3397	-2.1945
O	0.7077	5.1595	-2.4926
N	-0.0198	7.0889	-1.5652
C	-1.3702	6.5713	-1.4382
C	-1.9898	6.3266	-2.8262
O	-1.9143	7.1748	-3.7113
N	-2.6141	5.1360	-2.9416
C	-3.1820	4.6787	-4.1980
C	-3.1085	3.1555	-4.3031
C	-1.6864	2.5736	-4.3379
C	-1.7297	1.0585	-4.1218
C	-0.9559	2.9221	-5.6384
C	3.6383	2.1782	-2.8320
C	2.8332	2.9152	-3.7134
C	2.8203	2.5882	-5.0667
C	3.6074	1.5363	-5.5457
C	4.4132	0.8074	-4.6691
C	4.4260	1.1195	-3.3098
C	3.6466	2.5468	-1.3835
O	3.2414	3.6293	-0.9884
C	4.1583	1.4679	-0.4188
O	5.4059	1.4289	-0.2595
O	3.3039	0.6999	0.0879
H	3.0357	6.3896	-2.1088
H	2.3534	7.0815	-3.5870
H	-1.3606	5.6590	-0.8369
H	-1.9722	7.3175	-0.9138
H	-3.6566	2.7193	-3.4616
H	-3.6490	2.8438	-5.2075
H	-1.1128	3.0101	-3.5136
H	0.0409	2.4724	-5.6490
H	-1.5058	2.5459	-6.5108
H	-0.8329	4.0030	-5.7545
H	-2.3085	0.5627	-4.9123
H	-2.1929	0.8057	-3.1631
H	-0.7217	0.6368	-4.1343
H	-2.6436	5.1728	-5.0125
H	2.2242	3.7251	-3.3210
H	2.2004	3.1578	-5.7516
H	3.5979	1.2901	-6.6037
H	5.0372	0.0044	-5.0452
H	5.0660	0.5715	-2.6249
C	-5.0462	-3.3290	3.3639
C	-3.6732	-2.6913	3.6269
O	-3.5712	-1.4902	3.8684
C	-5.9752	-2.3924	2.5759
C	-5.5545	-2.2457	1.1299
N	-6.3348	-2.8290	0.1877
O	-4.5281	-1.6235	0.8158
N	-2.6075	-3.5331	3.6634
C	-1.2788	-3.0384	4.0057
C	-0.5483	-2.4699	2.7820
O	-0.9405	-2.7223	1.6299
C	-0.5460	-4.3163	4.4613
C	-1.1178	-5.3718	3.5017
C	-2.5921	-4.9621	3.3247
N	0.5633	-1.7921	3.0856

C	1.6364	-1.5526	2.1461
C	2.9220	-2.2054	2.6552
O	2.9387	-3.3698	3.0586
N	3.9993	-1.3929	2.5952
C	5.3449	-1.8016	2.9490
C	5.8101	-1.3750	4.3558
O	6.9638	-1.6387	4.6985
C	6.3267	-1.2050	1.9337
O	6.2293	0.2085	1.9678
N	4.9430	-0.6924	5.1282
C	5.3754	-0.1783	6.4191
H	-7.0037	-2.7595	2.6296
H	-5.9087	-1.3924	3.0120
H	-4.9626	-4.2943	2.8588
H	-0.8109	-4.5389	5.5000
H	0.5385	-4.2150	4.3867
H	-1.0172	-6.3941	3.8742
H	-0.6024	-5.3048	2.5405
H	-3.2494	-5.5253	3.9994
H	-2.9329	-5.1280	2.2968
H	-1.3535	-2.2790	4.7857
H	1.7933	-0.4872	1.9905
H	1.3751	-2.0100	1.1947
H	7.3334	-1.5193	2.2194
H	6.1063	-1.5942	0.9327
H	5.9337	0.5435	1.0925
H	5.3866	-2.8944	2.9165
H	6.1841	0.5474	6.2842
H	2.3122	8.0233	-2.0736
H	-4.2307	4.9977	-4.2777
H	-2.4585	4.4380	-2.2095
H	0.1061	8.0887	-1.5495
H	5.7552	-0.9864	7.0538
H	4.5147	0.2924	6.8947
H	3.9609	-0.6159	4.8797
H	0.8342	-1.5598	4.0650
H	-7.2390	-3.2471	0.4146
H	-6.1165	-2.6177	-0.7803
H	3.9135	-0.4917	2.1362
H	-5.4956	-3.5253	4.3448
C	-10.7836	-5.2204	1.4624
C	-9.8369	-4.4602	0.5582
O	-8.7013	-4.1599	0.9415
C	-11.1991	-4.3026	2.6174
N	-10.3286	-4.0693	-0.6372
C	-9.5637	-3.2332	-1.5449
C	-8.9377	6.8555	3.3167
C	-8.7683	5.6314	2.4237
C	-8.6670	5.9616	0.9361
N	-8.1245	4.9544	0.1848
O	-9.0846	7.0064	0.4508
H	-10.3128	-3.9479	3.1483
H	-11.8415	-4.8309	3.3270
H	-11.7468	-3.4301	2.2476
H	-11.6607	-5.5815	0.9141
H	-8.6662	-3.7529	-1.8969
H	-7.8950	5.0422	2.7125
H	-9.6325	4.9637	2.5385
H	-7.5922	4.2172	0.6334
H	-7.9340	5.1617	-0.7844
H	-9.7825	7.4614	2.9805
H	-9.1058	6.5562	4.3557
H	-8.0477	7.4912	3.2850
H	-10.2467	-6.0922	1.8470
H	-9.2497	-2.3082	-1.0519
H	-10.1841	-2.9811	-2.4053
H	-11.2620	-4.3484	-0.8933
H	0.7676	2.5542	-0.3585
H	1.7116	0.7594	4.1762

APH^+

C	11.4421	5.3589	-0.2237
C	11.1018	4.1171	-1.0592
C	9.8580	3.4467	-0.5679
C	8.6037	3.4416	-1.1348
N	9.8298	2.7800	0.6436

C	8.5765	2.3808	0.7976
N	7.7954	2.7641	-0.2496
H	10.6425	6.1034	-0.2966
H	11.9389	3.4094	-1.0143
H	10.9762	4.3967	-2.1124
H	8.2246	3.8576	-2.0560
H	8.1770	1.7898	1.6112
H	6.8221	2.4886	-0.3839
C	-9.3651	2.2738	-4.4858
C	-8.2926	2.9357	-3.6053
C	-7.3209	1.9146	-3.0636
C	-6.4382	1.2588	-3.9333
C	-7.3095	1.5441	-1.7145
C	-5.5895	0.2562	-3.4821
C	-6.4635	0.5391	-1.2409
C	-5.6065	-0.1150	-2.1330
O	-4.7773	-1.1277	-1.7554
H	-9.9273	1.5303	-3.9116
H	-8.7702	3.4676	-2.7744
H	-7.7584	3.6921	-4.1950
H	-6.4132	1.5432	-4.9826
H	-7.9492	2.0598	-1.0036
H	-4.8994	-0.2457	-4.1513
H	-6.4425	0.3058	-0.1813
H	-4.6658	-1.1752	-0.7660
C	-0.1529	-6.5259	-5.2009
C	-0.3668	-6.4464	-3.6853
C	-1.7468	-6.0121	-3.2170
C	-1.9621	-5.7559	-1.8549
C	-2.8384	-5.8521	-4.0794
C	-3.2060	-5.3637	-1.3670
C	-4.0919	-5.4610	-3.6081
C	-4.2847	-5.2167	-2.2469
O	-5.5346	-4.8533	-1.8311
H	-0.3539	-5.5643	-5.6855
H	-0.1391	-7.4270	-3.2461
H	0.3765	-5.7622	-3.2549
H	-1.1370	-5.8531	-1.1567
H	-2.7184	-6.0314	-5.1427
H	-3.3300	-5.1467	-0.3088
H	-4.9298	-5.3374	-4.2856
H	-5.5146	-4.6220	-0.8889
C	5.0755	-4.1042	-5.4891
C	5.2670	-4.9199	-4.2325
O	6.3303	-5.4791	-3.9742
C	5.9044	-2.8146	-5.4339
H	5.4066	-4.7185	-6.3327
H	6.9593	-3.0476	-5.2743
H	5.5728	-2.1805	-4.6068
H	5.8064	-2.2465	-6.3637
N	4.1993	-4.9528	-3.3915
C	4.3713	-5.4952	-2.0571
C	3.0740	-5.5354	-1.2662
C	2.3797	-4.1772	-1.0622
C	1.2350	-4.3582	-0.0602
C	3.3659	-3.0894	-0.6132
H	3.4340	-4.3163	-3.5550
H	5.1222	-4.9104	-1.5156
H	3.3095	-5.9529	-0.2795
H	2.3688	-6.2319	-1.7394
H	1.9359	-3.8676	-2.0235
H	1.6304	-4.4481	0.9577
H	0.5281	-3.5288	-0.0623
H	0.6685	-5.2701	-0.2794
H	4.1394	-2.8922	-1.3618
H	3.8655	-3.3899	0.3128
H	2.8696	-2.1374	-0.4151
C	9.2845	-5.3374	-0.7220
C	7.9315	-4.6141	-0.7001
C	8.0255	-3.1317	-0.9966
C	7.3846	-2.5917	-2.1176
C	8.7503	-2.2655	-0.1662
C	7.4508	-1.2236	-2.3913
C	8.8212	-0.8995	-0.4369
C	8.1637	-0.3708	-1.5496
H	9.9770	-4.9163	0.0137
H	7.4620	-4.7572	0.2828
H	7.2775	-5.0831	-1.4405
H	6.8558	-3.2629	-2.7839
H	9.2511	-2.6616	0.7133
H	6.9578	-0.8237	-3.2735
H	9.3731	-0.2371	0.2215
H	8.2154	0.6930	-1.7512
C	-2.6883	0.7720	0.6015
C	-1.4525	1.0202	0.0560
C	-1.2767	2.2811	-0.5990
N	-0.0674	2.7315	-0.9699
N	-2.3623	3.0530	-0.8635
C	-3.5402	2.7274	-0.3438
N	-3.7151	1.6419	0.4388
S	1.5542	-0.9187	-3.0937
C	1.2585	-0.1498	-1.6131
C	-4.7235	3.5955	-0.6234
N	0.1219	-0.5470	-1.0614
C	-0.5823	-1.5186	-1.7891
C	-1.8847	-2.0529	-1.3169
C	0.0728	-1.8371	-2.9447
C	-0.3705	-2.7597	-4.0338
C	-0.3324	0.0387	0.2346
H	1.9874	0.4567	-1.0663
H	-0.0128	3.6426	-1.4311
H	-0.6466	-0.7884	0.8700
H	0.5435	0.5119	0.6753
H	-5.4249	3.5852	0.2153
H	-4.3985	4.6091	-0.8556
H	-5.2609	3.1945	-1.4897
H	-2.9082	-0.1068	1.1982
H	-1.9031	-2.1873	-0.2347
H	-2.0631	-3.0271	-1.7705
H	-2.7033	-1.4004	-1.6260
H	-1.1022	-3.4756	-3.6565
H	0.4693	-3.3259	-4.4473
C	1.7473	-2.9312	7.4261
C	0.9026	-1.6773	7.1379
C	1.7111	-0.5661	6.4385
O	1.8276	-0.6790	5.1491
O	2.2042	0.3364	7.1224
C	-8.3590	2.6504	4.0269
C	-7.1276	1.8007	3.7087
C	-6.4709	2.0830	2.3440
O	-6.9091	3.0153	1.6323
O	-5.6899	1.1433	1.9278
H	-8.7607	2.3695	5.0055
H	-8.1108	3.7076	4.0533
H	-7.3404	0.7299	3.7458
H	-6.3594	1.9729	4.4748
C	-0.8852	1.8702	5.5446
C	-1.0163	1.2700	4.1470
C	0.0616	1.7769	3.2572
C	0.0415	2.6634	2.2084
N	1.3895	1.4251	3.4064
C	2.0937	2.0634	2.4314
N	1.3069	2.8168	1.6717
H	0.0738	1.6109	6.0017
H	-0.9829	0.1777	4.1988
H	-1.9866	1.5248	3.7074
H	-0.8103	3.2042	1.8222
H	3.1554	1.9377	2.3001
C	5.4146	7.0814	3.2642
C	5.1150	5.7212	3.9072
C	5.5628	4.4878	3.0983
C	5.3256	3.2071	3.9137
C	4.8703	4.3990	1.7322
H	4.8789	7.2066	2.3180
H	4.0349	5.6384	4.0945
H	5.5968	5.6827	4.8935
H	6.6454	4.5761	2.9190
H	4.2667	3.1085	4.1824
H	5.6220	2.3084	3.3628
H	5.8941	3.2228	4.8505
H	3.7801	4.3630	1.8322
H	5.1821	3.4945	1.2037
H	5.1167	5.2507	1.0900
H	-0.8361	-2.2002	-4.8528
H	-9.1415	2.5111	3.2786
H	11.5417	5.0807	0.8291
H	12.3785	5.8250	-0.5522
H	-10.0738	3.0118	-4.8767
H	-8.9091	1.7585	-5.3369
H	-0.9507	2.9621	5.5007
H	-1.6805	1.5077	6.2054
H	6.4853	7.1896	3.0539
H	5.1185	7.9043	3.9241
H	0.0462	-1.9502	6.5101
H	0.5157	-1.2666	8.0751
H	2.1653	-3.3326	6.4970
H	1.1493	-3.7133	7.9062
H	2.5829	-2.6827	8.0876
H	9.1621	-6.4023	-0.4980
H	9.7511	-5.2483	-1.7081
H	4.7863	-6.5020	-2.1596
H	0.8810	-6.8052	-5.4223
H	-0.8063	-7.2733	-5.6619
H	4.0163	-3.8701	-5.6519
H	-4.6909	1.4485	1.0275
C	2.2159	7.0067	-2.6145
C	0.9072	6.3294	-2.2685
O	0.7025	5.1331	-2.4985
N	-0.0348	7.1007	-1.6672
C	-1.3848	6.5839	-1.5346
C	-1.9868	6.2955	-2.9224
O	-1.8448	7.0886	-3.8483
N	-2.6663	5.1302	-2.9994
C	-3.2064	4.6396	-4.2580
C	-3.1978	3.1112	-4.2970
C	-1.8035	2.4683	-4.2446
C	-1.9206	0.9625	-3.9971
C	-0.9915	2.7511	-5.5127
C	3.6332	2.0686	-2.8636
C	2.8397	2.7857	-3.7718
C	2.8445	2.4280	-5.1173
C	3.6373	1.3646	-5.5616
C	4.4333	0.6574	-4.6590
C	4.4296	1.0015	-3.3075
C	3.6098	2.4577	-1.4219
O	3.1555	3.5275	-1.0440
C	4.1575	1.4190	-0.4342
O	5.4055	1.4297	-0.2911
O	3.3315	0.6456	0.1151
H	3.0229	6.4286	-2.1563
H	2.3526	6.9709	-3.6987
H	-1.3789	5.6910	-0.9049
H	-1.9942	7.3429	-1.0373
H	-3.8044	2.7320	-3.4666
H	-3.7125	2.7855	-5.2107
H	-1.2525	2.9047	-3.4054
H	-0.0139	2.2646	-5.4576
H	-1.5098	2.3710	-6.4024
H	-0.8201	3.8224	-5.6516
H	-2.4759	0.4719	-4.8064
H	-2.4490	0.7482	-3.0628
H	-0.9313	0.5015	-3.9463
H	-2.6171	5.0770	-5.0689
H	2.2296	3.6066	-3.4046
H	2.2346	2.9818	-5.8237
H	3.6405	1.0937	-6.6136
H	5.0630	-0.1534	-5.0091
H	5.0641	0.4719	-2.6032
C	-5.0353	-3.2698	3.4151
C	-3.6380	-2.6676	3.6410
O	-3.4911	-1.4597	3.8190
C	-5.9349	-2.3200	2.6037
C	-5.5283	-2.2240	1.1494
N	-6.2995	-2.8633	0.2351
O	-4.5187	-1.5900	0.7987
N	-2.5952	-3.5356	3.7183
C	-1.2588	-3.0516	4.0483
C	-0.5277	-2.5003	2.8170
O	-0.9245	-2.7556	1.6671
C	-0.5363	-4.3291	4.5218
C	-1.1406	-5.4003	3.6008

C -2.6143 -4.9800 3.4558
N 0.5907 -1.8281 3.1098
C 1.6510 -1.5868 2.1563
C 2.9530 -2.2057 2.6690
O 2.9884 -3.3586 3.1024
N 4.0188 -1.3794 2.5809
C 5.3696 -1.7634 2.9466
C 5.8319 -1.3028 4.3438
O 6.9890 -1.5486 4.6878
C 6.3517 -1.1842 1.9223
O 6.2729 0.2307 1.9427
N 4.9617 -0.6118 5.1052
C 5.3972 -0.0749 6.3860
H -6.9747 -2.6503 2.6738
H -5.8335 -1.3071 3.0050
H -4.9903 -4.2544 2.9430
H -0.7844 -4.5228 5.5704
H 0.5480 -4.2433 4.4264
H -1.0383 -6.4157 3.9911
H -0.6511 -5.3568 2.6244
H -3.2533 -5.4972 4.1833
H -2.9969 -5.1956 2.4524
H -1.3215 -2.2804 4.8181
H 1.7874 -0.5226 1.9751
H 1.3856 -2.0675 1.2175
H 7.3562 -1.5057 2.2074
H 6.1243 -1.5796 0.9252
H 5.9602 0.5560 1.0716
H 5.4222 -2.8562 2.9401
H 6.3052 0.5176 6.2460
H 2.2679 8.0438 -2.2770
H -4.2357 4.9994 -4.3914
H -2.5558 4.4621 -2.2372
H 0.0803 8.1011 -1.7129
H 5.6260 -0.8761 7.0992
H 4.5873 0.5366 6.7834
H 3.9725 -0.5727 4.8741
H 0.8656 -1.5801 4.0832
H -7.2075 -3.2613 0.4820
H -6.1086 -2.6538 -0.7394
H 3.9183 -0.4961 2.0902
H -5.4814 -3.4134 4.4063
C -10.7717 -5.1899 1.5659
C -9.8286 -4.4440 0.6453
O -8.6862 -4.1498 1.0126
C -11.1907 -4.2558 2.7069
N -10.3306 -4.0639 -0.5501
C -9.5731 -3.2400 -1.4758
C -8.9321 6.9114 3.2515
C -8.6378 5.7674 2.2878
C -8.4220 6.2142 0.8418
N -7.9726 5.2079 0.0323
O -8.6510 7.3568 0.4425
H -10.3061 -3.8866 3.2307
H -11.8285 -4.7771 3.4258
H -11.7443 -3.3926 2.3245
H -11.6481 -5.5640 1.0249
H -8.6795 -3.7663 -1.8279
H -7.7530 5.2027 2.5927
H -9.4569 5.0392 2.2925
H -7.6493 4.3406 0.4477
H -7.7012 5.4539 -0.9052
H -9.8010 7.4842 2.9185
H -9.1257 6.5272 4.2570
H -8.0891 7.6051 3.3053
H -10.2310 -6.0532 1.9640
H -9.2543 -2.3085 -0.9983
H -10.2006 -3.0000 -2.3344
H -11.2733 -4.3287 -0.7862
H 0.7223 2.5657 -0.3328
H 1.7355 0.7478 4.1193

IP

C 11.4745 5.2491 -0.5542
C 11.0395 4.0006 -1.3344
C 9.8386 3.3377 -0.7329
C 8.5601 3.2468 -1.2379
N 9.8973 2.7288 0.5079
C 8.6748 2.2766 0.7369
N 7.8264 2.5719 -0.2867
H 10.6853 6.0081 -0.5662
H 11.8711 3.2848 -1.3558
H 10.8232 4.2650 -2.3767
H 8.1194 3.5988 -2.1583
H 8.3461 1.7067 1.5959
H 6.8681 2.2259 -0.3663
C -9.4427 2.3967 -4.4220
C -8.1971 2.9033 -3.6769
C -7.3096 1.7810 -3.1866
C -6.5489 1.0241 -4.0890
C -7.2356 1.4477 -1.8297
C -5.7469 -0.0276 -3.6558
C -6.4280 0.4038 -1.3749
C -5.6787 -0.3384 -2.2924
O -4.8784 -1.3845 -1.9250
H -10.0514 1.7651 -3.7667
H -8.5097 3.5168 -2.8241
H -7.6268 3.5636 -4.3437
H -6.5751 1.2683 -5.1488
H -7.7895 2.0069 -1.0796
H -5.1498 -0.6060 -4.3531
H -6.3695 0.2212 -0.3056
H -4.7177 -1.3988 -0.9464
C -0.3575 -6.5269 -5.2229
C -0.4230 -6.2001 -3.7256
C -1.7849 -5.7848 -3.1975
C -2.0821 -5.9352 -1.8363
C -2.7758 -5.2193 -4.0127
C -3.3120 -5.5513 -1.3064
C -4.0117 -4.8299 -3.4998
C -4.2911 -5.0046 -2.1426
O -5.5381 -4.6682 -1.7023
H -0.5612 -5.6459 -5.8400
H -0.0831 -7.0718 -3.1537
H 0.3063 -5.4080 -3.5026
H -1.3384 -6.3686 -1.1735
H -2.5896 -5.0808 -5.0728
H -3.5133 -5.6805 -0.2460
H -4.7696 -4.3852 -4.1348
H -5.5543 -4.6280 -0.7342
C 4.8954 -4.1754 -5.6242
C 5.1305 -4.9627 -4.3577
O 6.2075 -5.4991 -4.1016
C 5.4985 -2.7664 -5.5227
H 5.3683 -4.7215 -6.4455
H 6.5784 -2.8249 -5.3688
H 5.0664 -2.2150 -4.6823
H 5.3097 -2.1971 -6.4377
N 4.0793 -4.9866 -3.5023
C 4.2344 -5.5279 -2.1683
C 2.9337 -5.4678 -1.3796
C 2.3362 -4.0617 -1.1943
C 1.1097 -4.1618 -0.2876
C 3.3610 -3.0570 -0.6516
H 3.2662 -4.4261 -3.7041
H 5.0212 -4.9810 -1.6402
H 3.1313 -5.8919 -0.3874
H 2.1845 -6.1169 -1.8522
H 1.9860 -3.6922 -2.1727
H 1.3990 -4.4649 0.7248
H 0.5827 -3.2147 -0.1977
H 0.3892 -4.8933 -0.6669
H 4.1979 -2.9035 -1.3403
H 3.7751 -3.4051 0.3012
H 2.9131 -2.0767 -0.4723
C 9.1723 -5.4223 -0.9215
C 7.8212 -4.6960 -0.9311
C 7.9374 -3.2218 -1.2532
C 7.4288 -2.7150 -2.4543
C 8.5689 -2.3342 -0.3712
C 7.5358 -1.3561 -2.7589
C 8.6781 -0.9774 -0.6710
C 8.1563 -0.4811 -1.8679
H 9.8551 -4.9883 -0.1837
H 7.3394 -4.8181 0.0484
H 7.1760 -5.1733 -1.6732
H 6.9750 -3.4066 -3.1543
H 8.9692 -2.7085 0.5673
H 7.1461 -0.9835 -3.7024
H 9.1617 -0.3004 0.0246
H 8.2421 0.5758 -2.0948
C -2.8677 0.7102 0.6451
C -1.6629 0.9257 0.0604
C -1.4285 2.1780 -0.6552
N -0.2425 2.4519 -1.0994
N -2.5298 3.0398 -0.8064
C -3.6660 2.7580 -0.2186
N -3.8666 1.6338 0.5216
S 1.4900 -0.5505 -3.0829
C 1.0811 0.0603 -1.5547
C -4.8245 3.7032 -0.3201
N 0.0080 -0.5193 -1.0511
C -0.5853 -1.4879 -1.8734
C -1.8313 -2.1825 -1.4499
C 0.1081 -1.6341 -3.0395
C -0.2092 -2.5053 -4.2113
C -0.5446 -0.0438 0.2482
H 1.6482 0.7823 -0.9976
H -0.2038 3.3568 -1.5736
H -0.8546 -0.9223 0.8123
H 0.2925 0.4327 0.7575
H -5.7300 3.1665 -0.6075
H -5.0263 4.1651 0.6513
H -4.6037 4.4890 -1.0418
H -3.1131 -0.1737 1.2224
H -1.7942 -2.4735 -0.3980
H -1.9805 -3.0820 -2.0448
H -2.6971 -1.5367 -1.6051
H -1.0124 -3.1981 -3.9582
H 0.6601 -3.0917 -4.5277
C 1.8120 -2.8502 7.3370
C 1.0839 -1.5210 7.5807
C 1.6969 -0.3649 6.8122
O 1.5731 -0.5206 5.4892
O 2.2260 0.6001 7.3314
C -8.2808 2.8322 4.0679
C -6.8917 2.5654 3.4812
C -6.9157 2.0067 2.0455
O -7.6721 2.5667 1.2127
O -6.1259 1.0373 1.7983
H -8.2293 3.4437 4.9749
H -8.9071 3.3394 3.3346
H -6.3120 1.8888 4.1147
H -6.3287 3.5088 3.4306
C -0.7921 1.9686 5.4605
C -0.7288 1.4445 4.0214
C 0.5391 1.8252 3.3241
C 0.7150 2.5660 2.1808
N 1.7894 1.4315 3.7910
C 2.6834 1.9162 2.9378
N 2.0739 2.6181 1.9554
H 0.0776 1.6573 6.0419
H -0.8397 0.3554 4.0188
H -1.5747 1.8288 3.4419
H 0.0161 3.0284 1.5046
H 3.7499 1.7584 2.9796
H 2.5132 2.9733 1.1013
C 5.5323 7.0787 3.0243
C 5.6074 5.7767 3.8291
C 6.0687 4.5368 3.0395
C 6.0756 3.3035 3.9549
C 5.1967 4.2933 1.8025
H 4.7780 7.0210 2.2339
H 4.6177 5.5623 4.2573
H 6.2823 5.9180 4.6836
H 7.0993 4.7093 2.6962
H 5.0770 3.1299 4.3747
H 6.3764 2.3962 3.4208
H 6.7605 3.4408 4.7991
H 4.1377 4.2750 2.0798

H 5.4420 3.3393 1.3305
H 5.3234 5.0732 1.0463
H -0.5402 -1.9062 -5.0667
H -8.7763 1.8899 4.3285
H 11.6671 4.9862 0.4895
H 12.3840 5.6914 -0.9766
H -10.0650 3.2275 -4.7720
H -9.1591 1.7943 -5.2914
H -0.8150 3.0633 5.4725
H -1.6932 1.6044 5.9641
H 6.4936 7.3054 2.5484
H 5.2714 7.9246 3.6691
H 0.0373 -1.6083 7.2632
H 1.0959 -1.2532 8.6395
H 1.8835 -3.0719 6.2685
H 1.2903 -3.6734 7.8344
H 2.8328 -2.8089 7.7283
H 9.0456 -6.4832 -0.6816
H 9.6524 -5.3499 -1.9024
H 4.5833 -6.5622 -2.2530
H 0.6394 -6.8897 -5.4898
H -1.0863 -7.2979 -5.4914
H 3.8234 -4.1097 -5.8469
H 1.8167 0.3273 4.9448
H -4.7931 1.4379 0.9868
C 2.2289 6.9954 -2.7962
C 0.9785 6.2413 -2.3846
O 0.8112 5.0523 -2.6579
N 0.0482 6.9506 -1.6841
C -1.2819 6.3816 -1.5397
C -1.9260 6.1847 -2.9236
O -1.7353 7.0014 -3.8229
N -2.6928 5.0798 -3.0360
C -3.2511 4.6847 -4.3233
C -3.3314 3.1606 -4.4405
C -1.9940 2.4217 -4.2628
C -2.2141 0.9081 -4.3404
C -0.9217 2.8852 -5.2546
C 3.7606 2.0362 -2.6029
C 2.9341 2.7946 -3.4484
C 2.9701 2.5696 -4.8215
C 3.8317 1.6059 -5.3536
C 4.6611 0.8593 -4.5127
C 4.6228 1.0620 -3.1352
C 3.6576 2.2441 -1.1396
O 3.1034 3.2297 -0.6533
C 4.2136 1.1544 -0.2181
O 5.4703 1.1535 -0.1006
O 3.3851 0.4016 0.3395
H 3.1010 6.4024 -2.5112
H 2.2293 7.0961 -3.8853
H -1.2270 5.4405 -0.9896
H -1.8907 7.0786 -0.9571
H -4.0486 2.7769 -3.7040
H -3.7535 2.9186 -5.4258
H -1.6133 2.6384 -3.2622
H -0.0097 2.2919 -5.1292
H -1.2603 2.7711 -6.2929
H -0.6508 3.9320 -5.0902
H -2.6067 0.6135 -5.3226
H -2.9286 0.5693 -3.5840
H -1.2733 0.3744 -4.1798
H -2.6218 5.1201 -5.1040
H 2.2628 3.5347 -3.0229
H 2.3206 3.1444 -5.4738
H 3.8580 1.4365 -6.4265
H 5.3373 0.1214 -4.9307
H 5.2678 0.4888 -2.4773
C -5.0447 -3.1354 3.4494
C -3.6208 -2.6093 3.6442
O -3.3805 -1.4086 3.7536
C -5.9259 -2.1889 2.6154
C -5.5922 -2.2256 1.1392
N -6.5014 -2.7813 0.3088
O -4.5180 -1.7781 0.7014
N -2.6439 -3.5454 3.8057
C -1.2881 -3.1194 4.1174
C -0.5709 -2.5592 2.8868
O -0.9003 -2.8861 1.7421
C -0.5933 -4.4321 4.5340
C -1.2678 -5.4634 3.6163
C -2.7277 -4.9848 3.5265
N 0.4825 -1.7779 3.1882
C 1.5847 -1.5421 2.2843
C 2.8611 -2.1727 2.8515
O 2.8326 -3.2006 3.5280
N 3.9887 -1.4914 2.5406
C 5.3066 -1.9696 2.9230
C 5.7198 -1.6349 4.3645
O 6.6999 -2.1880 4.8576
C 6.3666 -1.3514 2.0002
O 6.3847 0.0585 2.1383
N 5.0049 -0.6692 4.9847
C 5.4790 -0.0501 6.2079
H -6.9776 -2.4507 2.7605
H -5.7719 -1.1523 2.9311
H -5.0513 -4.1411 3.0213
H -0.8103 -4.6411 5.5868
H 0.4898 -4.3766 4.4022
H -1.1909 -6.4879 3.9886
H -0.8113 -5.4196 2.6246
H -3.3634 -5.4880 4.2670
H -3.1487 -5.1686 2.5330
H -1.3024 -2.3683 4.9113
H 1.7358 -0.4787 2.1073
H 1.3509 -2.0181 1.3331
H 7.3356 -1.7664 2.2938
H 6.1664 -1.6426 0.9625
H 6.0576 0.4434 1.2939
H 5.3379 -3.0605 2.8418
H 6.2370 0.7157 5.9997
H 2.3015 7.9875 -2.3445
H -4.2535 5.1153 -4.4510
H -2.6497 4.3853 -2.2835
H 0.0965 7.9553 -1.7659
H 5.9348 -0.8196 6.8330
H 4.6369 0.4048 6.7323
H 4.2601 -0.2342 4.4635
H 0.6808 -1.5762 4.1651
H -7.4003 -3.1444 0.6227
H -6.3308 -2.6933 -0.6871
H 3.9228 -0.7308 1.8603
H -5.4770 -3.2233 4.4537
C -11.0188 -4.9732 1.6547
C -10.0143 -4.3290 0.7225
O -8.8317 -4.2061 1.0567
C -11.2236 -4.0478 2.8582
N -10.4984 -3.8461 -0.4422
C -9.6675 -3.0883 -1.3609
C -8.8135 7.0937 3.2660
C -8.5852 5.8894 2.3628
C -8.2244 6.2596 0.9241
N -7.8512 5.1942 0.1697
O -8.2834 7.4111 0.4989
H -10.2649 -3.8527 3.3439
H -11.9006 -4.4969 3.5903
H -11.6474 -3.0876 2.5478
H -11.9690 -5.1755 1.1478
H -8.7939 -3.6758 -1.6587
H -7.7950 5.2462 2.7601
H -9.4826 5.2598 2.3251
H -7.8062 4.2433 0.5506
H -7.6100 5.3667 -0.7940
H -9.5924 7.7425 2.8574
H -9.1098 6.7737 4.2699
H -7.9055 7.6977 3.3501
H -10.6031 -5.9310 1.9809
H -9.3140 -2.1562 -0.9082
H -10.2487 -2.8482 -2.2516
H -11.4833 -3.9374 -0.6329

YI

C 11.3963 5.3047 -0.3983
C 10.8238 4.1850 -1.2800
C 9.6300 3.5032 -0.6792
C 8.3667 3.3498 -1.2093
N 9.6946 2.8912 0.5596
C 8.4918 2.3773 0.7636
N 7.6494 2.6324 -0.2758
H 10.6640 6.1077 -0.2634
H 11.6109 3.4387 -1.4546
H 10.5505 4.5826 -2.2651
H 7.9266 3.6793 -2.1382
H 8.1734 1.7925 1.6165
H 6.7066 2.2469 -0.3702
C -9.4414 2.2637 -4.5413
C -8.2642 2.8843 -3.7671
C -7.2985 1.8573 -3.2142
C -6.5306 1.0608 -4.0765
C -7.1451 1.6578 -1.8380
C -5.6577 0.0952 -3.5878
C -6.2676 0.6991 -1.3261
C -5.5223 -0.0932 -2.2059
O -4.6688 -1.0736 -1.7920
H -10.0220 1.5995 -3.8929
H -8.6521 3.4931 -2.9425
H -7.7291 3.5737 -4.4341
H -6.6120 1.2024 -5.1519
H -7.7086 2.2516 -1.1250
H -5.0617 -0.5170 -4.2562
H -6.1684 0.5944 -0.2505
H -4.5582 -1.0880 -0.8050
C -0.2757 -6.5913 -5.1643
C -0.4241 -6.4318 -3.6477
C -1.7792 -5.9562 -3.1521
C -2.0635 -5.9843 -1.7792
C -2.7725 -5.4549 -4.0029
C -3.2788 -5.5327 -1.2717
C -3.9952 -4.9983 -3.5117
C -4.2566 -5.0360 -2.1413
O -5.4845 -4.6146 -1.7138
H -0.3950 -5.6357 -5.6845
H -0.1928 -7.3888 -3.1632
H 0.3444 -5.7339 -3.2885
H -1.3169 -6.3669 -1.0888
H -2.5967 -5.4122 -5.0726
H -3.4687 -5.5642 -0.2017
H -4.7539 -4.6016 -4.1774
H -5.4741 -4.4652 -0.7554
C 4.9635 -4.2003 -5.5057
C 5.1833 -4.9893 -4.2352
O 6.2496 -5.5410 -3.9671
C 5.1404 -2.6873 -5.2906
H 5.6835 -4.5630 -6.2430
H 6.1672 -2.4591 -4.9964
H 4.4756 -2.3044 -4.5102
H 4.9216 -2.1394 -6.2119
N 4.1150 -5.0162 -3.3964
C 4.2649 -5.5386 -2.0517
C 2.9608 -5.4911 -1.2683
C 2.3165 -4.0996 -1.1306
C 1.1173 -4.2028 -0.1875
C 3.3057 -3.0235 -0.6638
H 3.3356 -4.4030 -3.5838
H 5.0395 -4.9733 -1.5230
H 3.1720 -5.8772 -0.2631
H 2.2320 -6.1788 -1.7185
H 1.9249 -3.7893 -2.1121
H 1.4455 -4.4477 0.8297
H 0.5578 -3.2717 -0.1344
H 0.4183 -4.9800 -0.5140
H 4.1387 -2.8950 -1.3625
H 3.7312 -3.2827 0.3124
H 2.8205 -2.0491 -0.5649
C 9.1837 -5.3867 -0.7367
C 7.8266 -4.6780 -0.8406
C 7.9463 -3.2016 -1.1523
C 7.4927 -2.6914 -2.3739
C 8.5239 -2.3140 -0.2341
C 7.6001 -1.3288 -2.6611

C	8.6331	-0.9538	-0.5174
C	8.1657	-0.4536	-1.7347
H	9.8074	-4.9440	0.0468
H	7.2825	-4.8062	0.1051
H	7.2357	-5.1620	-1.6228
H	7.0777	-3.3806	-3.1012
H	8.8791	-2.6918	0.7212
H	7.2483	-0.9485	-3.6161
H	9.0702	-0.2767	0.2083
H	8.2457	0.6062	-1.9496
C	-2.5483	0.6801	0.5488
C	-1.3902	0.9125	-0.1405
C	-1.2505	2.2064	-0.7477
N	-0.1099	2.5990	-1.2994
N	-2.2915	3.0907	-0.7339
C	-3.3941	2.7784	-0.0719
N	-3.5419	1.6107	0.5864
S	1.5518	-0.6626	-3.5086
C	1.2174	0.1440	-2.0220
C	-4.5162	3.7642	-0.0101
N	0.1855	-0.5153	-1.4630
C	-0.3949	-1.6092	-2.1469
C	-1.6201	-2.2845	-1.6248
C	0.2615	-1.8445	-3.3145
C	-0.0221	-2.8762	-4.3574
C	-0.2585	-0.0765	-0.1209
H	0.6542	1.9213	-1.4630
H	-0.0083	3.5325	-1.7005
H	-0.5423	-0.9520	0.4633
H	0.6268	0.3723	0.3340
H	-5.4814	3.2616	0.0631
H	-4.4049	4.3879	0.8843
H	-4.4837	4.4184	-0.8819
H	-2.7414	-0.2345	1.0972
H	-1.5418	-2.5374	-0.5655
H	-1.8047	-3.2078	-2.1727
H	-2.4951	-1.6427	-1.7609
H	-0.8620	-3.5062	-4.0617
H	0.8440	-3.5274	-4.5274
C	1.6884	-2.8271	7.4034
C	0.9812	-1.4875	7.6481
C	1.5652	-0.3442	6.8385
O	1.4528	-0.5506	5.5224
O	2.0572	0.6571	7.3259
C	-8.4021	2.7562	3.9612
C	-6.9022	2.6065	3.6835
C	-6.5436	2.1203	2.2677
O	-7.2082	2.5590	1.2950
O	-5.5407	1.3369	2.1877
H	-8.5894	3.3699	4.8480
H	-8.9007	3.2090	3.1053
H	-6.4196	1.9481	4.4104
H	-6.4119	3.5859	3.7844
C	-0.9271	1.9602	5.4632
C	-0.8725	1.3880	4.0434
C	0.3815	1.7743	3.3277
C	0.5449	2.5191	2.1864
N	1.6369	1.3860	3.7825
C	2.5200	1.8738	2.9198
N	1.9022	2.5795	1.9460
H	-0.0411	1.6887	6.0402
H	-0.9537	0.2969	4.0804
H	-1.7362	1.7335	3.4648
H	-0.1666	3.0181	1.5513
H	3.5858	1.7099	2.9439
H	2.3273	2.8841	1.0615
C	5.3902	7.1069	3.0864
C	5.4027	5.7784	3.8508
C	5.8503	4.5467	3.0403
C	5.8332	3.2989	3.9358
C	4.9841	4.3370	1.7916
H	4.6641	7.0944	2.2677
H	4.3958	5.5818	4.2463
H	6.0574	5.8749	4.7270
H	6.8857	4.7099	2.7069
H	4.8277	3.1299	4.3406
H	6.1330	2.3961	3.3941
H	6.5090	3.4179	4.7902
H	3.9226	4.3146	2.0606
H	5.2289	3.3940	1.2964
H	5.1209	5.1355	1.0563
H	-0.2722	-2.4114	-5.3177
H	-8.8633	1.7763	4.1286
H	11.6401	4.9087	0.5910
H	12.3028	5.7353	-0.8381
H	-10.1122	3.0378	-4.9288
H	-9.0845	1.6697	-5.3888
H	-0.9745	3.0540	5.4376
H	-1.8119	1.5941	5.9937
H	6.3738	7.3218	2.6523
H	5.1282	7.9391	3.7484
H	-0.0776	-1.5719	7.3717
H	1.0326	-1.2002	8.7006
H	1.7089	-3.0777	6.3391
H	1.1860	-3.6334	7.9463
H	2.7274	-2.7842	7.7435
H	9.0547	-6.4494	-0.5059
H	9.7303	-5.3073	-1.6817
H	4.6320	-6.5672	-2.1238
H	0.7181	-6.9775	-5.4083
H	-1.0191	-7.2868	-5.5671
H	3.9571	-4.4006	-5.8944
H	1.6773	0.2855	4.9562
H	-4.4453	1.4421	1.1887
C	2.1695	6.9646	-2.7791
C	0.9036	6.2450	-2.3661
O	0.7450	5.0386	-2.5753
N	-0.0490	6.9936	-1.7494
C	-1.3878	6.4442	-1.6098
C	-1.9996	6.1851	-2.9983
O	-1.8189	6.9797	-3.9170
N	-2.7336	5.0539	-3.0893
C	-3.2702	4.6017	-4.3694
C	-3.2945	3.0736	-4.4464
C	-1.9272	2.3929	-4.2707
C	-2.0905	0.8715	-4.2130
C	-0.9082	2.8118	-5.3359
C	3.7716	1.9974	-2.6011
C	3.0913	2.8447	-3.4900
C	3.3046	2.7256	-4.8591
C	4.2049	1.7742	-5.3490
C	4.8931	0.9417	-4.4652
C	4.6761	1.0441	-3.0923
C	3.4689	2.1020	-1.1531
O	2.8185	3.0459	-0.6950
C	4.0502	1.0688	-0.1834
O	5.3136	1.1369	-0.0883
O	3.2739	0.3363	0.4583
H	3.0275	6.3885	-2.4251
H	2.2107	6.9909	-3.8718
H	-1.3578	5.5343	-1.0076
H	-2.0039	7.1775	-1.0827
H	-3.9873	2.6848	-3.6892
H	-3.7241	2.7888	-5.4161
H	-1.5176	2.7096	-3.3099
H	0.0402	2.2865	-5.1819
H	-1.2676	2.5746	-6.3454
H	-0.6975	3.8845	-5.2891
H	-2.5250	0.4857	-5.1440
H	-2.7507	0.5704	-3.3937
H	-1.1251	0.3815	-4.0686
H	-2.6529	5.0406	-5.1573
H	2.3882	3.5729	-3.0988
H	2.7625	3.3692	-5.5455
H	4.3686	1.6821	-6.4190
H	5.5970	0.2085	-4.8449
H	5.2145	0.4012	-2.4056
C	-5.1106	-3.1887	3.4220
C	-3.6838	-2.7077	3.6924
O	-3.4477	-1.5368	3.9814
C	-5.9339	-2.1422	2.6439
C	-5.5348	-2.0533	1.1853
N	-6.3409	-2.6602	0.2810
O	-4.4969	-1.4730	0.8290
N	-2.7046	-3.6533	3.6976
C	-1.3430	-3.2611	4.0225
C	-0.6639	-2.5460	2.8501
O	-1.0406	-2.7178	1.6854
C	-0.6353	-4.6134	4.2422
C	-1.3165	-5.5100	3.1959
C	-2.7795	-5.0262	3.1806
N	0.4215	-1.8422	3.2112
C	1.5282	-1.5584	2.3250
C	2.8088	-2.1583	2.9165
O	2.7886	-3.1819	3.6019
N	3.9242	-1.4554	2.6177
C	5.2492	-1.9056	3.0069
C	5.6524	-1.5558	4.4471
O	6.6445	-2.0838	4.9450
C	6.2966	-1.2769	2.0759
O	6.2730	0.1361	2.1746
N	4.9116	-0.6095	5.0666
C	5.3487	-0.0042	6.3096
H	-6.9963	-2.3897	2.7178
H	-5.7543	-1.1510	3.0701
H	-5.1348	-4.1544	2.9112
H	-0.8418	-4.9691	5.2572
H	0.4464	-4.5304	4.1124
H	-1.2379	-6.5755	3.4256
H	-0.8662	-5.3332	2.2166
H	-3.4173	-5.6477	3.8222
H	-3.1931	-5.0397	2.1671
H	-1.3359	-2.6191	4.9067
H	1.6481	-0.4921	2.1524
H	1.3307	-2.0331	1.3652
H	7.2756	-1.6545	2.3859
H	6.1120	-1.6010	1.0454
H	5.9238	0.4887	1.3219
H	5.3027	-2.9965	2.9349
H	6.0916	0.7838	6.1314
H	2.2299	7.9845	-2.3931
H	-4.2865	4.9931	-4.5082
H	-2.6551	4.3823	-2.3268
H	0.0174	7.9939	-1.8647
H	5.8123	-0.7750	6.9276
H	4.4873	0.4220	6.8268
H	4.1527	-0.1963	4.5478
H	0.6292	-1.7331	4.1993
H	-7.2596	-3.0240	0.5357
H	-6.1365	-2.4882	-0.6980
H	3.8455	-0.7127	1.9146
H	-5.5780	-3.3388	4.4025
C	-10.7555	-5.1021	1.6624
C	-9.8578	-4.3240	0.7221
O	-8.7547	-3.9129	1.0945
C	-11.2732	-4.1537	2.7499
N	-10.3663	-4.0480	-0.4989
C	-9.6658	-3.2055	-1.4525
C	-8.9572	7.0087	3.1276
C	-8.6496	5.7965	2.2596
C	-8.2570	6.1500	0.8250
N	-7.7537	5.0973	0.1286
O	-8.4066	7.2729	0.3508
H	-10.4323	-3.6891	3.2698
H	-11.8818	-4.6912	3.4823
H	-11.8861	-3.3573	2.3165
H	-11.5873	-5.5721	1.1257
H	-8.7506	-3.6890	-1.8113
H	-7.8523	5.1902	2.6985
H	-9.5243	5.1368	2.2034
H	-7.5991	4.1836	0.5626
H	-7.4803	5.2552	-0.8289
H	-9.7340	7.6243	2.6677
H	-9.2923	6.6982	4.1221
H	-8.0729	7.6420	3.2447
H	-10.1545	-5.8985	2.1107
H	-9.3892	-2.2527	-0.9924
H	-10.3190	-3.0115	-2.3037
H	-11.2711	-4.4178	-0.7419

YIH^+

C	11.3077	5.4480	-0.2887
C	10.7816	4.3223	-1.1906
C	9.6087	3.5901	-0.6069
C	8.3828	3.3267	-1.1802
N	9.6632	3.0325	0.6572
C	8.4919	2.4434	0.8349
N	7.6789	2.5947	-0.2484
H	10.5485	6.2252	-0.1512
H	11.5970	3.6078	-1.3707
H	10.5009	4.7228	-2.1724
H	7.9595	3.5889	-2.1380
H	8.1761	1.8810	1.7038
H	6.7458	2.1840	-0.3436
C	-9.4275	2.0813	-4.6861
C	-8.1568	2.7997	-4.2022
C	-7.1979	1.8694	-3.4949
C	-6.3964	0.9779	-4.2216
C	-7.1129	1.8311	-2.0990
C	-5.5485	0.0786	-3.5838
C	-6.2658	0.9410	-1.4391
C	-5.4796	0.0566	-2.1857
O	-4.6495	-0.8557	-1.6063
H	-9.9643	1.6387	-3.8408
H	-8.4382	3.6171	-3.5283
H	-7.6577	3.2642	-5.0621
H	-6.4317	0.9937	-5.3086
H	-7.7246	2.5055	-1.5051
H	-4.9207	-0.6032	-4.1470
H	-6.2115	0.9514	-0.3569
H	-4.6336	-0.7490	-0.6192
C	-0.1280	-6.6439	-5.1338
C	-0.2815	-6.5095	-3.6155
C	-1.6352	-6.0380	-3.1114
C	-1.8787	-5.9878	-1.7311
C	-2.6640	-5.6112	-3.9597
C	-3.0834	-5.5203	-1.2135
C	-3.8784	-5.1417	-3.4585
C	-4.0939	-5.0876	-2.0804
O	-5.3062	-4.6348	-1.6398
H	-0.2666	-5.6830	-5.6392
H	-0.0498	-7.4745	-3.1462
H	0.4858	-5.8171	-3.2440
H	-1.1044	-6.3146	-1.0427
H	-2.5208	-5.6315	-5.0349
H	-3.2341	-5.4767	-0.1378
H	-4.6638	-4.7994	-4.1237
H	-5.2533	-4.3879	-0.7023
C	5.0800	-4.1794	-5.4216
C	5.2581	-5.0083	-4.1709
O	6.2466	-5.7153	-3.9781
C	4.8375	-2.6840	-5.1640
H	5.9821	-4.3219	-6.0197
H	5.6652	-2.2614	-4.5947
H	3.9175	-2.4855	-4.6053
H	4.7560	-2.1388	-6.1091
N	4.2278	-4.9320	-3.2917
C	4.3554	-5.5069	-1.9684
C	3.0472	-5.4319	-1.1926
C	2.4397	-4.0246	-1.0541
C	1.1994	-4.1031	-0.1646
C	3.4414	-2.9884	-0.5284
H	3.5460	-4.1989	-3.4153
H	5.1513	-4.9940	-1.4174
H	3.2361	-5.8317	-0.1882
H	2.3054	-6.0944	-1.6586
H	2.0965	-3.6872	-2.0446
H	1.4718	-4.3744	0.8620
H	0.6715	-3.1532	-0.1247
H	0.4877	-4.8473	-0.5352
H	4.3134	-2.8860	-1.1823
H	3.8042	-3.2714	0.4663
H	2.9857	-1.9985	-0.4469
C	9.2543	-5.2762	-0.5894
C	7.8847	-4.7459	-1.0298
C	7.9153	-3.2806	-1.4071
C	7.7754	-2.8693	-2.7361
C	8.1034	-2.3003	-0.4242
C	7.8109	-1.5079	-3.0669
C	8.1241	-0.9446	-0.7450
C	7.9767	-0.5419	-2.0741
H	9.6381	-4.7174	0.2713
H	7.1728	-4.8872	-0.2054
H	7.5113	-5.3340	-1.8728
H	7.6340	-3.6251	-3.5035
H	8.2225	-2.6064	0.6107
H	7.7077	-1.2023	-4.1061
H	8.2393	-0.2026	0.0365
H	7.9951	0.5142	-2.3224
C	-2.5131	0.7599	0.5131
C	-1.3496	0.9320	-0.2002
C	-1.1875	2.1955	-0.8465
N	-0.0406	2.5573	-1.4218
N	-2.2083	3.0954	-0.8458
C	-3.3029	2.8126	-0.1368
N	-3.4970	1.6903	0.5707
S	1.6191	-0.7836	-3.4825
C	1.2984	0.0482	-2.0045
C	-4.3854	3.8528	-0.0964
N	0.2286	-0.5602	-1.4607
C	-0.3864	-1.6337	-2.1461
C	-1.6288	-2.2724	-1.6187
C	0.2769	-1.9088	-3.3008
C	-0.0229	-2.9426	-4.3367
C	-0.2383	-0.0766	-0.1392
H	0.7181	1.8769	-1.5505
H	0.0615	3.4834	-1.8329
H	-0.5495	-0.9343	0.4581
H	0.6495	0.3623	0.3207
H	-5.3628	3.3757	-0.0698
H	-4.2857	4.4560	0.8139
H	-4.3123	4.5213	-0.9550
H	-2.6861	-0.1449	1.0868
H	-1.5160	-2.5969	-0.5809
H	-1.8817	-3.1485	-2.2147
H	-2.4770	-1.5832	-1.6695
H	-0.8737	-3.5572	-4.0396
H	0.8325	-3.6097	-4.4965
C	1.6155	-2.7763	7.4350
C	0.8657	-1.4534	7.6471
C	1.4619	-0.2942	6.8677
O	1.3678	-0.4762	5.5470
O	1.9480	0.6964	7.3819
C	-8.5078	2.6397	3.8261
C	-6.9792	2.7047	3.8062
C	-6.3597	2.2689	2.4860
O	-6.9332	2.4275	1.4059
O	-5.1574	1.7674	2.6309
H	-8.9167	3.2230	4.6556
H	-8.9208	3.0173	2.8919
H	-6.5305	2.1204	4.6134
H	-6.6399	3.7387	3.9602
C	-1.0427	1.9609	5.4310
C	-0.9716	1.4274	3.9978
C	0.2980	1.8179	3.3123
C	0.4836	2.5375	2.1584
N	1.5453	1.4528	3.8090
C	2.4453	1.9290	2.9569
N	1.8457	2.6052	1.9514
H	-0.1672	1.6719	6.0145
H	-1.0642	0.3364	4.0048
H	-1.8225	1.7951	3.4143
H	-0.2178	3.0037	1.4879
H	3.5114	1.7758	3.0102
H	2.2919	2.8914	1.0707
C	5.2308	7.1840	3.1056
C	5.2680	5.8541	3.8674
C	5.7690	4.6366	3.0655
C	5.7738	3.3902	3.9633
C	4.9333	4.3960	1.8017
H	4.5225	7.1514	2.2719
H	4.2597	5.6267	4.2419
H	5.9016	5.9683	4.7569
H	6.8049	4.8321	2.7517
H	4.7682	3.1966	4.3566
H	6.1013	2.4941	3.4265
H	6.4366	3.5266	4.8251
H	3.8689	4.3335	2.0519
H	5.2229	3.4628	1.3113
H	5.0525	5.1996	1.0689
H	-0.2605	-2.4808	-5.3018
H	-8.8479	1.6052	3.9408
H	11.5530	5.0470	0.6980
H	12.2042	5.9132	-0.7135
H	-10.1059	2.7709	-5.2003
H	-9.1756	1.2712	-5.3785
H	-1.0856	3.0554	5.4347
H	-1.9366	1.5849	5.9393
H	6.2161	7.4305	2.6926
H	4.9296	8.0057	3.7640
H	-0.1762	-1.5628	7.3205
H	0.8612	-1.1684	8.7016
H	1.7019	-3.0168	6.3716
H	1.1002	-3.5973	7.9428
H	2.6317	-2.7132	7.8353
H	9.1944	-6.3330	-0.3065
H	9.9853	-5.1809	-1.3982
H	4.6792	-6.5459	-2.0801
H	0.8739	-7.0063	-5.3806
H	-0.8552	-7.3492	-5.5495
H	4.2409	-4.5942	-5.9982
H	1.5881	0.3692	4.9859
H	-4.6381	1.6389	1.7466
C	2.0903	6.9600	-2.8007
C	0.8175	6.2333	-2.4380
O	0.6777	5.0298	-2.6717
N	-0.1495	6.9682	-1.8307
C	-1.4811	6.4034	-1.6836
C	-2.1068	6.1518	-3.0668
O	-2.0023	6.9843	-3.9646
N	-2.7597	4.9752	-3.1726
C	-3.2933	4.5092	-4.4477
C	-3.2804	2.9806	-4.5022
C	-1.8858	2.3434	-4.3885
C	-2.0019	0.8299	-4.1899
C	-0.9798	2.6808	-5.5775
C	3.8163	1.9784	-2.5518
C	3.1434	2.8234	-3.4488
C	3.3665	2.6988	-4.8158
C	4.2717	1.7467	-5.2956
C	4.9532	0.9167	-4.4040
C	4.7227	1.0221	-3.0336
C	3.5078	2.0936	-1.1061
O	2.8521	3.0394	-0.6607
C	4.0816	1.0707	-0.1197
O	5.3479	1.1054	-0.0340
O	3.2934	0.3751	0.5476
H	2.9324	6.4247	-2.3544
H	2.2135	6.9253	-3.8866
H	-1.4320	5.4854	-1.0950
H	-2.0983	7.1254	-1.1423
H	-3.9182	2.5939	-3.6979
H	-3.7562	2.6614	-5.4390
H	-1.3994	2.7502	-3.4987
H	0.0019	2.2115	-5.4529
H	-1.4111	2.3194	-6.5201
H	-0.8175	3.7591	-5.6681
H	-2.5054	0.3570	-5.0430
H	-2.5786	0.5879	-3.2919
H	-1.0144	0.3739	-4.0910
H	-2.6944	4.9564	-5.2462
H	2.4369	3.5538	-3.0673
H	2.8293	3.3399	-5.5084
H	4.4443	1.6521	-6.3641
H	5.6616	0.1824	-4.7729
H	5.2527	0.3784	-2.3418
C	-5.1244	-3.2601	3.3670
C	-3.7356	-2.7100	3.6917
O	-3.5544	-1.5212	3.9443
C	-6.0402	-2.1987	2.7282
C	-5.6405	-1.8990	1.2958

N -6.1923 -2.6760 0.3219
O -4.8068 -1.0252 1.0320
N -2.7303 -3.6227 3.7719
C -1.3874 -3.1759 4.1046
C -0.6994 -2.5242 2.8995
O -1.0460 -2.7800 1.7415
C -0.6531 -4.4908 4.4345
C -1.2904 -5.4774 3.4429
C -2.7623 -5.0309 3.3578
N 0.3553 -1.7671 3.2433
C 1.4781 -1.5077 2.3719
C 2.7426 -2.1126 2.9899
O 2.7056 -3.1332 3.6801
N 3.8674 -1.4188 2.7066
C 5.1802 -1.8788 3.1202
C 5.5666 -1.5094 4.5581
O 6.5471 -2.0354 5.0806
C 6.2460 -1.2784 2.1929
O 6.2567 0.1354 2.2679
N 4.8229 -0.5449 5.1439
C 5.2489 0.0925 6.3720
H -7.0749 -2.5526 2.7536
H -5.9413 -1.2667 3.2875
H -5.0692 -4.1437 2.7261
H -0.8700 -4.7786 5.4688
H 0.4283 -4.3906 4.3152
H -1.1942 -6.5212 3.7515
H -0.8205 -5.3618 2.4635
H -3.4041 -5.6163 4.0295
H -3.1510 -5.1347 2.3395
H -1.4194 -2.4750 4.9422
H 1.6096 -0.4445 2.1888
H 1.2907 -1.9927 1.4166
H 7.2153 -1.6722 2.5176
H 6.0553 -1.6128 1.1675
H 5.9097 0.4824 1.4113
H 5.2217 -2.9712 3.0647
H 5.9720 0.8952 6.1767
H 2.1068 8.0010 -2.4699
H -4.3202 4.8745 -4.5806
H -2.6126 4.2943 -2.4242
H -0.0875 7.9704 -1.9259
H 5.7336 -0.6566 7.0002
H 4.3805 0.5073 6.8872
H 4.0763 -0.1398 4.6014
H 0.5402 -1.6068 4.2295
H -7.0767 -3.1560 0.4926
H -5.9831 -2.3914 -0.6312
H 3.8070 -0.6787 1.9978
H -5.5696 -3.5922 4.3133
C -10.6014 -5.2530 1.5987
C -9.7437 -4.4416 0.6455
O -8.6664 -3.9618 1.0104
C -11.2631 -4.3181 2.6199
N -10.2653 -4.2219 -0.5826
C -9.6141 -3.3720 -1.5667
C -9.1130 6.8787 2.9589
C -8.7493 5.6530 2.1316
C -8.3153 5.9826 0.7034
N -7.7835 4.9216 0.0332
O -8.4657 7.0909 0.2022
H -10.5001 -3.7453 3.1525
H -11.8436 -4.8870 3.3515
H -11.9365 -3.6105 2.1262
H -11.3584 -5.8322 1.0577
H -8.6934 -3.8312 -1.9429
H -7.9481 5.0836 2.6116
H -9.6047 4.9694 2.0656
H -7.5547 4.0500 0.5033
H -7.4106 5.0887 -0.8889
H -9.8925 7.4607 2.4617
H -9.4684 6.5847 3.9512
H -8.2489 7.5374 3.0828
H -9.9421 -5.9608 2.1087
H -9.3542 -2.4082 -1.1210
H -10.2960 -3.2070 -2.4012
H -11.1392 -4.6628 -0.8208

TC

C 11.4860 5.3364 -0.4198
C 11.0226 4.1700 -1.3046
C 9.8486 3.4330 -0.7327
C 8.6108 3.2072 -1.2956
N 9.9058 2.8543 0.5220
C 8.7237 2.2900 0.7048
N 7.9024 2.4764 -0.3668
H 10.7001 6.0938 -0.3312
H 11.8598 3.4704 -1.4326
H 10.7654 4.5337 -2.3070
H 8.1821 3.4953 -2.2434
H 8.4046 1.7220 1.5690
H 6.9525 2.1045 -0.4426
C -9.3717 2.2975 -4.4629
C -7.9296 2.6260 -4.0433
C -7.1922 1.4359 -3.4699
C -6.9370 0.3088 -4.2647
C -6.7559 1.4092 -2.1405
C -6.2716 -0.8008 -3.7590
C -6.0785 0.3063 -1.6170
C -5.8222 -0.8035 -2.4319
O -5.1372 -1.8948 -2.0013
H -9.9521 1.9446 -3.6042
H -7.9393 3.4345 -3.3034
H -7.3845 3.0145 -4.9139
H -7.2539 0.3060 -5.3051
H -6.9486 2.2551 -1.4865
H -6.0519 -1.6602 -4.3841
H -5.7373 0.3125 -0.5882
H -4.8205 -1.7539 -1.0712
C -0.1893 -6.5264 -5.2599
C -0.2046 -5.4041 -4.2114
C -1.5312 -5.2908 -3.4960
C -1.7073 -5.7973 -2.2056
C -2.6383 -4.6950 -4.1196
C -2.9392 -5.7194 -1.5561
C -3.8743 -4.6076 -3.4873
C -4.0273 -5.1297 -2.2005
O -5.2712 -5.0590 -1.6258
H -0.9582 -6.3602 -6.0216
H 0.5914 -5.5764 -3.4786
H 0.0296 -4.4518 -4.7000
H -0.8681 -6.2578 -1.6931
H -2.5256 -4.2787 -5.1175
H -3.0497 -6.1110 -0.5476
H -4.7190 -4.1171 -3.9558
H -5.1987 -5.2758 -0.6864
C 5.0419 -4.1184 -5.6054
C 5.2500 -4.9964 -4.3899
O 6.2482 -5.6992 -4.2351
C 4.7402 -2.6489 -5.2665
H 5.9561 -4.1925 -6.1990
H 5.4866 -2.2474 -4.5769
H 3.7579 -2.5158 -4.8000
H 4.7447 -2.0356 -6.1723
N 4.2360 -4.9438 -3.4934
C 4.3695 -5.5024 -2.1645
C 3.0643 -5.3569 -1.3872
C 2.5102 -3.9223 -1.2947
C 1.2333 -3.9237 -0.4569
C 3.5357 -2.9235 -0.7436
H 3.5302 -4.2351 -3.6188
H 5.1833 -5.0001 -1.6315
H 3.2336 -5.7298 -0.3693
H 2.2997 -6.0067 -1.8348
H 2.2197 -3.5836 -2.3029
H 1.4333 -4.2537 0.5677
H 0.7979 -2.9270 -0.4084
H 0.4742 -4.5899 -0.8774
H 4.4326 -2.8597 -1.3688
H 3.8541 -3.2157 0.2637
H 3.1094 -1.9191 -0.6790
C 9.2966 -5.3552 -0.8802
C 7.9206 -4.8513 -1.3408
C 7.9440 -3.3921 -1.7403
C 7.9096 -2.9999 -3.0841
C 8.0458 -2.3975 -0.7594
C 7.9792 -1.6468 -3.4296
C 8.1010 -1.0467 -1.0968
C 8.0729 -0.6660 -2.4403
H 9.6635 -4.7765 -0.0264
H 7.2064 -4.9872 -0.5179
H 7.5599 -5.4553 -2.1779
H 7.8226 -3.7622 -3.8521
H 8.0830 -2.6900 0.2852
H 7.9695 -1.3607 -4.4786
H 8.1626 -0.2940 -0.3198
H 8.1306 0.3855 -2.7037
C -3.2369 0.9817 0.9954
C -1.9955 0.8810 0.4256
C -1.6016 1.9326 -0.4462
N -0.3883 1.9005 -1.0372
N -2.4250 2.9793 -0.6838
C -3.6231 2.9842 -0.0805
N -4.0658 2.0304 0.7474
S 1.2294 0.2850 -2.5680
C 0.5235 0.7282 -0.9327
C -4.5312 4.1458 -0.3693
N -0.1973 -0.4529 -0.5164
C -0.6991 -1.1900 -1.6205
C -1.8096 -2.1553 -1.3733
C -0.0432 -0.9621 -2.7741
C -0.1660 -1.6383 -4.0979
C -1.0252 -0.2361 0.6688
H 1.3439 0.9484 -0.2503
H -0.0846 2.7006 -1.5850
H -1.5288 -1.1590 0.9478
H -0.3597 0.0259 1.4936
H -4.8147 4.1437 -1.4261
H -5.4385 4.0870 0.2281
H -4.0114 5.0891 -0.1816
H -3.6032 0.2096 1.6654
H -1.5582 -2.8409 -0.5588
H -2.0324 -2.7382 -2.2639
H -2.7189 -1.6258 -1.0825
H -0.9710 -2.3756 -4.0797
H 0.7641 -2.1587 -4.3673
C 1.8498 -2.9182 7.3415
C 1.0779 -1.6115 7.5770
C 1.6850 -0.4326 6.8380
O 1.5392 -0.5489 5.5132
O 2.2265 0.5135 7.3781
C -8.2767 2.6840 4.0381
C -8.5052 2.0232 2.6676
C -7.3391 2.2827 1.7327
O -7.4117 3.0813 0.7976
O -6.2594 1.6079 2.0475
H -9.1035 2.4542 4.7166
H -7.3500 2.3152 4.4855
H -9.4100 2.4108 2.1936
H -8.6116 0.9412 2.7986
C -0.7900 1.8870 5.4801
C -0.7472 1.4314 4.0202
C 0.5258 1.8104 3.3333
C 0.7095 2.5487 2.1902
N 1.7702 1.3936 3.7944
C 2.6667 1.8575 2.9332
N 2.0682 2.5749 1.9554
H 0.0782 1.5372 6.0395
H -0.8836 0.3456 3.9649
H -1.5864 1.8635 3.4648
H 0.0088 3.0430 1.5381
H 3.7289 1.6720 2.9623
H 2.5037 2.8747 1.0752
C 5.4991 7.0791 3.1274
C 5.5130 5.7412 3.8750
C 6.0206 4.5314 3.0666
C 6.0065 3.2736 3.9478
C 5.2040 4.3122 1.7866
H 4.8045 7.0623 2.2820
H 4.4966 5.5161 4.2286

H 6.1312 5.8403 4.7771
H 7.0620 4.7265 2.7714
H 4.9939 3.0771 4.3211
H 6.3417 2.3837 3.4055
H 6.6541 3.3975 4.8230
H 4.1357 4.2512 2.0204
H 5.4978 3.3848 1.2881
H 5.3369 5.1262 1.0679
H -0.3809 -0.9238 -4.9012
H -8.2070 3.7720 3.9546
H 11.7096 4.9711 0.5859
H 12.3828 5.8162 -0.8275
H -9.8758 3.1761 -4.8795
H -9.3899 1.5080 -5.2209
H -0.7965 2.9801 5.5446
H -1.6919 1.5133 5.9759
H 6.4932 7.3217 2.7337
H 5.1939 7.8962 3.7898
H 0.0446 -1.7242 7.2264
H 1.0517 -1.3556 8.6387
H 1.9835 -3.1088 6.2732
H 1.3227 -3.7653 7.7905
H 2.8468 -2.8618 7.7883
H 9.2501 -6.4080 -0.5825
H 10.0325 -5.2606 -1.6854
H 4.6565 -6.5544 -2.2539
H 0.7819 -6.5840 -5.7624
H -0.3902 -7.4962 -4.7936
H 4.2286 -4.5367 -6.2131
H 1.7984 0.2947 4.9771
H -5.4261 1.8769 1.4580
C 2.2397 7.0039 -2.7179
C 1.0706 6.1537 -2.2501
O 0.9998 4.9439 -2.4605
N 0.0888 6.8330 -1.5796
C -1.2106 6.1974 -1.4476
C -1.8793 6.0752 -2.8271
O -1.7552 6.9684 -3.6640
N -2.5922 4.9468 -3.0137
C -3.2049 4.6482 -4.3020
C -3.6113 3.1775 -4.3909
C -2.4865 2.1651 -4.1062
C -3.0185 0.7393 -4.2627
C -1.2351 2.4073 -4.9562
C 4.1016 2.0663 -2.5535
C 3.4707 2.9438 -3.4514
C 3.7519 2.8528 -4.8104
C 4.6574 1.8958 -5.2786
C 5.2857 1.0239 -4.3863
C 5.0074 1.1022 -3.0250
C 3.7337 2.1342 -1.1246
O 2.9918 3.0169 -0.6824
C 4.2809 1.0819 -0.1540
O 5.5391 1.1003 -0.0124
O 3.4540 0.3634 0.4418
H 3.1636 6.4349 -2.5995
H 2.1051 7.2208 -3.7825
H -1.0960 5.2229 -0.9726
H -1.8361 6.8208 -0.8013
H -4.4415 2.9795 -3.6999
H -4.0094 3.0009 -5.3994
H -2.1906 2.2770 -3.0589
H -0.4763 1.6500 -4.7392
H -1.4700 2.3633 -6.0277
H -0.7836 3.3812 -4.7430
H -3.2560 0.5179 -5.3111
H -3.9317 0.5970 -3.6817
H -2.2846 0.0110 -3.9119
H -2.4910 4.9092 -5.0901
H 2.7471 3.6609 -3.0742
H 3.2552 3.5200 -5.5083
H 4.8707 1.8271 -6.3418
H 5.9903 0.2811 -4.7448
H 5.4969 0.4283 -2.3324
C -4.9748 -3.2458 3.4012
C -3.5716 -2.6716 3.6104
O -3.3411 -1.4636 3.5404
C -5.8955 -2.3095 2.5969
C -5.5981 -2.3709 1.1095
N -6.3707 -3.1871 0.3664
O -4.6553 -1.7285 0.6135
N -2.6162 -3.5633 3.9805
C -1.2653 -3.0979 4.2707
C -0.5242 -2.7033 2.9874
O -0.8002 -3.2197 1.9037
C -0.5832 -4.3397 4.8776
C -1.2501 -5.4908 4.1106
C -2.7064 -5.0289 3.9384
N 0.4629 -1.8128 3.2062
C 1.5726 -1.6128 2.3017
C 2.8451 -2.2046 2.9162
O 2.8167 -3.2023 3.6373
N 3.9738 -1.5245 2.6016
C 5.2871 -2.0053 2.9944
C 5.6886 -1.6916 4.4419
O 6.6421 -2.2780 4.9488
C 6.3566 -1.3754 2.0897
O 6.4052 0.0308 2.2510
N 4.9997 -0.6984 5.0498
C 5.4960 -0.0727 6.2596
H -6.9384 -2.5917 2.7718
H -5.7407 -1.2790 2.9240
H -4.9324 -4.2298 2.9261
H -0.8054 -4.3971 5.9485
H 0.5003 -4.3063 4.7448
H -1.1790 -6.4522 4.6251
H -0.7852 -5.5834 3.1268
H -3.3498 -5.4011 4.7473
H -3.1236 -5.3702 2.9863
H -1.3016 -2.2485 4.9576
H 1.7131 -0.5552 2.0815
H 1.3472 -2.1171 1.3643
H 7.3200 -1.8126 2.3755
H 6.1430 -1.6400 1.0481
H 6.0707 0.4331 1.4155
H 5.3248 -3.0950 2.8981
H 6.2299 0.7112 6.0319
H 2.3181 7.9474 -2.1727
H -4.0811 5.2914 -4.4536
H -2.5158 4.2139 -2.3082
H 0.0586 7.8285 -1.7514
H 5.9876 -0.8373 6.8625
H 4.6619 0.3611 6.8147
H 4.2884 -0.2348 4.5062
H 0.6448 -1.5342 4.1665
H -7.2188 -3.6337 0.7075
H -6.1819 -3.2335 -0.6310
H 3.9129 -0.7917 1.8881
H -5.4045 -3.4074 4.3980
C -11.0634 -5.0964 1.5051
C -10.0039 -4.5224 0.5863
O -8.8115 -4.5555 0.9036
C -11.1394 -4.2172 2.7576
N -10.4506 -3.9193 -0.5370
C -9.5624 -3.2114 -1.4428
C -8.8474 6.9449 3.2628
H -10.1567 -4.1614 3.2313
H -11.8526 -4.6211 3.4817
H -11.4533 -3.1997 2.5038
H -12.0391 -5.1603 1.0104
H -8.7803 -3.8793 -1.8154
H -8.5042 6.7254 4.2785
H -8.8083 8.0241 3.0997
H -9.8939 6.6341 3.1813
H -10.7569 -6.1113 1.7740
H -9.0735 -2.3679 -0.9455
H -10.1398 -2.8372 -2.2883
H -11.4419 -3.9075 -0.7159
C -7.9818 6.2356 2.2220
C -8.3033 6.6920 0.7983
N -8.1643 5.7273 -0.1482
O -8.6386 7.8443 0.5406
H -6.9223 6.4659 2.3961
H -8.0722 5.1482 2.2904
H -7.9454 4.7652 0.0895
H -8.3266 5.9892 -1.1082

TCH^+

C 11.5049 5.3282 -0.3546
C 11.0289 4.1798 -1.2562
C 9.8532 3.4389 -0.6920
C 8.6168 3.2180 -1.2598
N 9.9082 2.8469 0.5566
C 8.7265 2.2796 0.7310
N 7.9070 2.4769 -0.3401
H 10.7248 6.0894 -0.2488
H 11.8611 3.4774 -1.4009
H 10.7689 4.5619 -2.2509
H 8.1899 3.5156 -2.2055
H 8.4059 1.7028 1.5889
H 6.9572 2.1070 -0.4212
C -9.3509 2.3864 -4.4786
C -7.9584 2.7553 -3.9416
C -7.1964 1.5628 -3.4067
C -6.8220 0.5173 -4.2632
C -6.8646 1.4490 -2.0517
C -6.1386 -0.5968 -3.7925
C -6.1719 0.3390 -1.5624
C -5.7920 -0.6862 -2.4378
O -5.0877 -1.7791 -2.0409
H -9.9628 1.9401 -3.6879
H -8.0584 3.5041 -3.1477
H -7.3824 3.2345 -4.7446
H -7.0593 0.5825 -5.3226
H -7.1517 2.2249 -1.3468
H -5.8260 -1.3915 -4.4622
H -5.9304 0.2743 -0.5074
H -4.8053 -1.6829 -1.0931
C -0.1922 -6.4571 -5.3295
C -0.2062 -5.3536 -4.2620
C -1.5409 -5.2332 -3.5631
C -1.7445 -5.7687 -2.2884
C -2.6280 -4.6007 -4.1859
C -2.9835 -5.6828 -1.6535
C -3.8701 -4.5029 -3.5672
C -4.0511 -5.0543 -2.2961
O -5.3003 -4.9722 -1.7362
H -0.9472 -6.2655 -6.0991
H 0.5772 -5.5496 -3.5217
H 0.0500 -4.3986 -4.7334
H -0.9216 -6.2587 -1.7765
H -2.4941 -4.1623 -5.1718
H -3.1157 -6.0980 -0.6571
H -4.6980 -3.9826 -4.0334
H -5.2475 -5.2223 -0.8037
C 5.0469 -4.0615 -5.6401
C 5.2456 -4.9497 -4.4308
O 6.2340 -5.6679 -4.2842
C 4.7353 -2.5957 -5.2949
H 5.9677 -4.1274 -6.2244
H 5.4701 -2.1964 -4.5917
H 3.7452 -2.4692 -4.8428
H 4.7509 -1.9758 -6.1961
N 4.2344 -4.8906 -3.5316
C 4.3614 -5.4739 -2.2133
C 3.0527 -5.3534 -1.4394
C 2.4902 -3.9248 -1.3194
C 1.2191 -3.9506 -0.4738
C 3.5120 -2.9282 -0.7574
H 3.5387 -4.1705 -3.6465
H 5.1694 -4.9790 -1.6647
H 3.2216 -5.7459 -0.4289
H 2.2939 -5.9988 -1.9025
H 2.1919 -3.5719 -2.3205
H 1.4267 -4.3043 0.5412
H 0.7832 -2.9563 -0.3975
H 0.4587 -4.6084 -0.9053
H 4.4059 -2.8500 -1.3851
H 3.8367 -3.2330 0.2442
H 3.0812 -1.9269 -0.6763

C	9.2855	-5.3524	-0.9150
C	7.9101	-4.8441	-1.3730
C	7.9353	-3.3823	-1.7628
C	7.9004	-2.9815	-3.1040
C	8.0394	-2.3942	-0.7755
C	7.9722	-1.6263	-3.4410
C	8.0972	-1.0414	-1.1045
C	8.0689	-0.6520	-2.4456
H	9.6522	-4.7797	-0.0571
H	7.1953	-4.9848	-0.5516
H	7.5489	-5.4417	-2.2145
H	7.8123	-3.7387	-3.8769
H	8.0772	-2.6937	0.2671
H	7.9629	-1.3336	-4.4882
H	8.1616	-0.2933	-0.3230
H	8.1293	0.4009	-2.7022
C	-3.2228	0.8688	1.0273
C	-1.9859	0.8097	0.4582
C	-1.6148	1.8807	-0.4086
N	-0.4024	1.8828	-0.9853
N	-2.4642	2.9115	-0.6513
C	-3.6621	2.8969	-0.0698
N	-4.0551	1.9117	0.7581
S	1.2386	0.3199	-2.5405
C	0.5346	0.7183	-0.8978
C	-4.6132	4.0188	-0.3471
N	-0.1686	-0.4756	-0.4995
C	-0.6645	-1.2024	-1.6149
C	-1.7641	-2.1836	-1.3840
C	-0.0126	-0.9452	-2.7643
C	-0.1223	-1.6027	-4.0986
C	-0.9937	-0.2890	0.6905
H	1.3480	0.9499	-0.2112
H	-0.1207	2.6834	-1.5450
H	-1.4807	-1.2229	0.9616
H	-0.3328	-0.0182	1.5158
H	-4.8038	4.0755	-1.4221
H	-5.5617	3.8703	0.1721
H	-4.1557	4.9680	-0.0522
H	-3.5978	0.1005	1.6946
H	-1.5153	-2.8670	-0.5675
H	-1.9609	-2.7685	-2.2791
H	-2.6888	-1.6721	-1.1105
H	-0.9375	-2.3284	-4.1026
H	0.8052	-2.1317	-4.3588
C	1.8242	-2.9661	7.3085
C	1.0682	-1.6562	7.5745
C	1.6714	-0.4747	6.8377
O	1.5235	-0.5904	5.5124
O	2.2102	0.4734	7.3766
C	-8.2771	2.6950	4.0281
C	-8.4352	1.8873	2.7283
C	-7.2485	2.1190	1.7932
O	-7.3290	3.0196	0.9205
O	-6.2151	1.3999	1.9979
H	-9.0899	2.4732	4.7274
H	-7.3298	2.4447	4.5150
H	-9.3547	2.1793	2.2121
H	-8.4879	0.8201	2.9663
C	-0.7966	1.8630	5.4827
C	-0.7500	1.4096	4.0221
C	0.5257	1.7874	3.3391
C	0.7171	2.5411	2.2074
N	1.7660	1.3531	3.7943
C	2.6676	1.8227	2.9419
N	2.0766	2.5599	1.9745
H	0.0685	1.5088	6.0442
H	-0.8881	0.3239	3.9640
H	-1.5872	1.8452	3.4664
H	0.0229	3.0572	1.5653
H	3.7282	1.6280	2.9700
H	2.5144	2.8661	1.0975
C	5.5138	7.0572	3.1923
C	5.5135	5.7114	3.9255
C	6.0191	4.5068	3.1082
C	5.9960	3.2412	3.9779
C	5.2067	4.3030	1.8230
H	4.8262	7.0540	2.3409
H	4.4932	5.4891	4.2698
H	6.1259	5.7970	4.8328
H	7.0626	4.6998	2.8190
H	4.9806	3.0443	4.3436
H	6.3321	2.3552	3.4301
H	6.6386	3.3555	4.8580
H	4.1372	4.2451	2.0525
H	5.4981	3.3789	1.3168
H	5.3463	5.1232	1.1125
H	-0.3138	-0.8750	-4.8955
H	-8.2880	3.7702	3.8364
H	11.7319	4.9449	0.6436
H	12.4024	5.8086	-0.7599
H	-9.8763	3.2676	-4.8622
H	-9.2786	1.6572	-5.2919
H	-0.7993	2.9560	5.5483
H	-1.7019	1.4922	5.9741
H	6.5125	7.2979	2.8094
H	5.2081	7.8689	3.8608
H	0.0277	-1.7553	7.2411
H	1.0625	-1.4124	8.6392
H	1.9420	-3.1429	6.2361
H	1.2962	-3.8148	7.7534
H	2.8281	-2.9252	7.7415
H	9.2379	-6.4072	-0.6245
H	10.0220	-5.2529	-1.7191
H	4.6555	-6.5217	-2.3236
H	0.7854	-6.5207	-5.8189
H	-0.4147	-7.4320	-4.8841
H	4.2427	-4.4791	-6.2603
H	1.7863	0.2510	4.9772
H	-5.0629	1.8319	1.2371
C	2.2695	7.0431	-2.6620
C	1.1027	6.1838	-2.2030
O	1.0270	4.9784	-2.4367
N	0.1238	6.8489	-1.5135
C	-1.1767	6.2135	-1.3986
C	-1.8358	6.1107	-2.7849
O	-1.6714	6.9949	-3.6228
N	-2.5884	5.0077	-2.9780
C	-3.1777	4.7174	-4.2810
C	-3.5502	3.2393	-4.3953
C	-2.4052	2.2509	-4.1092
C	-2.9092	0.8144	-4.2554
C	-1.1606	2.5151	-4.9627
C	4.1167	2.0857	-2.5358
C	3.4964	2.9775	-3.4273
C	3.7921	2.9068	-4.7844
C	4.7014	1.9556	-5.2574
C	5.3189	1.0695	-4.3716
C	5.0266	1.1279	-3.0123
C	3.7328	2.1318	-1.1103
O	2.9778	3.0026	-0.6655
C	4.2800	1.0737	-0.1454
O	5.5372	1.0984	0.0003
O	3.4540	0.3475	0.4430
H	3.1909	6.4631	-2.5873
H	2.1115	7.3050	-3.7132
H	-1.0648	5.2315	-0.9375
H	-1.8063	6.8262	-0.7465
H	-4.3854	3.0133	-3.7187
H	-3.9317	3.0665	-5.4107
H	-2.1096	2.3737	-3.0627
H	-0.3869	1.7725	-4.7466
H	-1.3977	2.4639	-6.0332
H	-0.7271	3.4980	-4.7531
H	-3.1578	0.5859	-5.2996
H	-3.8115	0.6549	-3.6619
H	-2.1564	0.1020	-3.9138
H	-2.4562	5.0067	-5.0514
H	2.7697	3.6901	-3.0477
H	3.3045	3.5858	-5.4773
H	4.9263	1.9029	-6.3190
H	6.0264	0.3312	-4.7337
H	5.5089	0.4434	-2.3249
C	-4.9909	-3.2389	3.3477
C	-3.5792	-2.6856	3.5560
O	-3.3189	-1.4851	3.4576
C	-5.9024	-2.2964	2.5392
C	-5.5938	-2.3523	1.0547
N	-6.3849	-3.1301	0.2919
O	-4.6249	-1.7361	0.5729
N	-2.6444	-3.5852	3.9606
C	-1.2901	-3.1360	4.2628
C	-0.5296	-2.7524	2.9879
O	-0.7959	-3.2685	1.9015
C	-0.6290	-4.3837	4.8821
C	-1.3119	-5.5306	4.1238
C	-2.7614	-5.0496	3.9564
N	0.4612	-1.8671	3.2137
C	1.5722	-1.6663	2.3114
C	2.8485	-2.2491	2.9249
O	2.8281	-3.2412	3.6530
N	3.9726	-1.5669	2.5976
C	5.2905	-2.0383	2.9870
C	5.6883	-1.7299	4.4365
O	6.6466	-2.3107	4.9404
C	6.3530	-1.3910	2.0867
O	6.3883	0.0140	2.2627
N	4.9900	-0.7463	5.0492
C	5.4815	-0.1219	6.2612
H	-6.9460	-2.5818	2.7035
H	-5.7621	-1.2627	2.8646
H	-4.9597	-4.2256	2.8768
H	-0.8547	-4.4287	5.9528
H	0.4552	-4.3676	4.7532
H	-1.2509	-6.4903	4.6430
H	-0.8524	-5.6336	3.1383
H	-3.4006	-5.3903	4.7826
H	-3.1977	-5.4070	3.0193
H	-1.3220	-2.2842	4.9474
H	1.7099	-0.6084	2.0887
H	1.3512	-2.1752	1.3755
H	7.3208	-1.8226	2.3659
H	6.1398	-1.6466	1.0427
H	6.0625	0.4219	1.4269
H	5.3387	-3.1268	2.8833
H	6.2142	0.6643	6.0371
H	2.3667	7.9629	-2.0803
H	-4.0657	5.3431	-4.4352
H	-2.5384	4.2698	-2.2795
H	0.0970	7.8489	-1.6568
H	5.9736	-0.8860	6.8645
H	4.6452	0.3091	6.8150
H	4.2768	-0.2845	4.5068
H	0.6385	-1.5845	4.1739
H	-7.2446	-3.5648	0.6212
H	-6.1898	-3.1615	-0.7046
H	3.9056	-0.8325	1.8869
H	-5.4223	-3.3920	4.3450
C	-11.0678	-5.0585	1.4227
C	-10.0077	-4.4764	0.5099
O	-8.8145	-4.5153	0.8249
C	-11.1566	-4.1864	2.6796
N	-10.4539	-3.8657	-0.6095
C	-9.5656	-3.1483	-1.5077
C	-8.8334	6.9642	3.2890
H	-10.1773	-4.1269	3.1599
H	-11.8717	-4.5990	3.3968
H	-11.4748	-3.1693	2.4298
H	-12.0405	-5.1258	0.9226
H	-8.7555	-3.8016	-1.8436
H	-8.4090	6.8968	4.2958
H	-8.9286	8.0159	3.0111
H	-9.8406	6.5345	3.3176
H	-10.7571	-6.0731	1.6882
H	-9.1157	-2.2775	-1.0201
H	-10.1321	-2.8132	-2.3768
H	-11.4466	-3.8372	-0.7791
C	-7.9607	6.2446	2.2669
C	-8.4789	6.4980	0.8483
N	-8.5553	5.3965	0.0619
O	-8.8076	7.6222	0.4739

H -6.9393 6.6444 2.3065
H -7.8862 5.1724 2.4565
H -8.2334 4.4782 0.3752
H -8.8439 5.5361 -0.8945

R-Mandelate
Model E

AP

C 11.1550 5.6121 0.0247
C 10.7220 4.4540 -0.8839
C 9.6267 3.6291 -0.2794
C 8.3796 3.3364 -0.7884
N 9.7798 3.0316 0.9578
C 8.6444 2.3894 1.1806
N 7.7623 2.5457 0.1554
H 10.3283 6.3133 0.1807
H 11.5929 3.8136 -1.0801
H 10.3914 4.8367 -1.8573
H 7.8889 3.6161 -1.7086
H 8.4091 1.7762 2.0403
H 6.8454 2.0845 0.0876
C -9.5140 2.1268 -4.5873
C -8.4963 2.9610 -3.7896
C -7.4456 2.0851 -3.1489
C -6.3483 1.6242 -3.8899
C -7.5806 1.6336 -1.8311
C -5.4403 0.7181 -3.3503
C -6.6791 0.7302 -1.2725
C -5.6124 0.2524 -2.0414
O -4.7331 -0.6735 -1.5749
H -10.0172 1.4082 -3.9323
H -9.0217 3.5370 -3.0186
H -8.0228 3.6900 -4.4584
H -6.2060 1.9760 -4.9091
H -8.4007 2.0018 -1.2200
H -4.5928 0.3597 -3.9251
H -6.7751 0.4426 -0.2329
H -4.7766 -0.7463 -0.5825
C -0.0579 -6.3899 -5.5150
C -0.1439 -6.0244 -4.0279
C -1.5117 -5.6031 -3.5186
C -1.7976 -5.6796 -2.1486
C -2.5162 -5.0983 -4.3554
C -3.0216 -5.2685 -1.6271
C -3.7497 -4.6852 -3.8515
C -4.0108 -4.7693 -2.4816
O -5.2439 -4.3817 -2.0410
H -0.2685 -5.5285 -6.1574
H 0.1980 -6.8772 -3.4295
H 0.5776 -5.2216 -3.8181
H -1.0470 -6.0725 -1.4698
H -2.3427 -5.0243 -5.4242
H -3.2058 -5.3309 -0.5575
H -4.5196 -4.2924 -4.5068
H -5.2419 -4.2872 -1.0748
C 5.1052 -3.8195 -5.6545
C 5.3295 -4.6620 -4.4242
O 6.3708 -5.2878 -4.2333
C 6.0599 -2.6254 -5.7441
H 5.2577 -4.4727 -6.5213
H 7.0971 -2.9667 -5.7657
H 5.9393 -1.9663 -4.8820
H 5.8666 -2.0466 -6.6522
N 4.2840 -4.6849 -3.5608
C 4.4029 -5.3537 -2.2836
C 3.0864 -5.3641 -1.5173
C 2.4831 -3.9774 -1.2312
C 1.2518 -4.1381 -0.3357
C 3.5114 -3.0182 -0.6186
H 3.5342 -4.0234 -3.6882
H 5.1824 -4.8717 -1.6850
H 3.2676 -5.8671 -0.5593
H 2.3482 -5.9728 -2.0564
H 2.1356 -3.5483 -2.1854
H 1.5470 -4.4028 0.6857
H 0.6570 -3.2269 -0.2747
H 0.5898 -4.9226 -0.7142
H 4.3422 -2.8101 -1.2999
H 3.9297 -3.4455 0.2982
H 3.0731 -2.0551 -0.3500
C 9.2955 -5.1129 -0.8870
C 7.9352 -4.4698 -1.1836
C 8.0470 -2.9965 -1.5046
C 7.9330 -2.5391 -2.8216
C 8.2956 -2.0605 -0.4936
C 8.0475 -1.1776 -3.1152
C 8.3931 -0.7006 -0.7770
C 8.2685 -0.2524 -2.0938
H 9.7877 -4.6250 -0.0395
H 7.2814 -4.6015 -0.3109
H 7.4659 -4.9903 -2.0233
H 7.7573 -3.2640 -3.6085
H 8.4025 -2.3967 0.5318
H 7.9710 -0.8399 -4.1457
H 8.5511 0.0032 0.0294
H 8.3495 0.8081 -2.3103
C -2.6610 0.7173 0.6360
C -1.4596 0.9686 0.0108
C -1.3655 2.1953 -0.7127
N -0.2270 2.6415 -1.2549
N -2.4750 2.9567 -0.8830
C -3.6029 2.6016 -0.2652
N -3.7330 1.5326 0.5336
S 1.7864 -0.6827 -3.0740
C 1.4323 -0.1127 -1.5218
C -4.8037 3.4700 -0.4989
N 0.2244 -0.4699 -1.1179
C -0.4870 -1.2796 -2.0150
C -1.8215 -1.8315 -1.6511
C 0.2298 -1.4819 -3.1609
C -0.1766 -2.2235 -4.3935
C -0.3066 0.0294 0.1834
H 2.1209 0.4103 -0.8563
H -0.2123 3.5817 -1.6477
H -0.6096 -0.8434 0.7636
H 0.5350 0.5143 0.6801
H -5.1552 3.3321 -1.5269
H -5.6134 3.1952 0.1717
H -4.5439 4.5244 -0.3756
H -2.7957 -0.1660 1.2547
H -1.8062 -2.2310 -0.6324
H -2.0817 -2.6461 -2.3262
H -2.6092 -1.0782 -1.7176
H -1.0132 -2.8899 -4.1799
H 0.6446 -2.8311 -4.7863
C 1.6096 -3.2104 7.2554
C 0.7805 -1.9195 7.2544
C 1.5017 -0.7640 6.5439
O 1.6887 -0.9321 5.2680
O 1.8545 0.2322 7.1846
C -8.6084 2.2181 3.9443
C -7.0871 2.3324 3.9328
C -6.4928 1.9812 2.5826
O -7.0927 2.1970 1.5270
O -5.2866 1.4768 2.6623
H -9.0408 2.7382 4.8036
H -9.0312 2.6325 3.0316
H -6.6142 1.7172 4.7021
H -6.7768 3.3666 4.1363
C -1.1329 1.5841 5.5193
C -1.0597 1.2340 4.0268
C 0.1988 1.7200 3.3848
C 0.4364 2.6171 2.3670
N 1.4502 1.2935 3.7888
C 2.3700 1.9083 3.0115
N 1.7970 2.7261 2.1349
H -0.2704 1.1991 6.0689
H -1.1458 0.1486 3.9027
H -1.9131 1.6683 3.4948
H -0.2817 3.1886 1.7970
H 3.4280 1.7134 3.0902
H 0.6978 2.3290 -0.9564
C 5.0461 7.0430 3.5030
C 5.0721 5.6606 4.1658
C 5.7316 4.5445 3.3336
C 5.7516 3.2313 4.1303
C 5.0352 4.3452 1.9823
H 4.4372 7.0425 2.5937
H 4.0437 5.3529 4.4011
H 5.5950 5.7341 5.1288
H 6.7739 4.8352 3.1349
H 4.7389 2.9513 4.4415
H 6.1592 2.4028 3.5413
H 6.3550 3.3258 5.0397
H 3.9648 4.1526 2.1145
H 5.4731 3.4923 1.4594
H 5.1425 5.2210 1.3337
H -0.4857 -1.5275 -5.1813
H -8.9142 1.1678 3.9998
H 11.4477 5.2227 1.0031
H 11.9997 6.1645 -0.4023
H -10.2768 2.7607 -5.0523
H -9.0116 1.5590 -5.3770
H -1.1518 2.6701 5.6583
H -2.0413 1.1657 5.9647
H 6.0562 7.3662 3.2246
H 4.6288 7.7966 4.1796
H -0.1718 -2.0977 6.7360
H 0.5507 -1.6025 8.2753
H 1.8869 -3.4845 6.2329
H 1.0576 -4.0426 7.7061
H 2.5360 -3.0724 7.8224
H 9.1868 -6.1769 -0.6501
H 9.9597 -5.0229 -1.7527
H 4.7478 -6.3759 -2.4678
H 0.9474 -6.7434 -5.7607
H -0.7713 -7.1796 -5.7709
H 4.0660 -3.4707 -5.7009
H 1.6368 0.5290 4.4879
H -4.8317 1.4006 1.7452
C 1.9128 7.0976 -2.4112
C 0.6615 6.3263 -2.0737
O 0.5362 5.1356 -2.3721
N -0.3182 7.0151 -1.4320
C -1.6478 6.4376 -1.3563
C -2.2203 6.2324 -2.7704
O -2.0297 7.0712 -3.6489
N -2.9327 5.0941 -2.9091
C -3.4248 4.6475 -4.2024
C -2.8409 3.2771 -4.5764
C -1.3056 3.2163 -4.6155
C -0.8294 1.7735 -4.8203
C -0.7093 4.1673 -5.6583
C 3.8676 2.0541 -2.2418
C 3.0120 2.7130 -3.1374
C 3.1007 2.4621 -4.5069
C 4.0426 1.5575 -5.0023
C 4.9046 0.9117 -4.1156
C 4.8191 1.1541 -2.7435
C 3.7590 2.3557 -0.7507
O 2.4273 2.7271 -0.4597
C 4.2302 1.1562 0.1059
O 5.4772 1.0497 0.2650
O 3.3603 0.3709 0.5622
H 2.7812 6.4807 -2.1718
H 1.9219 7.2869 -3.4891
H -1.6139 5.4978 -0.8009
H -2.2911 7.1312 -0.8085
H -3.2081 2.5376 -3.8538
H -3.2516 2.9831 -5.5532
H -0.9344 3.5437 -3.6426
H 0.3834 4.0981 -5.6600
H -1.0642 3.9251 -6.6685
H -0.9673 5.2073 -5.4404
H -1.1622 1.3790 -5.7897
H -1.2207 1.1162 -4.0356
H 0.2629 1.7129 -4.7865

H	-3.1603	5.4172	-4.9296
H	2.2823	3.4218	-2.7619
H	2.4316	2.9803	-5.1878
H	4.1098	1.3657	-6.0695
H	5.6554	0.2196	-4.4825
H	5.4977	0.6476	-2.0658
C	-5.1186	-3.5843	3.1566
C	-3.7628	-2.9444	3.4951
O	-3.6737	-1.7554	3.7943
C	-6.1130	-2.5528	2.5920
C	-5.7408	-2.1140	1.1890
N	-6.3066	-2.7930	0.1532
O	-4.9224	-1.2066	0.9947
N	-2.6905	-3.7782	3.5191
C	-1.3741	-3.2790	3.8999
C	-0.6607	-2.5954	2.7267
O	-1.0247	-2.7797	1.5534
C	-0.6042	-4.5686	4.2524
C	-1.1664	-5.5699	3.2313
C	-2.6512	-5.1841	3.0994
N	0.4164	-1.8885	3.0955
C	1.5370	-1.6315	2.2172
C	2.7619	-2.3918	2.7343
O	2.6642	-3.5367	3.1841
N	3.9122	-1.6907	2.6468
C	5.1869	-2.2107	3.1060
C	5.6244	-1.6784	4.4821
O	6.7290	-1.9900	4.9250
C	6.3157	-1.8794	2.1191
O	6.6231	-0.5033	2.0986
N	4.7802	-0.8330	5.1126
C	5.1910	-0.2208	6.3621
H	-7.1194	-2.9810	2.5891
H	-6.0795	-1.6608	3.2205
H	-5.0065	-4.4221	2.4630
H	-0.8475	-4.8694	5.2766
H	0.4762	-4.4343	4.1658
H	-1.0373	-6.6122	3.5329
H	-0.6658	-5.4261	2.2705
H	-3.2887	-5.7987	3.7486
H	-3.0047	-5.2997	2.0687
H	-1.4622	-2.5852	4.7386
H	1.7517	-0.5680	2.1477
H	1.2948	-2.0026	1.2240
H	7.2007	-2.4184	2.4689
H	6.0451	-2.2529	1.1223
H	6.1203	-0.0520	1.3842
H	5.0834	-3.2969	3.1906
H	6.0135	0.4805	6.1873
H	1.9848	8.0507	-1.8822
H	-4.5201	4.5748	-4.1772
H	-2.8136	4.3799	-2.1850
H	-0.2600	8.0217	-1.4618
H	5.5428	-0.9748	7.0749
H	4.3316	0.3050	6.7810
H	3.7937	-0.8150	4.8715
H	0.6611	-1.7469	4.0905
H	-7.1618	-3.3340	0.2919
H	-6.1275	-2.4131	-0.7709
H	3.8985	-0.8217	2.1119
H	-5.5254	-4.0021	4.0857
C	-10.6239	-5.5813	1.2384
C	-9.7527	-4.7234	0.3416
O	-8.6640	-4.2922	0.7341
C	-11.2367	-4.7087	2.3416
N	-10.2694	-4.4090	-0.8673
C	-9.6032	-3.4972	-1.7822
C	-9.2897	6.4878	3.3180
C	-8.9730	5.2892	2.4326
C	-8.7079	5.6472	0.9699
N	-8.2151	4.6113	0.2318
O	-8.9522	6.7530	0.5010
H	-10.4459	-4.2082	2.9056
H	-11.8275	-5.3135	3.0349
H	-11.8904	-3.9403	1.9174
H	-11.4082	-6.0875	0.6644
H	-8.6370	-3.9000	-2.1035
H	-8.1067	4.7412	2.8150
H	-9.8078	4.5782	2.4471
H	-7.8854	3.7536	0.6657
H	-7.9459	4.8032	-0.7212
H	-10.1359	7.0498	2.9158
H	-9.5305	6.1636	4.3349
H	-8.4402	7.1748	3.3670
H	-9.9836	-6.3506	1.6790
H	-9.4253	-2.5288	-1.3048
H	-10.2337	-3.3506	-2.6595
H	-11.1656	-4.7911	-1.1233
H	4.4399	3.1863	-0.5182
H	2.3148	2.8404	0.5347

APH$^+$

C	11.2962	5.5216	-0.4931
C	10.8329	4.3092	-1.3114
C	9.7028	3.5683	-0.6631
C	8.4517	3.2837	-1.1658
N	9.8266	3.0434	0.6098
C	8.6703	2.4508	0.8603
N	7.8016	2.5715	-0.1819
H	10.4938	6.2617	-0.4052
H	11.6831	3.6258	-1.4409
H	10.5300	4.6225	-2.3180
H	7.9790	3.5208	-2.1070
H	8.4100	1.8966	1.7522
H	6.8794	2.1223	-0.2346
C	-9.4841	2.0562	-4.5917
C	-8.4275	2.7434	-3.7122
C	-7.4271	1.7528	-3.1654
C	-6.5385	1.0953	-4.0286
C	-7.3852	1.4216	-1.8076
C	-5.6565	0.1287	-3.5608
C	-6.5058	0.4551	-1.3180
C	-5.6429	-0.2019	-2.2003
O	-4.7817	-1.1803	-1.8053
H	-10.0327	1.3040	-4.0156
H	-8.9193	3.2638	-2.8826
H	-7.9139	3.5146	-4.3016
H	-6.5355	1.3470	-5.0865
H	-8.0282	1.9389	-1.1010
H	-4.9649	-0.3762	-4.2266
H	-6.4677	0.2561	-0.2531
H	-4.6798	-1.2232	-0.8151
C	-0.1642	-6.6449	-5.0869
C	-0.3000	-6.3689	-3.5862
C	-1.6695	-5.9153	-3.1076
C	-1.9095	-5.7779	-1.7325
C	-2.7236	-5.6085	-3.9769
C	-3.1438	-5.3647	-1.2396
C	-3.9683	-5.1958	-3.5006
C	-4.1885	-5.0807	-2.1273
O	-5.4356	-4.7129	-1.7082
H	-0.3464	-5.7429	-5.6811
H	-0.0213	-7.2737	-3.0312
H	0.4445	-5.6157	-3.2939
H	-1.1134	-5.9985	-1.0281
H	-2.5819	-5.6925	-5.0491
H	-3.2914	-5.2507	-0.1684
H	-4.7785	-4.9607	-4.1823
H	-5.4326	-4.5636	-0.7501
C	5.0324	-4.1674	-5.4628
C	5.2499	-4.9260	-4.1785
O	6.2671	-5.5854	-3.9711
C	6.1621	-3.1850	-5.7794
H	4.9751	-4.9176	-6.2615
H	7.1190	-3.7111	-5.8112
H	6.2307	-2.4091	-5.0141
H	5.9959	-2.7027	-6.7470
N	4.2244	-4.8534	-3.2931
C	4.3566	-5.4679	-1.9898
C	3.0581	-5.4621	-1.1966
C	2.4473	-4.0745	-0.9391
C	1.2675	-4.2181	0.0262
C	3.4862	-3.0713	-0.4217

H	3.5078	-4.1579	-3.4295
H	5.1461	-4.9636	-1.4242
H	3.2675	-5.9346	-0.2288
H	2.3123	-6.0938	-1.6975
H	2.0461	-3.6969	-1.8933
H	1.6231	-4.4037	1.0457
H	0.6383	-3.3297	0.0616
H	0.6233	-5.0540	-0.2655
H	4.2790	-2.8775	-1.1511
H	3.9563	-3.4496	0.4916
H	3.0422	-2.1050	-0.1750
C	9.2716	-5.2107	-0.6772
C	7.9106	-4.5752	-0.9868
C	8.0199	-3.1250	-1.4011
C	7.8722	-2.7445	-2.7392
C	8.2983	-2.1330	-0.4537
C	7.9782	-1.4015	-3.1126
C	8.3847	-0.7910	-0.8155
C	8.2219	-0.4191	-2.1516
H	9.7847	-4.6740	0.1275
H	7.2754	-4.6467	-0.0937
H	7.4159	-5.1418	-1.7803
H	7.6795	-3.5125	-3.4800
H	8.4338	-2.4131	0.5851
H	7.8788	-1.1217	-4.1585
H	8.5612	-0.0409	-0.0556
H	8.2957	0.6269	-2.4315
C	-2.7508	0.7813	0.6335
C	-1.5275	1.0290	0.0606
C	-1.4093	2.2341	-0.7098
N	-0.2662	2.6716	-1.2293
N	-2.5173	2.9903	-0.9523
C	-3.6745	2.6597	-0.4011
N	-3.8151	1.5951	0.4179
S	1.7260	-0.9266	-2.8479
C	1.3560	-0.1737	-1.3797
C	-4.8734	3.4928	-0.7088
N	0.1620	-0.5188	-0.9236
C	-0.5191	-1.4654	-1.7038
C	-1.8465	-1.9871	-1.2899
C	0.2032	-1.7892	-2.8171
C	-0.1864	-2.6918	-3.9431
C	-0.3749	0.1027	0.3213
H	2.0402	0.4375	-0.7909
H	-0.2429	3.5688	-1.7179
H	-0.6794	-0.7128	0.9779
H	0.4597	0.6345	0.7788
H	-5.6677	3.3262	0.0212
H	-4.5925	4.5465	-0.7483
H	-5.2614	3.2065	-1.6927
H	-2.9391	-0.0753	1.2737
H	-1.8660	-2.2211	-0.2229
H	-2.0757	-2.8993	-1.8391
H	-2.6360	-1.2712	-1.5170
H	-0.9578	-3.3942	-3.6241
H	0.6673	-3.2721	-4.3058
C	1.7306	-2.6659	7.4252
C	0.9135	-1.3693	7.3323
C	1.6393	-0.2671	6.5449
O	1.8102	-0.5121	5.2786
O	2.0140	0.7596	7.1217
C	-8.4555	2.6893	3.9029
C	-7.2329	1.8303	3.5844
C	-6.7279	1.9288	2.1438
O	-7.1297	2.8584	1.4142
O	-5.8558	1.0430	1.8093
H	-8.7298	2.5922	4.9588
H	-8.2579	3.7406	3.6972
H	-7.4012	0.7706	3.8076
H	-6.3916	2.1235	4.2268
C	-0.9680	2.0463	5.4162
C	-0.9217	1.5855	3.9548
C	0.3219	2.0318	3.2603
C	0.5368	2.8833	2.2002
N	1.5809	1.6161	3.6517
C	2.4823	2.1937	2.8261
N	1.8919	2.9767	1.9291

H -0.0952 1.7016 5.9764
H -1.0052 0.4939 3.9090
H -1.7844 1.9810 3.4070
H -0.1981 3.4481 1.6442
H 3.5415 2.0032 2.8908
H 0.6718 2.3507 -0.9719
C 5.2586 7.2682 2.9652
C 5.2865 5.9274 3.7089
C 5.8862 4.7471 2.9208
C 5.9051 3.4845 3.7952
C 5.1327 4.4881 1.6097
H 4.6089 7.2276 2.0856
H 4.2629 5.6586 4.0054
H 5.8515 6.0467 4.6429
H 6.9272 4.9963 2.6665
H 4.8964 3.2434 4.1486
H 6.2809 2.6130 3.2488
H 6.5344 3.6219 4.6812
H 4.0595 4.3605 1.7892
H 5.5071 3.5774 1.1362
H 5.2599 5.3089 0.8961
H -0.5839 -2.1143 -4.7851
H -9.3214 2.3901 3.3014
H 11.5658 5.2033 0.5172
H 12.1645 6.0079 -0.9525
H -10.2064 2.7783 -4.9873
H -9.0172 1.5461 -5.4401
H -0.9844 3.1396 5.4731
H -1.8671 1.6639 5.9104
H 6.2615 7.5509 2.6234
H 4.8883 8.0701 3.6131
H -0.0409 -1.5761 6.8286
H 0.6867 -0.9816 8.3292
H 2.0034 -3.0164 6.4251
H 1.1695 -3.4565 7.9353
H 2.6589 -2.4968 7.9803
H 9.1596 -6.2562 -0.3702
H 9.9194 -5.1826 -1.5596
H 4.7038 -6.4941 -2.1422
H 0.8459 -6.9948 -5.3174
H -0.8720 -7.4113 -5.4184
H 4.0664 -3.6474 -5.4543
H 1.7778 0.8924 4.3912
H -4.7918 1.3829 0.9826
C 2.0423 6.9849 -2.8976
C 0.7990 6.2444 -2.4503
O 0.6647 5.0274 -2.6041
N -0.1738 7.0059 -1.8785
C -1.5114 6.4556 -1.7479
C -2.0956 6.1403 -3.1370
O -1.9023 6.9013 -4.0815
N -2.8186 5.0012 -3.1952
C -3.3548 4.5042 -4.4535
C -3.2925 2.9753 -4.5016
C -1.8776 2.3850 -4.3948
C -1.9431 0.8707 -4.1724
C -1.0057 2.7317 -5.6055
C 3.7617 1.8676 -2.4601
C 2.8888 2.4727 -3.3770
C 2.8989 2.0822 -4.7162
C 3.7694 1.0828 -5.1582
C 4.6447 0.4856 -4.2508
C 4.6463 0.8760 -2.9104
C 3.7382 2.3224 -1.0033
O 2.4278 2.7438 -0.6788
C 4.2430 1.2037 -0.0635
O 5.4955 1.0890 0.0285
O 3.3896 0.4789 0.5094
H 2.9143 6.3545 -2.7179
H 1.9672 7.1668 -3.9745
H -1.4890 5.5715 -1.1070
H -2.1429 7.2040 -1.2618
H -3.9141 2.5674 -3.6955
H -3.7604 2.6392 -5.4365
H -1.3938 2.8214 -3.5169
H -0.0199 2.2657 -5.5105
H -1.4612 2.3723 -6.5370
H -0.8513 3.8109 -5.6949
H -2.4117 0.3643 -5.0258
H -2.5300 0.6225 -3.2821
H -0.9394 0.4547 -4.0452
H -2.7818 4.9652 -5.2621
H 2.2119 3.2517 -3.0411
H 2.2275 2.5693 -5.4172
H 3.7705 0.7793 -6.2015
H 5.3357 -0.2855 -4.5779
H 5.3391 0.4118 -2.2171
C -5.0601 -3.2042 3.4496
C -3.6673 -2.5987 3.6904
O -3.5171 -1.3853 3.8297
C -5.9498 -2.2498 2.6317
C -5.5626 -2.1858 1.1690
N -6.3910 -2.7860 0.2834
O -4.5246 -1.6198 0.7847
N -2.6304 -3.4653 3.8267
C -1.3005 -2.9651 4.1580
C -0.5888 -2.3811 2.9342
O -0.9501 -2.6686 1.7813
C -0.5497 -4.2376 4.6019
C -1.1513 -5.3089 3.6795
C -2.6317 -4.9075 3.5515
N 0.4840 -1.6362 3.2333
C 1.5868 -1.4412 2.3176
C 2.8256 -2.1596 2.8621
O 2.7429 -3.2692 3.3943
N 3.9696 -1.4629 2.6970
C 5.2594 -1.9495 3.1486
C 5.7201 -1.3627 4.4935
O 6.8316 -1.6589 4.9300
C 6.3575 -1.6222 2.1287
O 6.6041 -0.2337 2.0505
N 4.8949 -0.4796 5.0936
C 5.3411 0.2047 6.2908
H -6.9957 -2.5555 2.7192
H -5.8254 -1.2328 3.0144
H -5.0066 -4.1878 2.9761
H -0.7779 -4.4475 5.6520
H 0.5313 -4.1314 4.4872
H -1.0320 -6.3251 4.0631
H -0.6725 -5.2530 2.6986
H -3.2591 -5.4400 4.2782
H -3.0191 -5.1172 2.5488
H -1.3663 -2.2089 4.9433
H 1.7978 -0.3846 2.1699
H 1.3251 -1.8807 1.3570
H 7.2672 -2.1162 2.4812
H 6.0814 -2.0403 1.1524
H 6.1392 0.1447 1.2711
H 5.1816 -3.0344 3.2689
H 6.1839 0.8629 6.0565
H 2.1680 7.9419 -2.3859
H -4.3964 4.8304 -4.5765
H -2.7488 4.3512 -2.4107
H -0.1126 8.0019 -2.0291
H 5.6765 -0.5063 7.0544
H 4.5045 0.7866 6.6808
H 3.9053 -0.4591 4.8661
H 0.7462 -1.4146 4.2099
H -7.2905 -3.1827 0.5583
H -6.1990 -2.6393 -0.7015
H 3.9420 -0.6316 2.1056
H -5.5147 -3.3533 4.4361
C -10.7599 -5.2454 1.6820
C -9.8447 -4.4921 0.7412
O -8.7314 -4.1106 1.1154
C -11.2047 -4.2867 2.7914
N -10.3442 -4.1945 -0.4777
C -9.6132 -3.3711 -1.4241
C -9.0849 6.9190 3.0081
C -8.6986 5.7749 2.0818
C -8.4478 6.1936 0.6342
N -8.0051 5.1627 -0.1427
O -8.6381 7.3289 0.2125
H -10.3300 -3.8740 3.2987
H -11.8260 -4.8008 3.5299
H -11.7839 -3.4539 2.3806
H -11.6243 -5.6644 1.1547
H -8.7054 -3.8764 -1.7718
H -7.7921 5.2683 2.4283
H -9.4728 4.9986 2.0760
H -7.7462 4.2844 0.2950
H -7.7027 5.3733 -1.0807
H -9.9797 7.4310 2.6449
H -9.2785 6.5476 4.0192
H -8.2881 7.6669 3.0627
H -10.1910 -6.0775 2.1069
H -9.3196 -2.4235 -0.9636
H -10.2514 -3.1628 -2.2832
H -11.2534 -4.5469 -0.7302
H 4.4377 3.1628 -0.8906
H 2.3654 2.9480 0.3059

IP

C 11.5227 5.2065 -0.5049
C 11.0975 3.9557 -1.2848
C 9.8858 3.3011 -0.6962
C 8.6434 3.1135 -1.2614
N 9.8974 2.7928 0.5900
C 8.6825 2.3047 0.7858
N 7.8826 2.4821 -0.3018
H 10.7384 5.9702 -0.5392
H 11.9292 3.2390 -1.2892
H 10.8980 4.2117 -2.3325
H 8.2440 3.3709 -2.2309
H 8.3268 1.7881 1.6675
H 6.9441 2.0768 -0.4095
C -9.3570 2.3325 -4.5551
C -8.1653 2.7930 -3.7018
C -7.3001 1.6469 -3.2267
C -6.4697 0.9547 -4.1192
C -7.3084 1.2345 -1.8896
C -5.6735 -0.1057 -3.6944
C -6.5047 0.1846 -1.4432
C -5.6781 -0.4893 -2.3474
O -4.8700 -1.5292 -1.9835
H -10.0005 1.6568 -3.9823
H -8.5380 3.3477 -2.8329
H -7.5595 3.4984 -4.2860
H -6.4339 1.2571 -5.1637
H -7.9200 1.7402 -1.1453
H -5.0222 -0.6317 -4.3843
H -6.5049 -0.0443 -0.3826
H -4.7424 -1.5638 -1.0010
C -0.2951 -6.6376 -5.0352
C -0.3297 -6.3040 -3.5395
C -1.6793 -5.8695 -2.9977
C -1.9752 -6.0414 -1.6390
C -2.6617 -5.2723 -3.8004
C -3.2004 -5.6547 -1.1012
C -3.8919 -4.8775 -3.2792
C -4.1730 -5.0834 -1.9275
O -5.4198 -4.7514 -1.4824
H -0.4920 -5.7558 -5.6532
H 0.0049 -7.1786 -2.9691
H 0.4143 -5.5222 -3.3311
H -1.2355 -6.4976 -0.9869
H -2.4733 -5.1130 -4.8571
H -3.4052 -5.8058 -0.0442
H -4.6425 -4.4026 -3.9006
H -5.4407 -4.7397 -0.5148
C 4.9707 -4.3137 -5.4289
C 5.1893 -5.0731 -4.1427
O 6.2581 -5.6162 -3.8668
C 5.6122 -2.9198 -5.3803
H 5.4196 -4.8987 -6.2373
H 6.6937 -3.0030 -5.2524
H 5.2169 -2.3307 -4.5481
H 5.4157 -2.3727 -6.3072
N 4.1271 -5.0754 -3.3002
C 4.2637 -5.5817 -1.9502

C 2.9563 -5.4877 -1.1761
C 2.3571 -4.0747 -1.0581
C 1.1231 -4.1367 -0.1584
C 3.3761 -3.0435 -0.5551
H 3.3293 -4.4986 -3.5168
H 5.0504 -5.0269 -1.4292
H 3.1450 -5.8685 -0.1647
H 2.2106 -6.1560 -1.6267
H 2.0135 -3.7506 -2.0544
H 1.4066 -4.3845 0.8708
H 0.5886 -3.1903 -0.1240
H 0.4111 -4.8920 -0.5058
H 4.2341 -2.9443 -1.2276
H 3.7581 -3.3304 0.4308
H 2.9346 -2.0482 -0.4589
C 9.1865 -5.4629 -0.6470
C 7.8486 -4.7186 -0.7529
C 8.0132 -3.2646 -1.1377
C 7.6542 -2.8226 -2.4158
C 8.5538 -2.3359 -0.2382
C 7.8204 -1.4846 -2.7803
C 8.7192 -0.9994 -0.5968
C 8.3493 -0.5677 -1.8727
H 9.8412 -5.0002 0.0988
H 7.3225 -4.7854 0.2088
H 7.2281 -5.2200 -1.5005
H 7.2649 -3.5468 -3.1223
H 8.8372 -2.6628 0.7589
H 7.5426 -1.1594 -3.7793
H 9.1268 -0.2885 0.1135
H 8.4797 0.4735 -2.1463
C -3.0392 0.7072 0.5892
C -1.8451 0.9721 0.0043
C -1.6707 2.2318 -0.7117
N -0.4910 2.5521 -1.1393
N -2.8038 3.0453 -0.8783
C -3.9273 2.7160 -0.2894
N -4.0766 1.5904 0.4583
S 1.5343 -0.4919 -3.0021
C 1.0145 0.2155 -1.5491
C -5.1221 3.6129 -0.3996
N -0.0497 -0.3946 -1.0543
C -0.5423 -1.4594 -1.8244
C -1.7781 -2.1853 -1.4192
C 0.2205 -1.6511 -2.9392
C 0.0078 -2.6198 -4.0575
C -0.6685 0.0757 0.2145
H 1.4966 1.0636 -1.0795
H -0.4768 3.4582 -1.6043
H -0.9357 -0.8104 0.7878
H 0.1223 0.6103 0.7380
H -6.0066 3.0418 -0.6838
H -5.3395 4.0819 0.5649
H -4.9305 4.3976 -1.1311
H -3.2484 -0.1821 1.1725
H -1.7701 -2.4404 -0.3577
H -1.8729 -3.1093 -1.9875
H -2.6617 -1.5786 -1.6280
H -0.7586 -3.3464 -3.7867
H 0.9257 -3.1652 -4.3017
C 1.7376 -2.6712 7.4592
C 0.9828 -1.3433 7.6156
C 1.6163 -0.2107 6.8282
O 1.4909 -0.3819 5.5075
O 2.1629 0.7516 7.3346
C -8.2950 2.9655 3.9354
C -6.9813 2.6683 3.2064
C -7.1752 1.9314 1.8666
O -8.0544 2.3715 1.0830
O -6.3963 0.9507 1.6332
H -8.1606 3.6929 4.7428
H -9.0341 3.3490 3.2323
H -6.2951 2.0936 3.8339
H -6.4724 3.6133 2.9655
C -0.8265 2.1103 5.4380
C -0.8071 1.5625 4.0117
C 0.4558 1.9053 3.2915
C 0.6196 2.5733 2.1039
N 1.7092 1.5296 3.7649
C 2.5923 1.9442 2.8636
N 1.9742 2.5923 1.8513
H 0.0500 1.7943 6.0053
H -0.9422 0.4763 4.0268
H -1.6541 1.9565 3.4405
H -0.0837 2.9957 1.4055
H 3.6567 1.7711 2.8902
H 2.3695 2.8120 0.9316
C 5.5448 7.1402 2.9576
C 5.4926 5.8200 3.7352
C 5.9683 4.5713 2.9673
C 5.9214 3.3413 3.8865
C 5.1479 4.3287 1.6931
H 4.8668 7.1313 2.0983
H 4.4628 5.6426 4.0769
H 6.0993 5.9172 4.6451
H 7.0140 4.7294 2.6653
H 4.9058 3.1879 4.2715
H 6.2257 2.4261 3.3686
H 6.5784 3.4731 4.7533
H 4.0795 4.2726 1.9292
H 5.4456 3.3894 1.2188
H 5.2858 5.1284 0.9584
H -0.3245 -2.1043 -4.9655
H -8.7040 2.0493 4.3765
H 11.6890 4.9487 0.5443
H 12.4437 5.6408 -0.9099
H -9.9627 3.1819 -4.8892
H -9.0142 1.7893 -5.4420
H -0.8283 3.2055 5.4319
H -1.7215 1.7741 5.9717
H 6.5551 7.3358 2.5795
H 5.2577 7.9830 3.5952
H -0.0481 -1.4594 7.2597
H 0.9495 -1.0334 8.6626
H 1.8451 -2.9397 6.4045
H 1.2104 -3.4782 7.9769
H 2.7441 -2.5958 7.8813
H 9.0335 -6.5092 -0.3618
H 9.7109 -5.4446 -1.6076
H 4.6048 -6.6207 -2.0032
H 0.6901 -7.0202 -5.3179
H -1.0429 -7.3956 -5.2881
H 3.8991 -4.2249 -5.6465
H 1.7449 0.4558 4.9509
H -4.9953 1.3641 0.9178
C 2.3108 6.9298 -2.8986
C 1.2072 6.1873 -2.1833
O 1.2468 4.9694 -1.9782
N 0.1398 6.9472 -1.8091
C -1.1559 6.3026 -1.6625
C -1.7071 6.0044 -3.0697
O -1.3241 6.6870 -4.0241
N -2.6012 5.0039 -3.1491
C -3.1589 4.6017 -4.4363
C -3.2584 3.0761 -4.5457
C -1.9284 2.3250 -4.3706
C -2.1636 0.8117 -4.3897
C -0.8776 2.7388 -5.4066
C 3.9015 1.9344 -2.7439
C 2.9623 2.4498 -3.6477
C 3.0050 2.0880 -4.9957
C 3.9758 1.2010 -5.4573
C 4.9127 0.6840 -4.5605
C 4.8806 1.0462 -3.2151
C 3.8857 2.3741 -1.2806
O 2.6101 2.8283 -0.8544
C 4.3054 1.2229 -0.3303
O 5.5605 1.0613 -0.2285
O 3.4219 0.5573 0.2486
H 3.2528 6.3925 -2.7856
H 2.0516 6.9650 -3.9625
H -1.0553 5.4039 -1.0547
H -1.8360 6.9843 -1.1432
H -3.9762 2.7063 -3.8032
H -3.6886 2.8357 -5.5280
H -1.5243 2.5707 -3.3853
H 0.0406 2.1602 -5.2643
H -1.2338 2.5595 -6.4295
H -0.6176 3.7985 -5.3195
H -2.5900 0.4877 -5.3481
H -2.8565 0.5077 -3.5999
H -1.2234 0.2726 -4.2410
H -2.5197 5.0274 -5.2117
H 2.1886 3.1300 -3.3094
H 2.2719 2.5017 -5.6811
H 4.0064 0.9186 -6.5057
H 5.6775 -0.0028 -4.9079
H 5.6090 0.6347 -2.5249
C -5.0732 -3.0258 3.4973
C -3.6583 -2.4614 3.6485
O -3.4449 -1.2513 3.7018
C -5.9807 -2.1508 2.6167
C -5.6540 -2.2673 1.1432
N -6.5767 -2.8442 0.3477
O -4.5740 -1.8603 0.6780
N -2.6600 -3.3683 3.8409
C -1.3101 -2.9063 4.1284
C -0.6059 -2.3827 2.8742
O -0.9392 -2.7542 1.7449
C -0.5902 -4.1907 4.5904
C -1.2472 -5.2655 3.7111
C -2.7160 -4.8181 3.6100
N 0.4403 -1.5787 3.1413
C 1.5483 -1.3826 2.2318
C 2.8147 -1.9891 2.8461
O 2.7698 -3.0078 3.5367
N 3.9495 -1.3041 2.5711
C 5.2521 -1.7991 2.9885
C 5.6534 -1.4677 4.4349
O 6.6083 -2.0544 4.9397
C 6.3369 -1.2199 2.0707
O 6.3343 0.1948 2.1295
N 4.9667 -0.4798 5.0530
C 5.4279 0.0891 6.3083
H -7.0259 -2.4258 2.7836
H -5.8535 -1.0942 2.8723
H -5.0633 -4.0561 3.1324
H -0.8018 -4.3660 5.6505
H 0.4914 -4.1204 4.4532
H -1.1500 -6.2748 4.1185
H -0.7939 -5.2472 2.7171
H -3.3398 -5.3086 4.3689
H -3.1359 -5.0416 2.6241
H -1.3328 -2.1268 4.8943
H 1.7001 -0.3300 2.0000
H 1.3220 -1.9053 1.3038
H 7.2989 -1.6111 2.4167
H 6.1712 -1.5655 1.0438
H 6.0451 0.5124 1.2398
H 5.2633 -2.8916 2.9238
H 6.1949 0.8559 6.1411
H 2.4220 7.9554 -2.5377
H -4.1569 5.0418 -4.5647
H -2.7459 4.3960 -2.3322
H 0.0786 7.8312 -2.2991
H 5.8681 -0.7100 6.9064
H 4.5812 0.5309 6.8365
H 4.2368 -0.0194 4.5321
H 0.6460 -1.3708 4.1153
H -7.4651 -3.2170 0.6795
H -6.3923 -2.8506 -0.6486
H 3.9114 -0.5725 1.8560
H -5.4944 -3.0601 4.5098
C -11.1520 -4.8543 1.6351
C -10.0945 -4.2984 0.7058
O -8.9050 -4.2894 1.0390
C -11.2478 -3.9315 2.8537
N -10.5334 -3.7637 -0.4537
C -9.6381 -3.0775 -1.3679
C -8.8027 7.2085 3.0265
C -8.7004 5.9230 2.2173

C -8.4378 6.1513 0.7290
N -8.1951 5.0087 0.0366
O -8.4510 7.2682 0.2162
H -10.2695 -3.8463 3.3318
H -11.9620 -4.3175 3.5866
H -11.5703 -2.9272 2.5616
H -12.1223 -4.9497 1.1349
H -8.7883 -3.7205 -1.6128
H -7.9062 5.2825 2.6099
H -9.6223 5.3356 2.3070
H -8.1916 4.0777 0.4699
H -8.0291 5.0961 -0.9543
H -9.5888 7.8562 2.6301
H -9.0223 6.9879 4.0758
H -7.8680 7.7744 2.9802
H -10.8337 -5.8549 1.9424
H -9.2516 -2.1467 -0.9395
H -10.1771 -2.8438 -2.2866
H -11.5243 -3.7486 -0.6348
H 4.6330 3.1693 -1.1477
H 2.3102 3.6271 -1.3304

YI

C 11.3606 5.3381 -0.5581
C 10.9679 4.0679 -1.3238
C 9.7680 3.3876 -0.7364
C 8.5864 3.0289 -1.3492
N 9.7388 3.0114 0.5931
C 8.5609 2.4345 0.7707
N 7.8225 2.4251 -0.3747
H 10.5635 6.0872 -0.6124
H 11.8190 3.3731 -1.3129
H 10.7718 4.3021 -2.3772
H 8.2296 3.1503 -2.3610
H 8.1926 1.9790 1.6803
H 6.9046 1.9690 -0.4806
C -9.4022 1.8812 -4.7516
C -8.1049 2.5914 -4.3316
C -7.1741 1.6743 -3.5729
C -6.4278 0.6997 -4.2492
C -7.0764 1.7188 -2.1780
C -5.6224 -0.2020 -3.5629
C -6.2688 0.8287 -1.4700
C -5.5393 -0.1415 -2.1669
O -4.7525 -1.0615 -1.5419
H -9.9403 1.5131 -3.8720
H -8.3510 3.4622 -3.7131
H -7.6004 2.9773 -5.2265
H -6.4774 0.6479 -5.3345
H -7.6506 2.4558 -1.6220
H -5.0404 -0.9528 -4.0860
H -6.2059 0.8990 -0.3905
H -4.7105 -0.8904 -0.5630
C -0.1666 -6.9231 -4.7119
C -0.3097 -6.6759 -3.2064
C -1.6764 -6.2216 -2.7203
C -1.9077 -6.0801 -1.3443
C -2.7275 -5.8917 -3.5841
C -3.1209 -5.6128 -0.8473
C -3.9506 -5.4226 -3.1032
C -4.1519 -5.2717 -1.7304
O -5.3708 -4.8162 -1.3077
H -0.3556 -6.0143 -5.2915
H -0.0330 -7.5909 -2.6666
H 0.4324 -5.9262 -2.9002
H -1.1154 -6.3288 -0.6441
H -2.5940 -5.9867 -4.6566
H -3.2623 -5.4967 0.2243
H -4.7524 -5.1525 -3.7820
H -5.3130 -4.5052 -0.3901
C 5.0593 -4.5168 -5.1392
C 5.2203 -5.2968 -3.8519
O 6.2073 -5.9915 -3.6102
C 4.7529 -3.0256 -4.9209
H 5.9939 -4.6419 -5.6903
H 5.4650 -2.5747 -4.2255
H 3.7523 -2.8497 -4.5143
H 4.8063 -2.4796 -5.8674
N 4.1843 -5.1774 -2.9829
C 4.3276 -5.6430 -1.6170
C 3.0368 -5.4675 -0.8251
C 2.4312 -4.0518 -0.8239
C 1.1966 -4.0467 0.0773
C 3.4348 -2.9659 -0.4111
H 3.4958 -4.4610 -3.1560
H 5.1553 -5.1137 -1.1333
H 3.2433 -5.7601 0.2124
H 2.2823 -6.1710 -1.2027
H 2.0781 -3.8160 -1.8403
H 1.4801 -4.1879 1.1269
H 0.6451 -3.1119 0.0044
H 0.5009 -4.8464 -0.1964
H 4.2976 -2.9233 -1.0831
H 3.8117 -3.1523 0.6009
H 2.9776 -1.9723 -0.4193
C 9.2284 -5.3707 -0.2555
C 7.8605 -4.7669 -0.6049
C 7.9576 -3.3771 -1.1949
C 7.8241 -3.1555 -2.5718
C 8.2044 -2.2779 -0.3643
C 7.9187 -1.8617 -3.0936
C 8.2869 -0.9853 -0.8777
C 8.1412 -0.7725 -2.2492
H 9.7656 -4.7390 0.4593
H 7.2534 -4.7220 0.3090
H 7.3339 -5.4208 -1.3053
H 7.6369 -4.0016 -3.2259
H 8.3222 -2.4379 0.7032
H 7.8211 -1.7080 -4.1656
H 8.4485 -0.1473 -0.2104
H 8.2060 0.2356 -2.6464
C -2.5646 0.7263 0.4020
C -1.4096 0.8493 -0.3354
C -1.2271 2.0901 -1.0150
N -0.0867 2.4007 -1.6314
N -2.2201 3.0222 -1.0167
C -3.3122 2.7841 -0.2872
N -3.5236 1.6829 0.4467
S 1.5013 -1.0447 -3.5651
C 1.2164 -0.1434 -2.1197
C -4.3717 3.8470 -0.2585
N 0.1488 -0.7174 -1.5334
C -0.4817 -1.8209 -2.1523
C -1.7155 -2.4252 -1.5683
C 0.1575 -2.1533 -3.3054
C -0.1799 -3.2226 -4.2922
C -0.3030 -0.1608 -0.2386
H 0.6983 1.7365 -1.6813
H 0.0917 3.3690 -1.8733
H -0.6059 -0.9789 0.4150
H 0.5875 0.3112 0.1822
H -5.3585 3.3898 -0.3087
H -4.3176 4.4022 0.6848
H -4.2375 4.5503 -1.0809
H -2.7447 -0.1526 1.0125
H -1.5744 -2.7198 -0.5252
H -2.0018 -3.3120 -2.1314
H -2.5534 -1.7236 -1.6121
H -0.9967 -3.8464 -3.9267
H 0.6794 -3.8746 -4.4867
C 1.6149 -2.3683 7.6192
C 0.8488 -1.0418 7.7217
C 1.4546 0.0659 6.8770
O 1.3453 -0.1782 5.5678
O 1.9628 1.0721 7.3371
C -8.4717 2.9102 3.7155
C -6.9431 2.9515 3.6614
C -6.3576 2.4322 2.3561
O -6.9495 2.5363 1.2794
O -5.1628 1.9145 2.5117
H -8.8554 3.5458 4.5183
H -8.8984 3.2401 2.7695
H -6.4854 2.4072 4.4911
H -6.5873 3.9875 3.7493
C -1.0104 2.2681 5.3532
C -0.9998 1.6307 3.9639
C 0.2526 1.9642 3.2234
C 0.4164 2.5836 2.0101
N 1.5088 1.6601 3.7376
C 2.3929 2.0713 2.8354
N 1.7747 2.6507 1.7836
H -0.1167 2.0125 5.9236
H -1.1031 0.5445 4.0523
H -1.8660 1.9650 3.3827
H -0.2985 2.9843 1.3120
H 3.4613 1.9357 2.8842
H 2.2069 2.8361 0.8687
C 5.2991 7.3064 2.7355
C 5.2283 6.0164 3.5624
C 5.7627 4.7456 2.8721
C 5.6983 3.5563 3.8429
C 5.0073 4.4269 1.5756
H 4.6666 7.2507 1.8440
H 4.1853 5.8339 3.8585
H 5.7854 6.1624 4.4973
H 6.8178 4.9133 2.6106
H 4.6738 3.4146 4.2090
H 6.0202 2.6211 3.3731
H 6.3324 3.7287 4.7197
H 3.9327 4.3336 1.7669
H 5.3641 3.4861 1.1478
H 5.1447 5.2062 0.8190
H -0.4863 -2.7937 -5.2535
H -8.8234 1.8892 3.8960
H 11.5177 5.0982 0.4965
H 12.2786 5.7823 -0.9595
H -10.0674 2.5537 -5.3041
H -9.1821 1.0187 -5.3892
H -1.0409 3.3604 5.2762
H -1.8878 1.9452 5.9236
H 6.3242 7.5052 2.4016
H 4.9657 8.1683 3.3232
H -0.1848 -1.1846 7.3830
H 0.8221 -0.6845 8.7537
H 1.7143 -2.6854 6.5771
H 1.1011 -3.1551 8.1798
H 2.6253 -2.2647 8.0257
H 9.1183 -6.3670 0.1859
H 9.8519 -5.4621 -1.1510
H 4.6156 -6.6984 -1.6431
H 0.8485 -7.2580 -4.9429
H -0.8641 -7.6926 -5.0593
H 4.2635 -4.9775 -5.7398
H 1.5711 0.6401 4.9681
H -4.6647 1.7116 1.6314
C 2.1524 6.7738 -3.1477
C 1.0004 6.1576 -2.3971
O 1.1064 5.0445 -1.8602
N -0.1319 6.8947 -2.3463
C -1.4159 6.3034 -1.9972
C -2.1236 5.9856 -3.3209
O -2.1154 6.8218 -4.2284
N -2.6705 4.7627 -3.4060
C -3.2502 4.2738 -4.6522
C -3.3333 2.7462 -4.6306
C -1.9747 2.0283 -4.5442
C -2.1635 0.5492 -4.1988
C -1.1403 2.1875 -5.8205
C 3.8675 1.9366 -2.7180
C 2.9358 2.4691 -3.6161
C 2.9456 2.0942 -4.9595
C 3.8912 1.1827 -5.4292
C 4.8259 0.6516 -4.5396
C 4.8170 1.0236 -3.1959
C 3.8901 2.3962 -1.2630
O 2.6642 2.9778 -0.8396
C 4.2573 1.2607 -0.2759
O 5.5038 0.9916 -0.2505
O 3.3765 0.7288 0.4239
H 3.0390 6.7728 -2.5084

H 2.3771 6.1428 -4.0131
H -1.2585 5.4229 -1.3758
H -2.0097 7.0308 -1.4348
H -3.9579 2.4396 -3.7822
H -3.8685 2.4136 -5.5295
H -1.4015 2.4759 -3.7273
H -0.1715 1.6905 -5.7039
H -1.6498 1.7363 -6.6816
H -0.9475 3.2387 -6.0588
H -2.7575 0.0383 -4.9671
H -2.6834 0.4253 -3.2445
H -1.1990 0.0420 -4.1267
H -2.6384 4.6420 -5.4811
H 2.1835 3.1687 -3.2703
H 2.2044 2.5107 -5.6361
H 3.8956 0.8859 -6.4741
H 5.5678 -0.0620 -4.8847
H 5.5361 0.5921 -2.5092
C -5.1315 -3.0307 3.5874
C -3.7465 -2.4281 3.8413
O -3.5989 -1.2279 4.0589
C -6.0782 -2.0415 2.8820
C -5.6983 -1.8541 1.4266
N -6.2645 -2.6998 0.5234
O -4.8625 -1.0047 1.0915
N -2.7086 -3.3050 3.9012
C -1.3665 -2.8260 4.1953
C -0.6836 -2.2550 2.9446
O -1.0576 -2.5684 1.8091
C -0.6234 -4.1122 4.6093
C -1.2503 -5.1625 3.6778
C -2.7252 -4.7327 3.5590
N 0.3885 -1.4979 3.2304
C 1.5016 -1.2792 2.3308
C 2.7740 -1.8296 2.9797
O 2.7439 -2.8223 3.7104
N 3.8958 -1.1320 2.6886
C 5.2000 -1.5912 3.1397
C 5.5798 -1.1886 4.5729
O 6.5474 -1.7236 5.1108
C 6.2877 -1.0522 2.2025
O 6.2794 0.3622 2.1753
N 4.8545 -0.2015 5.1453
C 5.2683 0.4097 6.3952
H -7.1060 -2.4100 2.9513
H -5.9895 -1.0667 3.3654
H -5.0709 -3.9617 3.0184
H -0.8419 -4.3367 5.6588
H 0.4577 -4.0151 4.4854
H -1.1485 -6.1845 4.0508
H -0.7760 -5.1058 2.6952
H -3.3676 -5.2885 4.2540
H -3.1035 -4.8897 2.5434
H -1.4004 -2.0705 4.9835
H 1.6178 -0.2282 2.0766
H 1.3169 -1.8301 1.4113
H 7.2494 -1.4172 2.5806
H 6.1273 -1.4549 1.1961
H 5.9735 0.6143 1.2672
H 5.2200 -2.6853 3.1283
H 6.0150 1.1966 6.2281
H 1.9538 7.7924 -3.4864
H -4.2513 4.7026 -4.7884
H -2.5342 4.1169 -2.6206
H -0.2221 7.6363 -3.0317
H 5.7198 -0.3605 7.0225
H 4.3976 0.8386 6.8944
H 4.1080 0.2073 4.6053
H 0.5931 -1.3083 4.2079
H -7.1346 -3.1881 0.7418
H -6.0613 -2.5033 -0.4531
H 3.8505 -0.4217 1.9482
H -5.5533 -3.2910 4.5661
C -10.7553 -5.0996 1.8794
C -9.8285 -4.4033 0.9000
O -8.6966 -4.0444 1.2382
C -11.2783 -4.0841 2.9033
N -10.3438 -4.1513 -0.3244
C -9.6258 -3.3868 -1.3305
C -9.0466 7.0981 2.6118
C -8.6996 5.8269 1.8473
C -8.2766 6.0767 0.3999
N -7.7446 4.9824 -0.2140
O -8.4328 7.1548 -0.1621
H -10.4409 -3.6010 3.4126
H -11.9053 -4.5731 3.6538
H -11.8744 -3.3059 2.4163
H -11.5873 -5.5885 1.3600
H -8.6604 -3.8531 -1.5497
H -7.8989 5.2765 2.3497
H -9.5611 5.1482 1.8246
H -7.5304 4.1276 0.2923
H -7.3960 5.0954 -1.1535
H -9.8121 7.6679 2.0802
H -9.4132 6.8585 3.6145
H -8.1716 7.7467 2.7103
H -10.1737 -5.8769 2.3828
H -9.4401 -2.3623 -0.9924
H -10.2190 -3.3577 -2.2448
H -11.2843 -4.4538 -0.5211
H 4.6959 3.1362 -1.1583
H 2.4781 3.8175 -1.2942

YIH$^+$

C 11.4444 5.2448 -0.5884
C 10.9429 4.0426 -1.3992
C 9.7475 3.3791 -0.7838
C 8.5141 3.1263 -1.3448
N 9.7796 2.8972 0.5119
C 8.5865 2.3607 0.7176
N 7.7805 2.4800 -0.3734
H 10.6844 6.0325 -0.5491
H 11.7575 3.3100 -1.4823
H 10.6972 4.3500 -2.4231
H 8.1059 3.3412 -2.3210
H 8.2509 1.8492 1.6100
H 6.8579 2.0321 -0.4742
C -9.4086 2.1319 -4.5995
C -8.1833 2.7363 -3.8913
C -7.2666 1.6896 -3.2957
C -6.5641 0.8036 -4.1260
C -7.1070 1.5503 -1.9130
C -5.7460 -0.1886 -3.5992
C -6.2823 0.5655 -1.3638
C -5.5983 -0.3128 -2.2115
O -4.7929 -1.3162 -1.7603
H -10.0090 1.5458 -3.8963
H -8.5190 3.4156 -3.0993
H -7.6270 3.3516 -4.6115
H -6.6561 0.8945 -5.2061
H -7.6250 2.2094 -1.2228
H -5.2000 -0.8718 -4.2409
H -6.1764 0.5053 -0.2852
H -4.6400 -1.2650 -0.7795
C -0.2841 -6.7838 -4.8571
C -0.4350 -6.5930 -3.3445
C -1.7999 -6.1375 -2.8564
C -2.0604 -6.0861 -1.4794
C -2.8252 -5.7308 -3.7192
C -3.2834 -5.6469 -0.9798
C -4.0561 -5.2875 -3.2360
C -4.2926 -5.2435 -1.8615
O -5.5273 -4.8349 -1.4389
H -0.4432 -5.8481 -5.4015
H -0.1798 -7.5335 -2.8394
H 0.3166 -5.8691 -3.0019
H -1.2873 -6.3925 -0.7802
H -2.6677 -5.7519 -4.7923
H -3.4534 -5.6125 0.0936
H -4.8400 -4.9640 -3.9120
H -5.5100 -4.6513 -0.4873
C 4.9661 -4.4332 -5.2976
C 5.1777 -5.1763 -3.9998
O 6.2368 -5.7374 -3.7217
C 5.5892 -3.0297 -5.2667
H 5.4292 -5.0253 -6.0926
H 6.6710 -3.0951 -5.1310
H 5.1790 -2.4299 -4.4493
H 5.3913 -2.5021 -6.2045
N 4.1167 -5.1501 -3.1534
C 4.2623 -5.6245 -1.7912
C 2.9595 -5.5160 -1.0101
C 2.3300 -4.1112 -0.9548
C 1.1248 -4.1519 -0.0149
C 3.3273 -3.0194 -0.5425
H 3.3550 -4.5199 -3.3556
H 5.0485 -5.0536 -1.2862
H 3.1654 -5.8461 0.0160
H 2.2218 -6.2188 -1.4210
H 1.9464 -3.8529 -1.9545
H 1.4459 -4.3331 1.0177
H 0.5669 -3.2186 -0.0245
H 0.4251 -4.9458 -0.2968
H 4.1751 -2.9478 -1.2311
H 3.7297 -3.2192 0.4570
H 2.8558 -2.0329 -0.5220
C 9.1823 -5.4410 -0.4842
C 7.8352 -4.7194 -0.6171
C 7.9796 -3.2724 -1.0346
C 7.5720 -2.8569 -2.3067
C 8.5428 -2.3228 -0.1717
C 7.7097 -1.5241 -2.7006
C 8.6826 -0.9918 -0.5605
C 8.2622 -0.5868 -1.8292
H 9.8249 -4.9523 0.2555
H 7.3031 -4.7722 0.3423
H 7.2272 -5.2468 -1.3571
H 7.1653 -3.5971 -2.9859
H 8.8627 -2.6278 0.8213
H 7.3876 -1.2185 -3.6923
H 9.1059 -0.2639 0.1229
H 8.3688 0.4507 -2.1252
C -2.6462 0.6703 0.4716
C -1.4827 0.8535 -0.2212
C -1.3072 2.1276 -0.8581
N -0.1529 2.4768 -1.4060
N -2.3238 3.0413 -0.8739
C -3.4397 2.7705 -0.2145
N -3.6175 1.6257 0.4739
S 1.4351 -0.8646 -3.5205
C 1.1097 0.0004 -2.0652
C -4.5429 3.7792 -0.1922
N 0.0830 -0.6422 -1.4757
C -0.4978 -1.7658 -2.1093
C -1.7135 -2.4242 -1.5462
C 0.1513 -2.0439 -3.2711
C -0.1296 -3.1220 -4.2661
C -0.3618 -0.1438 -0.1568
H 0.6258 1.7972 -1.5236
H 0.0010 3.4379 -1.6963
H -0.6480 -0.9894 0.4683
H 0.5216 0.3280 0.2778
H -5.5180 3.2960 -0.1191
H -4.4340 4.4228 0.6881
H -4.4869 4.4108 -1.0794
H -2.8619 -0.2190 1.0520
H -1.6067 -2.6634 -0.4860
H -1.9221 -3.3515 -2.0782
H -2.5872 -1.7782 -1.6661
H -0.9582 -3.7491 -3.9349
H 0.7436 -3.7683 -4.4154
C 1.7015 -2.5125 7.5440
C 0.9727 -1.1707 7.7012
C 1.5696 -0.0636 6.8512
O 1.4374 -0.3035 5.5425
O 2.0908 0.9391 7.3039
C -8.3642 2.9705 3.8753
C -6.8821 2.7581 3.5497
C -6.5912 2.2242 2.1360
O -7.2493 2.6865 1.1705

O	-5.6401	1.3782	2.0468
H	-8.4958	3.6009	4.7604
H	-8.8746	3.4341	3.0322
H	-6.3991	2.1001	4.2765
H	-6.3544	3.7214	3.6080
C	-0.8925	2.2026	5.4079
C	-0.8962	1.5571	4.0226
C	0.3422	1.8978	3.2629
C	0.4819	2.5640	2.0720
N	1.6075	1.5621	3.7319
C	2.4727	1.9998	2.8247
N	1.8347	2.6272	1.8126
H	0.0072	1.9466	5.9698
H	-0.9925	0.4710	4.1156
H	-1.7712	1.8860	3.4514
H	-0.2453	3.0062	1.4130
H	3.5398	1.8478	2.8405
H	2.2390	2.8377	0.8914
C	5.4478	7.2173	2.8196
C	5.3763	5.9101	3.6182
C	5.8617	4.6463	2.8811
C	5.8028	3.4345	3.8238
C	5.0600	4.3756	1.6012
H	4.7877	7.1951	1.9466
H	4.3399	5.7420	3.9443
H	5.9666	6.0216	4.5372
H	6.9113	4.7986	2.5900
H	4.7843	3.2949	4.2067
H	6.1052	2.5076	3.3255
H	6.4560	3.5792	4.6916
H	3.9893	4.3067	1.8228
H	5.3784	3.4346	1.1450
H	5.1967	5.1664	0.8564
H	-0.3939	-2.7028	-5.2436
H	-8.8582	2.0120	4.0713
H	11.6535	4.9354	0.4389
H	12.3574	5.6688	-1.0213
H	-10.0461	2.9130	-5.0271
H	-9.1021	1.4630	-5.4101
H	-0.9209	3.2944	5.3249
H	-1.7644	1.8852	5.9895
H	6.4661	7.4037	2.4588
H	5.1500	8.0717	3.4366
H	-0.0770	-1.2827	7.4022
H	0.9953	-0.8311	8.7392
H	1.7481	-2.8156	6.4942
H	1.1946	-3.2961	8.1151
H	2.7311	-2.4391	7.9066
H	9.0426	-6.4828	-0.1760
H	9.7140	-5.4374	-1.4410
H	4.6083	-6.6622	-1.8238
H	0.7237	-7.1364	-5.0939
H	-0.9989	-7.5196	-5.2400
H	3.8955	-4.3593	-5.5247
H	1.6684	0.5133	4.9491
H	-4.5283	1.4844	1.0688
C	2.2245	6.8476	-3.0346
C	1.0520	6.2215	-2.3198
O	1.1119	5.0651	-1.8731
N	-0.0487	6.9964	-2.1931
C	-1.3472	6.4276	-1.8603
C	-2.0520	6.1201	-3.1884
O	-2.0077	6.9458	-4.1018
N	-2.6469	4.9176	-3.2709
C	-3.2265	4.4463	-4.5251
C	-3.3721	2.9228	-4.5028
C	-2.0462	2.1496	-4.3800
C	-2.3049	0.6719	-4.0748
C	-1.1588	2.3028	-5.6207
C	3.8173	1.9565	-2.7441
C	2.8823	2.5059	-3.6288
C	2.8897	2.1599	-4.9800
C	3.8339	1.2581	-5.4701
C	4.7681	0.7059	-4.5930
C	4.7641	1.0504	-3.2417
C	3.8520	2.3973	-1.2830
O	2.6307	2.9814	-0.8436

C	4.2293	1.2477	-0.3149
O	5.4808	1.0050	-0.2890
O	3.3537	0.6790	0.3620
H	3.1147	6.7642	-2.4058
H	2.4153	6.2749	-3.9470
H	-1.2082	5.5482	-1.2329
H	-1.9306	7.1673	-1.3040
H	-4.0348	2.6411	-3.6744
H	-3.8937	2.6117	-5.4169
H	-1.4876	2.5630	-3.5345
H	-0.2156	1.7648	-5.4815
H	-1.6544	1.8928	-6.5096
H	-0.9166	3.3509	-5.8265
H	-2.8796	0.2004	-4.8808
H	-2.8786	0.5447	-3.1524
H	-1.3654	0.1250	-3.9689
H	-2.5841	4.7851	-5.3433
H	2.1331	3.1995	-3.2649
H	2.1485	2.5917	-5.6467
H	3.8378	0.9844	-6.5211
H	5.5066	-0.0012	-4.9589
H	5.4860	0.6073	-2.5646
C	-5.1004	-3.0069	3.5820
C	-3.6798	-2.4746	3.7943
O	-3.4683	-1.2798	3.9897
C	-5.9591	-2.0465	2.7361
C	-5.5775	-2.0727	1.2716
N	-6.3955	-2.7344	0.4220
O	-4.5360	-1.5300	0.8651
N	-2.6796	-3.3956	3.8600
C	-1.3220	-2.9596	4.1494
C	-0.6477	-2.3477	2.9165
O	-1.0309	-2.6242	1.7746
C	-0.6006	-4.2785	4.4933
C	-1.2695	-5.2750	3.5326
C	-2.7369	-4.8095	3.4663
N	0.4312	-1.6029	3.2111
C	1.5312	-1.3662	2.2996
C	2.8145	-1.9274	2.9185
O	2.7957	-2.9315	3.6328
N	3.9320	-1.2257	2.6191
C	5.2446	-1.6933	3.0387
C	5.6518	-1.3137	4.4715
O	6.6292	-1.8579	4.9815
C	6.3167	-1.1427	2.0900
O	6.3049	0.2724	2.0894
N	4.9362	-0.3397	5.0788
C	5.3744	0.2458	6.3341
H	-7.0150	-2.3088	2.8463
H	-5.7969	-1.0193	3.0758
H	-5.1076	-4.0100	3.1486
H	-0.8043	-4.5421	5.5366
H	0.4801	-4.1979	4.3545
H	-1.1819	-6.3135	3.8611
H	-0.8159	-5.1871	2.5427
H	-3.3726	-5.3784	4.1569
H	-3.1436	-4.9172	2.4555
H	-1.3246	-2.2398	4.9714
H	1.6450	-0.3104	2.0645
H	1.3316	-1.8999	1.3722
H	7.2842	-1.5132	2.4436
H	6.1447	-1.5310	1.0797
H	5.9888	0.5458	1.1916
H	5.2612	-2.7873	3.0119
H	6.1305	1.0242	6.1702
H	2.0626	7.8957	-3.2931
H	-4.2058	4.9154	-4.6808
H	-2.5594	4.2720	-2.4839
H	-0.1149	7.7930	-2.8147
H	5.8220	-0.5406	6.9440
H	4.5154	0.6790	6.8493
H	4.1828	0.0844	4.5606
H	0.6479	-1.4370	4.1901
H	-7.3010	-3.1079	0.7077
H	-6.1868	-2.6566	-0.5684
H	3.8747	-0.5025	1.8937
H	-5.5500	-3.0906	4.5786

C	-10.9009	-4.9686	1.8477
C	-9.9362	-4.3014	0.8887
O	-8.7816	-4.0347	1.2365
C	-11.2676	-3.9702	2.9510
N	-10.4338	-3.9599	-0.3200
C	-9.6573	-3.2040	-1.2871
C	-8.8999	7.1874	2.8667
C	-8.6111	5.9505	2.0278
C	-8.2345	6.2625	0.5797
N	-7.7275	5.1922	-0.0864
O	-8.3955	7.3689	0.0717
H	-10.3617	-3.6168	3.4487
H	-11.9184	-4.4320	3.6988
H	-11.7883	-3.1012	2.5369
H	-11.7980	-5.3276	1.3308
H	-8.7675	-3.7631	-1.5946
H	-7.8141	5.3481	2.4726
H	-9.4922	5.2974	1.9981
H	-7.5983	4.2851	0.3684
H	-7.4908	5.3129	-1.0590
H	-9.6726	7.7995	2.3953
H	-9.2324	6.9059	3.8706
H	-8.0080	7.8137	2.9632
H	-10.3929	-5.8367	2.2775
H	-9.3302	-2.2478	-0.8673
H	-10.2732	-3.0119	-2.1659
H	-11.3903	-4.1930	-0.5328
H	4.6595	3.1357	-1.1773
H	2.4548	3.8299	-1.2858

TC

C	11.5532	5.4308	-0.6779
C	11.1733	4.1845	-1.4904
C	9.9802	3.4685	-0.9322
C	8.7773	3.1848	-1.5413
N	9.9771	2.9815	0.3622
C	8.7934	2.4129	0.5216
N	8.0289	2.5084	-0.6030
H	10.7469	6.1716	-0.7028
H	12.0315	3.4989	-1.5059
H	10.9745	4.4585	-2.5338
H	8.4010	3.3945	-2.5313
H	8.4311	1.9061	1.4067
H	7.0776	2.1309	-0.6921
C	-9.2409	1.9188	-4.6653
C	-7.8068	2.2711	-4.2385
C	-7.0878	1.1126	-3.5842
C	-6.8027	-0.0493	-4.3160
C	-6.7084	1.1471	-2.2381
C	-6.1614	-1.1350	-3.7335
C	-6.0584	0.0674	-1.6372
C	-5.7689	-1.0779	-2.3901
O	-5.1041	-2.1481	-1.8831
H	-9.8366	1.6130	-3.7989
H	-7.8280	3.1205	-3.5464
H	-7.2447	2.6073	-5.1201
H	-7.0779	-0.0998	-5.3671
H	-6.9190	2.0267	-1.6357
H	-5.9187	-2.0230	-4.3082
H	-5.7543	0.1227	-0.5980
H	-4.7763	-1.9405	-0.9702
C	0.0201	-6.8583	-4.7308
C	-0.0567	-5.7103	-3.7138
C	-1.4080	-5.5957	-3.0453
C	-1.6037	-6.0135	-1.7263
C	-2.5144	-5.0732	-3.7330
C	-2.8504	-5.9140	-1.1087
C	-3.7653	-4.9643	-3.1331
C	-3.9348	-5.3907	-1.8136
O	-5.1892	-5.2920	-1.2642
H	-0.7236	-6.7290	-5.5240
H	0.7161	-5.8449	-2.9500
H	0.1797	-4.7700	-4.2224
H	-0.7661	-6.4148	-1.1641
H	-2.3890	-4.7284	-4.7563
H	-2.9746	-6.2339	-0.0766

H -4.6086 -4.5257 -3.6530
H -5.1319 -5.4750 -0.3167
C 5.2346 -4.4363 -5.2079
C 5.3244 -5.3085 -3.9763
O 6.0890 -6.2721 -3.9165
C 4.5043 -3.0980 -5.0809
H 6.2678 -4.2671 -5.5282
H 4.9527 -2.4795 -4.2978
H 3.4394 -3.2217 -4.8542
H 4.5671 -2.5378 -6.0180
N 4.4819 -4.9574 -2.9820
C 4.5419 -5.5695 -1.6805
C 3.2069 -5.4350 -0.9470
C 2.6239 -4.0087 -0.9125
C 1.3229 -4.0032 -0.1131
C 3.6135 -2.9703 -0.3645
H 4.0070 -4.0722 -3.0464
H 5.3373 -5.1019 -1.0888
H 3.3471 -5.7834 0.0845
H 2.4696 -6.1054 -1.4084
H 2.3590 -3.7158 -1.9406
H 1.5016 -4.2611 0.9360
H 0.8504 -3.0226 -0.1376
H 0.6014 -4.7212 -0.5137
H 4.5420 -2.9239 -0.9432
H 3.8854 -3.2092 0.6701
H 3.1785 -1.9671 -0.3791
C 9.4552 -5.2845 -0.3683
C 8.0469 -5.0092 -0.9203
C 7.9293 -3.6406 -1.5574
C 7.8696 -3.4906 -2.9484
C 7.9190 -2.4871 -0.7611
C 7.8022 -2.2197 -3.5248
C 7.8466 -1.2164 -1.3295
C 7.7920 -1.0805 -2.7189
H 9.7342 -4.5390 0.3828
H 7.3280 -5.0899 -0.0954
H 7.7717 -5.7757 -1.6525
H 7.8737 -4.3786 -3.5724
H 7.9766 -2.5895 0.3187
H 7.7667 -2.1213 -4.6068
H 7.8298 -0.3357 -0.6982
H 7.7500 -0.0912 -3.1634
C -3.2268 1.0301 0.8642
C -1.9812 0.8595 0.3238
C -1.5421 1.8433 -0.6041
N -0.3232 1.7282 -1.1656
N -2.3230 2.9080 -0.9124
C -3.5249 2.9884 -0.3182
N -4.0152 2.0916 0.5461
S 1.2570 -0.0258 -2.5709
C 0.5664 0.5470 -0.9699
C -4.3715 4.1909 -0.6276
N -0.1817 -0.5822 -0.4647
C -0.6868 -1.4004 -1.5104
C -1.8018 -2.3365 -1.1886
C -0.0332 -1.2610 -2.6785
C -0.1996 -2.0027 -3.9615
C -1.0333 -0.2484 0.6757
H 1.3993 0.8216 -0.3234
H 0.0514 2.5085 -1.6908
H -1.5582 -1.1347 1.0232
H -0.3831 0.0769 1.4902
H -4.4353 4.3498 -1.7060
H -5.3746 4.0703 -0.2206
H -3.9087 5.0878 -0.1998
H -3.6248 0.3127 1.5753
H -1.5413 -2.9760 -0.3398
H -2.0467 -2.9685 -2.0392
H -2.6997 -1.7811 -0.9126
H -0.9856 -2.7544 -3.8743
H 0.7298 -2.5121 -4.2508
C 1.9134 -2.3148 7.5874
C 1.0973 -1.0157 7.6677
C 1.7105 0.1116 6.8553
O 1.5921 -0.1009 5.5403
O 2.2353 1.0982 7.3367
C -8.2274 2.9393 3.7935
C -8.4837 2.3393 2.3999
C -7.2893 2.5013 1.4761
O -7.3346 3.1963 0.4559
O -6.2190 1.8614 1.8774
H -9.1193 2.8470 4.4198
H -7.4022 2.4177 4.2843
H -9.3436 2.8111 1.9195
H -8.6920 1.2679 2.4991
C -0.7481 2.3169 5.3543
C -0.7448 1.7066 3.9542
C 0.5016 2.0428 3.2049
C 0.6449 2.6842 2.0008
N 1.7643 1.7000 3.6773
C 2.6320 2.1060 2.7584
N 1.9972 2.7229 1.7370
H 0.1479 2.0515 5.9162
H -0.8580 0.6191 4.0159
H -1.6079 2.0615 3.3811
H -0.0806 3.1030 1.3236
H 3.6972 1.9404 2.7750
H 2.3900 2.9130 0.8071
C 5.5198 7.3770 2.6798
C 5.3968 6.0762 3.4820
C 5.9756 4.8173 2.8072
C 5.8868 3.6205 3.7660
C 5.2822 4.5006 1.4751
H 4.9496 7.3334 1.7462
H 4.3379 5.8889 3.7109
H 5.8953 6.2088 4.4514
H 7.0384 5.0015 2.5920
H 4.8494 3.4596 4.0833
H 6.2440 2.6942 3.3049
H 6.4772 3.7973 4.6719
H 4.2027 4.3762 1.6209
H 5.6770 3.5767 1.0437
H 5.4287 5.2977 0.7389
H -0.4658 -1.3295 -4.7856
H -7.9670 3.9975 3.7283
H 11.7171 5.1541 0.3669
H 12.4646 5.9000 -1.0652
H -9.7378 2.7724 -5.1390
H -9.2460 1.0884 -5.3786
H -0.7795 3.4102 5.2980
H -1.6219 1.9844 5.9251
H 6.5646 7.5812 2.4186
H 5.1457 8.2307 3.2546
H 0.0856 -1.1907 7.2824
H 1.0149 -0.6686 8.7002
H 2.0996 -2.5960 6.5472
H 1.3881 -3.1334 8.0880
H 2.8866 -2.1876 8.0707
H 9.5080 -6.2736 0.0982
H 10.2006 -5.2431 -1.1692
H 4.8209 -6.6162 -1.8183
H 1.0102 -6.9044 -5.1957
H -0.1755 -7.8213 -4.2483
H 4.7834 -5.0479 -5.9992
H 1.8339 0.7082 4.9424
H -5.3762 2.0506 1.2646
C 2.3150 6.8430 -3.1719
C 1.1899 6.1692 -2.4182
O 1.2916 5.0188 -1.9710
N 0.0590 6.9004 -2.2717
C -1.2038 6.2584 -1.9341
C -1.9300 5.9834 -3.2564
O -1.9606 6.8550 -4.1284
N -2.4579 4.7564 -3.3842
C -3.0951 4.3293 -4.6251
C -3.5388 2.8703 -4.5120
C -2.4075 1.8634 -4.2298
C -2.9975 0.5110 -3.8282
C -1.4316 1.7305 -5.4036
C 3.9535 1.9894 -2.8067
C 3.0895 2.5636 -3.7466
C 3.1019 2.1396 -5.0748
C 3.9819 1.1388 -5.4855
C 4.8555 0.5727 -4.5563
C 4.8421 0.9930 -3.2278
C 3.9248 2.4651 -1.3567
O 2.6317 2.9226 -0.9642
C 4.3765 1.3664 -0.3658
O 5.6391 1.2238 -0.2895
O 3.5250 0.7264 0.2823
H 3.2527 6.6901 -2.6332
H 2.4136 6.3579 -4.1482
H -1.0025 5.3528 -1.3631
H -1.8062 6.9391 -1.3247
H -4.2917 2.7882 -3.7176
H -4.0504 2.5896 -5.4418
H -1.8307 2.2226 -3.3714
H -0.6031 1.0668 -5.1380
H -1.9349 1.3171 -6.2868
H -0.9963 2.6950 -5.6868
H -3.5782 0.0699 -4.6459
H -3.6741 0.6217 -2.9772
H -2.2118 -0.1883 -3.5371
H -2.3911 4.4729 -5.4528
H 2.3815 3.3262 -3.4411
H 2.4090 2.5825 -5.7848
H 3.9868 0.8032 -6.5186
H 5.5485 -0.2063 -4.8574
H 5.5090 0.5317 -2.5102
C -4.8715 -2.9917 3.6232
C -3.4797 -2.3711 3.7741
O -3.2749 -1.1701 3.5932
C -5.8175 -2.1609 2.7387
C -5.5250 -2.3673 1.2639
N -6.2865 -3.2635 0.6073
O -4.5915 -1.7638 0.7053
N -2.5059 -3.2070 4.2196
C -1.1634 -2.6955 4.4722
C -0.4130 -2.4284 3.1604
O -0.6827 -3.0521 2.1339
C -0.4752 -3.8586 5.2128
C -1.1146 -5.0920 4.5588
C -2.5734 -4.6714 4.3155
N 0.5669 -1.5128 3.2944
C 1.6610 -1.3602 2.3604
C 2.9643 -1.8095 3.0245
O 2.9923 -2.7390 3.8306
N 4.0554 -1.0872 2.6633
C 5.3900 -1.5072 3.0637
C 5.8184 -1.1032 4.4830
O 6.8174 -1.6209 4.9782
C 6.4212 -0.9334 2.0851
O 6.4146 0.4815 2.1119
N 5.0962 -0.1349 5.0910
C 5.5461 0.4754 6.3302
H -6.8527 -2.4459 2.9510
H -5.6832 -1.1001 2.9624
H -4.8086 -4.0158 3.2451
H -0.7120 -3.8093 6.2810
H 0.6095 -3.8237 5.0921
H -1.0412 -5.9935 5.1721
H -0.6307 -5.2815 3.5982
H -3.2280 -4.9751 5.1437
H -2.9652 -5.1081 3.3921
H -1.2193 -1.7803 5.0669
H 1.7439 -0.3298 2.0134
H 1.4584 -1.9856 1.4931
H 7.4042 -1.3137 2.3860
H 6.1922 -1.2994 1.0777
H 6.1072 0.7774 1.2198
H 5.4444 -2.6006 3.0449
H 6.2849 1.2648 6.1423
H 2.1498 7.9120 -3.3202
H -3.9535 4.9784 -4.8326
H -2.3326 4.0853 -2.6239
H -0.0553 7.6922 -2.8933
H 6.0183 -0.2943 6.9429
H 4.6883 0.8996 6.8548
H 4.3210 0.2621 4.5835
H 0.7477 -1.1528 4.2276

H -7.1310 -3.6846 0.9871
H -6.0996 -3.4047 -0.3816
H 3.9703 -0.4261 1.8857
H -5.2941 -3.0663 4.6334
C -11.0065 -5.0018 1.7905
C -9.9190 -4.5375 0.8429
O -8.7284 -4.6325 1.1559
C -11.0219 -4.0621 3.0001
N -10.3362 -3.9630 -0.3067
C -9.4145 -3.3544 -1.2493
C -8.8259 7.1270 2.7010
C -9.0592 5.8148 1.9635
C -9.7795 5.9904 0.6272
N -9.5382 4.9899 -0.2636
O -10.5464 6.9222 0.4061
H -10.0357 -4.0465 3.4696
H -11.7561 -4.3856 3.7432
H -11.2721 -3.0397 2.6993
H -11.9869 -5.0288 1.3019
H -8.6011 -4.0478 -1.4769
H -8.1226 5.2747 1.8005
H -9.6924 5.1566 2.5724
H -8.7974 4.3079 -0.1099
H -9.9721 5.0657 -1.1709
H -9.7631 7.6803 2.7933
H -8.4185 6.9461 3.7007
H -8.1218 7.7646 2.1578
H -10.7612 -6.0201 2.1058
H -8.9711 -2.4350 -0.8524
H -9.9472 -3.1199 -2.1713
H -11.3269 -3.8795 -0.4703
H 4.6489 3.2834 -1.2399
H 2.4096 3.7727 -1.3923

TCH$^+$

C 11.5562 5.2098 -0.4982
C 11.1539 3.9132 -1.2150
C 9.9204 3.2883 -0.6359
C 8.7080 3.0427 -1.2444
N 9.8781 2.8646 0.6795
C 8.6625 2.3691 0.8507
N 7.9122 2.4609 -0.2823
H 10.7765 5.9713 -0.6058
H 11.9847 3.1985 -1.1471
H 10.9934 4.1053 -2.2829
H 8.3506 3.2275 -2.2462
H 8.2718 1.9092 1.7490
H 6.9763 2.0523 -0.3979
C -9.3491 2.4641 -4.5051
C -8.1076 2.8731 -3.6916
C -7.2826 1.6944 -3.2173
C -6.7441 0.7853 -4.1405
C -7.0394 1.4581 -1.8584
C -6.0068 -0.3191 -3.7298
C -6.2964 0.3564 -1.4271
C -5.7730 -0.5419 -2.3660
O -5.0470 -1.6407 -2.0267
H -10.0086 1.8268 -3.9073
H -8.4203 3.4654 -2.8237
H -7.4862 3.5374 -4.3086
H -6.9002 0.9466 -5.2044
H -7.4239 2.1386 -1.1021
H -5.5850 -1.0147 -4.4480
H -6.1327 0.2127 -0.3650
H -4.7909 -1.6174 -1.0684
C -0.3278 -6.5398 -5.1003
C -0.4379 -5.8655 -3.7229
C -1.8515 -5.6468 -3.2211
C -2.2044 -5.9863 -1.9112
C -2.8392 -5.0499 -4.0203
C -3.4846 -5.7469 -1.4112
C -4.1220 -4.8024 -3.5383
C -4.4517 -5.1594 -2.2282
O -5.7410 -4.9439 -1.8087
H -0.7938 -5.9337 -5.8835
H 0.1071 -6.4617 -2.9833
H 0.0805 -4.8981 -3.7502
H -1.4635 -6.4410 -1.2602
H -2.6027 -4.7613 -5.0403
H -3.7276 -6.0171 -0.3863
H -4.8736 -4.3218 -4.1544
H -5.7938 -5.1202 -0.8585
C 4.9468 -4.2351 -5.4899
C 5.1720 -5.0066 -4.2116
O 6.2374 -5.5686 -3.9563
C 5.7581 -2.9332 -5.5188
H 5.2543 -4.8788 -6.3209
H 6.8274 -3.1504 -5.4694
H 5.5026 -2.2922 -4.6713
H 5.5597 -2.3760 -6.4392
N 4.1139 -5.0118 -3.3660
C 4.2457 -5.5329 -2.0211
C 2.9200 -5.4949 -1.2756
C 2.2668 -4.1052 -1.1739
C 1.0218 -4.2051 -0.2955
C 3.2324 -3.0302 -0.6567
H 3.3422 -4.3908 -3.5563
H 4.9992 -4.9561 -1.4742
H 3.1037 -5.8656 -0.2593
H 2.2141 -6.1973 -1.7397
H 1.9252 -3.7910 -2.1739
H 1.2796 -4.5374 0.7156
H 0.5276 -3.2416 -0.2070
H 0.2968 -4.9168 -0.7035
H 4.0986 -2.8983 -1.3130
H 3.6104 -3.2913 0.3388
H 2.7357 -2.0599 -0.5865
C 9.1734 -5.4477 -0.7334
C 7.8358 -4.7089 -0.8881
C 8.0150 -3.2671 -1.3079
C 7.8640 -2.8901 -2.6476
C 8.3711 -2.2860 -0.3736
C 8.0547 -1.5627 -3.0369
C 8.5523 -0.9577 -0.7560
C 8.3944 -0.5914 -2.0943
H 9.8090 -4.9605 0.0132
H 7.2930 -4.7466 0.0653
H 7.2256 -5.2276 -1.6324
H 7.5952 -3.6498 -3.3743
H 8.4969 -2.5670 0.6684
H 7.9428 -1.2874 -4.0823
H 8.8070 -0.2062 -0.0170
H 8.5390 0.4430 -2.3885
C -3.0372 0.6669 0.7527
C -1.8186 0.5814 0.1543
C -1.4669 1.6031 -0.7791
N -0.2740 1.5467 -1.3842
N -2.3126 2.6376 -1.0422
C -3.5062 2.6345 -0.4475
N -3.8780 1.6854 0.4235
S 1.1915 -0.1514 -2.9867
C 0.6416 0.3533 -1.3143
C -4.5039 3.7048 -0.7646
N -0.0717 -0.7900 -0.8007
C -0.6952 -1.5384 -1.8395
C -1.8534 -2.4102 -1.4838
C -0.1246 -1.3683 -3.0467
C -0.3642 -2.0976 -4.3281
C -0.8157 -0.4931 0.4217
H 1.5127 0.6085 -0.7126
H 0.0511 2.3466 -1.9133
H -1.2808 -1.3979 0.8093
H -0.1009 -0.1513 1.1716
H -4.8962 3.5443 -1.7737
H -5.3378 3.6704 -0.0608
H -4.0282 4.6873 -0.7444
H -3.4013 -0.0466 1.4839
H -1.6053 -3.1081 -0.6800
H -2.2019 -2.9803 -2.3418
H -2.6847 -1.7976 -1.1315
H -1.2063 -2.7849 -4.2305
H 0.5179 -2.6826 -4.6250
C 1.7635 -2.7008 7.4239
C 1.0418 -1.3615 7.6390
C 1.6415 -0.2312 6.8218
O 1.4259 -0.4011 5.5130
O 2.2265 0.7261 7.2922
C -8.2557 3.0120 3.9873
C -6.9717 2.2700 3.6270
C -6.6357 2.2503 2.1324
O -6.9315 3.2362 1.4159
O -5.9776 1.2272 1.7340
H -8.3683 3.0997 5.0746
H -8.2653 4.0105 3.5540
H -6.9787 1.2365 3.9934
H -6.1105 2.7583 4.1138
C -0.7865 2.1106 5.4565
C -0.8670 1.5099 4.0500
C 0.3527 1.8043 3.2387
C 0.4659 2.4432 2.0285
N 1.6204 1.4083 3.6493
C 2.4612 1.7842 2.6939
N 1.8059 2.4271 1.7009
H 0.1205 1.8028 5.9776
H -1.0139 0.4259 4.1095
H -1.7455 1.8975 3.5229
H -0.2728 2.9017 1.3918
H 3.5189 1.5776 2.6609
H 2.1851 2.6363 0.7693
C 5.5983 7.1362 3.0025
C 5.3170 5.8307 3.7578
C 5.7876 4.5403 3.0579
C 5.5895 3.3313 3.9847
C 5.0802 4.3077 1.7161
H 5.0645 7.1741 2.0475
H 4.2383 5.7467 3.9532
H 5.7979 5.8819 4.7434
H 6.8641 4.6376 2.8529
H 4.5307 3.2184 4.2499
H 5.9213 2.3992 3.5156
H 6.1474 3.4554 4.9193
H 3.9948 4.2356 1.8561
H 5.4236 3.3754 1.2592
H 5.2748 5.1151 1.0024
H -0.5812 -1.4126 -5.1559
H -9.1368 2.4778 3.6031
H 11.6857 5.0162 0.5700
H 12.4922 5.6170 -0.8968
H -9.9187 3.3430 -4.8245
H -9.0689 1.9017 -5.4013
H -0.7765 3.2046 5.4074
H -1.6492 1.8077 6.0591
H 6.6676 7.2426 2.7872
H 5.2861 8.0061 3.5898
H -0.0100 -1.4569 7.3420
H 1.0732 -1.0613 8.6888
H 1.8590 -2.9302 6.3594
H 1.2246 -3.5142 7.9185
H 2.7760 -2.6619 7.8362
H 9.0169 -6.4865 -0.4232
H 9.7186 -5.4539 -1.6826
H 4.6280 -6.5561 -2.0867
H 0.7216 -6.6841 -5.3730
H -0.8211 -7.5165 -5.0976
H 3.8818 -4.0151 -5.6274
H 1.6922 0.3971 4.9191
H -4.8769 1.6195 0.8897
C 2.3440 6.9953 -2.8444
C 1.2761 6.1659 -2.1639
O 1.4100 4.9510 -1.9697
N 0.1592 6.8369 -1.7857
C -1.0722 6.1121 -1.5083
C -1.8677 6.0702 -2.8199
O -1.8958 7.0667 -3.5452
N -2.4541 4.8986 -3.1138
C -3.1407 4.7054 -4.3857
C -3.4495 3.2246 -4.6207
C -2.2499 2.2678 -4.4976
C -2.7095 0.8191 -4.6758
C -1.1017 2.6159 -5.4503

C	4.0624	1.9184	-2.7547
C	3.2130	2.4688	-3.7212
C	3.3551	2.1313	-5.0672
C	4.3532	1.2441	-5.4660
C	5.2119	0.7024	-4.5076
C	5.0701	1.0324	-3.1614
C	3.9146	2.3289	-1.2918
O	2.5901	2.7548	-0.9776
C	4.3084	1.1927	-0.3190
O	5.5608	1.0772	-0.1383
O	3.4210	0.4998	0.2202
H	3.3118	6.7836	-2.3843
H	2.4028	6.6842	-3.8918
H	-0.8242	5.1203	-1.1324
H	-1.6469	6.6478	-0.7462
H	-4.2278	2.8963	-3.9198
H	-3.8891	3.1298	-5.6224
H	-1.8602	2.3456	-3.4784
H	-0.2697	1.9181	-5.3094
H	-1.4243	2.5560	-6.4974
H	-0.7181	3.6262	-5.2737
H	-3.0697	0.6401	-5.6970
H	-3.5248	0.5745	-3.9899
H	-1.8884	0.1304	-4.4724
H	-2.5068	5.1136	-5.1792
H	2.4301	3.1581	-3.4263
H	2.6758	2.5575	-5.8002
H	4.4644	0.9780	-6.5133
H	5.9973	0.0150	-4.8036
H	5.7345	0.5932	-2.4256
C	-5.0617	-2.9887	3.4817
C	-3.6347	-2.4637	3.6578
O	-3.3437	-1.2788	3.4868
C	-5.9284	-2.1079	2.5638
C	-5.5978	-2.2711	1.0914
N	-6.4584	-2.9796	0.3334
O	-4.5685	-1.7737	0.5998
N	-2.7205	-3.3596	4.1156
C	-1.3548	-2.9247	4.3863
C	-0.5868	-2.6511	3.0871
O	-0.8813	-3.2251	2.0367
C	-0.7281	-4.1436	5.0922
C	-1.4335	-5.3229	4.4077
C	-2.8693	-4.8194	4.1920
N	0.4443	-1.8009	3.2536
C	1.5480	-1.6774	2.3277
C	2.8473	-2.1202	2.9993
O	2.8711	-3.0169	3.8406
N	3.9461	-1.4454	2.5800
C	5.2759	-1.8577	3.0018
C	5.6167	-1.5702	4.4705
O	6.4929	-2.2271	5.0277
C	6.3256	-1.1483	2.1358
O	6.2168	0.2591	2.2580
N	4.9831	-0.5178	5.0415
C	5.4620	0.0543	6.2867
H	-6.9821	-2.3541	2.7270
H	-5.7701	-1.0508	2.7978
H	-5.0604	-4.0226	3.1242
H	-0.9615	-4.1107	6.1617
H	0.3573	-4.1613	4.9741
H	-1.4016	-6.2451	4.9932
H	-0.9678	-5.5073	3.4370
H	-3.5269	-5.0984	5.0267
H	-3.3002	-5.2198	3.2699
H	-1.3635	-2.0280	5.0113
H	1.6412	-0.6548	1.9583
H	1.3475	-2.3215	1.4731
H	7.3116	-1.4853	2.4765
H	6.1987	-1.4515	1.0903
H	5.9698	0.5893	1.3577
H	5.3856	-2.9412	2.8885
H	6.2714	0.7755	6.1134
H	2.1454	8.0676	-2.7990
H	-4.0688	5.2896	-4.3996
H	-2.3390	4.1100	-2.4752
H	-0.0103	7.7224	-2.2494
H	5.8523	-0.7569	6.9019
H	4.6362	0.5495	6.8008
H	4.3955	0.0412	4.4421
H	0.6256	-1.4480	4.1896
H	-7.3441	-3.3514	0.6723
H	-6.2647	-3.0300	-0.6620
H	3.8606	-0.7821	1.8057
H	-5.5131	-3.0125	4.4816
C	-11.1553	-4.7774	1.6244
C	-10.0924	-4.2370	0.6892
O	-8.8964	-4.2905	0.9935
C	-11.2424	-3.8615	2.8504
N	-10.5371	-3.6482	-0.4424
C	-9.6430	-2.9746	-1.3683
C	-8.7485	7.2657	3.1202
C	-8.4506	6.1506	2.1273
C	-8.1000	6.6295	0.7203
N	-7.5607	5.6453	-0.0498
O	-8.3020	7.7732	0.3231
H	-10.2635	-3.7886	3.3302
H	-11.9596	-4.2472	3.5801
H	-11.5566	-2.8525	2.5656
H	-12.1281	-4.8577	1.1262
H	-8.8383	-3.6481	-1.6767
H	-7.6356	5.5085	2.4726
H	-9.3219	5.4906	2.0258
H	-7.3774	4.7229	0.3373
H	-7.3128	5.8670	-1.0013
H	-9.5329	7.9237	2.7369
H	-9.0708	6.8512	4.0804
H	-7.8636	7.8854	3.2940
H	-10.8498	-5.7834	1.9259
H	-9.1871	-2.0865	-0.9186
H	-10.2066	-2.6731	-2.2517
H	-11.5308	-3.6001	-0.6014
H	4.6172	3.1506	-1.0950
H	2.4090	3.6386	-1.3531
